# Enzymatic Metal–Hydrogen Atom Transfer with a Cobalt Protoporphyrin Cofactor

**DOI:** 10.1021/jacs.5c19000

**Published:** 2026-01-30

**Authors:** Carly L. Masonheimer, Michael J. Rourke, Reece S. Gardner, Ryan L. Hall, Lydia J. Perkins, Thomas C. Brunold, Andrew R. Buller

**Affiliations:** † Department of Chemistry, University of Wisconsin—Madison, Madison, Wisconsin 53706, United States

## Abstract

Introduction of unnatural cofactors in biocatalysis may open the door to new reactive enzymatic intermediates, and in turn, new biochemical reactions. Here, we employed a de novo biosynthesized, non-natural cofactor, cobalt protoporphyrin IX (CoPPIX), to generate a mononuclear cobalt hydride in the active site of CYP119, a model P450 enzyme. We show that this cobalt hydride intermediate engages in metal–hydrogen atom transfer (MHAT) reactivity, a well-studied and highly utilized reactivity pattern in synthetic chemistry, but which is not known to operate in Nature. We paired convenient in vivo CoPPIX biosynthesis with a colorimetric screen to enable rapid directed evolution. Thus, we engineered CYP119 for MHAT-mediated deallylation of nitrophenols, with the goal of generating not one prolific catalysis, but a diverse set of MHAT-compatible enzymes. Because many silanes hydrolyze quickly, we additionally sought enzymes that accelerate metal-hydride formation from a more persistent silane. This evolution yielded 80 diverse active site recombinants that catalyze MHAT. Serendipitously, we found many variants reduced the aromatic ring of the colorimetric probe, a reaction not previously known. Detailed mechanistic analysis established this is a radical, MHAT-mediated reductive dearomatization that occurs efficiently under aerobic conditions, albeit on a limited suite of nitrophenyl ethers. These results lay a framework for further engineering and study of biocatalytic MHAT and the unique role of metal substitution to tune reactivity.

## Graphical abstract

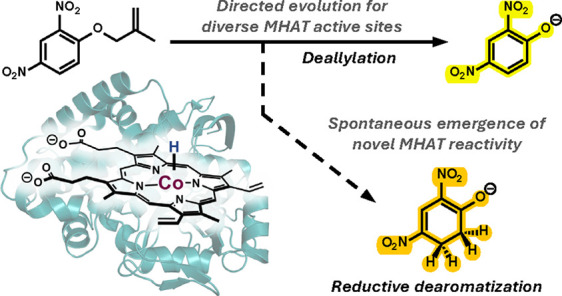

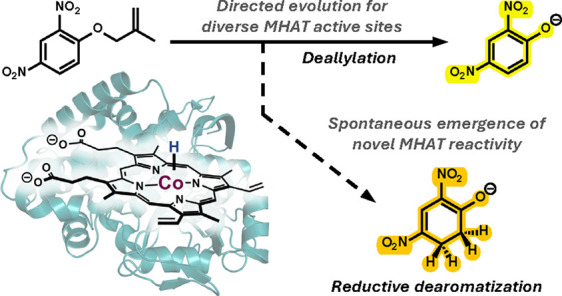

## Introduction

The discovery of new-to-nature enzymatic intermediates has dramatically expanded the scope of chemical transformations accessed via biocatalysis. Successful strategies to generate new-to-nature enzymatic reactions often rely on high-energy substrates, such as diazo compounds, or increasing reactivity through photoexcitation.^1^ Another attractive strategy for translating advances from organic synthesis to enzymes has been the incorporation of non-natural cofactors, often through chemical conjugation or noncanonical amino acid incorporation.^2,3^ Recently, we developed a simple procedure to induce biosynthesis of a non-native cobalt-substituted protoporphyrin (CoPPIX) cofactor in *E. coli*, which is isoelectronic with broadly utilized cobalt-salen catalysts.^3,6,7^ Inspired by these advances, we sought to evolve CoPPIX-containing enzymes for metal–hydrogen atom transfer (MHAT) chemistry.^5^

Mononuclear metal hydrides are versatile species in synthetic chemistry. By altering the metal identity, oxidation state, and ligand environment, these species can exhibit protic, hydridic, or hydrogen atom-like character.^8,9^ Hydridic metal-hydrides, which operate via 2-electron chemistry, have found wide application in the reduction of ketones and imines. The 1-electron, MHAT reactivity of some metal hydrides is mild and chemoselective, particularly those based on Fe­(acac)_2_ or Co-salen scaffolds.^4,10,11^ In these reactions, MHAT to an alkene forms a carbon-centered radical intermediate that is subsequently leveraged for a variety of transformations, including alkene hydrofunctionalization,^12–14^ carbon–carbon bond formation,^15–19^ and many other catalytic applications.^4,20–24^

Natural enzymes that react via monomeric metal hydrides to perform native functions are not known, although a cobalt hydride (Co–H) intermediate is hypothesized to form during the photosensing cycle of the adenylsylcobalamin-dependent transcription factor CarH.^25^ Several groups have leveraged enzyme-based systems to generate metal hydrides implicated in abiological ketoreductases,^9,26^ styrene alkylation,^27^ alkene hydrogenation,^28^ and hydrogen evolution catalysts.^29,30^

Recent proposals also describe enzymes that form monomeric metal hydrides and react via MHAT. For instance, mononuclear nonheme iron­(II) and 2-oxoglutarate-dependent oxygenases are hypothesized to catalyze a nonstereoselective Mukaiyama hydration (Figure 1A).^31^ Recently, a Co-salen catalyst was noncovalently appended to streptavidin and performed MHAT radical cyclizations,^32^ paralleling synthetic developments with Co-salen (Figure 1B). Because the Co-salen-streptavidin catalyst is not fully biologically derived, classical directed evolution and subsequent synthetic implementation is cumbersome, albeit still effective.^33^ Concurrent with the preparation of this manuscript, examples of native heme-containing cytochrome P450 enzymes repurposed for MHAT transformations, including asymmetric radical cyclizations^34^ and hydrogenations of unactivated alkenes (Figure 1C–D) have been reported.^35^

**Figure 1:**
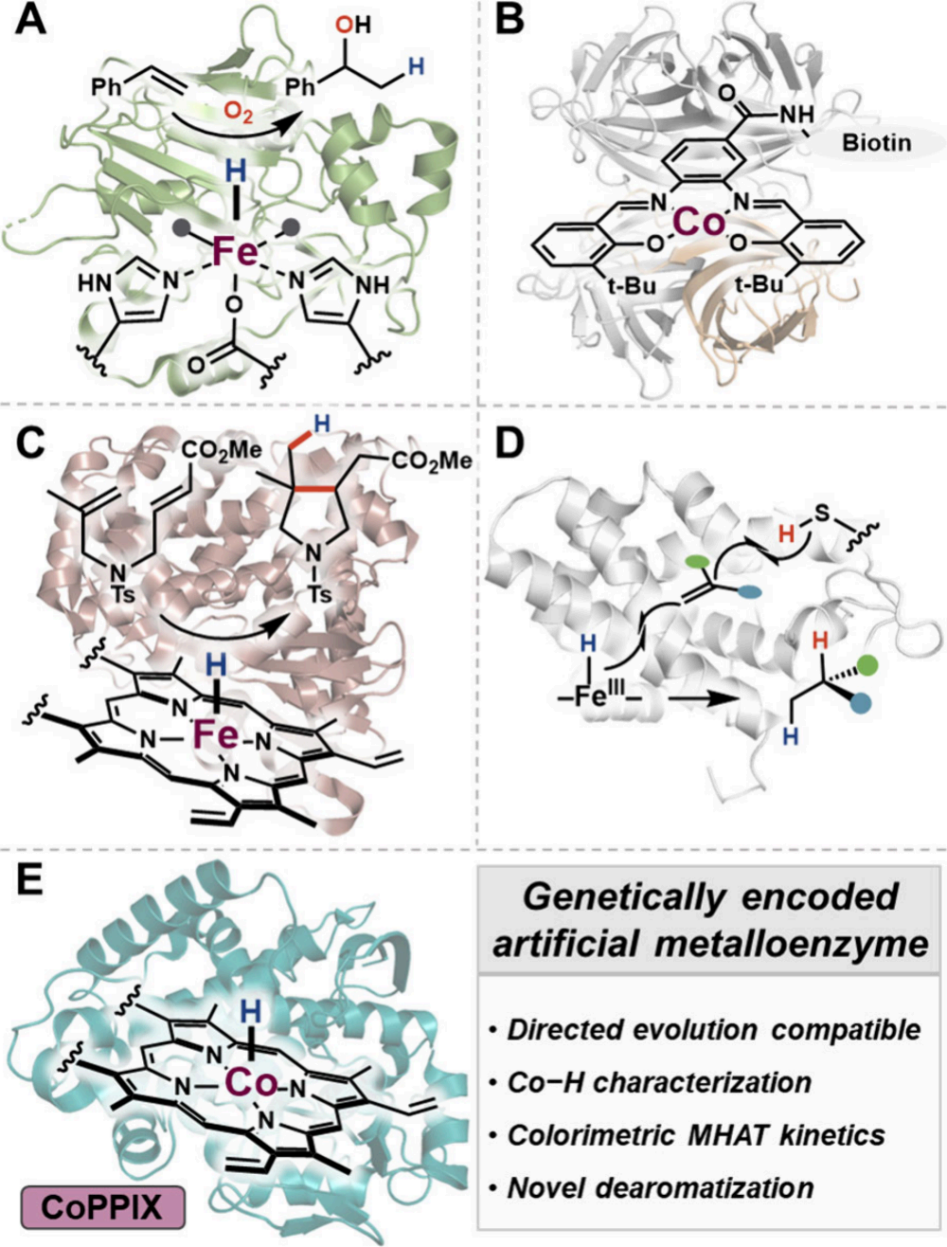
Metal–hydrogen atom transfer in biocatalysis. (A) Fe/2OG enzyme catalyzed Mukaiyama hydration (PDB: 6L86). (B) Biotin–streptavidin Co–salen metalloenzyme employed in MHAT (PDB: 6J6J). (C) Native heme Fe–hydride catalyzes MHAT cyclization (PDB: 2IJ2). (D) Self-contained MHAT hydrogenation with cysteine (PDB: 7UTE). (E) This work: biosynthesis of CoPPIX provides access to a new-to-nature cobalt-hydride (PDB: 1I07).

Previously and in collaboration with the Burstyn group, we developed a method for producing CoPPIX-substituted hemoproteins in *E. coli* by supplementing cultures with Co^2+^ at induction.^36,37^ This discovery afforded unprecedented access to study and develop the chemistry of an artificial metalloenzyme. We hypothesized that CoPPIX, which is isoelectronic with the premier synthetic MHAT catalyst, Co-salen, could react via hydrogen atom transfer within a suitable enzyme environment. Furthermore, the reactivity of the key cobalt-hydride intermediate might be readily tuned through directed evolution. Indeed, it has been shown that the cofactor’s electronic environment in hemoproteins can be adjusted by mutating the axial ligand and surrounding residues.^38–40^

Here, we demonstrate that Co-substituted P450 enzymes can be leveraged for MHAT reactivity (Figure 1E) and can be engineered to exhibit improved deallylation activity. During evolution for deallylation, a new reaction emerged: reductive dearomatization of electron-deficient phenol ethers to cyclic 1,3-dienes. This study provides experimental evidence of tunable enzymatic MHAT reactions and establishes a framework for further mechanistic inquiry and elaboration through directed evolution.

## Results and Discussion

### Reaction of Co-Substituted CYP119 with Phenylsilane

We chose a model P450 enzyme, CYP119 *from S. acidocaldarius*, to explore the reactivity of the CoPPIX cofactor. This thermostable enzyme has multiple solved structures^41,42^ and has been engineered for native oxidation and non-native carbene transfer reactions.^43,44^ CoPPIX-bound CYP119 (CoCYP119) can be prepared in minimal, Fe-deficient media, yielding >99% Co-loaded protein that retains axial thiolate ligation as evidenced by electron paramagnetic resonance (EPR) spectroscopy analysis of Co­(II)­CYP119.^36^ For the present studies, we employed a modified method for CoPPIX production in rich media,^37,45^ resulting in a titer of ∼71 mg CYP119 L^–1^ culture that is >95% Co-loaded, evidenced by ICP-MS and UV–vis spectroscopic analysis of the cofactor content (Figure S1).

We first tested reactivity of CoCYP119 with phenylsilane (PS) and dimethylphenylsilane (DMPS), common hydride sources,^9,46,47^ by monitoring with ultraviolet–visible (UV–vis) spectroscopy. Previous UV–vis and EPR analyses support Co­(III) as the resting oxidation state for CoCYP119 (Figure 2A).^36^ Upon addition of 10 mM PS to CoCYP119 at pH 6.0, the UV–visible spectrum showed an unusual ‘split Soret’ that slowly disappeared (Figure 2B). We observed similar, but less intense spectral changes when 0.5 mM DMPS was added to CoCYP119 (Figure S2). As a control, we measured spectra of free Co­(III)­PPIX and its reaction with 10 mM PS (Figure S2). No drastic changes in the Soret were observed; instead, the spectrum resembled that of the dithionite-reduced Co­(II)­PPIX species (Figure S3). During measurements with both CoCYP119 and CoPPIX, we observed bubbles in the cuvette and analysis by gas chromatography confirmed formation of H_2_ (Figure S4).

**Figure 2:**
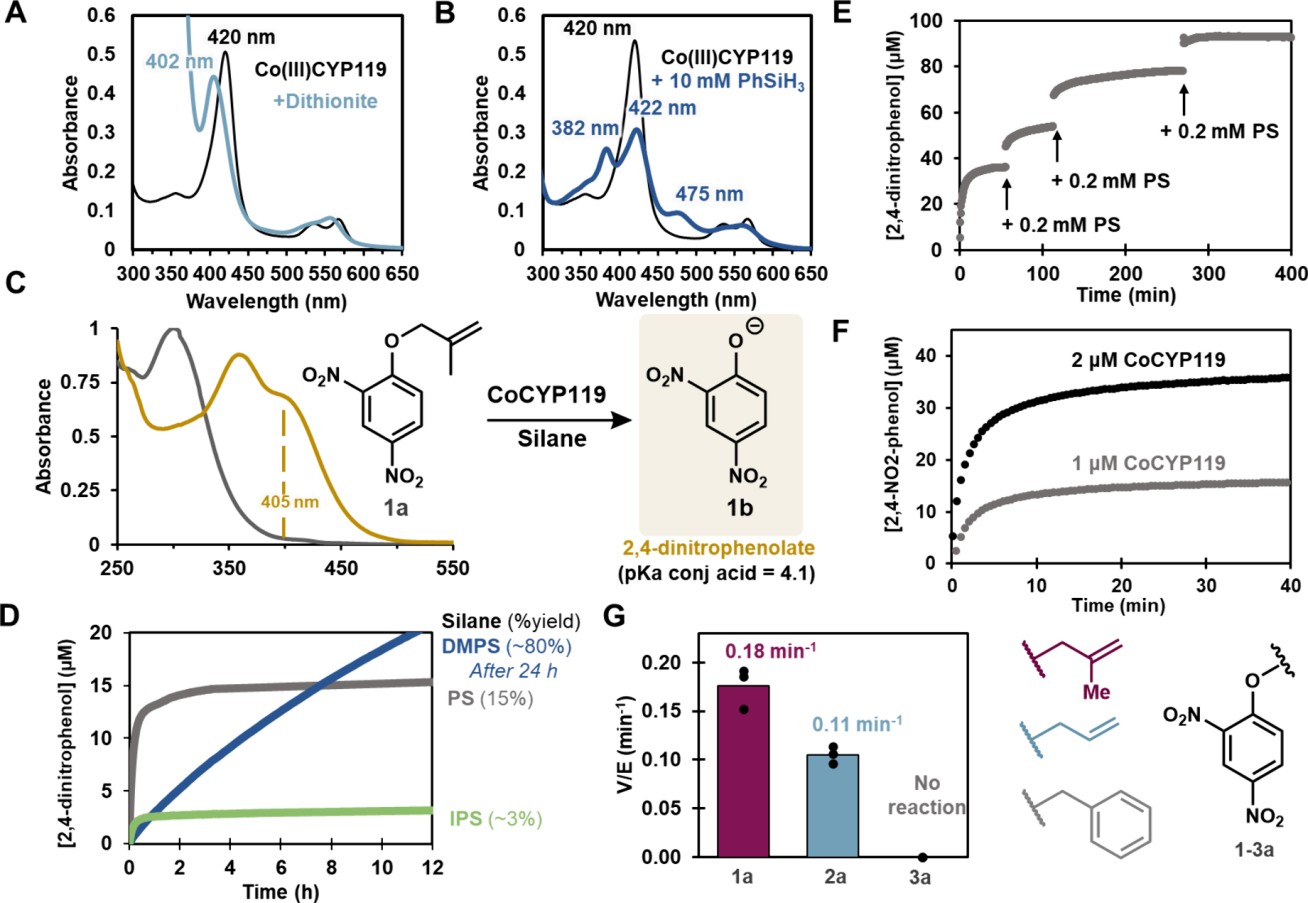
Spectral characterization and metal–hydrogen atom transfer activity of CoCYP119. (A) UV–vis spectrum of as-isolated 20 μM CoCYP119 in 200 mM potassium phosphate buffer, pH 6.0 (black). Reduced CoCYP119 spectrum (light blue) following the addition of a few crystals of solid sodium dithionite at 25 °C. (B) UV–vis spectrum of as-purified 20 μM CoCYP119 in 200 mM potassium phosphate buffer pH 6.0 (black). Addition of 10 mM PS in DMSO at 25 °C (dark blue). Difference spectra can be found in Figure S2. (C) Deallylation of **1a** (black trace, 100 μM) to **1b** (yellow trace, 100 μM) serves as a model deallylation reaction. The amount of product is quantified by the change in absorbance at 405 nm. (D) Deallylation of **1a** (100 μM) by CoCYP119 (1 μM) in the presence of 0.5 mM PS (gray), 0.5 mM DMPS (blue), or 0.5 mM iPrOPS (green). (E) Iterative addition of PS (0.2 mM per addition) to CoCYP119 (2 μM)-catalyzed deallylation of **1a** (100 μM) yields corresponding increases in product formation over the course of 8 h. (F) Halving the concentration of CoCYP119 under otherwise identical reaction conditions to panel C halves the yield of the reaction. (G) Initial rates from reaction of CoCYP119 (1 μM) and DMPS (200 μM) with various allyl-protected dinitrophenolate compounds (50 μM).

To identify candidate species responsible for the UV–vis spectrum with a split Soret band (Figure 2B, blue trace, CoCYP119 + PS), we turned to density functional theory (DFT) computations. First, several models of the Co­(III)­PPIX cofactor in the resting CoCYP119 enzyme were subjected to DFT geometry optimizations, and the corresponding UV–vis spectra were computed using time-dependent DFT (TD-DFT).^48^ The best agreement between the experimental (Figure 2B, black trace) and TD-DFT computed absorption spectra was observed for the Co­(III)­PPIX model with axial thiolate/hydroxide ligation (Figures S5A, S6). This methodology was applied to viable models of the intermediate formed in the reaction of CoCYP119 with PS. The TD-DFT computed spectrum for the Co­(III)­PPIX model with axial thiol/hydride coordination was found to reproduce nearly quantitatively the split Soret band pattern observed experimentally (Figure S7). These results are all consistent with CoCYP119 reacting with PS to generate a cobalt-hydride intermediate. While definitive characterization lies beyond the scope of this study, the formation of a Co–H species is supported by silane consumption, H_2_ evolution, and precedent from Co-salen systems.^4^

To enable high-throughput and continuous kinetic analyses we developed a colorimetric probe that releases nitrophenolate upon deallylation, a reaction known to occur through MHAT (Figure 2C).^49^ As CYP119 is derived from an acidophilic organism,^50^ we elected to use a 2,4-dinitrophenyl-1-butenylether (**1a**) as a probe with improved sensitivity at pH 6.0 compared to the more standard 4-nitro analog. Reaction of **1a** with CoCYP119 and PS resulted in rapid deallylation to 2,4-dinitrophenol (**1b**), albeit in modest yield (∼15%) (Figure 2D). Isobutene was observed as a byproduct of this reaction, consistent with a MHAT-mediated deallylation reaction mechanism (Figure S8).

### Inefficient Cobalt-Hydride Formation Limits CoCYP119-Mediated MHAT

Initial reactions with CoCYP119 proceeded to low yield and we sought to identify the impediments to the reaction. Reaction yield was improved by adding several equivalents of PS over the course of the reaction (Figure 2E), confirming that the enzyme is active under these conditions and that the low initial yield was due to PS decomposition through a competing process. To test if this parasitic pathway was enzyme-mediated, such as protonation of the Co–H species, we assessed the effect of catalyst loading on the yield. Doubling the enzyme concentration under PS-limited conditions doubled the yield (Figure 2F), indicating that a nonenzymatic process, such as hydrolysis, competes directly with the enzymatic reaction. Recent work by Azam et al. and Hodur et al. supports the conclusion that PhSiH_3_ hydrolysis limited our reaction system. Both studies demonstrated that PhSiH_3_ undergoes hydrolysis under aqueous conditions.^51,52^ Substitution of PS with RuebenSilane (iPrOPS) yielded a similarly fast, but low-yielding reaction (Figure 2D).^47^ We next tested reactions with dimethylphenylsilane (DMPS). Consistent with inefficient Co–H formation with this substrate observed via UV–vis spectroscopy, deallylation proceeded slower with DMPS than with PS. However, deallylation reactivity with DMPS persisted for several hours, eventually resulting in >80% yield (Figure 2D).

We compared the rates of CoCYP119-mediated deallylation of **1a** and the less substituted allyl ether (**2a**) with DMPS as the hydride source. The more substituted alkene **1a** reacted roughly 2-fold faster than **2a** (Figure 2G). The corresponding benzyl ether substrate (**3a**) did not react, which is consistent with a radical deallylation mechanism. As controls, we tested the impact of the metal identity and the protein scaffold on the deallylation reaction. Reaction of CoPPIX with DMPS in the absence of enzyme gave rapid deallylation that quickly stopped (Figure S9). In contrast, neither the heme cofactor alone nor FeCYP119 catalyzed the deallylation of **1a** (Figure S9). While concurrent studies affirm that heme is competent for biocatalytic MHAT,^34,35^ these data demonstrate that simple metal substitution has a major effect on MHAT reactivity.

### Directed Evolution for Improved MHAT Using a Colorimetric Probe

As we considered future directions for biocatalytic MHAT, we recognized that slow metal hydride formation and silane instability would be an impediment to any future reactions. Biocatalytic deallylation itself would likely have only niche synthetic utility. However, we envisioned using this reaction as a convenient tool for high-throughput screening to acquire a diverse set of variants that can accelerate MHAT catalysis.

We targeted nine active site residues in CoCYP119 for site-saturation mutagenesis (SSM), encompassing positions on both faces of the cofactor (Figure S10). Variants were expressed in rich media with added Co^2+^,^37^ and assayed with standard 96-well directed evolution procedures. Enzymes were prepared as clarified, heat-treated lysate to remove unstable variants and limit background absorbance for UV–vis assays. We screened libraries using a 10:1 ratio of PS to **1a**.

Enzymatic MHAT reactivity was highly sensitive to active site mutations. Although axial residue mutations and metal substitution in P450s activate non-natural carbene and nitrene transfer reactions,^39,43,53^ mutation at the analogous Cys317 in CoCYP119 yielded no active MHAT variants (Figure S11B). As CYP119 folds and can bind heme with every possible axial substitution,^54^ the lack of activity exhibited by axial ligand variants indicates that the Cys ligand is privileged for efficient MHAT under these conditions. While other proximal face mutations were neutral or deleterious (Figure S11), activating mutations were found in all SSM libraries screened in the substrate binding pocket (Figure S12).

We validated screening results for two of the most activated variants, L69H and V254D. Each mutation substantially enhanced deallylation activity compared to wild-type (WT) CoCYP119, but boosts in initial velocity were greater than the increases in total turnover number (TTN), consistent with nonenzymatic decomposition of PS (Figure 3A). Revisiting DMPS as a hydride source, V254D accelerated deallylation approximately 4-fold compared to WT. Combined with mechanistic analysis of the parent enzyme, these data establish that an enzyme scaffold can accelerate Co–H formation with a sterically hindered, less hydridic silane (Figure 3A).^55^

**Figure 3:**
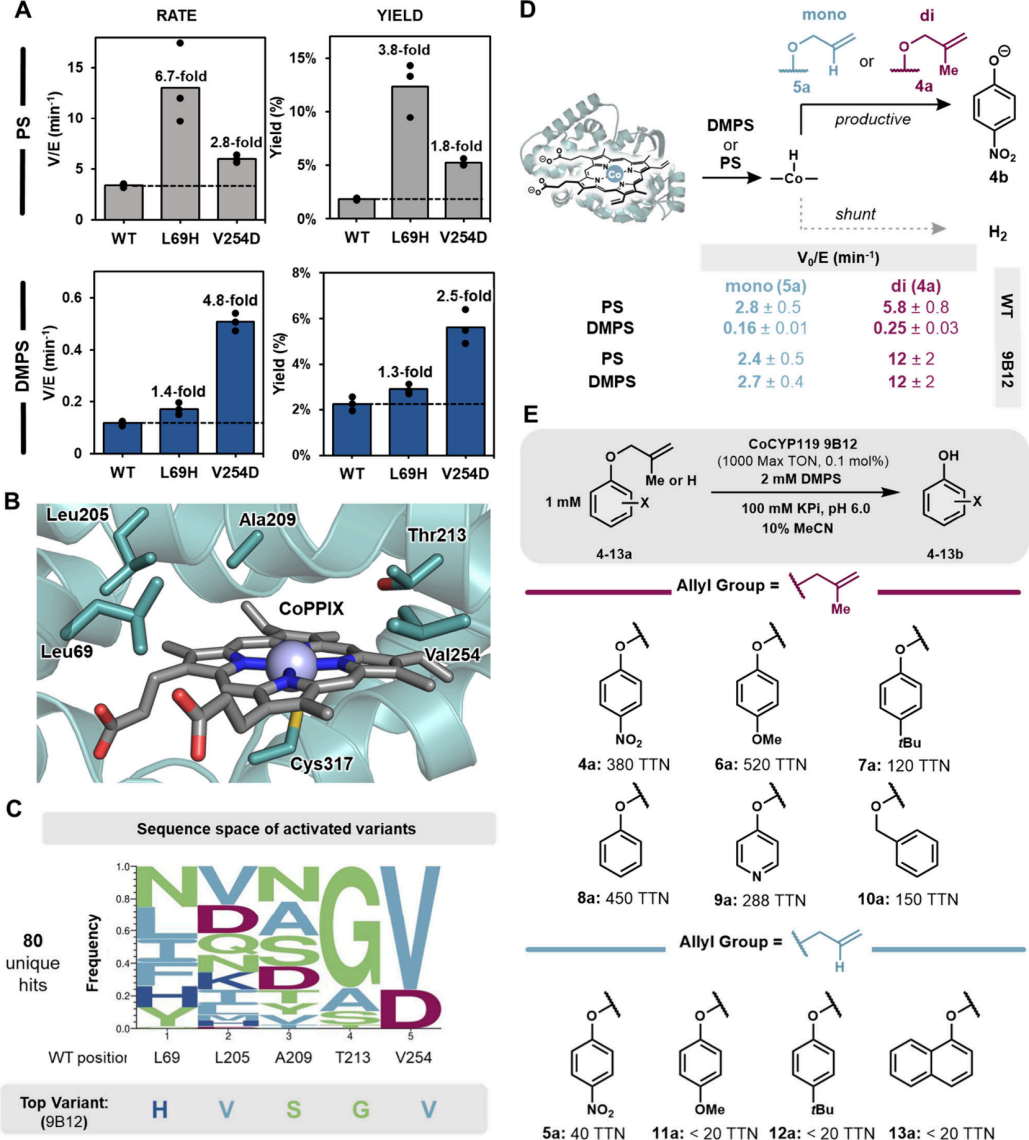
Deallylation activity of engineered CoCYP119. (A) Comparison of initial rate and yield for WT CoCYP119 to SSM variants V254D and L69H in triplicate. CoCYP119 or variant (purified, 0.1 μM) was reacted with 200 μM PS (gray bars) or DMPS (blue bars) and 100 μM **1a**. Bars represent the ranges of the triplicate measurements. (B) Active site model for CoCYP119 (PDB: 1IO7). (C) WebLOGO displaying residues most prevalent in the top 80 variants from recombination. (D) Reaction scheme and initial velocity measurements for reaction of substrate **5a** and **4a** (100 μM) with DMPS or PS (0.2 μM), using either WT CoCYP119 or 9B12 (1–2 μM enzyme). Rate values are reported with the standard deviation of triplicate measurements. (E) Substrate scope for deallylation of phenols by 9B12.

We next recombined neutral and activating mutations at five positions (Figure 3B,C) and screened this library for deallylation using one equivalent of DMPS. The most active variants from recombination were combined into a single plate consisting of 80 unique sequences. When these enzymes were assayed in parallel with **1a** and DMPS, we observed a peculiar brownish color in several wells that changed to orange as the reaction progressed (Figure S13A). Subsequent analysis showed this orange product resulted from a new, unexpected reaction: direct HAT to the highly electron deficient aromatic ring (*vida infra*, “Discovery of MHAT mediated reductive dearomatization”). To assess evolutionary outcomes for deallylation specifically, we rescreened the recombinant master plate with *p*-nitro-O-2-methyl-allyl ether (**4a**), which cleanly yielded the expected deallylation product **4b** and revealed activated enzymes with diverse sequences (Figure S13B, Table S4). As a control, we assayed deallylation of **4a** using standard screening conditions for the iron-containing enzymes and did not observe any deallylation product. These results affirm a distinct role for metal substitution in evolution of new activity (Figure S14).

The absence of detectable deallylation activity by the iron enzyme under screening conditions is notable given recent demonstrations that Fe and heme hydrides can perform MHAT chemistry. Low levels of activity were observed, however, using purified Fe9B12 (Figures S16).^31,34,35^ Our DFT calculations comparing iron and cobalt hydrides reveal consistently shorter and stronger Co–H bonds relative to their Fe–H counterparts (See Supporting Information). We infer that this increased bond strength renders the cobalt hydride less susceptible to protonation and other off-cycle decomposition pathways, thereby preserving the productive MHAT catalyst. The advantage of cobalt hydrides over their iron counterparts may also arise from differences in how the two metals support the sequence of steps required for productive deallylation and/or subsequent electron transfers (Figure S17).^56^

### Mechanistic Interrogation of Improved MHAT Activity in CoCYP119

We leveraged continuous kinetic analysis of the deallylation reaction to uncover how directed evolution improved MHAT reactivity. The most promising catalyst from evolution, 9B12, exhibited a substantial improvement in both initial rate and TTN compared to the WT enzyme for deallylation of **4a** (Figures 3D, S15–16). With WT CoCYP119, deallylation is nearly an order of magnitude slower with DMPS than with PS, consistent with Co–H formation being rate-limiting. Satisfyingly, the reaction rate with 9B12 and DMPS was accelerated nearly 50-fold relative to WT, such that deallylation of **4a** with PS or DMPS occurred at the same rate.

We next considered the efficiency of the second step of the deallylation reaction: HAT to the alkene substrate. We measured rates for deallylation of mono- (**5a**) or disubstituted (**4a**) alkene substrates (Figure 3D). Intriguingly, with both enzymes we observed a modest decrease in rate with **5a**, regardless of the silane used. These effects could result from either a shift in the rate-determining span when a monosubstituted alkene is used or they may reflect competition with a shunt reaction, such as formation of H_2_. Together these data illustrate that the increases in MHAT activity observed with 9B12 are driven primarily by increased rate of Co–H formation. Importantly, evolution of 9B12 provided increased reactivity with a more well-behaved substrate (DMPS), which drastically increases efficiency and synthetic utility of the reaction.

Because the selective pressure during directed evolution was principally for an increased rate of Co–H formation with DMPS, we hypothesized that the resulting catalyst would have ‘generalist’ properties and react with a range of allyl substrates. Under standard conditions (2 eq. of DMPS, 0.1 mol % 9B12) parent **4a** reacted to 38% yield, corresponding to 380 turnovers. Substitution at the *p*-position had minimal impact (**6**–**9a**, Figure 3E). Deallylation of a nonaryl ether (**10a**) would not benefit from resonance stabilization of an oxy radical, but was moderately well-tolerated by the enzyme, which may be explained by a variety of mechanisms (Figure S17). Monosubstituted allyl groups (**11**–**13a**) all reacted, albeit slowly and to low yield, consistent with kinetic observations with **5a**.

### Discovery of MHAT Mediated Reductive Dearomatization

At the outset, our approach to engineering was to generate many diverse variants of CoCYP119 that are all compatible with MHAT, and could be screened for new modes of reactivity. Serendipitously, color changes during screening for deallylation with **1a** suggested that a new MHAT reaction had already emerged (Figure S18). LC-MS analysis indicated that, in addition to the expected deallylation product (**1b**), a new product was forming with +2 amu relative to 2,4-dinitrophenolate **1b** (Figure S19).

We considered whether phenolate **1b** underwent subsequent reaction after deallylation. However, when assayed independently, **1b** was not reactive. When 9B12 was reacted with 2,4-dinitroanisole (**14a**), which lacks the reactive alkene handle, we observed the same product as was formed with **1a** (Figures 4A, S19). Isolation of this material revealed formation of a dearomatized 1,3-diene product (**14b**) in 54% yield. The structure of this compound was confirmed through X-ray crystallography (Figure 5A). We hypothesized that this product forms through reductive dearomatization to yield methyl ether **14d**, which undergoes spontaneous hydrolysis to form **14b**. MHAT-mediated alkene reduction is well-known, but reduction of aromatic rings via MHAT has not been previously reported.^57^ Several potential mechanisms could account for this reactivity. We began a mechanistic inquiry by confirming the source of each hydrogen in the product. Reactions with D_1_-DMPS incorporated deuterium at the 5-position (**5D-14b**) of **14b**. Reactions in deuterated buffer resulted in deuterium incorporation at the 6-position (**6D-14b**) (Figure 5B), indicative of protonation at the 6-position. Deuterium exchange was not observed at these positions upon exposure of **14b** to D_2_O. These patterns could arise from either a radical (H atom) or two-electron (hydride) mechanism. In the case of a radical mechanism, incorporation of deuterium from the buffer indicates a reductive radical-polar crossover,^58^ providing a possible pathway for Co­(II/III) turnover. We conducted reactions under anaerobic conditions which, to our surprise, had a marginal impact on the yield of the transformations (Table S6). Whereas previous MHAT-based hydrogenations typically required exogenous oxidants and thiols to enable efficient cooperative catalysis,^59–61^ we observed no improvement with diverse additives, and often a deleterious effect (Tables S7–8). We therefore returned to aerobic conditions for further kinetic analysis.

**Figure 4:**
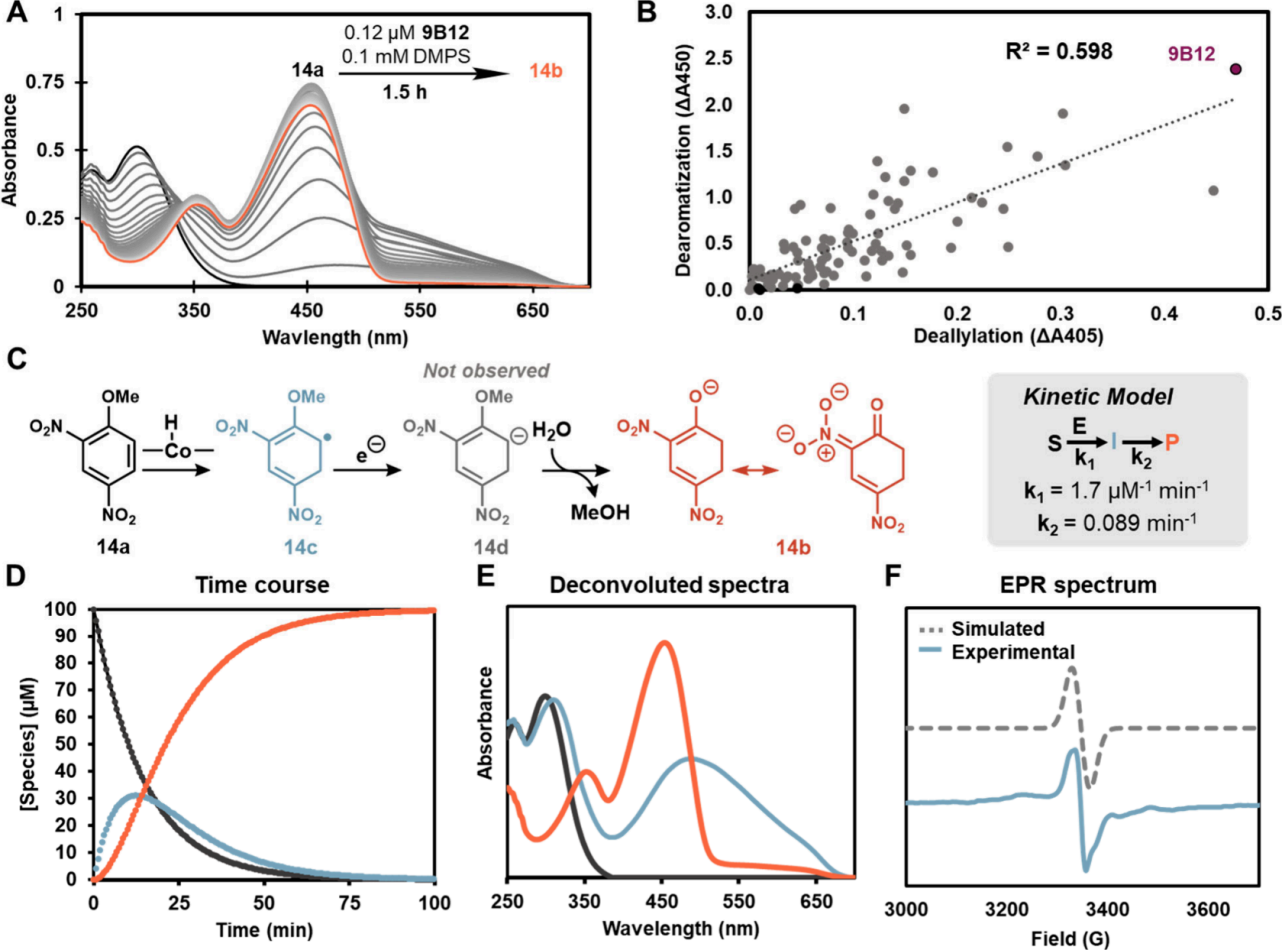
Reductive dearomatization proceeds through a radical mechanism. (A) UV–vis time course spectra for dearomatization of **14a** with 9B12. (B) Correlation plot for the top hits from the recombination library screened against dearomatization (*y*-axis) and deallylation (*x*-axis). Each point represents a unique protein variant evolved for MHAT. (C) Kinetic scheme for dearomatization of **14a** by 9B12. Best fit parameters for the model are shown in the gray box. (D) Deconvoluted time course for the dearomatization of **14a**. Concentrations of the substrate, radical intermediate, and product are shown in black, light blue and orange, respectively. (E) Deconvoluted electronic absorption spectra for the substrate (black), intermediate (light blue) and product (orange). (F) EPR spectrum for the radical intermediate **14c** and corresponding fit.

**Figure 5:**
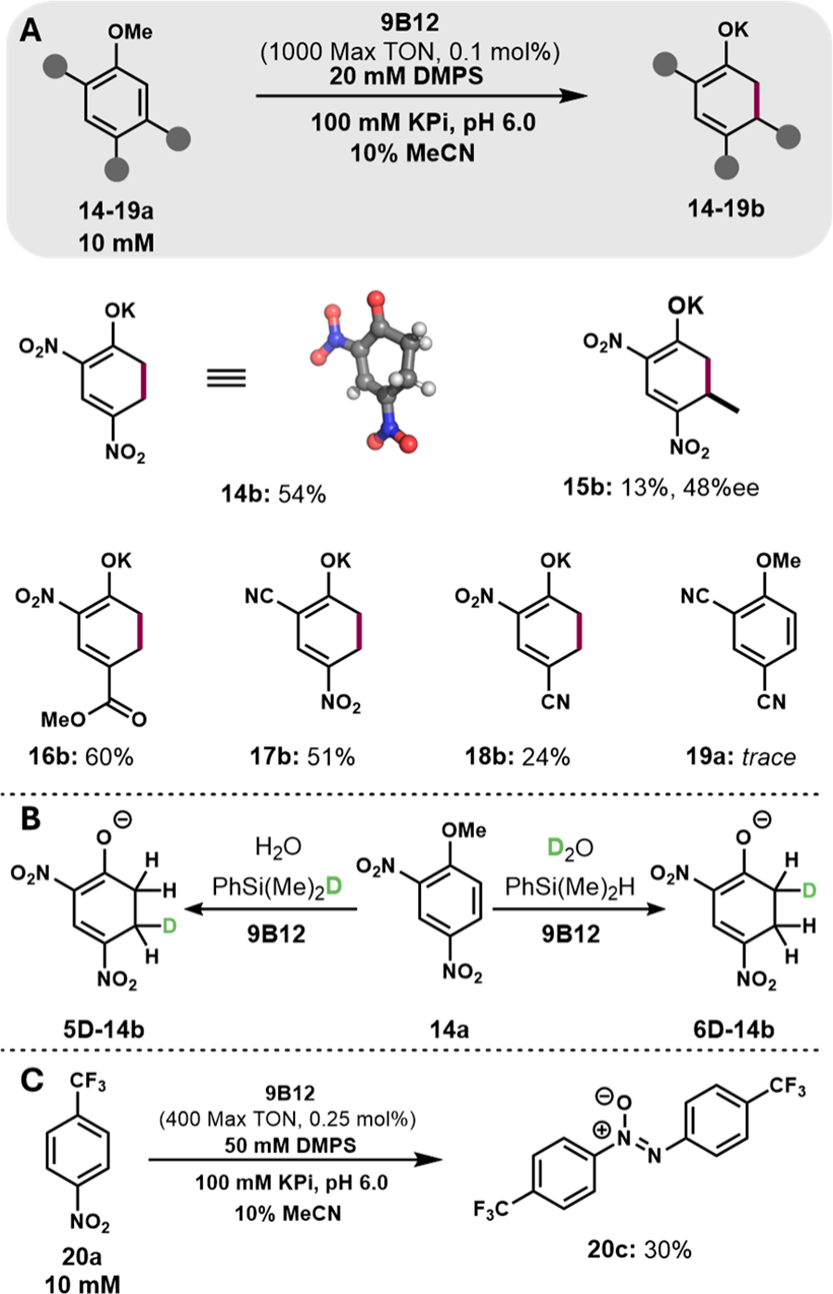
Substrate scope of reductive dearomatization. (A) Isolated yields of substituted cyclohexa-1,3-dienes. (B) Deuteration studies for dearomatization of **14a** by 9B12, using D_2_O and D_1_-DMPS. (C) Azoxybenzene formation via nitro reduction of **20a** by 9B12.

We collected full spectral time-course data at a variety of enzyme concentrations (Figures 4A, S20) and conducted a global spectral fit paired with singular value decomposition (SVD) analysis.^62^ This analysis identified a simple, two-step irreversible model fit the data (Figure 4C–D), and provided a deconvoluted electronic absorption spectrum for the intermediate with a notably broad absorption band (Figure 4E). We compared this experimental spectrum to DFT-calculated spectra of candidate anionic and radical intermediates and found that the closest spectral match came from the radical intermediate **14c**. Computed spectra for the other putative intermediates are a poor match (Figures S21–22). We freeze-quenched a reaction of 2,4-dinitroanisole **14a** with 9B12 and analyzed the sample by EPR spectroscopy. This analysis gave unambiguous evidence for a radical intermediate (Figure 4F). While multiple mechanistic pathways may operate simultaneously, the reductive dearomatization of **14a** is primarily achieved through a radical, MHAT-mediated pathway.^24^

### Scope and Limitations of MHAT Dearomatization of Aryl Methyl Ethers

We next surveyed the scope of the dearomatization by 9B12 using a selection of systematically varied aryl methyl ethers (Figure 5A). Reaction with an aryl ether bearing a methyl at the 5-carbon, the site of HAT, yielded chiral product **15b** (48% ee), consistent with enzyme-mediated HAT.

Substituting one nitro group with π-electron withdrawing groups such as the *o*-nitro, *p*-ester (**16b**) as well as cyano was well tolerated (**17b**, **18b**), but replacing both with nitriles (**19a**) led to trace reactivity. We evaluated a range of additional substitution patterns and only 2,4-anisole analogs furnished the 1,3-diene products (Figure S23). Many other substrates were consumed over the course of the reactions and only with 4-trifluoromethyl-nitrobenzene (**20a**) could we isolate a product. We observed formation of an azozybenze (**20c**), which we attribute to reduction of the nitro group and dimerization (Figure 5C). This result illustrates how, in the absence of substituent effects that direct HAT to a single site on the aromatic ring, reduction of the nitro group may also occur.

From these data, we attribute the strict dependence on substitution pattern reflects two features: electronic stabilization of intermediates and rapid hydrolysis of the product. Following initial HAT to the parent compound, **14a**, a resonance-stabilized radical intermediate undergoes reductive radical-polar crossover, forming an anionic intermediate which is resonance-stabilized by both nitro substituents (Figure S24). Spontaneous hydrolysis of the methyl ether forms an anionic species whose electron-rich character prevents subsequent reduction, cleanly yielding a cyclic 1,3-diene. For substrates without this specific 1,2,4-substitution pattern, the absence of one or more key stabilizing effects rendered the reaction unproductive.

### Emergence of Reductive Dearomatization Was Frequent during Directed Evolution

We explored whether radical dearomatization activity was accessible to other evolved MHAT variants by rescreening our collection of active variants with DMPS and **14a**, monitoring absorbance at 450 nm. Of the 80 variants tested, nearly all exhibited measurable dearomatization activity (Figures S25–27, Table S4). Dearomatization activity was modestly well correlated with deallylation activity (R^2^ = 0.58), consistent with Co–H formation as a shared rate-limiting step for many variants (Figure 4B). The most active deallylation variant, 9B12, was also the most active variant for dearomatization. We considered whether these evolved proteins might also be activating for reaction with the native heme cofactor. We expressed and purified Fe9B12 and tested the enzyme for dearomatization activity. As with deallylation (above), we found this enzyme can catalyze dearomatization of **14a** with DMPS, but that the Co-loaded enzyme had roughly 3-fold higher activity (Figure S27). This discovery demonstrates that MHAT is engineerable for native heme-loaded P450s, and is consistent with recently reported hydride transfer and MHAT reactivity for heme containing proteins.^34,35^ Future investigations may explore the differences in reactivity between Co and Fe dependent MHAT biocatalysts.

## Conclusions

Here we report that a P450 enzyme scaffold utilizes the new-to-nature cofactor CoPPIX to form a metal-hydride intermediate, enabling metal–hydrogen atom transfer (MHAT) into both alkenes and electron deficient arenes. The requisite CoPPIX cofactor is fully biosynthesized by *E. coli*, and thus the system is simple to engineer. As the Co–H intermediate described here was not previously known in enzymology, the principal goal of our study was to engineer a suite of biocatalysts that can use a water-stable silane to form a Co-hydride intermediate that is capable of engaging in MHAT.

To this end, we developed a nitrophenolate-based colorimetric assay as a continuous readout of MHAT-mediated deallylation activity. PS, a substrate commonly used for MHAT in synthetic contexts, was prone to hydrolysis and may prove problematic in some biocatalytic implementations. Assays with DMPS, a more water-stable silane, revealed that Co–H formation was rate-limiting for the reaction. We utilized a colorimetric probe to screen thousands of enzyme variants over two rounds of directed evolution, resulting in a panel of 80 enzyme variants with diverse protein sequences—all of which can react readily with DMPS to form a Co–H intermediate.

While future studies will assay these variants for new reactions, here, we focused on the serendipitous discovery of MHAT-mediated reductive dearomatization of nitroaryl ethers. The scope of the reaction was limited by the electronics of the substrate, but the reactivity demonstrated has, to our knowledge, not been observed previously in biocatalytic or synthetic settings. This reaction is, thus, the first wholly new transformation discovered with an artificial metalloenzyme. The reactions developed here are relatively mild, do not require expensive organometallic reagents, and may be subject to further genetic optimization. Therefore, these results represent a valuable addition to the biocatalytic toolbox and open many avenues for future inquiry into the fundamental properties of metal-hydrides and their synthetic applications.

## Supplementary Material



## Notes

Experimental procedures, characterization data, NMR spectra, and SFC/HPLC traces (PDF)

## #

Department of Chemistry, University of Manchester, Manchester M13 9PL, United Kingdom; Manchester Institute of Biotechnology, University of Manchester, Manchester M1 7DN, United Kingdom

## ‡

New England Biolabs; Ipswich, MA, 01938

## Abbreviations

CoPPIX: cobalt protoporphyrin IX
MHAT: metal–hydrogen atom transfer
Co–H: cobalt hydride
CoCYP119: cobalt-bound CYP119
PS: phenylsilane
DMPS: dimethylphenylsilane
electron paramagnetic resonance: EPR
UV–vis: ultraviolet–visible
DFT: density functional theory
TD-DFT: time-dependent DFT
iPrOPS: RuebenSilane
WT: wild-type
TTN: total turnover number
