# Supplementary material for: Enzymatic Metal–Hydrogen Atom Transfer with a Cobalt Protoporphyrin Cofactor
Source: J Am Chem Soc. 2026 Jan 30;148(5):5443–52. doi: 10.1021/jacs.5c19000 (PMC12903868; doi:10.1021/jacs.5c19000)
Supplement: Supplementary file 1 [file ja5c19000_si_001.pdf]

Supporting Information
for

Enzymatic metal-hydrogen atom transfer with a cobalt protoporphyrin cofactor

Authors: Carly L. Masonheimer[1]†, Michael J. Rourke[1]†, Reece S. Gardner[1], Ryan L. Hall[1], Lydia J. Perkins[1]‡*, Thomas C. Brunold[1]*, Andrew R. Buller[1]*

Affiliations:

[1]Department of Chemistry, University of Wisconsin–Madison; Madison, WI, 53706, USA.

†These authors contributed equally to this work

‡ Present address: New England Biolabs; Ipswich, MA, 01938

*To whom correspondence should be addressed: Lydia J. Perkins (PerkinsLydiaJ@gmail.com), Thomas C. Brunold (brunold@chem.wisc.edu), Andrew R. Buller (andrew.buller@manchester.ac.uk)

SI Table of Contents

	PAGE
**List of Abbreviations Used in Supplemental Information**	7
**Supplemental Figures Referenced in the Main Text**	
**Figure S1.** Quantification of CoCYP119 metal loading via ICP-MS and spectral deconvolution.	8
**Figure S2.** Comparison of spectral changes upon addition of sodium dithionite, PS and DMPS to CoCYP119	9
**Figure S3.** Comparison of spectral changes upon addition of sodium dithionite and PS to CoPPIX	10
**Figure S4.** Formation of H_2 gas by CoCYP119 incubated with PS and DMPS	11
**Figure S5.** DFT optimized structures of CoPPIX-hydride intermediate	12
**Figure S6.** TD-DFT calculated spectra for putative cobalt hydride and comparison to experimental spectra.	13
**Figure S7.** TD-DFT calculated spectra for combined putative cobalt hydride and hydroxide thiolate and comparison to experimental spectra	13
**Figure S8.** Detection of isobutene in the headspace of a reaction with **1a** and CoCYP119.	14
**Figure S9.** Reaction of substrate **1a** and DMPS with various catalyst scaffolds.	15
**Figure S10.** Structural model of residues selected for site saturation mutagenesis (PDB 1IO7).	16
**Figure S11.** Retention of function curves for site saturation mutagenesis (SSM) library at residues on the proximal face of the CoPPIX cofactor, including the axial ligand, screened with substrate **1a** and PS.	17
**Figure S12.** Retention of function curves for site saturation mutagenesis (SSM) library at residues on the distal face of the CoPPIX cofactor, screened with substrate **1a** and PS.	18
**Figure S13.** Images of cobalt-substituted master plate screening with deallylation substrates **1a** and **4a**.	19
**Figure S14.** Image of heme-containing master plate variants screened with deallylation substrate **1a**.	20
**Figure S15.** Retention of function curve for master plate of active recombination variants for deallylation of the substrate **4a** and DMPS.	21
**Figure S16.** Turnover numbers for variants along the evolutionary lineage of 9B12, using substrate **4a** and DMPS.	22
**Figure S17.** Hypothetical mechanisms for fragmentation of allyl group in M-HAT deallylation reaction.	23
**Figure S18.** Time course spectra of reaction of **1a** and **4a** with CoCYP119 9B12.	24

**Figure S19.** Comparison of LC-MS traces of reaction mixtures: 9B12 with **1a** and **14a** versus a dinitrophenol (**1b**) standard.	25
**Figure S20.** Time course spectra for dearomatization of 2,4-dinitroanisole **14a** with 9B12 and DMPS.	26
**Figure S21.** DFT optimized structures of **14a**, three viable reaction intermediates, and two possible products.	27
**Figure S22.** TD-DFT calculated spectra of putative radical and anionic reaction intermediates, and comparison to (experimental) deconvoluted intermediate spectra.	28
**Figure S23.** Scope of nitroarenes screened in the reductive dearomatization reaction for which we observed high conversion but were not able to isolate products.	29
**Figure S24.** Stabilizing interactions favoring product outcomes for M-HAT dearomatization of nitroarenes and *N*-heterocycles.	30
**Figure S25.** Image of cobalt-substituted master plate screening with 2,4-dinitroanisole **14a.**	31
**Figure S26.** Retention of function curve for master plate of active recombination variants for dearomatization of 2,4-dinitroanisole **14a** and DMPS.	32
**Figure S27.** Dearomatization activity across the evolutionary lineage of CoCYP119 9B12, measured with **14a** and DMPS substrates.	33a
**Supplemental Tables**	
**Table S1.** Primers for generation of site-saturation and recombination libraries.	34
**Table S2.** Gene sequences used.	35
**Table S3.** Active site recombination library amino acid residues possible at each site.	37
**Table S4.** Sequence identity and measured fold activities of activated variants in the recombination master plate.	38
**Experimental Methods**	
General experimental methods	41
Equipment and instrumentation	41
**Cloning, expression, and protein preparations**	
Cloning and expression of CoCYP119 and variants	43
Purification of CoCYP119 and variants	44

Preparation of 9B12 heat-treated lysate	45
**Figure S28.** SDS-PAGE analysis of CoCYP119 9B12.	45
**Table S5.** Protein yields obtained for CoCYP119 WT and 9B12	45
Modified pyridine hemochrome assay for cofactor-loaded protein quantification	46
ICP-MS analysis of CoCYP119 samples	46
Spectroscopic analysis of CoCYP119 metallocofactor content: spectral deconvolution	47
**Figure S29.** Spectral deconvolution to quantify cofactor metal loading.	48
**Mechanistic interrogations of initial enzymatic M-HAT**	
Measurement of initial rates and progress curves using UV-vis: cuvette and plate reader	49
**Figures S30-32.** UV-vis standard curves for products **1b** and **4b.**	49
**Figure 33.** Plate reader standard curve for product **4b**.	51
**Figures S34 – S41.** Initial velocity traces for **2a** and **4a**.	51
**Directed evolution and screening**	
SSM library construction, expression, and screening	58
Recombination library construction, expression, and screening	59
Analytical scale reactions – total turnover determination: LC-MS and UV-vis - plate reader	61
Construction of standard curves for deallylation scope and TTN experiments	62
**Figures S42-49.** LC-MS standard curves for product quantification.	62
**Kinetic modeling**	
Kinetic analysis of dearomatization time course using Kintek Explorer	66

**Figure S50.** Singular value decomposition (SVD) component spectra and time dependencies	68
**Figures S51-54.** Singular Value Decomposition (SVD) and kinetic fit of UV-visible spectra according to various kinetic models for dearomatization of **14a.**	71
**Reaction optimizations**	
Dearomatization Reaction Optimization - Anaerobic vs Aerobic Reaction Conditions	75
**Table S6.** Effect of oxygen on the dearomatization reaction.	76
Dearomatization Reaction Optimization - Thiol Additive Screen	76
**Table S7.** List of thiols screened and effect on the dearomatization reaction.	77
Dearomatization Reaction Optimization - Oxidant Additive Screen	77
**Table S8.** List of oxidants screened and effect on the dearomatization reaction.	78
**Mechanistic analysis of dearomatization**	
Deuterium Incorporation Experiments: D1-DMPS and D_2O with **14a**	78
**Figure S55.** Deuteration patterns of **14b** when D1-DMPS or D_2O are used.	81
Tempo Trapping Experiment	82
**Figure S56.** Low-resolution LC-MS analysis of the **14c**-TEMPO adduct.	83
**Preparative scale enzymatic reactions**	
General Procedure for dearomatization of nitroanisole derivatives	83
Reductive Dearomatization Reaction Scope	84
**Figure S57.** UV absorbance at 350nm from chiral HPLC analysis of **15b.**	86
Azoxybenzene synthesis: scale up of 1-nitro-4-(trifluoromethyl)benzene **S5**	89
Unproductive enzymatic M-HAT to nitroarenes	90

**Figure S58.** Scope of nitroarenes screened in the reductive dearomatization reaction for which we observed high conversion, but were not able to isolate products.	91
**Figures S59-67.** UPLC traces showing conversion of nitroarene substrates for which we were not able to isolate products	92
**Figure S68.** Substrates exhibiting limited or no reactivity in the enzyme-mediated dearomatization of nitroarenes reaction.	101
**UV-visible and EPR spectroscopy of reaction intermediates**	
Electronic absorption spectra measurements of CoCYP119 and PS adducts	102
General procedure for EPR characterization of dearomatization radical intermediate	102
**Computational characterization of enzyme and other reaction intermediates**	
Geometry optimizations	103
TD-DFT computed absorption spectra	104
Comparison of calculated iron and cobalt-bound hydride species	106
**Figure S69.** Potential energy curves (PECs) for the of the low spin, metal-hydride thiol(ate) species.	106
**Synthesis of substrates**	
General procedure for preparation of protected dinitro substrates **1-3a**	108
Characterization of **1-3a**	108
Preparation of deuterated dimethyl(phenyl)silane and characterization	110
General procedure for methallylation of phenols	111
Characterization of methallylated phenols	112
General procedure for allylation of phenols and anisole synthesis	116
Characterization of allylated phenols	116
NMR spectra of novel compounds	120

**Small molecule X-ray crystallography**	
Data collection and refinement	144
**Figure S70.** A molecular drawing of **14b**	146
**Table S9.** Crystal data and structure refinement for **14b**	147
**References**	148

List of abbreviations

TB: Terrific Broth
LB: Luria-Burtani
AMP: ampicillin
IPTG: isopropyl β-D-1-thiogalactopyranoside
5ALA: 5-aminolevulinic acid
PPIX: Protoporphyrin IX (dianion)
CYP119: *Sulfolobus acidocaldarius* cytochrome P450 119
UV-Vis: ultraviolet-visible light spectroscopy (electronic absorption spectroscopy)
UPLC: ultra pressure liquid chromatography
ICP: inductively coupled plasma
MS: mass spectrometry
OD_{600}: optical density at 600nm
NMR: nuclear magnetic resonance spectroscopy
TTN: total turnover number
PS: phenylsilane ($PhSiH_3$)
DMPS: dimethylphenylsilane ($PhSi(CH_3)_2H$)
DMSO: dimethyl sulfoxide
MeCN: acetonitrile
KPi: potassium phosphate
DFT: Density functional theory
HPLC: High pressure liquid chromatography
Apparent yield: (product peak intensity)/(Substrate + Product peak intensities)
This formula expresses the apparent yield as the ratio of the product peak intensity to the sum of both the substrate and product peak intensities.

Supplemental Figures

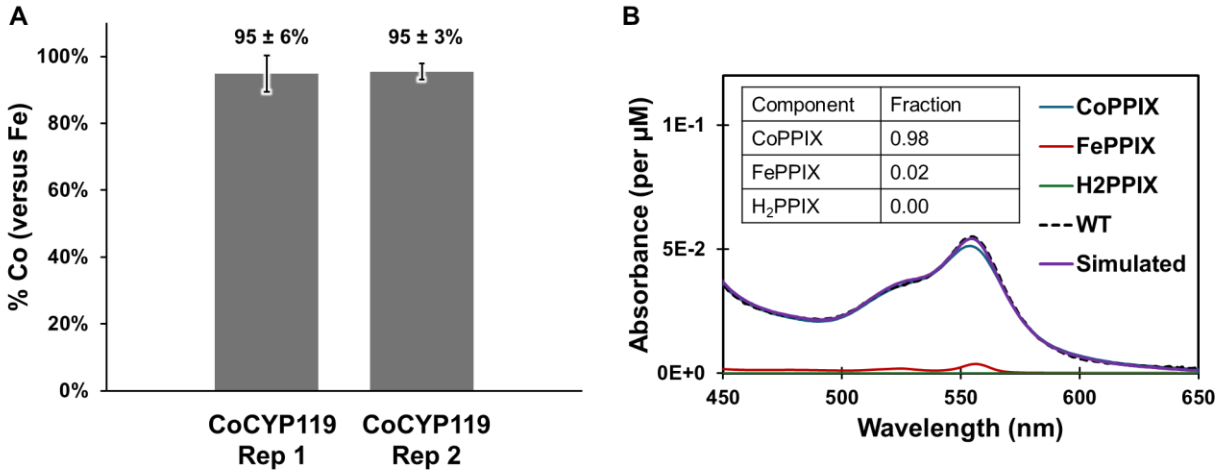

Figure S1. Quantification of CoCYP119 metal loading via ICP-MS and spectral deconvolution. (A) Duplicate samples of CoCYP119 WT were digested in nitric acid prior to analysis. Data are the average of triplicate ICP-MS measurements. Additional experimental details can be found in the supplemental section titled "ICP-MS analysis of CoCYP119 samples". (B) Spectral deconvolution reports a metal loading consistent with ICP-MS. A linear combination of standard spectra for bis-pyridine complexes of CoPPIX (blue trace), FePPIX (red trace) and unmetalated PPIX (black trace) generate a simulated spectrum (purple trace) that matches the pyridine-extracted cofactor spectrum of isolated WT CoCYP119 (black dashed trace). Cofactor loadings are calculated based sum of least squares fit of the simulated spectrum to the experimental and known extinction coefficients for each pyridine-metalloporphyrin complex. Additional experimental details can be found in the supplemental section titled "Spectroscopic analysis of CoCYP119 metallcofactor content".

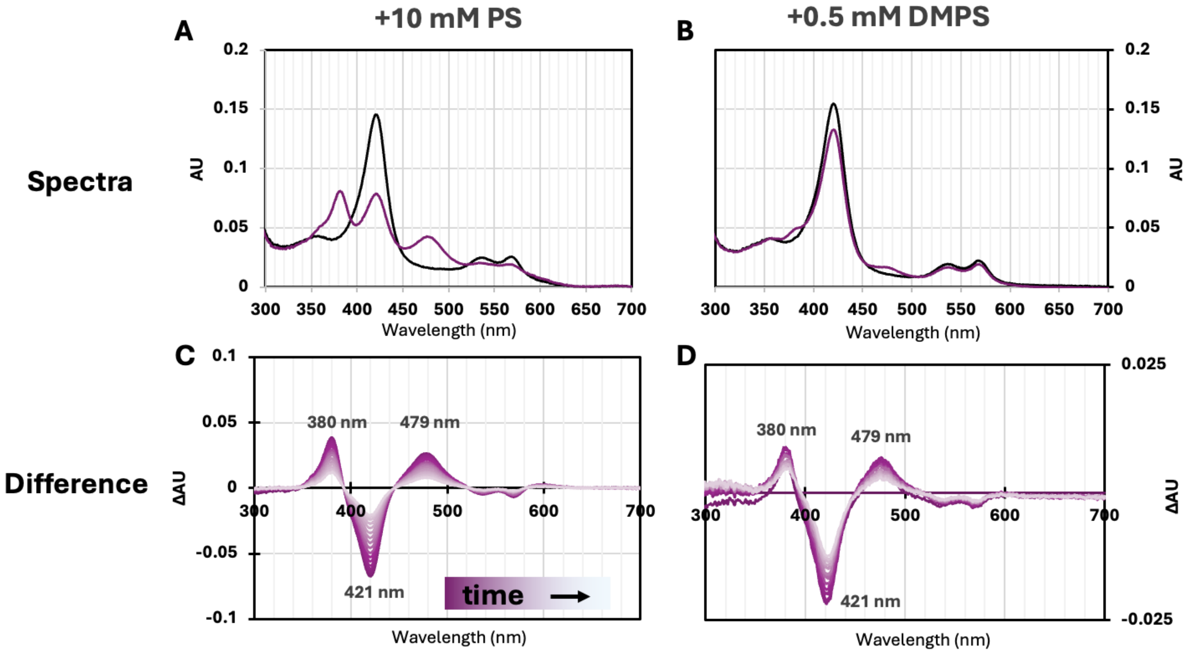

Figure S2. Comparison of spectral changes upon addition of sodium dithionite, phenylsilane (PS) and dimethylphenylsilane (DMPS) to CoCYP119. A-B) Black traces represent as-isolated spectra of CoCYP119 (20µM) in potassium phosphate buffer pH 6.0. The purple trace shows the spectra immediately following the addition of either 10 mM phenylsilane (A) or 0.5 mM DMPS (B). C-D) Difference spectra taken every 1 min following the addition of 10 mM PS (C) or 0.5 mM DMPS (D). The darkest purple color represents the earliest time point, while the lighter color represents the last time points (roughly 10 min).

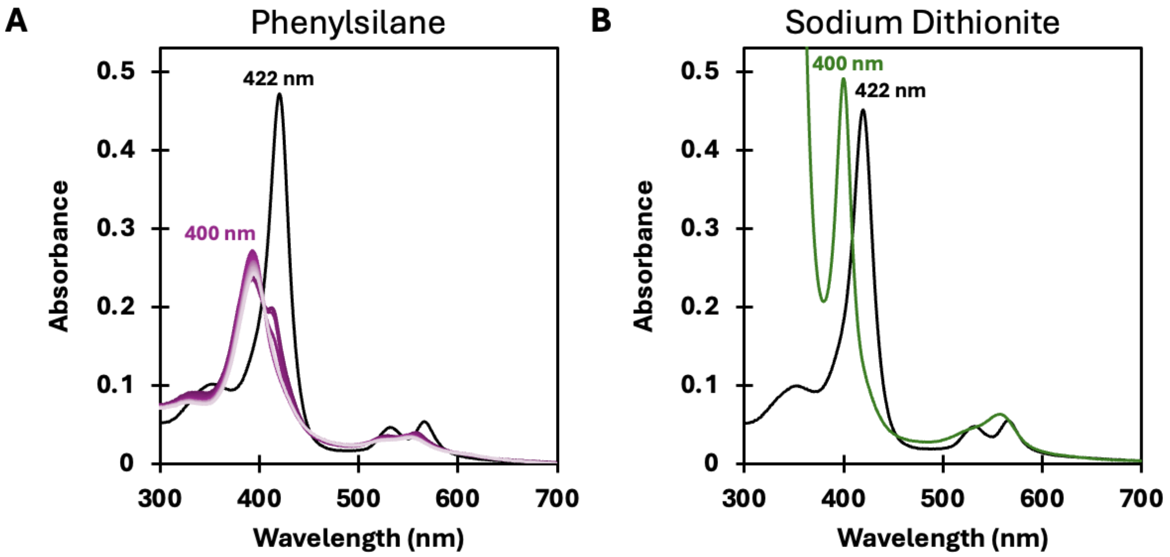

Figure S3. Comparison of spectral changes upon addition of sodium dithionite and phenylsilane (PS) to CoPPIX. Black traces represent the electronic absorption spectrum of CoPPIX (20µM) in potassium phosphate buffer pH 6.0 (~2% DMSO). The colored traces show the spectrum following the addition of either 10 mM phenylsilane (A, purple) or a few crystals of sodium dithionite (B, green)

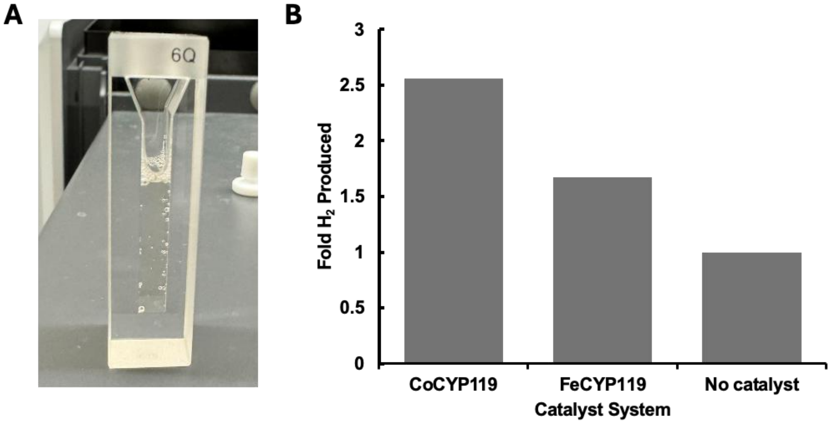

Figure S4. Formation of H_2 gas by CoCYP119 incubated with phenylsilane (PS) and dimethylphenylsilane (DMPS). A) Bubbles which form following addition of phenylsilane (5 mM) to CoCYP119 (2 μM) in 100 mM potassium phosphate buffer, pH 6.0. B) Quantification of H_2 formation by GC. A 2 mL glass crimp vial was charged with 0.8 mL of a 5.0 μM solution of WT CoCYP119, WT FeCYP119, or buffer only in KPi buffer pH = 6.0. The vial was sealed with a vial crimper. A 5 mM solution of dimethylphenylsilane (0.2 mL) was added to each sealed vial with a glass Hamilton syringe. The vials were gently flicked to mix, and incubated at 50°C overnight. Following overnight incubation, 100 μL of the headspace of each vial was sampled with a Hamilton syringe and injected onto the GC. The retention time of peaks were compared to a known standards to identify the increased presence of H_2 gas in experimental vials relative to atmosphere. The fold-increase H_2 production compared to the no enzyme control (i.e. production above background hydrolysis) was calculated for each catalyst, and is plotted in (B). CoCYP119 was found to increase the concentration of H_2 in solution by roughly 2.5-fold relative to background hydrolysis.

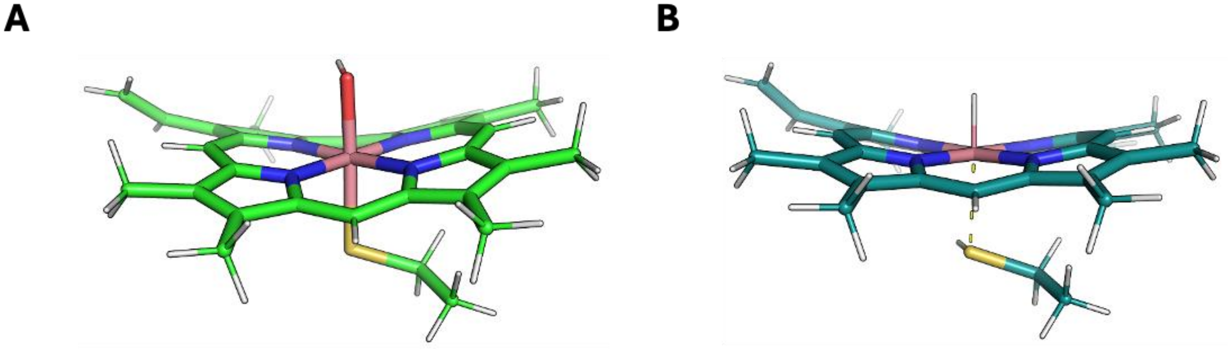

Figure S5. DFT optimized structures of CoPPIX-hydride intermediate. **A**. DFT optimized model of the hydroxide thiolate resting state of CoCYP119. **B.** DFT optimized model of the hydride thiol intermediate of CoCYP119.

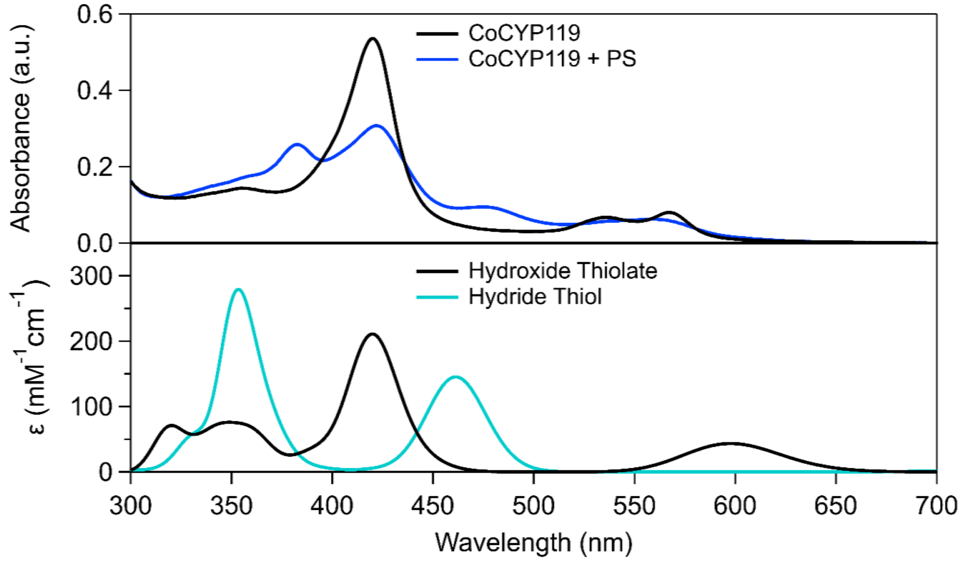

Figure S6. Top: Experimental absorption spectra of 20 µM CoCYP119 before (black) and immediately after (blue) the addition of 10 mM phenylsilane. Bottom: TD-DFT computed absorption spectra for the hydroxide thiolate resting state (black) and the hydride thiol intermediate species (teal). The computed spectra were uniformly red shifted by -5440 cm^{-1} to align the Soret band in the computed spectrum of the hydroxide thiolate resting state to the Soret band at 421 nm in the experimental spectrum of the CoCYP119.

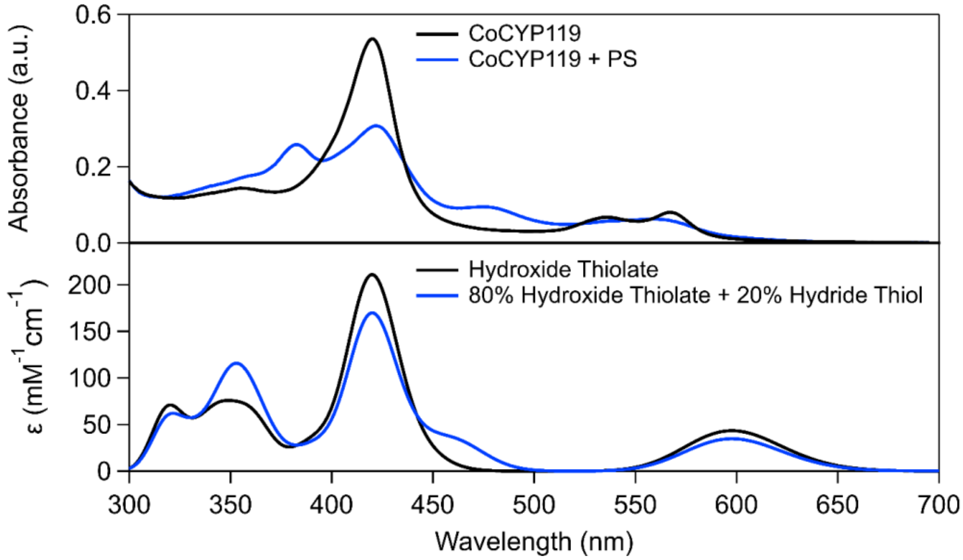

Figure S7. Top: Experimental absorption spectra of 20 µM CoCYP119 before (black) and immediately after (blue) the addition of 10 mM phenylsilane. Bottom: TD-DFT computed absorption spectra for the hydroxide thiolate resting state (black) and combined 80% hydroxide thiolate resting state with 20% hydride thiol intermediate species (blue). The computed spectra were uniformly red shifted by -5440 cm^{-1} to align the Soret band in the computed spectrum of the hydroxide thiolate resting state to the Soret band at 421 nm in the experimental spectrum of the CoCYP119.

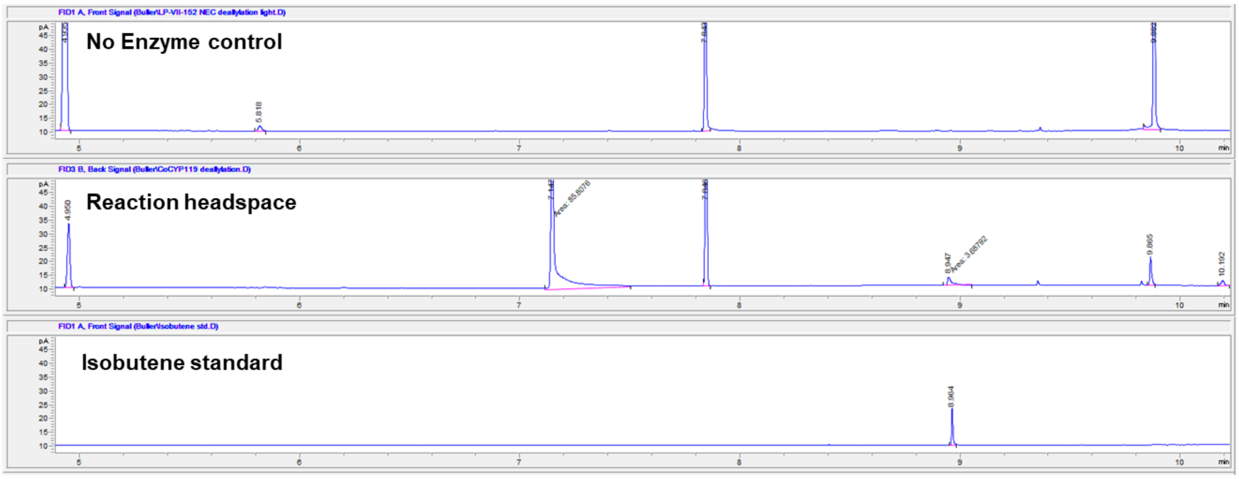

Figure S8. Detection of isobutene in the headspace of a reaction with **1a** and CoCYP119. Reaction conditions: 2 mM DMPS, 1 mM substrate **1a**, 10 µM WT CoCYP119 in KPi pH 6.0 with 20% DMSO. Reactions were run overnight at room temperature in a sealed 2 mL crimp vial. 500 µL of ethyl acetate was added to the sealed vial to quench, and the organic supernatant was analyzed by GC.

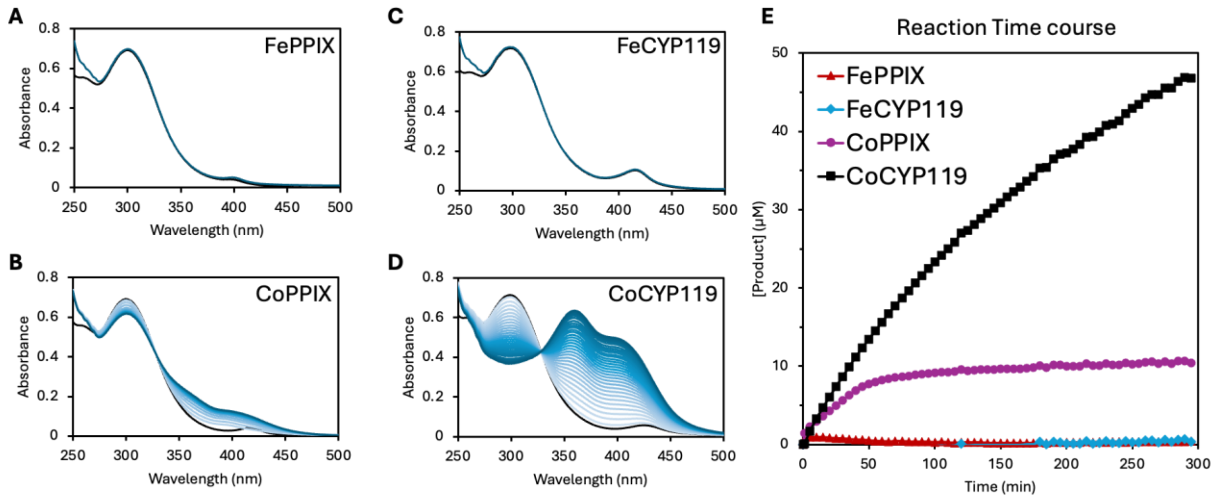

Figure S9. Reaction of 2-Me-Allyl-2,4-dinitrophenol ether **1a** (0.05 mM) and Dimethylphenylsilane (DMPS) (0.1 mM) with various catalyst scaffolds (5 µM each). A quartz microcuvette (Starna) was charged with 380 µL of 100 mM potassium phosphate buffer (pH 6.0), and the spectrometer was blanked. Next, 20 µL of a 125 µM solution of each enzyme or cofactor (5 µM final concentration) was added. The Soret absorbance of each catalyst can be observed near 400 nm. A DMSO solution containing **1a** (50 µL of 0.5 mM solution, final concentration of 100 µM) was added, and a zero time point spectrum was taken (black traces). Finally, 50 µL of a 0.1 mM DMSO solution of DMPS was added to each reaction, and a spectrum of each reaction was taken every 3 minutes for a total of 5 hours. **A-D**) Individual spectra for each catalyst system, with black traces representing the zero time point and colored traces representing subsequent time points, from light to dark over time. The concentration of product over time is plotted in **E**, as calculated based on the change in absorbance at 400 nm and an extinction coefficient of 9700 M^{-1}.

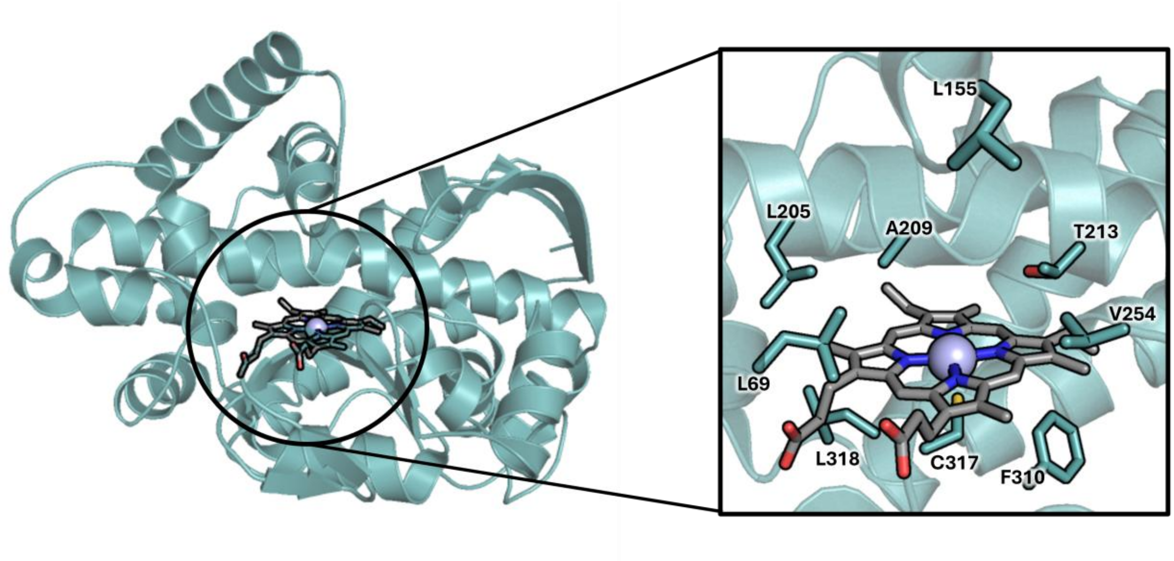

Figure S10. Structural model of residues selected for site saturation mutagenesis (PDB 1IO7).[1]

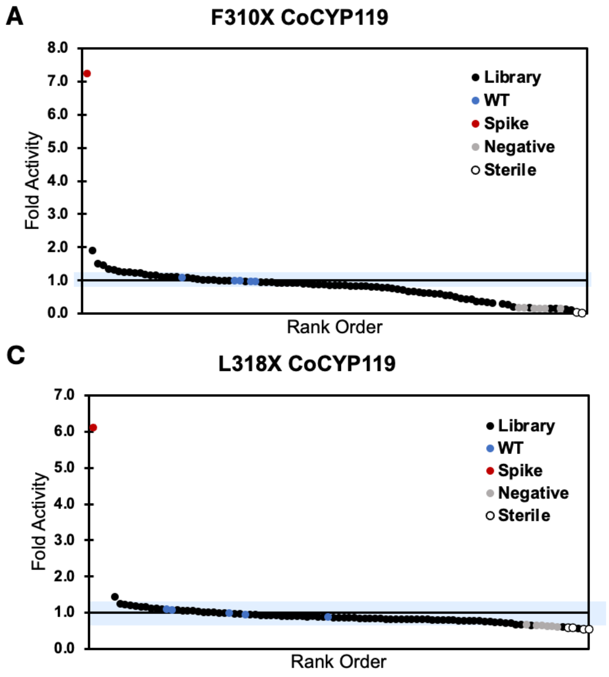

Figure S11. Retention of function curves for site saturation mutagenesis (SSM) library at residues on the proximal face of the CoPPIX cofactor, including the axial ligand, screened with substrate **1a** and PS. The light blue box indicates the range of activity measured for the parent enzyme (WT). Additional experimental details can be found in the supplemental section titled "SSM library construction, expression, and screening".

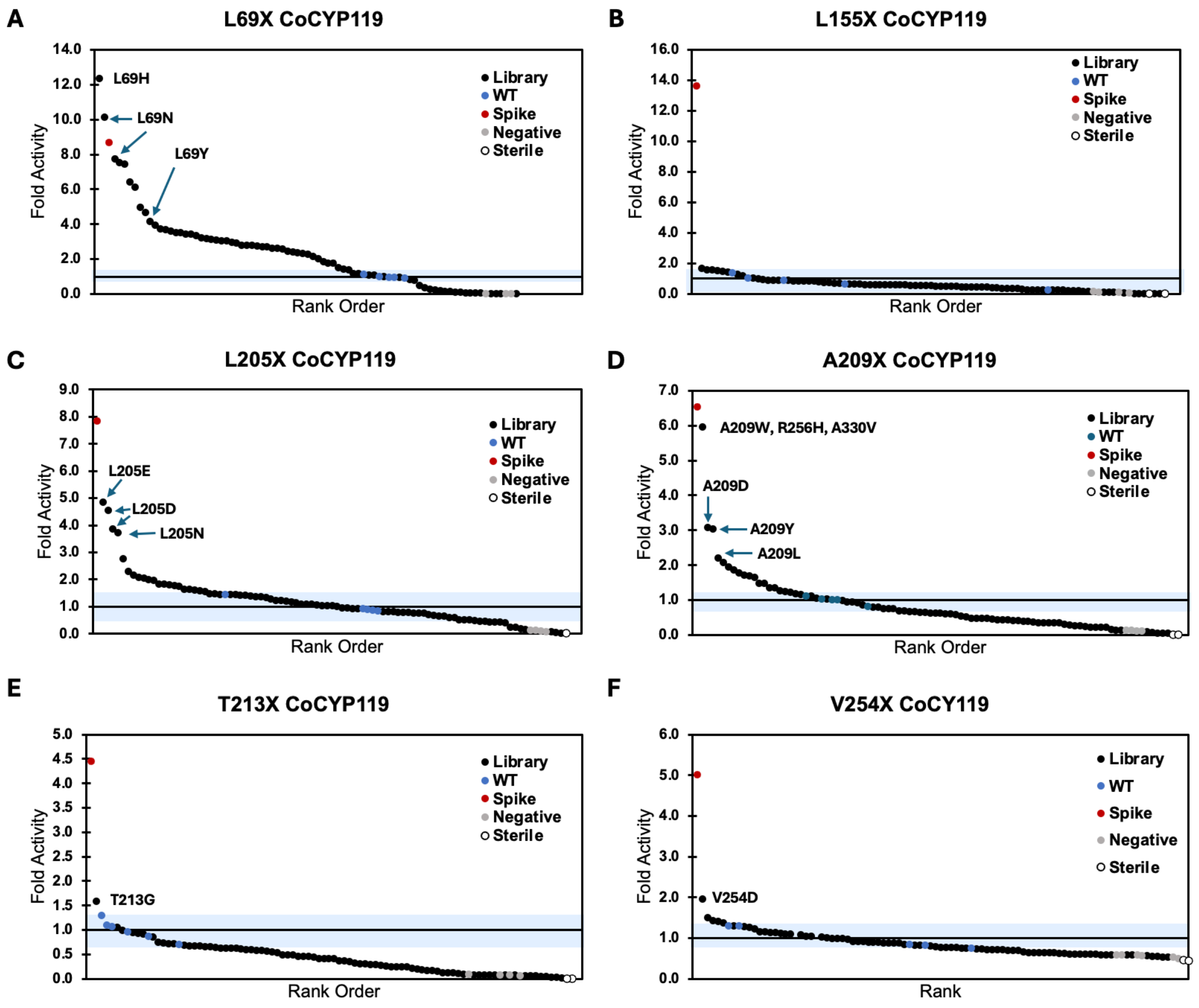

Figure S12. Retention of function curves for site saturation mutagenesis (SSM) library at residues on the distal face of the CoPPIX cofactor, screened with substrate **1a** and PS. The light blue box indicates the range of activity measured for the parent enzyme (WT). Additional experimental details can be found in the supplemental section titled "SSM library construction, expression, and screening".

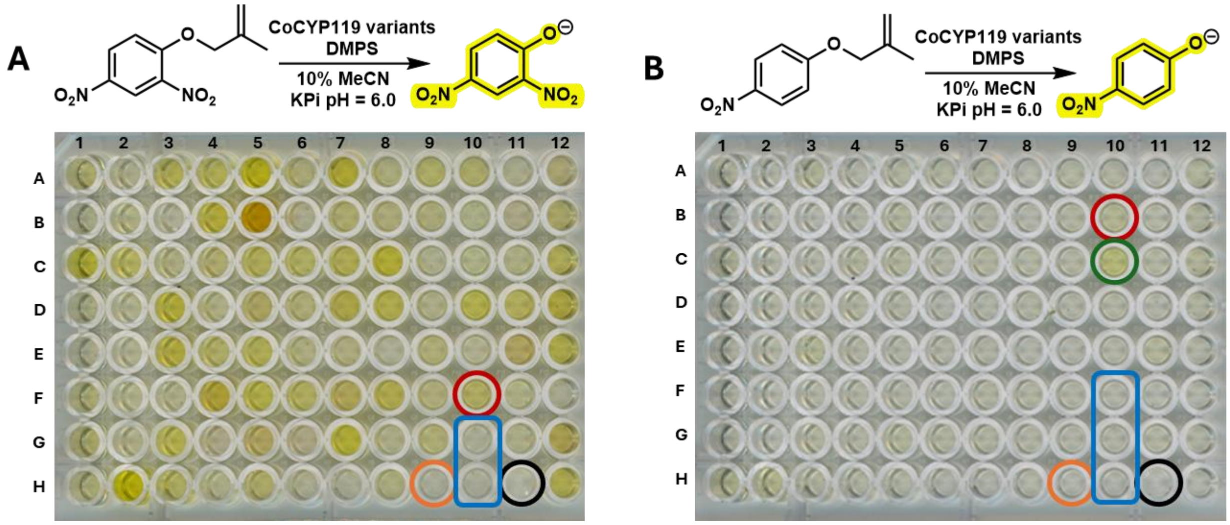

Figure S13. Images of cobalt-substituted master plate screening with (A) dinitro **1a** and (B) mono nitro **4a** deallylation substrates. The yellow color in some wells indicates the presence of nitrophenolate, for which absorbance is quantified at 405 nm using a plate reader. The presence of orange-ish color, such as in well B5 in (A), indicated the presence of a new dearomatization reaction which involved HAT to the arene. Screening with the 4-nitrophenolate (B) clarified analysis of the dearomatization reaction. Control wells have been denoted by colored circles: orange outline indicates negative wells where a non-heme enzyme is expressed, black outline indicates sterile wells, blue outline indicates parent wells with CoCYP119 V254D, red outline indicates wells spiked with purified CoCYP119 WT and green outline indicates a well spiked with purified CoCYP119 V254D. Additional experimental details can be found in the supplemental section titled "Recombination library construction, expression, and screening".

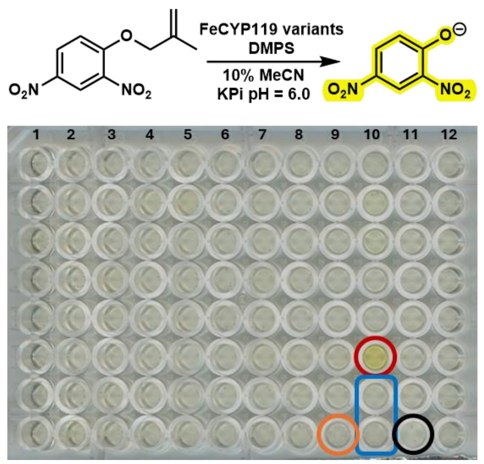

Figure S14. Image of heme-containing master plate variants screened with dinitro deallylation substrate **1a**. The yellow color of some wells indicates the presence of nitrophenolate, for which absorbance is quantified at 405 nm using a plate reader. Control wells have been denoted by colored circles: orange outline indicates a negative well where a non-heme enzyme is expressed, black outline indicates a sterile well, blue outline indicates parent wells with CoCYP119 V254D and red outline indicates a well spiked with purified CoCYP119 WT. Additional experimental details can be found in the supplemental section titled "Recombination library construction, expression, and screening".

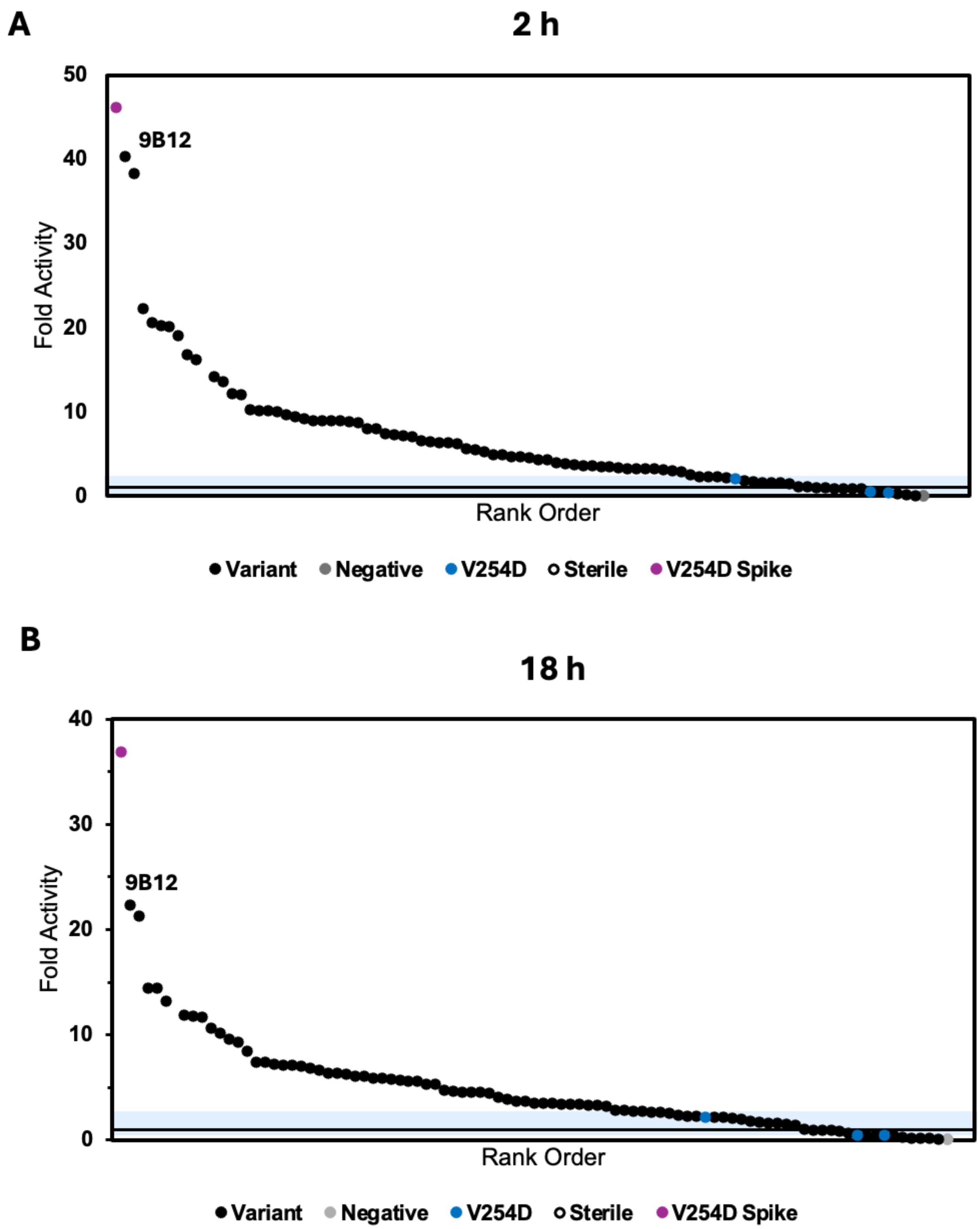

Figure S15. Retention of function curve for master plate of active recombination variants for deallylation of the mononitrated substrate **4a** and DMPS at (A) 2 h and (B) overnight (~18 h). The light blue box indicates the range of activity measured for the parent enzyme (V254D). Additional experimental details can be found in the supplemental section titled "Recombination library construction, expression, and screening".

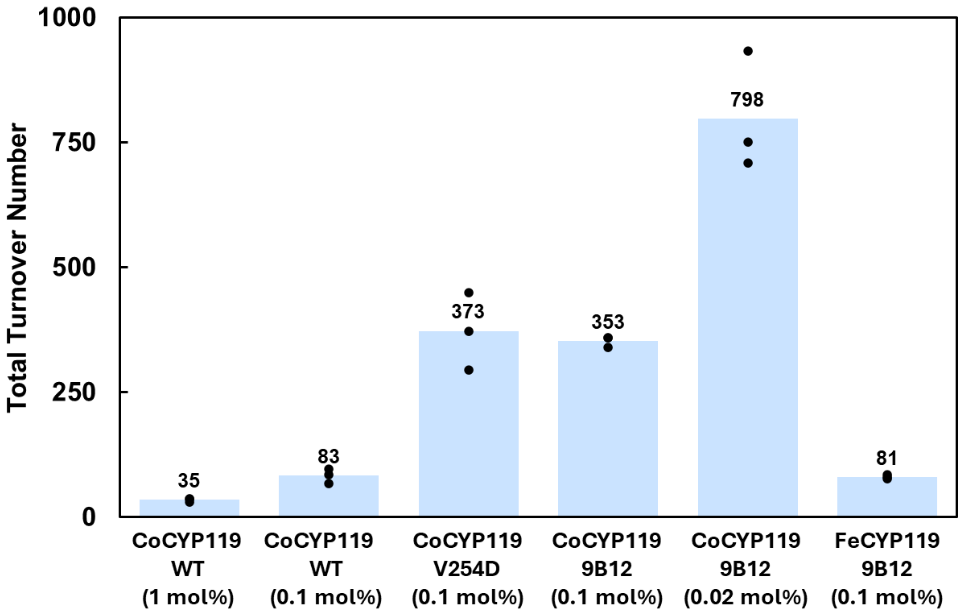

Figure S16. Turnover numbers for variants along the evolutionary lineage of 9B12, using substrate **4a** and DMPS. Dots represent triplicate measurement data points, and bars represent the average of these data points. Average turnover numbers are reported above each bar. These measurements were made using LC-MS analysis. Note that the Fe-loaded enzyme was assayed under different conditions than the Co-loaded enzyme due to low activity. Additional experimental details (including those differences) are described in the supplemental section titled "Analytical scale reactions – yield and total turnover determination".

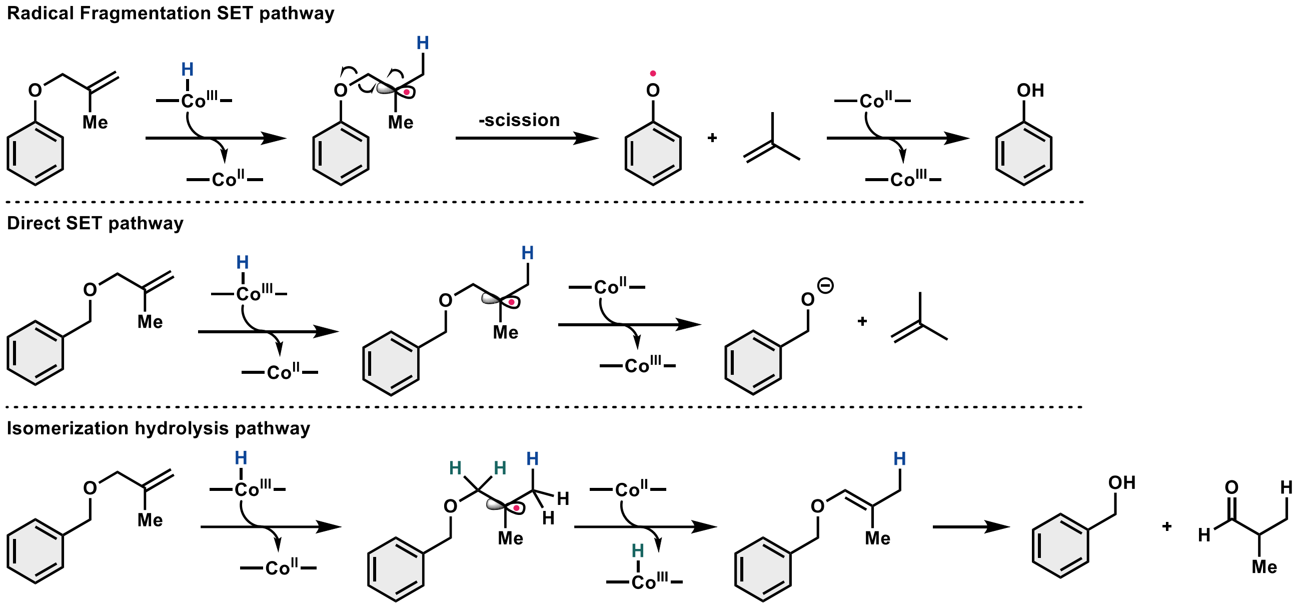

Figure S17. Hypothetical mechanisms for fragmentation of allyl group in M-HAT deallylation reaction.

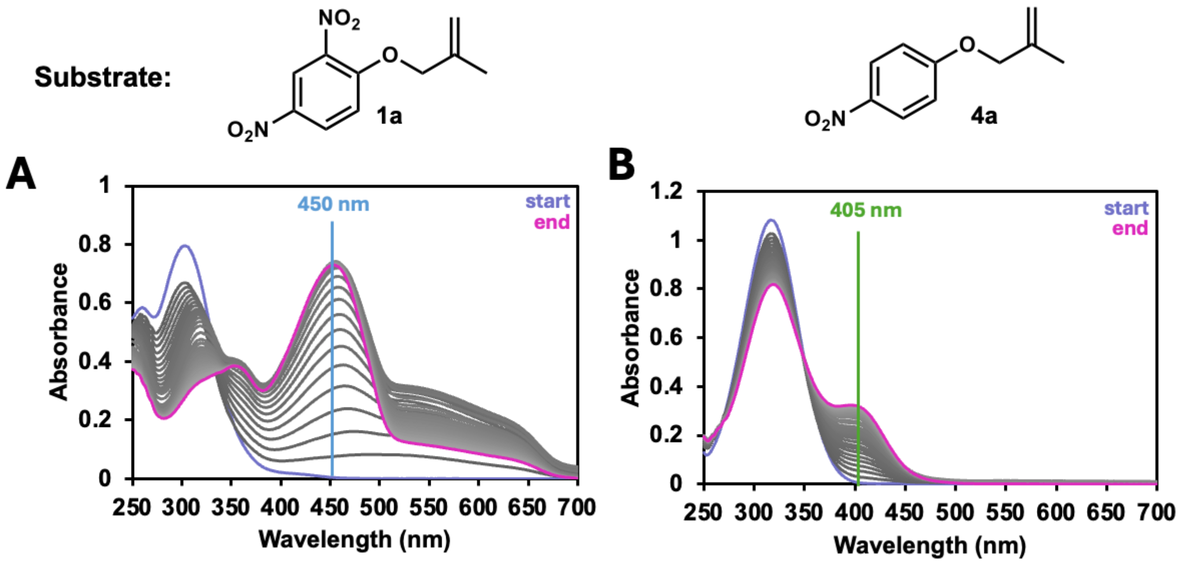

Figure S18. Time course spectra of reaction of **1a** (A) and **4a** (B) with CoCYP119 9B12. DMPS (0.2 mM) was added to a solution of either **1a** or **4a** and 9B12 (0.1 µM) in 200 mM KPi pH 6.0 with 10% acetonitrile. Spectra were recorded every three minutes for ~1 h. The purple trace represents the spectrum of the reaction solution prior to addition of DMPS, and the pink trace represents the final spectrum taken. Grey traces represent time points taken between the start and end.

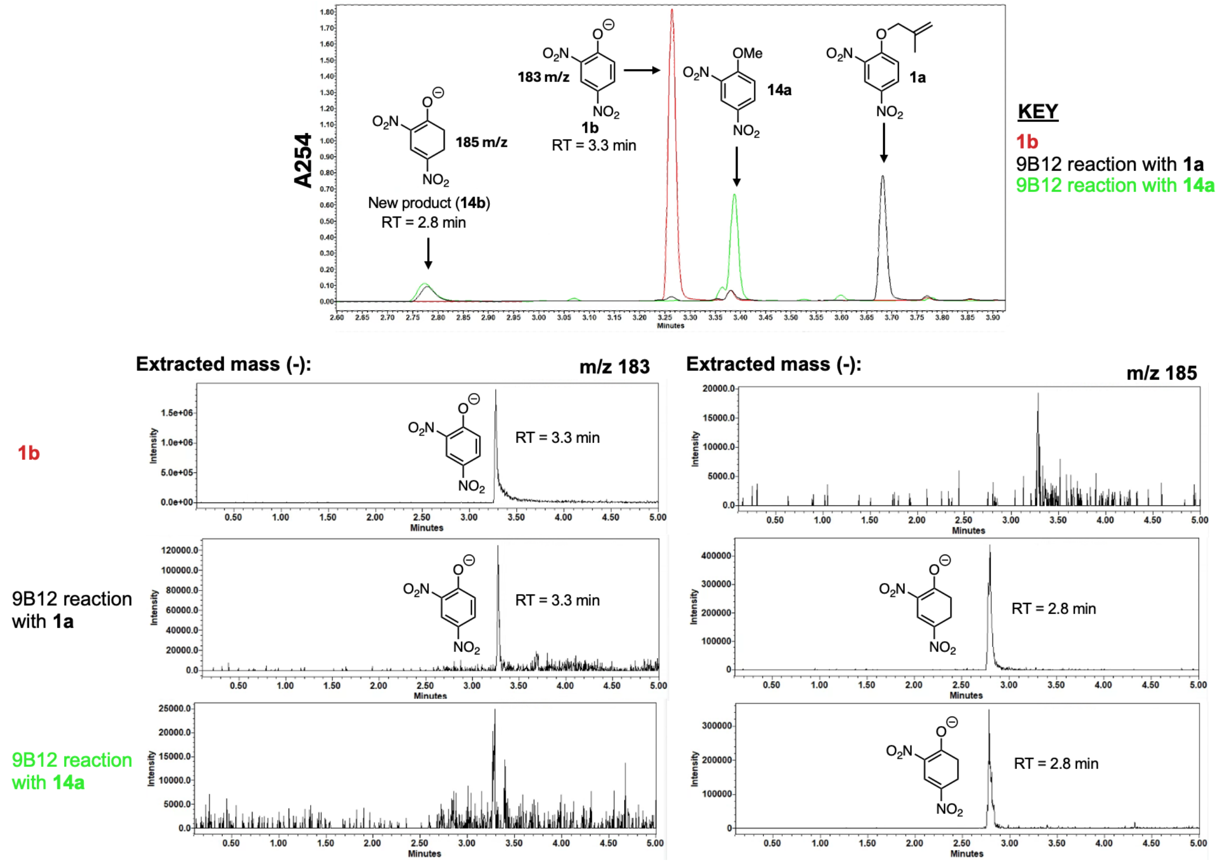

Figure S19. Comparison of LC-MS traces of reaction mixtures: 9B12 with **1a** and **14a** versus a dinitrophenol (**1b**) standard. Reaction were conducted with 1 mM **1a** or **14a**, 2 mM DMPS, 1 µM Co-9B12 and 10% MeCN in 100 mM KPi pH 6.0. Reactions were quenched after 18 h with equal volume acetonitrile, filtered, and injected onto LC-MS. The concentration of the **1b** standard is 0.5 mM. Data collected using a C18 column and a mobile phase of MeCN:H_2O containing 0.1% formic acid.

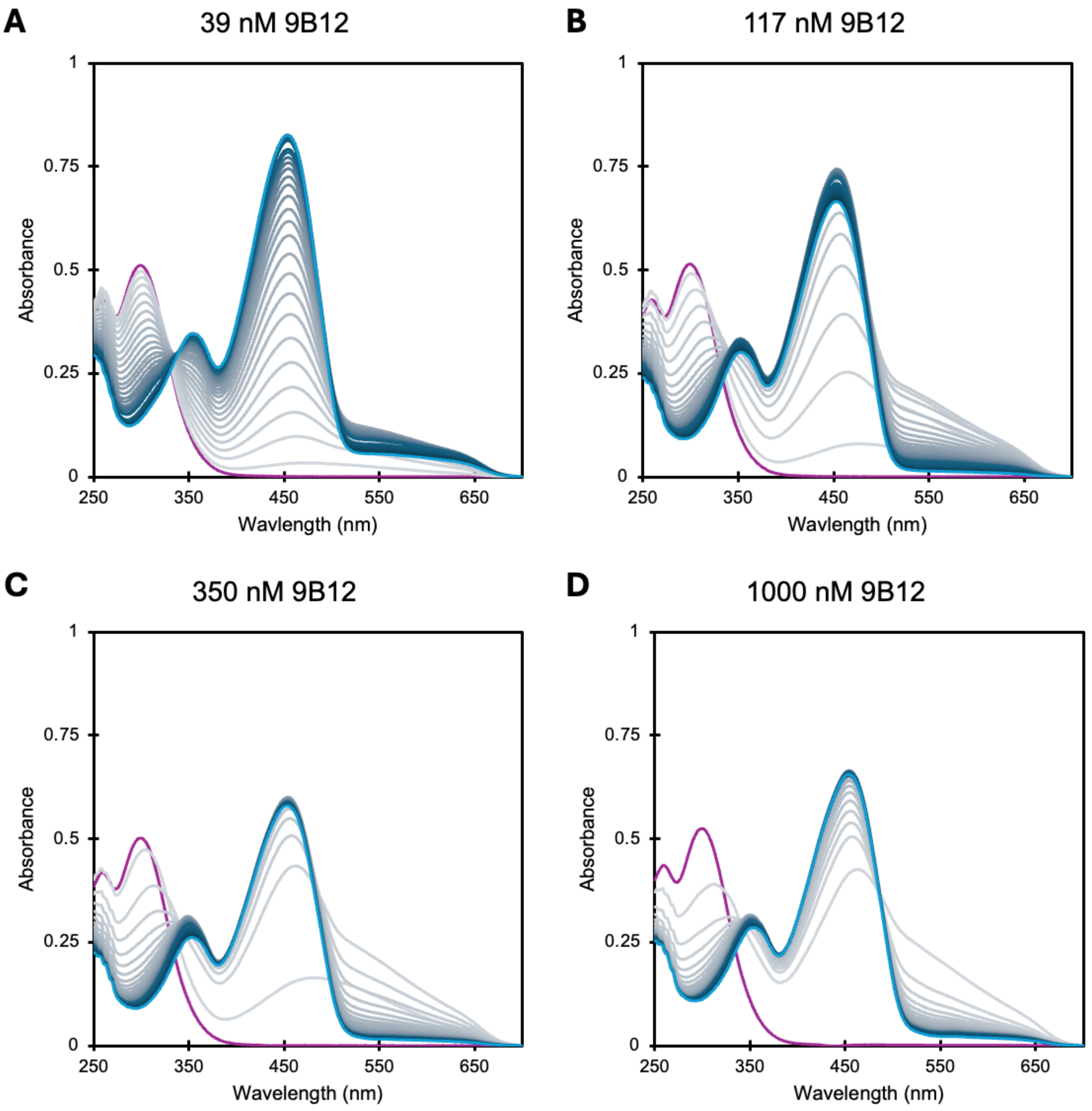

Figure S20. Time course spectra for dearomatization of 2,4-dinitroanisole **14a** (100 µM) with 9B12 (variable concentrations) and DMPS (200 µM) in potassium phosphate buffer, pH 6.0, taken over 2 hours. In each plot, the purple trace represents the absorbance spectra of the reaction just prior to addition of DMPS. The grey-blue gradient traces represent spectra taken every 3 min. The light blue trace represents the final spectra taken, at 2 h. These data were fit using singular value decomposition kinetic analysis. See section "Kinetic analysis of dearomatization time course using Kintek Explorer" for more details.

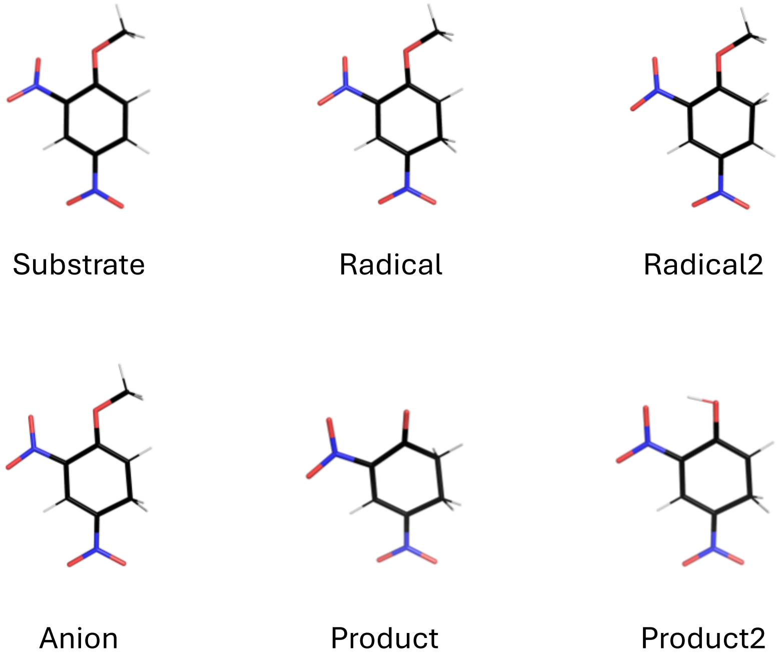

Figure S21. DFT optimized structures of the 2,4-dinitroanisole **14a**, three viable reaction intermediates (the radical species Radical and Radical2, as well as the anionic species Anion), and two possible products (the oxyanion Product and carbanion Product2). Note that the computed energy of Radical2 is 20.1 kJ/mol higher than that of Radical (consistent with the deuteration experiments described in the main text) and the computed energy of the carbanion Product2 is 26.9 kJ/mol higher than that of the oxyanion Product.

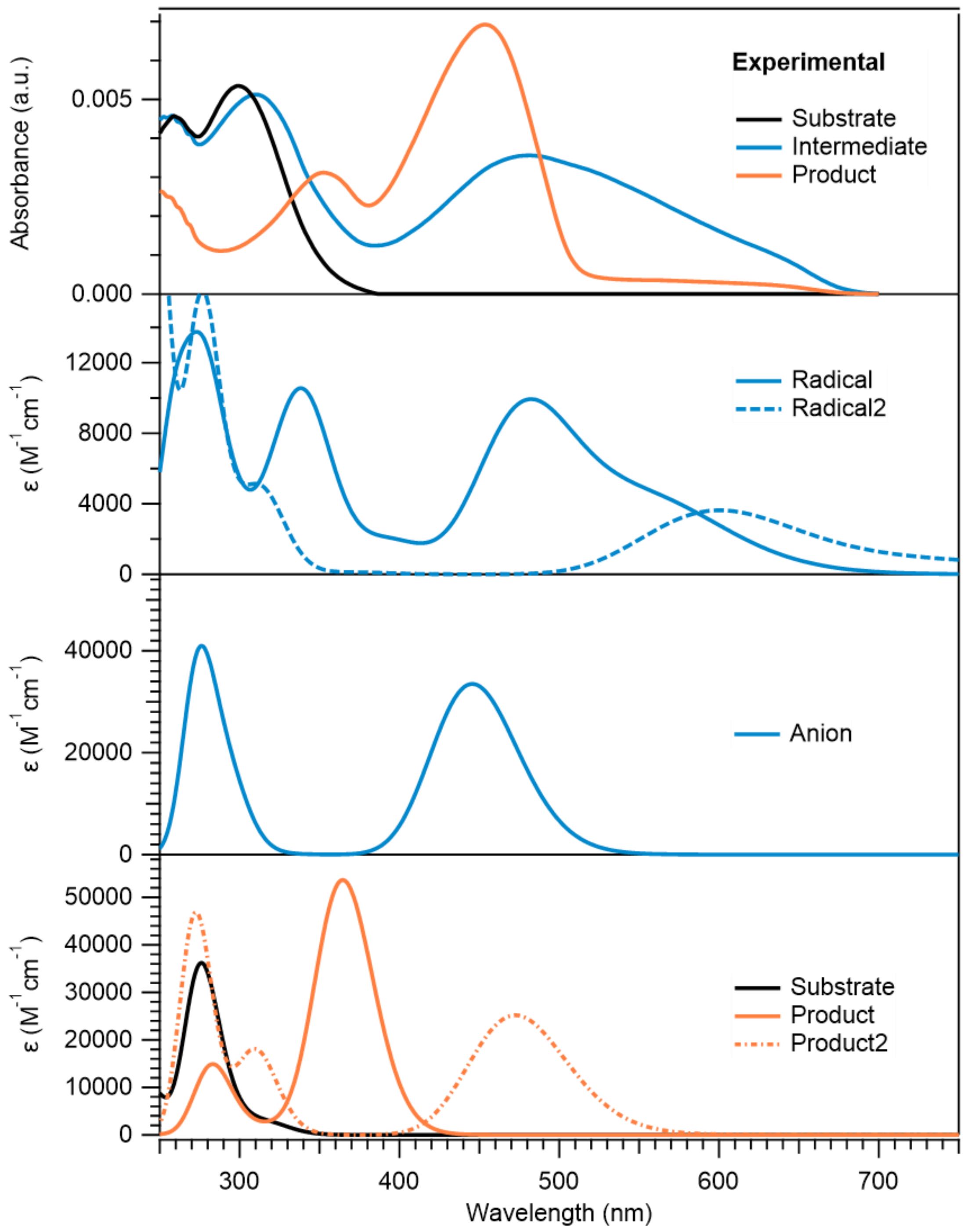

Figure S22. TD-DFT calculated spectra of putative radical and anionic reaction intermediates, and comparison to (experimental) deconvoluted intermediate spectra. Top panel: Deconvoluted electronic absorption spectra for the substrate (black), intermediate (light blue), and product (orange) for the dearomatization of the 2,4-dinitroanisole **14a** by 9B12. Bottom three panels: TD-DFT computed Abs spectra for the models of the two radical intermediates (light blue), the anion intermediate (light blue), as well as the substrate (black) and oxyanion and carbanion products (orange).

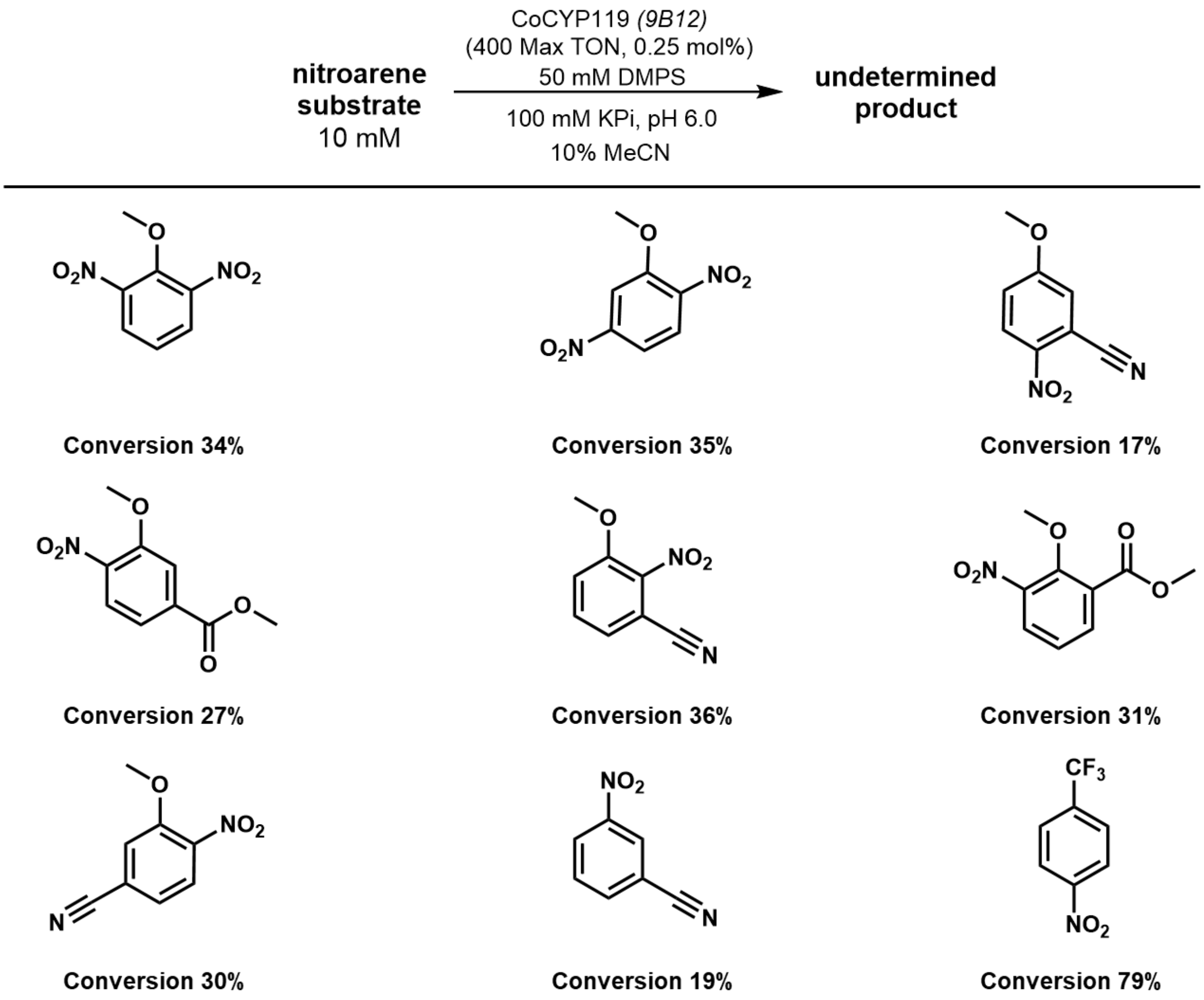

Figure S23. Scope of nitroarenes screened in the reductive dearomatization reaction for which we observed high conversion but were not able to isolate products. Conversion reported relative to a no-enzyme control as determined by LC-MS analysis and detection a 254 nm.

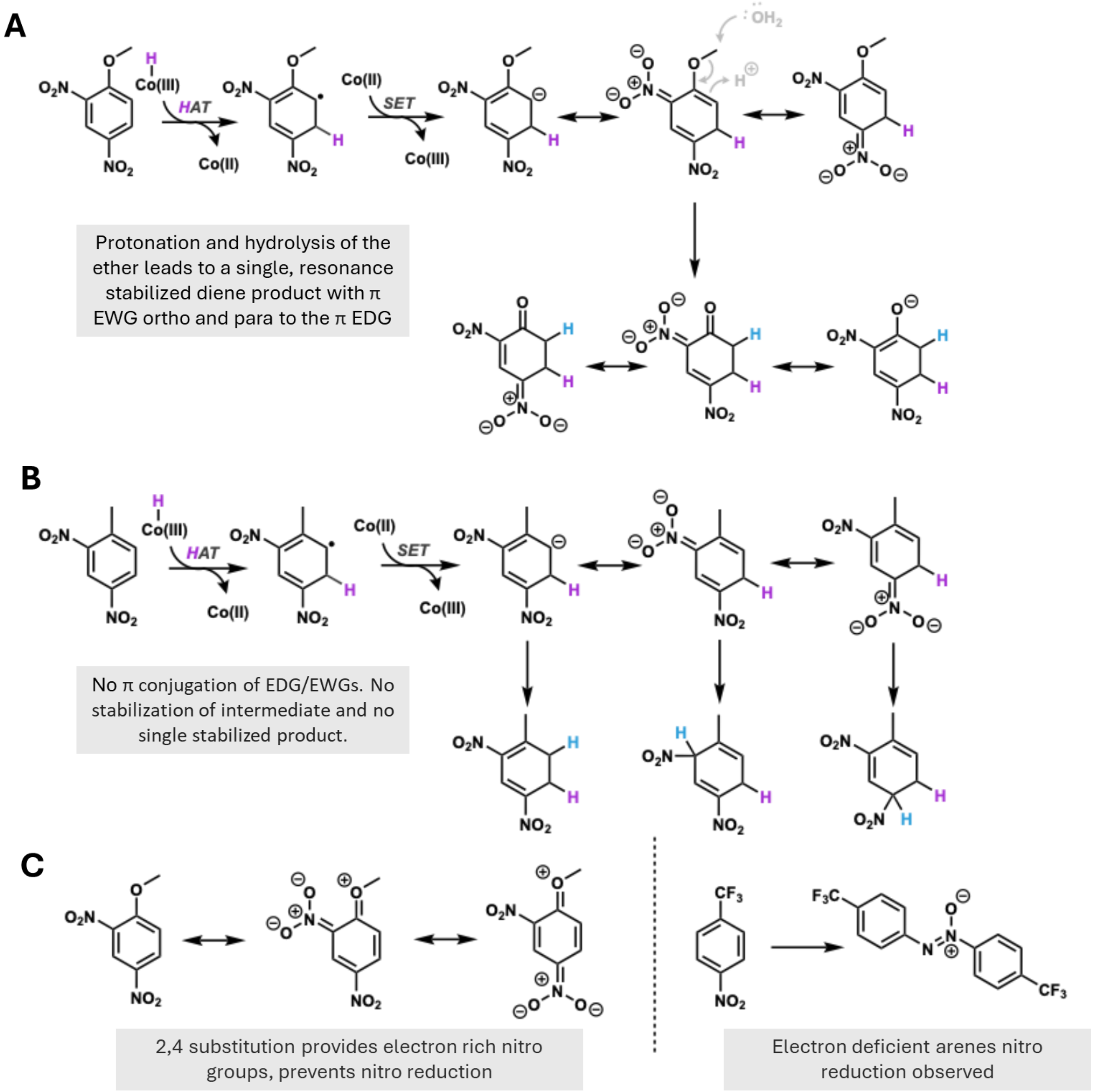

Figure S24. Stabilizing interactions favoring product outcomes for M-HAT dearomatization of nitroarenes.

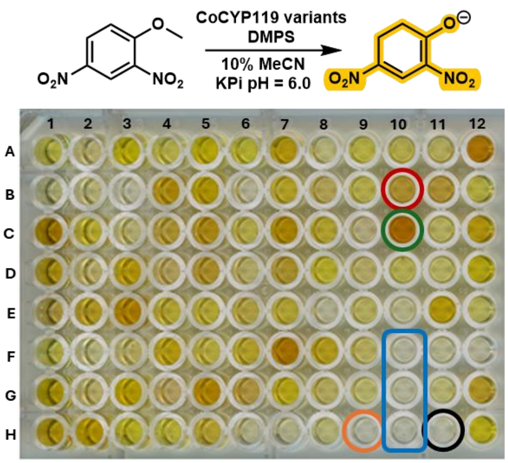

Figure S25. Image of cobalt-substituted master plate screening with dinitroanisole **14a**. Control wells have been denoted by colored circles: orange indicates a negative well where a non-heme enzyme is expressed, black indicates a sterile well, blue indicates parent wells with CoCYP119 V254D, red indicates a well spiked with purified CoCYP119 WT and green indicates a well spiked with purified CoCYP119 V254D. Additional experimental details are described in the supplemental section titled "Recombination library construction, expression, and screening".

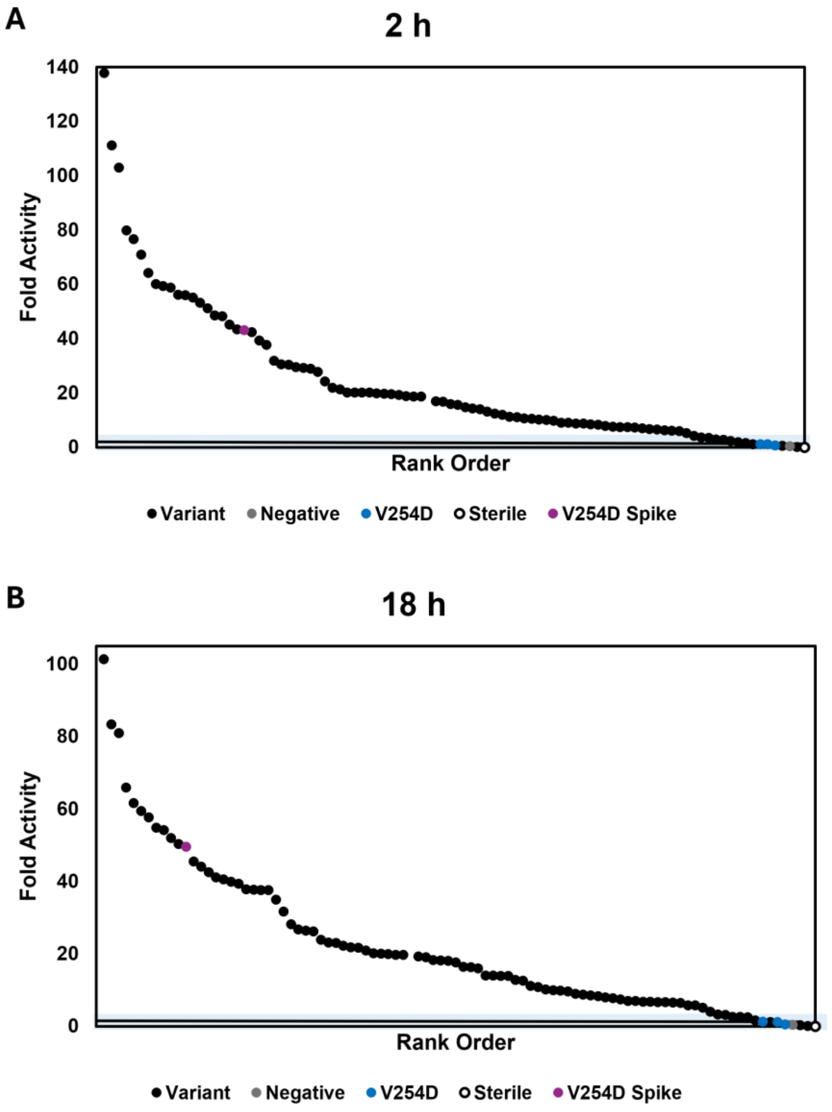

Figure S26. Retention of function curve for master plate of active recombination variants for dearomatization of 2,4-dinitroanisole **14a** with DMPS at (A) 2 h and (B) overnight (~18 h). The light blue box indicates the range of activity measured for the parent enzyme (V254D). Additional experimental details can be found in the supplemental section titled "Recombination library construction, expression, and screening".

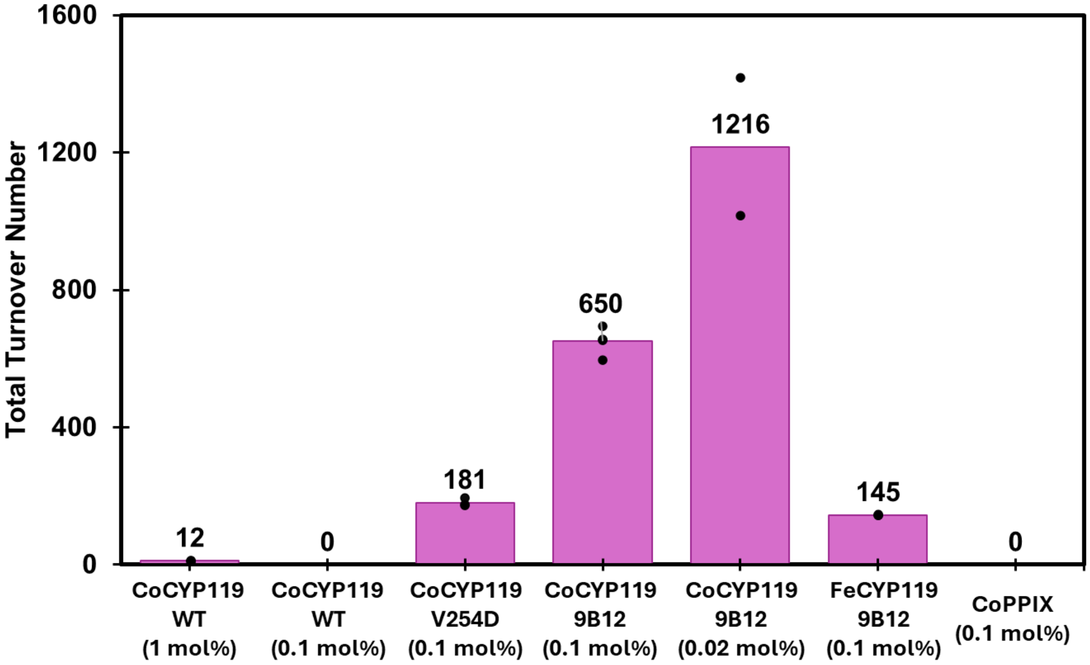

Figure S27. Dearomatization activity across the evolutionary lineage of CoCYP119 9B12, measured with **14a** and DMPS substrates. Dots represent replicate measurement data points, and bars represent the average of these data points. Average turnover numbers are reported above each bar. These measurements were made using LC-MS analysis. Additional experimental details are described in the supplemental section titled "Analytical scale reactions – yield and total turnover determination".

Supplemental Tables

Table S1. Primers for generation of site-saturation and recombination libraries. **XXX** denotes a 12:9:1 mixture of primers bearing NDT, VHG, TTG degenerate codons, respectively, for a total of 22-codons represented in the resulting libraries.[2] Degenerate codons in recombination library primers are bolded.

Protein	Forward Primer (5' to 3')	Reverse Primer (5' to 3')
pET22b(+)-CYP119	GAAATAATTTTGTTTAACTTTAAGAAGGAGATATACATATG	GCCGGATCTCAATGGTGATGGTGATGGTGCTCGAG
L69X	GACATCCCAACCCGCTATACCATG**XXX**ACGTCGGATCCG	CATGGTATAGCGGGTTGGGATGTCAAAGCGGATC
L205X	CGAATCTTTCTGACATTGAAAAATTAGGATATATCATT**XXX**TTATTGATTGCC	AATGATATATCCTAATTTTTCAATGTCAGAAAGATTCGAATTAACTACGCGGC
A209X	GGATATATCATTTTATTATTGATT**XXX**GGGAACGAGACG	AATCAATAATAAAATGATATATCCTAATTTTTCAATGTCAGAAAGATTCGAATTAACTACGC
T213X	CATTTTATTATTGATTGCCGGGAACGAG**XXX**ACTACTAATCTG	CTCGTTCCCGGCAATCAATAATAAAATGATATATCCTAATTTTTC
V254X	GGCTCTTCGCTATTCACCCCCG**XXX**ATGCGCACAGTTC	CGGGGGTGAATAGCGAAGAGCCTCTTCAATAGCTTTCAGGTAAAGG
F310X	CGTAACCCGAATCCTCATCTGAGT**XXX**GGGAGTGGG	ACTCAGATGAGGATTCGGGTTACGATCTGGAATGAACTTTTCTCCG
C317X	GTTTCGGGAGTGGGATCCACCTG**XXX**CTGGGGGC	CAGGTGGATCCCACTCCCGAAACTCAGATGAGGATTCGG
L318X	CGGGAGTGGGATCCACCTGTGC**XXX**GGGGCACC	GCACAGGTGGATCCCACTCCCGAAACTCAGATGAGG
L69[HWC]	GACATCCCAACCCGCTATACCATG**HWC**ACGTCGGATCCG	CATGGTATAGCGGGTTGGGATGTCAAAGCGGATC
L205[VWK] A209[DHC] T213[RSC]	GACATTGAAAAATTAGGATATATCATT**VWK**TTATTGATT**DHC**GGGAACGAG**RSC**ACTACTAATCTG	CTCGTTCCCGGCAATCAATAATAAAATGATATATCCTAATTTTTC

Table S2. Gene sequences used. Sequences with coding mutations relative to parent have the target mutation denoted in **red**.

Name	Sequence
CYP119 WT	ATGTACGATTGGTTTTCGGAGATGCGTAAAAAGATCCTGTTTACTACGATGGTAATA<br>TTTGGCAAGTATTTTCATATCGCTACACCAAGGAGGTATTAAACAATTTTTCAAAATT<br>TTCTTCCGATTTAACAGGGTACCACGAGCGCCTTGAGGACTTGCGCAATGGAAAGAT<br>CCGCTTTGACATCCCAACCCGCTATACCATGCTTACGTCGGATCCGCCGCTGCATGAC<br>GAATTGCGTAGTATGAGTGCCGATATCTTTTCTCCGCAAAAGTTGCAAACCCTGGAA<br>ACTTTTATTCGCGAAACGACCCGTAGTTTATTGGACTCGATTGACCCTCGCGAGGACG<br>ATATTGTTAAGAAGCTGGCCGTGCCCCTTCCAATTATCGTGATTTCCAAAATCCTGGG<br>TCTGCCAATCGAGGACAAAGAGAAGTTCAAGGAATGGAGCGACCTTGTAGCGTTTCG<br>TCTGGGCAAACCTGGCGAGATCTTCGAATTAGGAAAGAAGTATCTGGAATTGATCGG<br>ATATGTGAAAGACCATCTTAATTCAGGTACGGAAGTGGTCAGCCGCGTAGTTAATTC<br>GAATCTTTCTGACATTGAAAAATTAGGATATATCATTTTATTATTGATTGCCGGGAAC<br>GAGACGACTACTAATCTGATTAGCAATTCAGTTATTGACTTCACGCGCTTCAACTTGT<br>GGCAACGCATTCGCGAAGAAAACCTTTACCTGAAAGCTATTGAAGAGGCTCTTCGCT<br>ATTCACCCCCGGTTATGCGCACAGTTCGTAAAACCAAGGAGCGTGTAAAGTTGGGCG<br>ACCAGACGATTGAGGAAGGAGAATACGTGCGCGTCTGGATTGCATCAGCGAACCGC<br>GACGAAGAGGTCTTTCACGACGGAGAAAAGTTCATTCCAGATCGTAACCCGAATCCT<br>CATCTGAGTTTCGGGAGTGGGATCCACCTGTGCCTGGGGGCACCTTTGGCACGTTTGG<br>AAGCCCGTATTGCCATCGAGGAGTTCTCGAAGCGTTTTCGTCACATCGAAATTCTTGA<br>CACAGAAAAGGTGCCAAACGAAGTGTTGAACGGGTATAAGCGTTTGGTAGTTCGTCT<br>GAAGAGCAATGAGCTCGAGCACCATCACCATCACCATTGA
CYP119 L69H	ATGTACGATTGGTTTTCGGAGATGCGTAAAAAGATCCTGTTTACTACGATGGTAATA<br>TTTGGCAAGTATTTTCATATCGCTACACCAAGGAGGTATTAAACAATTTTTCAAAATT<br>TTCTTCCGATTTAACAGGGTACCACGAGCGCCTTGAGGACTTGCGCAATGGAAAGAT<br>CCGCTTTGACATCCCAACCCGCTATACCATG**CAT**ACGTCGGATCCGCCGCTGCATGAC<br>GAATTGCGTAGTATGAGTGCCGATATCTTTTCTCCGCAAAAGTTGCAAACCCTGGAA<br>ACTTTTATTCGCGAAACGACCCGTAGTTTATTGGACTCGATTGACCCTCGCGAGGACG<br>ATATTGTTAAGAAGCTGGCCGTGCCCCTTCCAATTATCGTGATTTCCAAAATCCTGGG<br>TCTGCCAATCGAGGACAAAGAGAAGTTCAAGGAATGGAGCGACCTTGTAGCGTTTCG<br>TCTGGGCAAACCTGGCGAGATCTTCGAATTAGGAAAGAAGTATCTGGAATTGATCGG<br>ATATGTGAAAGACCATCTTAATTCAGGTACGGAAGTGGTCAGCCGCGTAGTTAATTC<br>GAATCTTTCTGACATTGAAAAATTAGGATATATCATTTTATTATTGATTGCCGGGAAC<br>GAGACGACTACTAATCTGATTAGCAATTCAGTTATTGACTTCACGCGCTTCAACTTGT<br>GGCAACGCATTCGCGAAGAAAACCTTTACCTGAAAGCTATTGAAGAGGCTCTTCGCT<br>ATTCACCCCCGGTTATGCGCACAGTTCGTAAAACCAAGGAGCGTGTAAAGTTGGGCG<br>ACCAGACGATTGAGGAAGGAGAATACGTGCGCGTCTGGATTGCATCAGCGAACCGC<br>GACGAAGAGGTCTTTCACGACGGAGAAAAGTTCATTCCAGATCGTAACCCGAATCCT<br>CATCTGAGTTTCGGGAGTGGGATCCACCTGTGCCTGGGGGCACCTTTGGCACGTTTGG<br>AAGCCCGTATTGCCATCGAGGAGTTCTCGAAGCGTTTTCGTCACATCGAAATTCTTGA<br>CACAGAAAAGGTGCCAAACGAAGTGTTGAACGGGTATAAGCGTTTGGTAGTTCGTCT<br>GAAGAGCAATGAGCTCGAGCACCATCACCATCACCATTGA
CYP119 V254D	ATGTACGATTGGTTTTCGGAGATGCGTAAAAAGATCCTGTTTACTACGATGGTAATA<br>TTTGGCAAGTATTTTCATATCGCTACACCAAGGAGGTATTAAACAATTTTTCAAAATT<br>TTCTTCCGATTTAACAGGGTACCACGAGCGCCTTGAGGACTTGCGCAATGGAAAGAT<br>CCGCTTTGACATCCCAACCCGCTATACCATGCTTACGTCGGATCCGCCGCTGCATGAC<br>GAATTGCGTAGTATGAGTGCCGATATCTTTTCTCCGCAAAAGTTGCAAACCCTGGAA<br>ACTTTTATTCGCGAAACGACCCGTAGTTTATTGGACTCGATTGACCCTCGCGAGGACG<br>ATATTGTTAAGAAGCTGGCCGTGCCCCTTCCAATTATCGTGATTTCCAAAATCCTGGG<br>TCTGCCAATCGAGGACAAAGAGAAGTTCAAGGAATGGAGCGACCTTGTAGCGTTTCG<br>TCTGGGCAAACCTGGCGAGATCTTCGAATTAGGAAAGAAGTATCTGGAATTGATCGG<br>ATATGTGAAAGACCATCTTAATTCAGGTACGGAAGTGGTCAGCCGCGTAGTTAATTC<br>GAATCTTTCTGACATTGAAAAATTAGGATATATCATTTTATTATTGATTGCCGGGAAC<br>GAGACGACTACTAATCTGATTAGCAATTCAGTTATTGACTTCACGCGCTTCAACTTGT

	GGCAACGCATTCGCGAAGAAAACCTTTACCTGAAAGCTATTGAAGAGGCTCTTCGCT<br>ATTCACCCCCG**GAT**ATGCGCACAGTTCGTAAAACCAAGGAGCGTGTAAAGTTGGGCG<br>ACCAGACGATTGAGGAAGGAGAATACGTGCGCGTCTGGATTGCATCAGCGAACCGC<br>GACGAAGAGGTCTTTCACGACGGAGAAAAGTTCATTCCAGATCGTAACCCGAATCCT<br>CATCTGAGTTTCGGGAGTGGGATCCACCTGTGCCTGGGGGCACCTTTGGCACGTTTGG<br>AAGCCCGTATTGCCATCGAGGAGTTCTCGAAGCGTTTTCGTCACATCGAAATTCTTGA<br>CACAGAAAAGGTGCCAAACGAAGTGTTGAACGGGTATAAGCGTTTGGTAGTTCGTCT<br>GAAGAGCAATGAGCTCGAGCACCATCACCATCACCATTGA
CYP119 9B12	ATGTACGATTGGTTTTCGGAGATGCGTAAAAAAGATCCTGTTTACTACGATGGTAATA<br>TTTGGCAAGTATTTTCATATCGCTACACCAAGGAGGTATTAAACAATTTTTCAAAATT<br>TTCTTCCGATTTAACAGGGTACCACGAGCGCCTTGAGGACTTGCGCAATGGAAAGAT<br>CCGCTTTGACATCCCAACCCGCTATACCATG**CAT**ACGTCGGATCCGCCGCTGCATGAC<br>GAATTGCGTAGTATGAGTGCCGATATCTTTTCTCCGCAAAAGTTGCAAACCCTGGAA<br>ACTTTTATTCGCGAAACGACCCGTAGTTTATTGGACTCGATTGACCCTCGCGAGGACG<br>ATATTGTTAAGAAGCTGGCCGTGCCCCTTCCAATTATCGTGATTTCCAAAATCCTGGG<br>TCTGCCAATCGAGGACAAAGAGAAGTTCAAGGAATGGAGCGACCTTGTAGCGTTTCG<br>TCTGGGCAAACCTGGCGAGATCTTCGAATTAGGAAAGAAGTATCTGGAATTGATCGG<br>ATATGTGAAAGACCATCTTAATTCAGGTACGGAAGTGGTCAGCCGCGTAGTTAATTC<br>GAATCTTTCTGACATTGAAAAATTAGGATATATCATT**GTG**TTATTGATT**AGC**GGGAA<br>CGAG**GGC**ACTACTAATCTGATTAGCAATTCAGTTATTGACTTCACGCGCTTCAACTTG<br>TGGCAACGCATTCGCGAAGAAAACCTTTACCTGAAAGCTATTGAAGAGGCTCTTCGC<br>TATTCACCCCCGGTTATGCGCACAGTTCGTAAAACCAAGGAGCGTGTAAAGTTGGGC<br>GACCAGACGATTGAGGAAGGAGAATACGTGCGCGTCTGGATTGCATCAGCGAACCG<br>CGACGAAGAGGTCTTTCACGACGGAGAAAAGTTCATTCCAGATCGTAACCCGAATCC<br>TCATCTGAGTTTCGGGAGTGGGATCCACCTGTGCCTGGGGGCACCTTTGGCACGTTTG<br>GAAGCCCGTATTGCCATCGAGGAGTTCTCGAAGCGTTTTCGTCACATCGAAATTCTTG<br>ACACAGAAAAGGTGCCAAACGAAGTGTTGAACGGGTATAAGCGTTTGGTAGTTCGTC<br>TGAAGAGCAATGAGCTCGAGCACCATCACCATCACCATTGA

Table S3. Active site recombination library amino acid residues possible at each site. Total library size is 4320 possible variants.

**Site:**	**L69**	**L205**	**A209**	**T213**	**V254**
**Deg. Codon:**	HWC	VWK	DHC	RSC	-
**Mutations:**	N	K	N	T	V
	I	N	T	G	D
	H	M	I	S	
	L	I	D	A	
	Y	Q	A		
	F	H	V		
		L	Y		
		E	S		
		D	F		
		V			
**Library size:**	**6**	**10**	**9**	**4**	**2**

Table S4. Sequence identity and measured fold activities of activated variants in the recombination master plate. The variant "9B12" was in well H02 of this master plate, and is colored blue. Deallylation is abbreviated deallyl. and dearomatization is abbreviated dearom.

Master Plate Well	Recombination Plate and Well	Sequence Identity	Deallyl. (ΔA405)	Dearom. (ΔA450)	Fold Deallyl.	Fold Dearom.
**Parent**	**-**	**V254D**	**0.02**	**0.01**	**1.0**	**1.0**
A01	10A04	L69I L205V T213G	0.25	0.47	11.6	34.1
A02	11A11	L205N A209N T213G V254D	0.04	0.15	1.7	11.0
A03	12A11	L69I L205V A209S T213G	0.28	1.45	12.9	105.9
A04	13A12	L69H L205Q A209S T213G V254D	0.05	0.32	2.2	23.5
A05	14A01	L69N L205D A209S T213G	0.16	1.29	7.2	94.2
A06	15A02	L205V A209S T213G	0.15	0.49	7.1	35.5
A07	16A01	L69H L205N A209N T213G	0.13	0.96	6.2	70.4
A08	4A08	L205M A209N T213G	0.02	0.11	1.0	8.1
A09	5A08	L69Y L205I T213G	0.13	0.38	6.2	27.6
A10	6E05	L69Y L205V T213G	0.11	0.32	5.2	23.4
A11	7A07	L69I L205K A209N T213A V254D	0.01	0.22	0.4	16.3
A12	8A11	L69F L205N A209D T213A	0.22	0.95	10.4	69.5
B01	10A08	L69Y L205D A209V T213G V254D	0.01	0.14	0.6	10.5
B02	11A06	L69I L205K T213S	0.06	0.13	2.6	9.2
B03	12C02	L205K A209S T213G	0.00	0.00	0.0	0.0
B04	13C09	L69Y L205Q A209N T213G	0.05	0.92	2.3	67.4
B05	14A02	L69N L205Q T213G	0.18	1.27	8.2	93.1
B07	16B01	L69I L205K A209D T213G	0.07	0.56	3.3	40.7
B08	4B08	L69Y L205Q A209N T213G V254D	0.00	0.23	0.2	16.9
B09	5B11	L69F L205Q T213G	0.09	0.29	4.0	21.5
B11	7B06	L205N A209N T213A V254D	0.05	0.41	2.1	29.8
B12	8A04	L205E A209S T213G	0.16	0.38	7.2	27.6
C01	10B09	L69F L205D T213G	0.30	1.91	14.1	139.5
C02	11A09	L69N A209N T213G	0.05	0.54	2.5	39.1
C03	12C09	L69N L205I A209V T213G	0.03	0.13	1.6	9.2
C04	13E02	L205K T213G	0.19	0.46	9.1	33.4
C05	14B07	L69Y L205V T213G	0.20	0.74	9.3	54.1
C06	15F06	L69H L205I A209T T213G	0.06	0.29	2.6	21.1
C08	4C01	L69N L205V T213G	0.09	0.46	4.4	33.9
C09	5B02	L205V V254D	0.07	0.07	3.3	4.9
C11	7C02	L69N L205D A209I T213G	0.07	0.19	3.3	13.6
C12	8A09	L69Y L205K T213G	0.24	0.88	11.4	64.4
D01	10C07	L205Q A209D T213A V254D	0.14	0.88	6.5	64.5

D02	11C06	L205V A209S T213A	0.08	0.16	3.8	11.4
D03	12D02	L69F L205D A209S T213G	0.12	1.03	5.5	75.6
D04	13H02	L205D A209Y T213A	0.15	0.19	6.9	13.9
D06	15G10	L69Y L205D A209V T213G V254D	0.00	0.15	0.1	10.8
D07	16F12	L69Y L205M A209N T213G	0.04	0.46	1.9	34.0
D08	4C06	L69H A209N T213G	0.08	0.54	3.6	39.3
D09	5C04	L69I L205Q A209S T213S V254D	0.02	0.17	0.9	12.2
D10	6G09	L69H L205V A209T T213G	0.07	0.25	3.2	18.6
D11	7F01	L69N L205Q A209S T213G V254D	0.01	0.17	0.4	12.7
D12	8B12	L69I L205N A209S T213G	0.14	0.94	6.7	68.4
E01	10C09	L205V T213A V254D	0.13	0.42	5.9	30.8
E02	11D02	L205D A209Y T213A V254D	0.09	0.62	4.4	45.5
E03	12F04	L69Y L205D A209N T213G	0.30	1.35	14.2	99.1
E04	9D02	L69N L205D A209T T213G	0.07	0.44	3.4	32.2
E05	14H09	L69N L205I A209N T213G	0.03	0.45	1.5	32.6
E06	15H11	L69N L205Q A209D	0.07	0.32	3.2	23.6
E07	3A02	L69F L205Q A209T T213G	0.07	0.46	3.2	33.3
E08	4D12	L69N A209N T213S V254D	0.00	0.02	0.2	1.3
E09	5C09	L69F L205D A209Y T213G V254D	0.00	0.23	0.2	16.5
E10	6H08	L69I L205I T213A	0.05	0.05	2.2	3.8
E11	7F03	L205N A209D T213G	0.21	1.00	10.0	72.9
E12	8D03	L69N L205D A209S T213G	0.06	0.25	2.7	18.0
F01	10D11	L69I L205V A209S T213G	0.10	0.42	4.6	30.5
F02	11F02	L69N L205M A209V T213G	0.02	0.09	0.9	6.3
F03	12H01	L69N L205H T213A	0.07	0.20	3.5	14.5
F04	9E03	L69F L205D A209N T213G	0.08	0.88	3.6	64.7
F05	17B03	L69H L205V A209T T213G	0.07	0.37	3.4	26.9
F06	3H01	L69N L205V A209N T213G	0.03	0.50	1.5	36.9
F07	3A03	L69F L205N A209D T213G	0.13	1.22	6.1	89.2
F08	4F12	L69F L205D A209T T213G	0.10	0.66	4.4	48.0
F09	5E08	L205H A209S T213G	0.06	0.22	2.6	15.9
F11	7H03	L69H L205V T213G V254D	0.00	0.05	0.1	3.8
F12	8D07	L69N L205K A209I T213A V254D	0.02	0.03	0.9	2.1
G01	10E10	L69I L205N A209D T213G	0.12	1.40	5.7	102.2
G02	11H11	L69H L205K A209N T213G V254D	0.01	0.15	0.4	10.9
G03	9C10	L69N A209D T213G	0.15	1.96	6.9	143.7
G04	9F07	L69F L205D T213S	0.06	0.16	2.8	11.4
G06	3F08	L205I T213G V254D	0.13	0.32	6.0	23.3

G07	3D12	L205V A209N T213G	0.12	0.82	5.4	59.8
G08	4F03	L69F L205H A209Y T213G	0.06	0.20	2.7	14.9
G09	5H12	L69N L205V A209T T213G	0.03	0.18	1.3	13.0
G11	7H09	L69I L205Q A209D T213S	0.11	0.15	5.2	10.8
G12	8F04	L69N L205N A209N T213G	0.10	0.61	4.6	44.9
H01	10H07	L69N L205K A209Y T213S	0.45	1.07	20.9	78.1
H02	9B12	L69H L205V A209S T213G	0.47	2.39	21.9	174.7
H03	9D11	L69H A209S T213G	0.12	0.61	5.8	44.7
H04	9H11	L69Q L205I A209D T213A	0.12	0.42	5.5	31.0
H06	9H02	L69I L205V A209D T213S	0.03	0.05	1.5	3.7
H07	3D08	L69F L205I A209Y T213G V254D	0.02	0.06	0.8	4.6
H08	4G04	L69H L205V A209V T213G	0.04	0.14	2.1	10.4
H12	8G03	L69N L205V A209V T213G	0.04	0.88	2.0	64.5

Experimental Methods

General experimental methods

All chemicals and chemical standards were purchased from commercial suppliers (Sigma-Aldrich, VWR, Goldbio, Thermo Fisher, Frontier biosciences, Fluka), and used without further purification. Immobilized metal affinity chromatography resin was purchased from Thermofisher. Terrific Broth (TB) was purchased from DOT Scientific. Hyper Broth™ was purchased from Molecular Dimensions.

Equipment and instrumentation

Parent glycerol stocks for all growth strains were obtained from New England Biolabs (BL21(DE3) *E. coli*). New Brunswick I26R, 120 V/60 Hz shakers (Eppendorf) were used for cell growth. Electroporation (for transformation and cloning) was achieved using a Bio-Rad MicroPulser at 2,500 V. Optical density measurements of liquid cultures were recorded on an Ultrospec 10 cell density meter (Amersham Biosciences). Centrifugation was accomplished via an Avanti J-15R or J-25I centrifuge (Beckman Coulter). Preparative flash chromatographic separations were performed on an Isolera One Flash Purification system (Biotage). Ultra pressure liquid chromatography – mass spectrometry (UPLC-MS) data were collected on an Acquity UHPLC with an Acquity QDA MS detector (Waters) using a BEH C18 column (Waters). ICP-MS data were collected with an Agilent 8900 triple quadrupole ICP-MS. An Envision® 2105 multimode plate reader (Perkin Elmer) was used to measure absorbance in 96-well plates. Absorbance measurements used for kinetic studies were collected on a UV-2600 Shimadzu spectrophotometer, set to a spectral bandwidth of 0.5 nm and equipped with a Shimadzu CPS-100 6-cell temperature-controlled cell positioner. GC analysis for isobutene was conducted on an Agilent 7890A GC with dual DB5 columns (20 m ×180 μm × 0.18 μm), dual

FID detectors, and hydrogen as the carrier gas. GC analysis of reaction headspace for H_2 gas was conducted using Shimadzu GC-2010 plus with an injector temperature set to 200°C and He_2 as the carrier gas. The GC was outfitted with an RT-Q-Bond precolumn (0.53 mm inner diameter, 30.0 m length), a Plot MS5A main column (5 angstrom mol sieve packed column, 0.53 mm ID, 30.0 m), and a TCD detector. The oven was held at 60°C through the duration of elution. Nuclear magnetic resonance (NMR) spectroscopy data were collected on a Bruker 400 or 500 MHz spectrometer equipped with a BBFO or DCH cryoprobe, respectively. Data are reported as follows: chemical shift (multiplicity [singlet (s), doublet (d), doublet of doublets (dd), multiplet (m)], coupling constants [Hz], integration). All NMR spectra were recorded at ambient temperature (about 25 °C). Enantiomeric excess of an isolated product was determined by chiral HPLC by utilizing a Waters e2695 separations module with a 2998 PDA detector, Daicel CHIRALPAK® column, and HPLC grade hexanes and isopropanol. Traces were obtained by using Empower 3® software.

Cloning, expression, and protein preparations

Cloning and expression of CoCYP119 and variants

The *Sulfolobus acidocaldarius* CYP119 gene appended with a C-terminal 6-His tag and flanked by regions complementary to a pET22b vector was purchased as a codon optimized gBlock from Integrated DNA Technologies (IDT). This DNA fragment was inserted into a pET22b vector using the New England Biolabs HiFi Assembly Kit. BL21 (DE3) *E. coli* cells were subsequently transformed with the resulting cyclized DNA product by electroporation. After 45 min of recovery in Terrific Broth (TB) at 37 °C and 200 rpm, cells were plated onto Luria-Burtani (LB) agar plates with 100 μg/mL ampicillin (AMP) and incubated overnight. Single colonies were used to inoculate 10 mL TB or HyperBroth + 100 μg/mL AMP, which were grown overnight at 37 °C and 200 rpm. Expression cultures, typically 1 L of TB or HyperBroth-AMP in a 2.8 L baffled Fernbach flask, were inoculated from these starter cultures (1% inoculum, OD 3-8) followed by the addition of a few drops of antifoam 204 (Sigma Aldrich). The cultures were incubated at 37°C with shaking at 220 rpm for 6-7 hours until an OD of 6 was achieved. Prior to induction of protein expression, the cultures were placed on ice/water for 30 minutes, after which δ-ALA (250 μM), IPTG (500 μM), and $CoCl_2$ (1 mM) were added to reach the desired final concentrations. The cultures were then returned to the incubator and maintained at 25°C while shaking at 180 rpm overnight. After 18-24 hr, the cultures reached OD 4-8. Cultures were centrifuged at 4,000 x g and 4°C for 15 minutes, and the resulting pellets were stored at -20°C for later use.

Purification of CoCYP119 and variants

Cell pellets were thawed and resuspended in lysis buffer (100 mM potassium phosphate buffer (pH = 7.0), 100 mM NaCl, 1 mg/mL hen egg white lysozyme (GoldBio), 0.2 mg/mL DNase I (GoldBio), 2 mM $MgCl_2$, and 2 mM PMSF (added as 200 mM in DMSO)). A volume of 4 mL of lysis buffer per 1 gram of wet cell pellet was used. After 30 minutes of shaking at 37 °C and 200 rpm, the cells were subjected to a 65 °C heat treatment in a water bath for 45 min. The resulting lysate was clarified of cellular debris by centrifugation at 75,000xg for 15 min, resulting in an orange-red supernatant. The supernatant was applied to a gravity column of Ni/NTA beads (GoldBio) that was equilibrated with 100 mM potassium phosphate buffer (pH = 7.0). The column was washed with approximately 3 column volumes of 50 mM imidazole, 100 mM NaCl, 100 mM potassium phosphate buffer (pH = 7.0). CoCYP119 was eluted with 250 mM imidazole, 100 mM NaCl, 100 mM potassium phosphate buffer (pH = 7.0). Elution of the desired protein was monitored by the disappearance of the red color from the column. The eluent was iteratively dialyzed into storage buffer (100 mM potassium phosphate buffer, pH = 6.0) while stirring at room temperature until the imidazole concentration was <0.01 mM (about five 100-fold dialyses). Excess imidazole was found to inhibit subsequent reactions. Dialyzed protein solutions were concentrated to <2 mL using Amicon® Ultra 15 mL Centrifugal Filters (Millipore) with a molecular weight cutoff of 30 kDa. Purified enzyme was flash frozen in pellet form by pipetting enzyme dropwise into a crystallization dish filled with liquid nitrogen. The enzyme pellets were transferred to a plastic conical and stored at -80 °C until further use.

Preparation of 9B12 heat-treated lysate

Cell lysis was performed by preparing a lysis buffer (4 mL per gram of cell pellet) containing 100 mM KPi (pH 6.0), 100 mM NaCl, 1 mg/mL lysozyme, 0.2 mg/mL DNase I, 2 mM $MgCl_2$, and 2 mM PMSF (added as 200 mM in DMSO). The cell pellets were incubated in this buffer for 30 minutes at 37°C, followed by heat treatment at 65°C for 50 minutes. The lysate was then centrifuged at 25,000xg for 30 minutes.

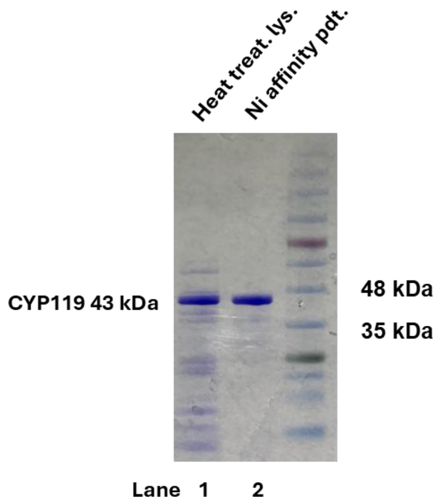

Figure S28. SDS-PAGE analysis of CoCYP119 9B12 (43 kDa). Lane 1: heat-treated lysate; Lane 2: Ni affinity-purified product.

The resulting supernatant was concentrated using a 30kDa MW cutoff centrifugal filtration device and dialyzed into 100 mM KPi pH = 6.0, with the buffer changed four times. The final concentration of the lysate was adjusted to 50 μM CoCyp119 9B12, as determined by a cobalt-specific pyridine hemochromagen assay, as described below. Finally, the lysate was flash frozen for use in scale-up procedures (Lane 1) or further processed through nickel affinity chromatography for protein purification (Lane 2).

Table S5. Protein yields obtained for CoCYP119 WT and 9B12.

Variant	Yield (mg/L culture)
WT	71
9B12	33

Modified pyridine hemochrome assay for cofactor-loaded protein quantification

A modified version of the pyridine hemochrome assay was used to quantify the concentration of CoPPIX or heme in purified protein samples.[3] Protein solution (20 μL) was added to 380 μL of pyridine hemochrome reagent (40 mL pyridine, 36 mL H2O, and 4 mL 1.0 M NaOH) in a quartz cuvette (Starna). The sample was capped and mixed thoroughly to form the pyridine metalloporphyrin complex. A few crystals of sodium dithionite were added and mixed thoroughly again. Spectra were recorded at 25 °C. The concentration of CoPPIX-loaded CYP119 was calculated using the background corrected absorbance at 556 nm and the extinction coefficient of the pyridine CoPPIX complex (ε_{556} = 9.7 mM^{-1}) or the pyridine heme complex (ε_{556} = 32 mM^{-1}).

ICP-MS analysis of CoCYP119 samples

ICP-MS analysis was conducted based on a previous procedure.[4] CoCYP119 samples were digested by addition of 145 μL of trace analysis grade 70% nitric acid to protein stocks (200 to 400 μL). Samples were incubated at 90°C for an hour to ensure complete digestion, subsequently diluted with >18.2 MΩ water to give a final concentration of 1% nitric acid, and filtered before analysis. Cobalt and iron standard solutions (0 ppb, 10 ppb, 50 ppb, 100 ppb, 500 ppb, 800 ppb) were prepared in 1% nitric acid solutions from 1000 ppm atomic absorption standards. Cobalt and iron content of protein and standard solutions was quantified by tandem mass spectrometry via detection of ions with m/z of 59→59 ion and 56→56 ion while using oxygen as the reaction gas.

Spectroscopic analysis of CoCYP119 metallocofactor content: spectral deconvolution

The metalation of PPIX confers unique spectral features in the Q bands (450-600 nm) (Fig S29 A) and these differences can be leveraged to determine the metal loading of an enzyme sample. UV-vis spectra were recorded at 25 °C for 1 μM samples of protoporphyrin IX (MedChemExpress), hemin chloride (Sigma) and protoporphyrin IX cobalt chloride (Sigma) using the modified pyridine hemachrome assay (above). A linear combination of these standard spectra were compared the pyridine hemochrome spectrum of each isolated protein sample and a residual (R^2, the squared difference between the linear combination of standard spectra and the protein sample spectrum) was calculated for every wavelength between 450 and 600 nm and summed. Using the Equation Solver Add-in on Excel, standardsthe sum of the R^2 values was minimized, by augmenting the scalar by which each standard spectrum was multiplied . From these scaling factors and known extinction coefficinents (pyridine CoPPIX complex, ε_{556} = 9.7 mM^{-1}; pyridine heme complex, ε_{556} = 32 mM^{-1}) the fraction of each MPPIX was determined. This method was used for quality control to ensure that enzyme stocks freshly prepared were ≥95% Co-loaded before usage. An example of the output of this method is shown below for a sample of CoCYP119 9B12 (Fig S29 B).

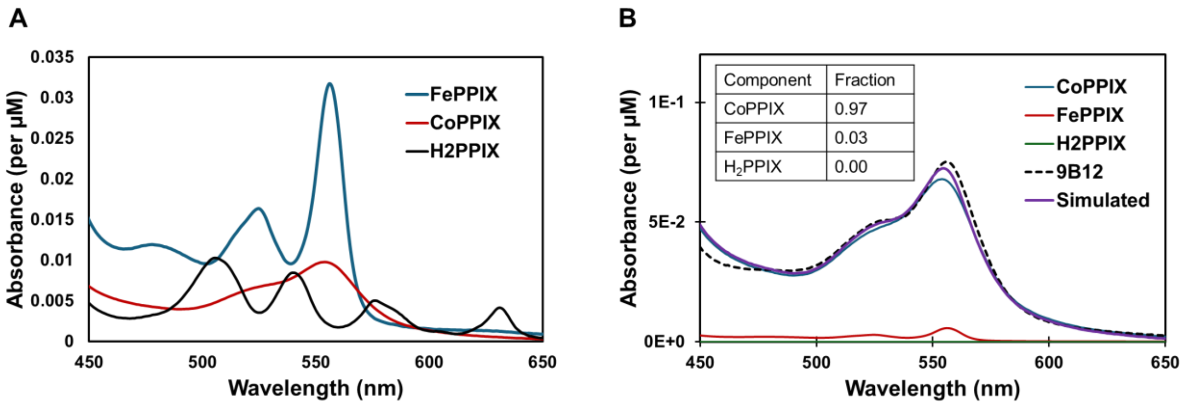

Figure S29. Spectral deconvolution to quantify cofactor metal loading. (A) Difference in Q-band spectral features for pyridine hemachrome complexes of hemin chloride (blue trace), protoporphyrin IX cobalt chloride (red trace) and protoporphyrin IX (black trace). (B) Example of spectral deconvolution being used to check adequate cobalt loading in a sample of CoCYP119 9B12. Q-band region of standard spectra for CoPPIX (blue trace), FePPIX (red trace) and unmetalated PPIX (black trace) are determined to make a simulated spectrum (purple trace) that matches the experimental spectrum (black dashed trace) for a protein sample.

Mechanistic interrogations of initial enzymatic M-HAT

Measurement of initial rates and progress curves using UV-vis

Measurement of rates and time courses using a cuvette-style spectrophotometer

Organic solvent (MeCN or DMSO) and allyl substrate were added to a quartz UV cuvette (Starna). The appropriate volume of buffer (potassium phosphate buffer pH = 6.0) and enzyme was added, and the cuvette was inverted to mix. Silane, suspended in organic solvent, was added to initiate the reaction, and the cuvette was once again inverted to mix. Product was detected via absorbance at 405 nm. These absorbance values were compared to the extinction coefficient of products 2,4-dinitrophenol (**1b**) and 4-nitrophenol (**4b**) to determine concentration. Standard curves for **1b** and **4b** were measured in 20% DMSO in 100 mM KPi pH = 6.0 or 10% acetonitrile in 100 mM KPi pH = 6.0, matching the reaction conditions for each experiment.

Measurement of initial rates using a plate reader spectrophotometer

Reaction plates were assembled in triplicate using a multichannel pipette. First, potassium phosphate buffer (100 mM, pH = 6.0) was added to each well, followed by the appropriate volume of enzyme. The solution was mixed thoroughly by pipetting. To initiate the reaction, silane and allyl substrate was added as a solution in DMSO and the solution was quickly mixed before loading into the UV-vis plate reader and measuring absorbance every 15 seconds. Product was detected via absorbance at 405 nm. These absorbance values were compared to the standard curve for 2,4-dinitrophenol (**1b**), which was measured under identical conditions.

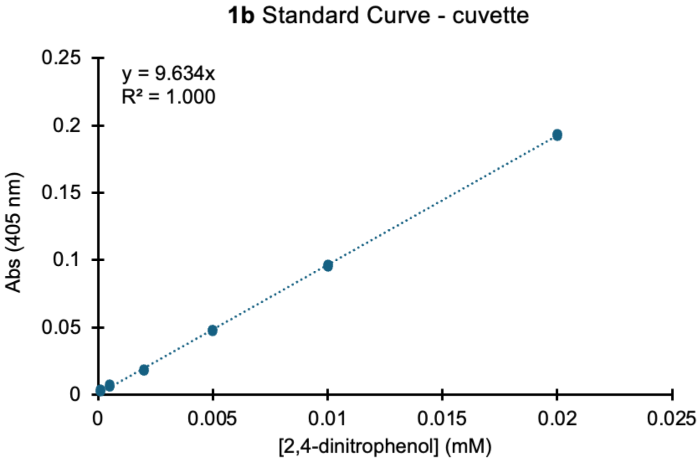

Figure S30. Standard curve for **1b** measured in 20% DMSO/KPi pH 6.0, measured in a cuvette-style spectrophotometer. Calculated extinction coefficient for **1b** at 405 nm is 9630 $M^{-1}cm^{-1}$.

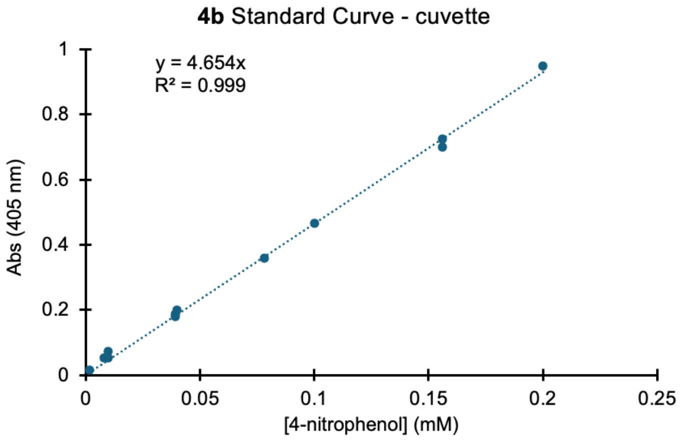

Figure S31. Standard curve for **4b** measured in 10% Acetonitrile/KPi pH 6.0, measured in a cuvette-style spectrophotometer. Calculated extinction coefficient for **4b** at 405 nm is 4654 $M^{-1}cm^{-1}$.

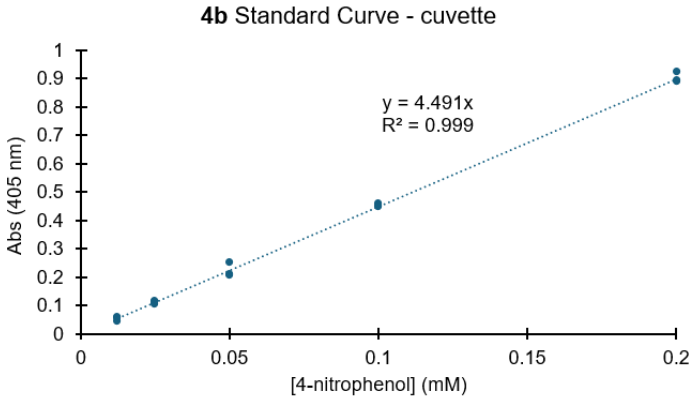

Figure S32. Standard curve for **4b** measured in 20% DMSO/KPi, pH 6.0 measured in a cuvette-style spectrophotometer. Calculated extinction coefficient for **4b** is 4491 M^{-1} cm^{-1}.

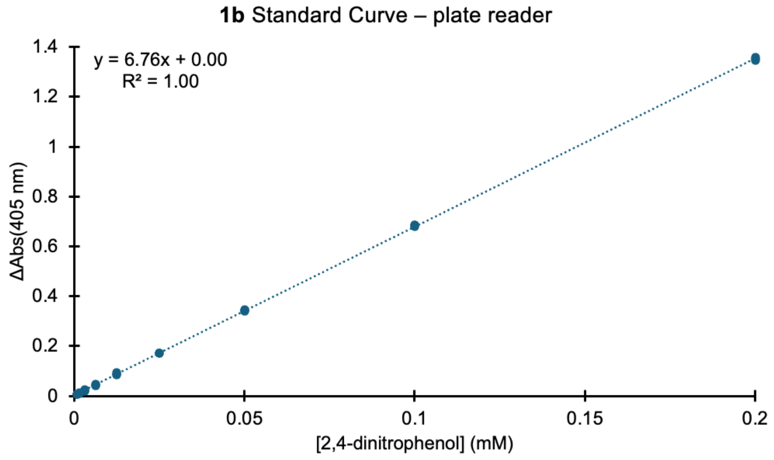

Figure S33. Standard curve for **4b** measured in 20% DMSO/KPi pH 6.0, measured in a plate reader spectrophotometer.

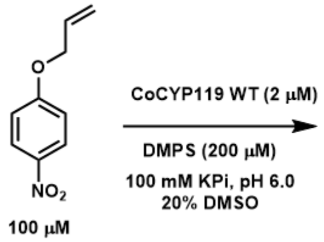

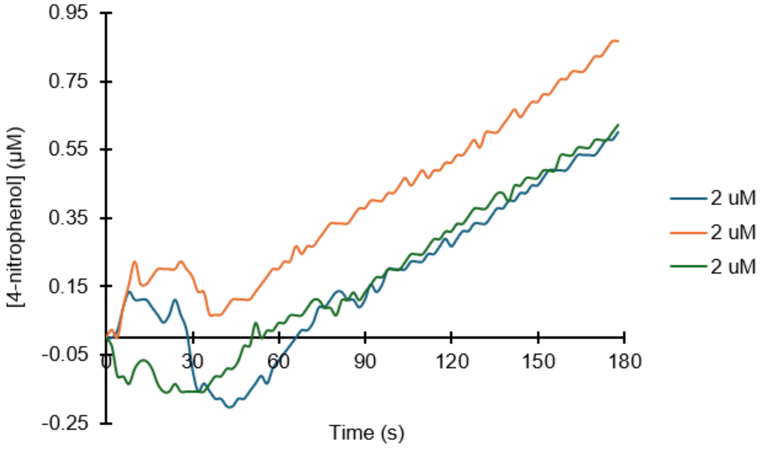

Figure S34. Initial velocity measurement of **2a** (1-(allyloxy)-2,4-dinitrobenzene, 100 μM), CoCYP119 WT (2 μM), and DMPS (200 μM). Scattering due to mixing effects introduced noise early in the time courses. Initial rates were calculated after 60 s. Reaction was performed in triplicate with the same enzyme loading (2 μM).

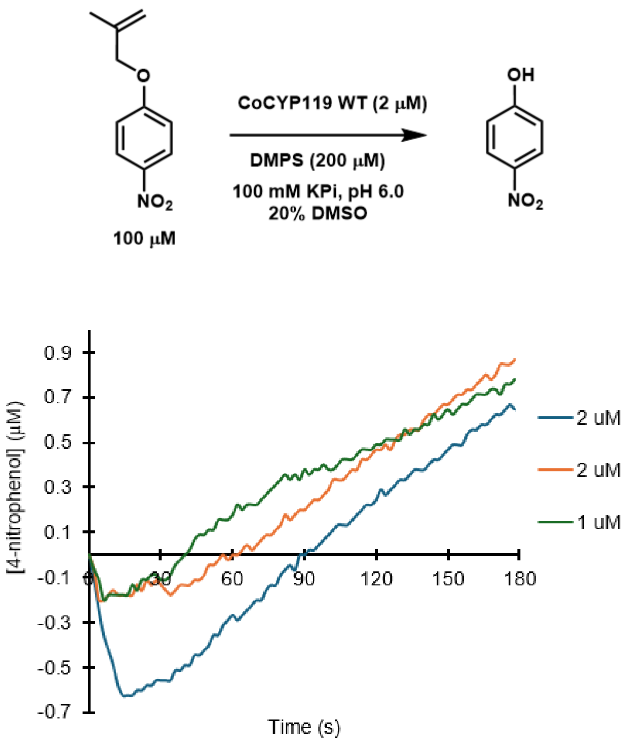

Figure S35. Initial velocity measurement of **4a** (100 µM), CoCYP119 WT (1 µM or 2 µM), DMPS (200 µM). Scattering due to mixing effects introduced noise early in the time courses. Initial rates were calculated after 60 s. Reaction was performed in triplicate with two different enzyme loadings (1 µM or 2 µM).

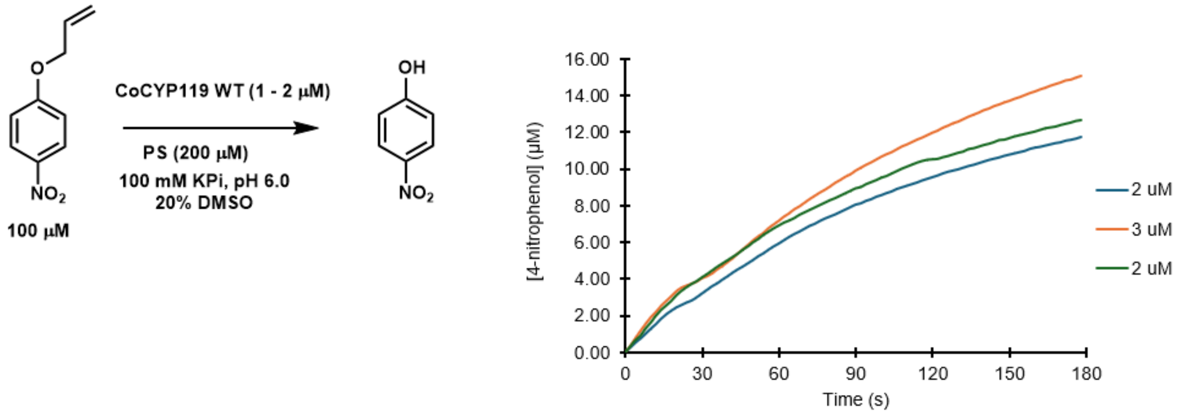

Figure S36. Initial velocity measurement of **2a** (100 µM), CoCYP119 WT (1 µM or 2 µM), PS (200 µM). Initial rates were calculated from 0 to 60 s. Reaction was performed in triplicate with two different enzyme loadings (1 µM or 2 µM).

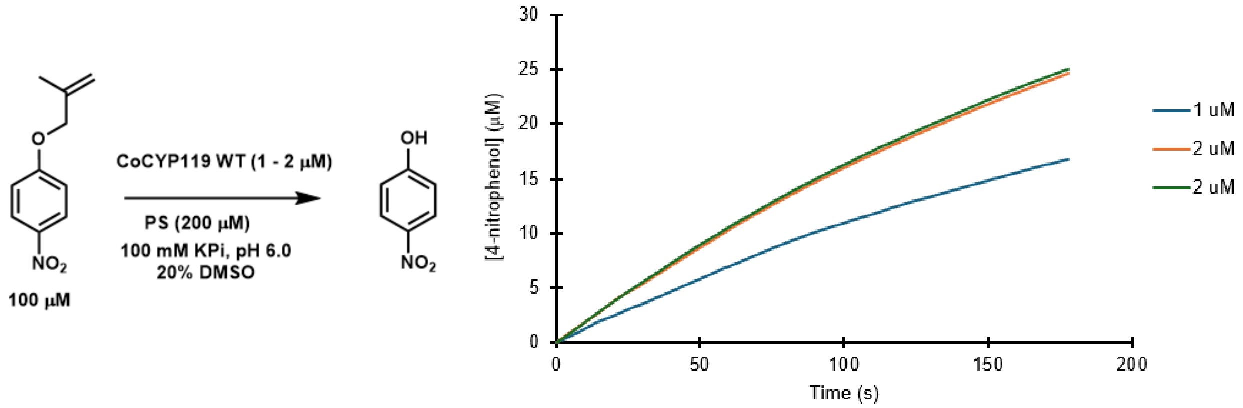

Figure S37. Initial velocity measurements of 4a (100 µM), CoCYP119 WT (1 µM or 2 µM), PS (200 µM). Initial rates were calculated from 0 s to 20 s. Reaction was performed in triplicate with two different enzyme loadings (1 µM or 2 µM).

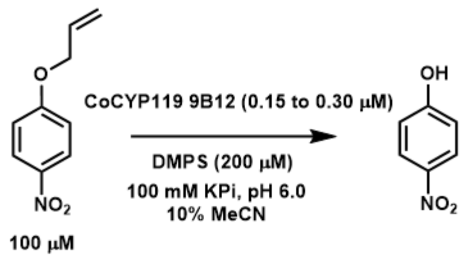

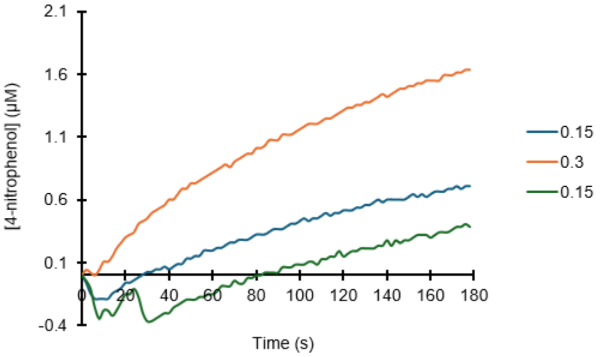

Figure S38. Initial velocity measurement of **2a** (100 μM), CoCYP119 9B12 (0.15 μM or 0.30 μM), DMPS (200 μM). Initial rates were calculated from 30 s to 60 s. Reaction was performed in triplicate with two different enzyme loadings (0.30 μM or 0.15 μM).

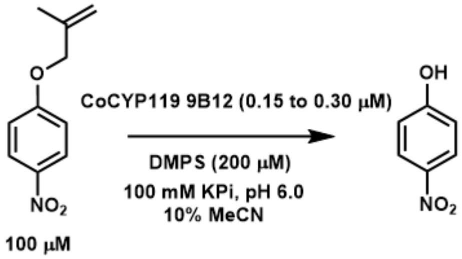

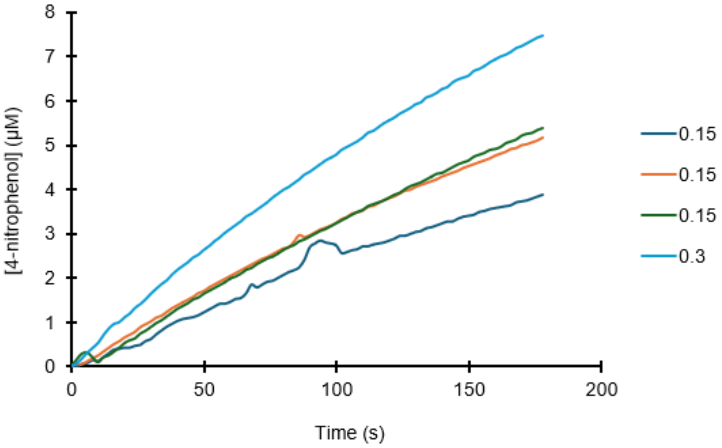

Figure S39. Initial velocity measurement of **4a** (100 μM), CoCYP119 9B12 (0.15 μM or 0.30 μM), DMPS (200 μM). Initial rates were calculated from 0 s to 60 s. Reaction was performed in triplicate with two different enzyme loadings (0.30 μM or 0.15 μM).

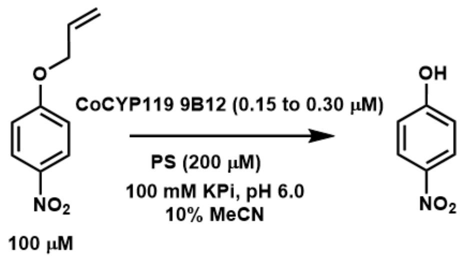

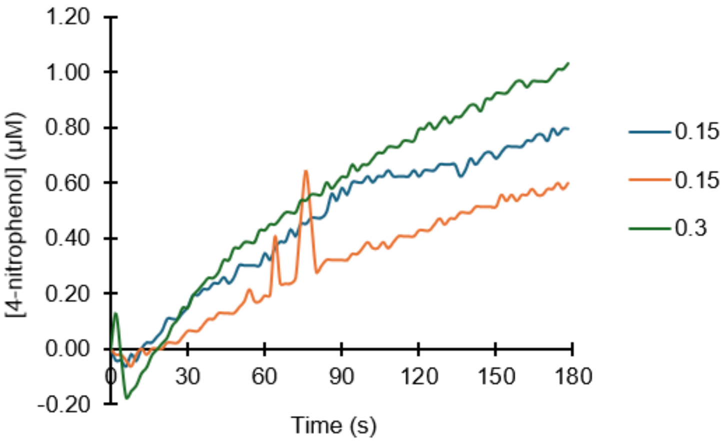

Figure S40. Initial velocity measurement of **2a** (100 μM), CoCYP119 9B12 (0.15 μM or 0.30 μM), PS (200 μM). Initial rates were calculated from 16 s to 50 s. Spikes in absorbance in the orange trace were observed periodically throughout data collection and included here as a representation of normal experimental artifact. Such spikes may be caused by transient scattering effects, potentially bubbles of H2, that occur during the reaction. Reaction was performed in triplicate with two different enzyme loadings (0.30 μM or 0.15 μM).

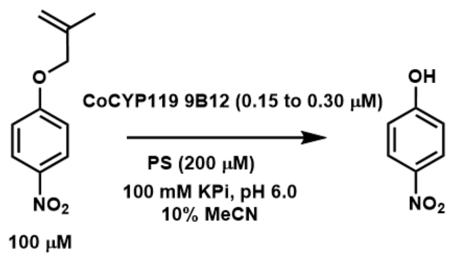

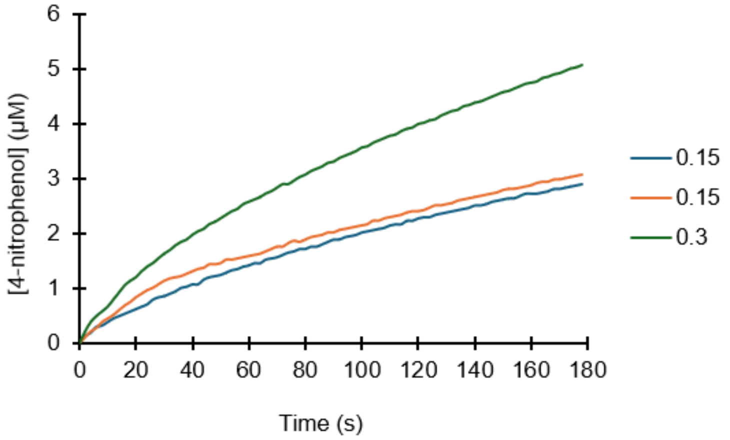

Figure S41. Initial velocity measurement of **4a** (100 μM), CoCYP119 9B12 (0.15 μM or 0.30 μM), PS (200 μM). Initial rates were calculated from 0 s to 30 s. Reaction was performed in triplicate with two different enzyme loadings (0.30 μM or 0.15 μM).

Directed evolution and screening

SSM library construction, expression, and screening

The 22-codon trick[2] was used to construct site saturation libraries at Leu69, Leu155, Leu205, Ala209, Thr213, Val254, Phe310, Cys317 and Leu318 in CYP119. Three primers with the codons NDT, VHG, and TGG were ordered from IDT and mixed in a 12:9:1 ratio. Primer solutions were subsequently used for single overlap PCR amplification of a pET22b plasmid bearing the CYP119 gene. The resulting linearized plasmid was purified by gel electrophoresis, and extracted using the Macherey-Nagel Gel DNA Recovery Kit. The linearized vector was circularized by the Gibson Assembly method.[5] *E. coli* cells were transformed with the resulting cyclized DNA product by electroporation. After 45 min. of recovery in TB media at 37 °C and 200 rpm, cells were plated onto LB agar plates with 100 μg/mL AMP and incubated overnight.

A 96-deep well plate containing TB + 100 μg/mL AMP (500 μL) was inoculated by a single colony in each well and starter plates were grown overnight at 37 °C and 200 rpm. Wells A5, B5, G5, and H5 were left sterile. Wells A6, B6, G6, and H6 were chosen as negative controls in which a non-heme enzyme was expressed (such as *Pf*TrpB^{2B9} or UstD^{QE}). Wells A7, B7, C7, F7, G7, and H7 were inoculated with parent CoCYP119 WT. Expression cultures in a fresh 96-deep well plate containing TB + 100 μg/mL AMP (650 μL) were inoculated using starter culture (50 μL) and grown at 37 °C and 200 rpm until cultures reached an OD_{600} of at least 1.0 (about 5 hours). Expression cultures were chilled on ice for 30 min and then induced with 50 μL of an aqueous solution containing 3.75 mM 5ALA, 15 mM $CoCl_2$, 15 mM IPTG (final concentrations of 0.25 mM, 1 mM, and 1 mM, respectively). Cultures were expressed overnight at 25 °C and 180 rpm. The following day, the plate was spun down at 4,000xg for 15 min at 25 °C and the pellets were frozen at -20 °C until use.

Frozen cell pellets in a 96-deep well plate were thawed and resuspended in 300 μL of 100 mM potassium phosphate buffer (pH 6.0). After applying an Alumaseal sealing film (VWR) to the plate with a rubber roller (VWR), the plate was placed in a water bath at 65 °C for 15 min. The resulting lysate was clarified of cellular debris by centrifugation at 4,000xg at 25 °C for 20 min.

Organic reaction master mix was created by adding 25 mM PS and 2.5 mM of substrate **1a** to DMSO (final volume of 6.25 mL). A flat bottom polystyrene 96-well microplate (Corning) was charged with 50 μL organic master mix in each well. Reactions were initiated by the addition of 200 μL clarified lysate to each well of the 96-well plate (final concentrations in the reaction were 5 mM PS, 0.5 mM **1a**, and 20% DMSO). As a positive control, 2-4 μM purified CoCYP119 WT was added to well F7. Reaction progress was tracked by recording the absorbance at 405 nm on the plate reader (1 scan every 30 s, 100 total scans).

Recombination library construction, expression, and screening

Degenerate codons were designed to include activating mutations found in SSM screening at Leu69, Leu205, Ala209, and Thr213 in CYP119 (see Table S3). Primers with these degenerate codons were ordered from IDT (primers sequences are given in Table S1). Primer solutions were subsequently used for single overlap PCR amplification of a 1:1 mixture of pET22b plasmids bearing the CYP119 WT and CYP119 V254D genes. The resulting linearized plasmid was purified by gel electrophoresis, and extracted using the Macherey-Nagel Gel DNA Recovery Kit. The linearized vector was circularized by the Gibson Assembly method.[5] *E. coli* cells were transformed with the resulting cyclized DNA product by electroporation. After 45

minutes of recovery in TB media at 37 °C and 200 rpm, cells were plated onto LB agar plates with 100 μg/mL AMP and incubated overnight.

A 96-deep well plate containing TB + 100 μg/mL AMP (500 μL) was inoculated by a single colony in each well and starter plates were grown overnight at 37 °C and 200 rpm. Wells A5 and B5 were left sterile. Wells G5 and H5 were chosen as negative controls in which a non-heme enzyme was expressed (such as *Pf*TrpB^{2B9} or UstD^{QE}). Wells C5, D5, E5 and F5 were inoculated with parent CoCYP119 V254D. Expression cultures in a fresh 96-deep well plate containing TB + 100 μg/mL AMP (650 μL) were inoculated using starter culture (50 μL) and grown at 37 °C and 200 rpm until cultures reached an OD_{600} of at least 1.0 (about 5 hours). Expression cultures were chilled on ice for 30 min and then induced with 50 μL of an aqueous solution containing 3.75 mM 5ALA, 15 mM $CoCl_2$, 15 mM IPTG (final concentrations of 0.25 mM, 1 mM, and 1 mM, respectively). Cultures were expressed overnight at 25 °C and 180 rpm. The following day, the plate was spun down at 4,000xg for 15 min at 25 °C and the pellets were frozen at -20 °C until use.

Frozen cell pellets in a 96-deep well plate were thawed and resuspended in 400 μL 100 mM potassium phosphate buffer (pH = 6.0). After applying an Alumaseal sealing film (VWR) to the plate with a rubber roller (VWR), the plate was placed in a water bath at 65 °C for 15 min. The resulting lysate was clarified of cellular debris by centrifugation at 4,000xg at 25 °C for 20 min.

Organic reaction master mix was created by adding 10 mM DMPS and 10 mM **1a**, **4a**, or **14a** to MeCN (final volume of 4 mL per plate). A flat bottom polystyrene 96-well microplate (Corning) was charged with 25 μL organic master mix and 25 μL 100 mM KPi pH = 6.0 in each well. Reactions were initiated by the addition of 200 μL clarified lysate to each well of the 96-

well plate (final concentrations in the reaction were 1 mM DMPS, 1 mM **1a**, **4a**, or **14a**, and 10% MeCN). As a positive control, 2-4 μM purified CoCYP119 V254D was added to well E5. A sealing film was applied to the reaction plate with a rubber roller, reaction time points were recorded at 1 hour and 6 hours by recording the absorbance at 405 nm for deallylation screening or 450 nm for dearomatization screening, and the reaction continued overnight at room temperature. End point absorbance measurements were taken after 18 h.

Analytical scale reactions – yield and total turnover determination

LC-MS analysis – deallylation

A 1.5 mL microcentrifuge tube was loaded with 100 mM potassium phosphate buffer (pH = 6.0, final reaction volume of 100 μL). CoCYP119 variant (usually 1 μM, 0.01 mol% catalyst, 1,000 max TTN) or FeCYP119 9B12 (10 μM, 0.01 mol% catalyst, 1,000 max TTN) was thawed and added to the tube. An organic master mix was created by adding substrate and DMPS to MeCN (10 mM and 20 mM, respectively for reactions with Co-loaded enzymes, and 100 mM and 200 mM, respectively for reactions with Fe9B12). Reactions were initiated by the addition of 10 μL organic master mix (1 mM substrate and 2 mM DMPS final concentrations for reactions with Co-loaded enzymes, and 10 mM substrate and 20 mM DMPS for Fe9B12 reactions, 10% MeCN) and were left at room temperature (or 40 °C for Fe9B12 reactions) for 18 h. Reactions were quenched by the addition of 1 volume of acetonitrile (final volume of 200 μL). Quenched reactions were spun down at 15,000xg for 10 minutes prior to injection on UPLC-MS. The amount of product was quantified by comparison to a standard curve (see below).

UV-vis analysis – plate reader – deallylation

Reaction plates were assembled in triplicate using a multichannel pipette. First, potassium phosphate buffer (100 mM, pH = 6.0) was added to each well, followed by the

appropriate volume of enzyme. The solution was mixed thoroughly by pipetting. To initiate the reaction, silane and allyl substrate (**1a)** was added as a solution in DMSO and the solution was mixed and then incubated at room temperature for 1 hour for PS or 6 hours for DMPS. Product was quantified via absorbance at 405 nm. These absorbance values were compared to the standard curve for 2,4-dinitrophenol (**1b**), which was measured under identical conditions (see Figure S32).

Construction of standard curves for deallylation scope and TTN experiments

All standard curve samples for each deallylation product and the dearomatized product of 2,4-dinitroanisole were made using 55% MeCN and 45% 100 mM KPi pH = 6.0 containing 0.5 mM of the internal standard to match the matrix of quenched deallylation reactions. Starting from three independent 2 mM stock solutions of the standard, serial 1:2 dilutions were carried out to make solutions ranging from 1 mM to 0.0625 mM in triplicate. Each sample was analyzed by UPLC with 3 µL injections, unless otherwise specified. Standard curves were constructed from the response ratio, or standard peak area divided by the internal standard peak area, for each sample.

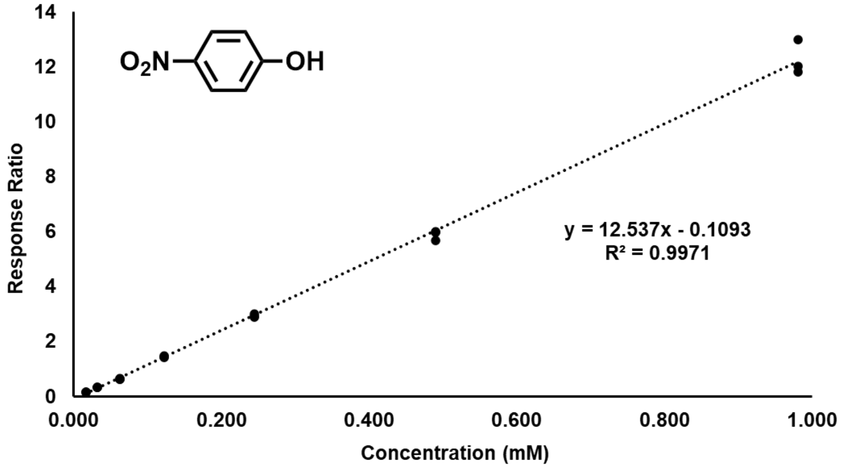

Figure S42. Standard curve for 4-nitrophenol **4/5b**. Phenol was used as the internal standard and data were collected using absorbance at 254 nm with a C18 column and a mobile phase of MeCN:H_2O containing 0.1% formic acid.

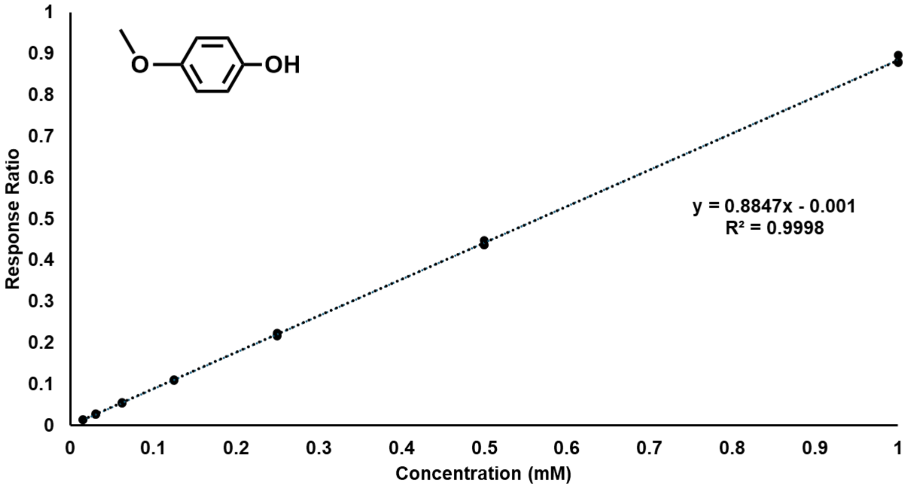

Figure S43. Standard curve for 4-methoxyphenol **6/11b**. 4-nitrophenol was used as the internal standard and data were collected at 254 nm with a C18 column and a mobile phase of MeCN:H_2O containing 0.1% formic acid.

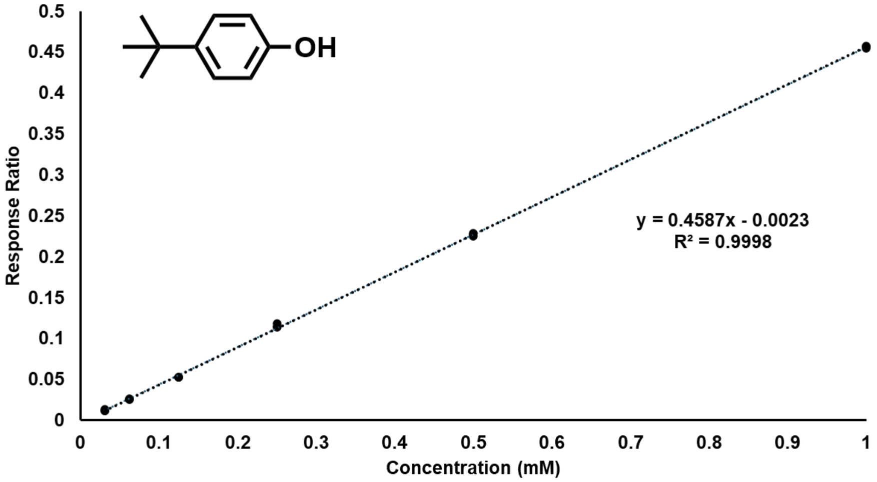

Figure S44. Standard curve for 4-tertbutylphenol **7/12b**. Dinitrotoluene was used as the internal standard and data were collected using absorbance at 280 nm with a C18 column and a mobile phase of MeCN:H_2O containing 0.1% formic acid.

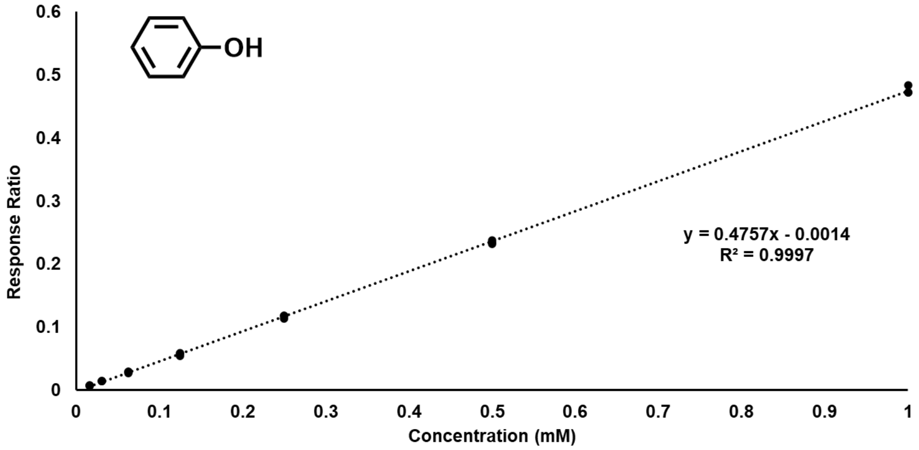

Figure S45. Standard curve for phenol **8b**. 4-nitrophenol was used as the internal standard and data were collected using absorbance at 254 nm with a C18 column and a mobile phase of MeCN:H_2O containing 0.1% formic acid.

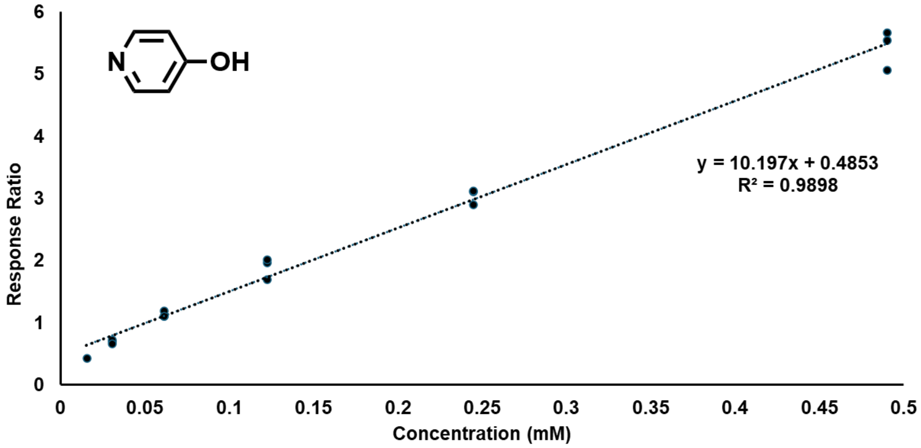

Figure S46. Standard curve for 4-hydroxypyridine **9b**. L-tryptophan was used as the internal standard. Data were collected using absorbance at 254 nm with an Intrada amino acid column (Imtak, USA) and a MeCN:100mM Ammonium formate mobile phase containing 0.1% formic acid.

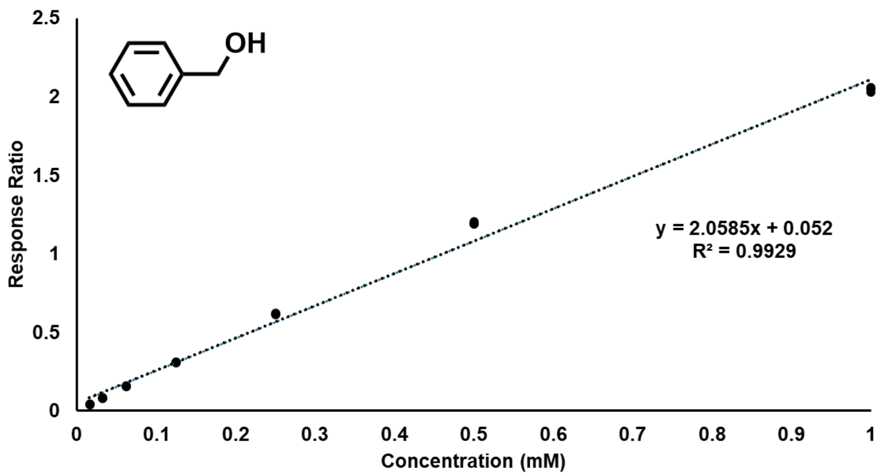

Figure S47. Standard curve for benzyl alcohol **10b**. 4-nitrophenol was used as the internal standard and data were collected using absorbance at 254 nm with a C18 column and a mobile phase of MeCN:H_2O containing 0.1% formic acid.

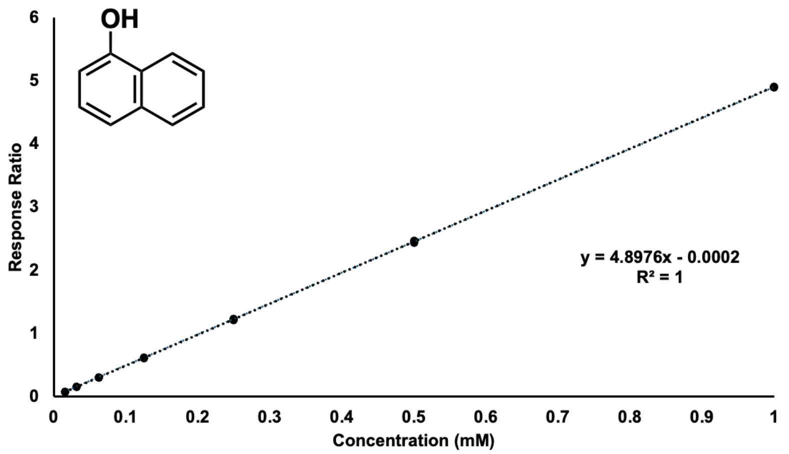

Figure S48. Standard curve for 1-naphthol **13b**. 4-tertbutylphenol was used as the internal standard and data were collected using absorbance at 280 nm with a C18 column and a mobile phase of MeCN:H_2O containing 0.1% formic acid.

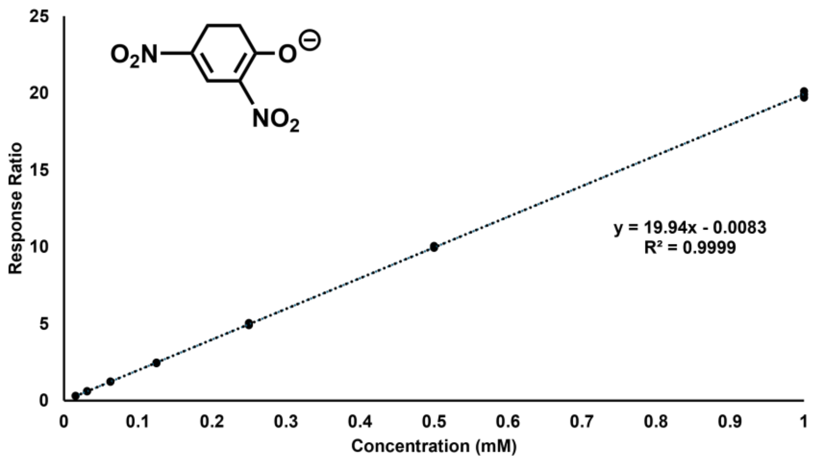

Figure S49. Standard curve for the dearomatized product of dinitroanisole **14b**. 4-tertbutylphenol was used as the internal standard and data were collected using absorbance at 254 nm with a C18 column and a mobile phase of MeCN:H_2O containing 0.1% formic acid.

Kinetic modeling

Kinetic analysis of dearomatization time course using Kintek Explorer

The reaction of CoCYP119 9B12 with DMPS and 2,4-dinitroanisole was monitored by UV-visible spectroscopy (see Figure S18). A volume of 380 µL of 200 mM Potassium phosphate buffer pH = 6.0 was added to a Starna semimicro quartz cuvette, and the instrument was blanked from 250 to 700 nm.

CoCYP119 9B12 (20 µL of a 0-20 µM stock solution) was added the cuvette for a final reaction concentration of 1 µM, 0.35 µM, 0.117 µM, or 0.039 µM for a 500 µL reaction. The cuvette was stoppered and inverted to thoroughly mix. Next, 50 µL of a 1.0 mM stock of 2,4-dinitroanisole (**14a**) in MeCN was added, for a final reaction concentration of 100 µM. The cuvette was once again stoppered, and inverted to mix. A spectrum from 250 to 700 nm was taken. The spectrometer was then set to measure full spectra (250-700 nm) every 3min (180 s) for 2 hours. Finally, 50 µL of a 1.0 mM stock of DMPS was added to initiate the reaction (final [DMPS] = 100 µM). The cuvette was stoppered, and quickly mixed by inversion several times before recording the first spectra at roughly 30 s – 1 min.

Time course spectra data were fit globally using Kintek Explorer software, which leverages Singular Value Decomposition (SVD) analysis and reaction time-course simulation.[6] 3D spectra time courses were uploaded as individual experiments for each enzyme concentration dataset, and five SVD component spectra were used to deconvolute each dataset, such that the amplitude of each SVD component can be plotted with respect to time. The SVD components and their time dependency are plotted in Figure S49.

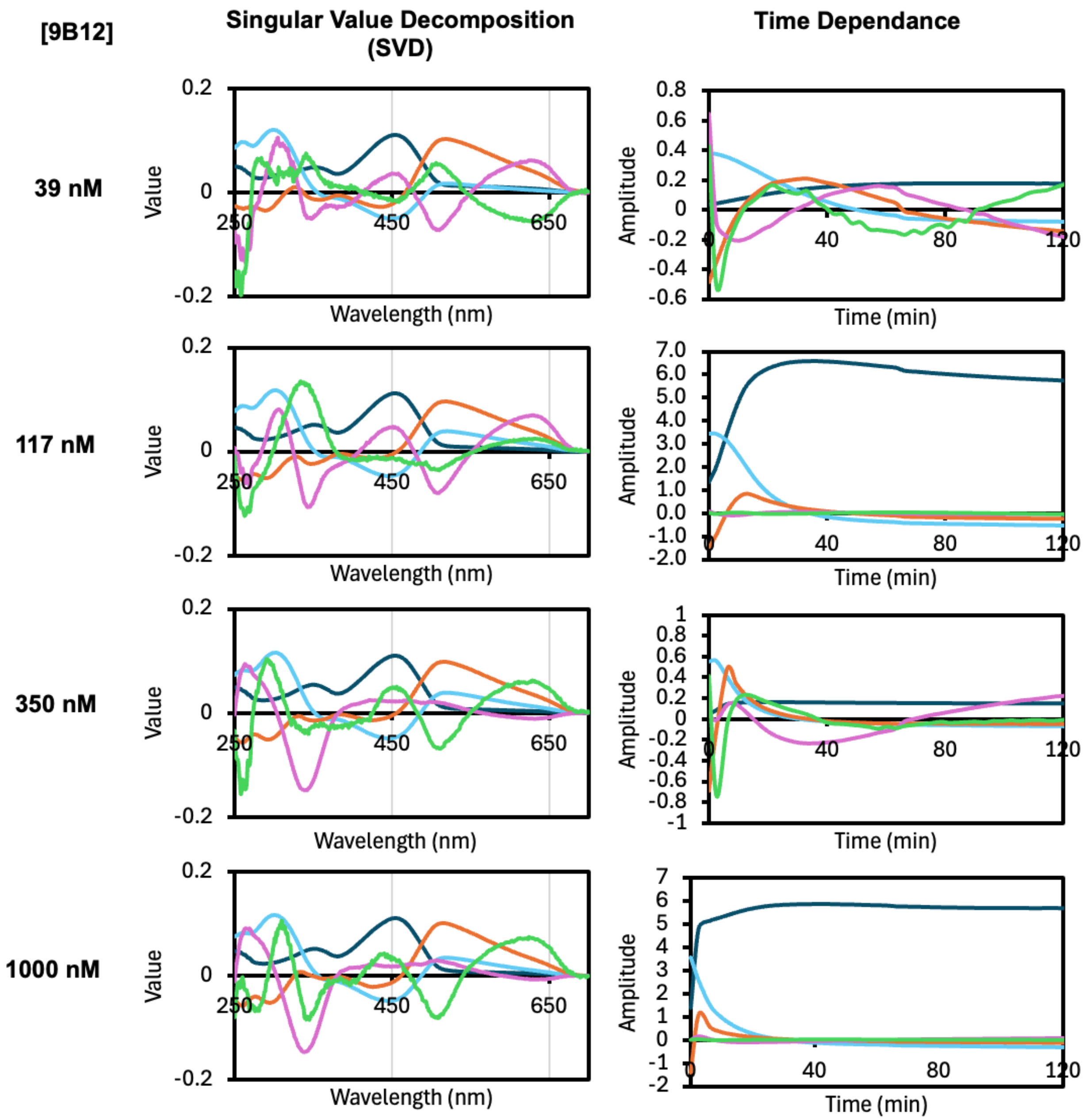

Figure S50. Singular value decomposition (SVD) component spectra (left) and the time dependance of each SVD component for each dataset (right).

The time dependance of these SVD spectra was globally fit to each of the following models, where each experimental observable (Substrate (S), Intermediate (I), and Product (P)) is expressed as a linear combination of the SVD components.

Model A, Two step: E + S = I + E, I = P

Model B, Ping-pong: E + S = I + F, I + F = P + E

Model C, One-step: E + S = P + E

Model D, Branched: E + S = I + E, I = P, E + S = P + E

Graphical representations of these models can be found in panel A of Figures S51-54. The variables P, S, and I were designated as observables. The initial concentrations for I and P were set to zero, and the concentration for E was set to 1, 0.35, 0.117, and 0.039 for each experiment, as appropriate (μM units). The initial concentration of S was set to 100 (μM) for each experiment. The values for k_{-1}, k_{-2}, and k_{-3} (as appropriate) were fixed at zero for irreversible reactions. To ensure that fits converged to a global minimum, a variety of initial conditions were tested for each kinetic parameter. For each model, FitSpace (KinTek Explorer) was used to calculate the codependence of rate constants. While models A and D converged well, parameters for models B and C either did not converge or did not fit the data well (see panels D in Figure S55-58).

The program also generated reconstructed absorbance spectra for each observable (substrate, intermediate, and product) which accounts fits to the model and the observed time course spectra (Panels B, Figure S55-58). For models B and C, sharp discontinuities in these deconvoluted spectra are indicative that the models do not fit the data. Further, comparison of the deconvoluted substrate spectra to the known spectrum of 2-4-dintroanisole starting material (See Figure S19) reveals that only models A and D recapitulate these data well.

Progress curves for each observable are generated by the Kintek Explorer software, and these curves can be compared across the models (Panels I – J, Figures S55-58). The most notable qualitative difference between models is the amplitude of the intermediate observed across

different enzyme concentrations. In the observed absorbance time course data, the maximal absorbance past 550 nm increases as enzyme concentration is increased. Only models A and B are able to recapitulate this effect.

In summary, while both the two step and branched models can describe the data, the additional complexity of the branched model is not necessary to find a good fit. Indeed, the kinetic parameter which described the branched pathway in this mechanism (k_3), is not well-defined. Thus, the two step-irreversible model (model A) is the minimal model needed to accurately describe the data.

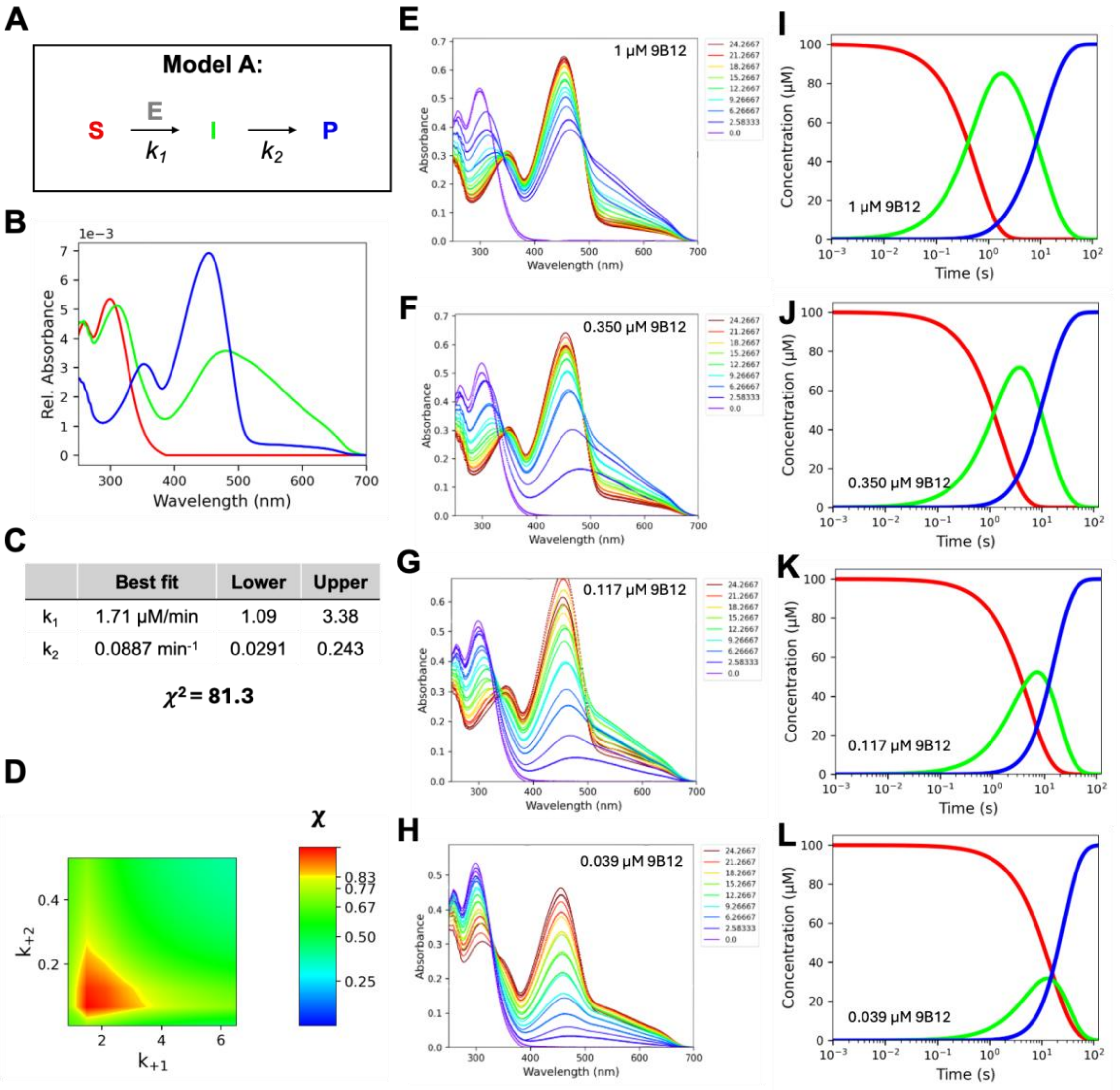

Figure S51. Singular Value Decomposition (SVD) and kinetic fit according to **Model A: two step.** This model qualitatively fits the data very well, and describes the enzyme-dependent increase in the maximal concentration of intermediate that is observed in the data. A) Graphical representation of model A, which is a two-step, irreversible process. B) Deconvoluted component spectra obtained by SVD and kinetic fits according to model A. The red trace represents the deconvoluted spectrum of starting material, the green trace represents the deconvoluted spectrum of the intermediate, and the blue trace represents the product. C) Best fit values for each kinetic parameter, including upper and lower bounds for each value, and the overall Chi-squared value for the fit. D) Contour analysis obtained from FitSpace (Kintek Explorer). Colored areas indicate pairwise values of k_1 and k_2 for which χ^2 is closest to its global minimum, as shown in the key. E-H) Superposition of kinetic time course data (dashed lines) and the result of the best fit simulation data (solid lines) at that time point, for each enzyme concentration. Only the first 30 min of reaction time are shown in this plot, for clarity. I-L) Simulated concentration time courses for the substrate (blue), intermediate (green) and product (blue). These data are plotted on a log time axis, for clarity.

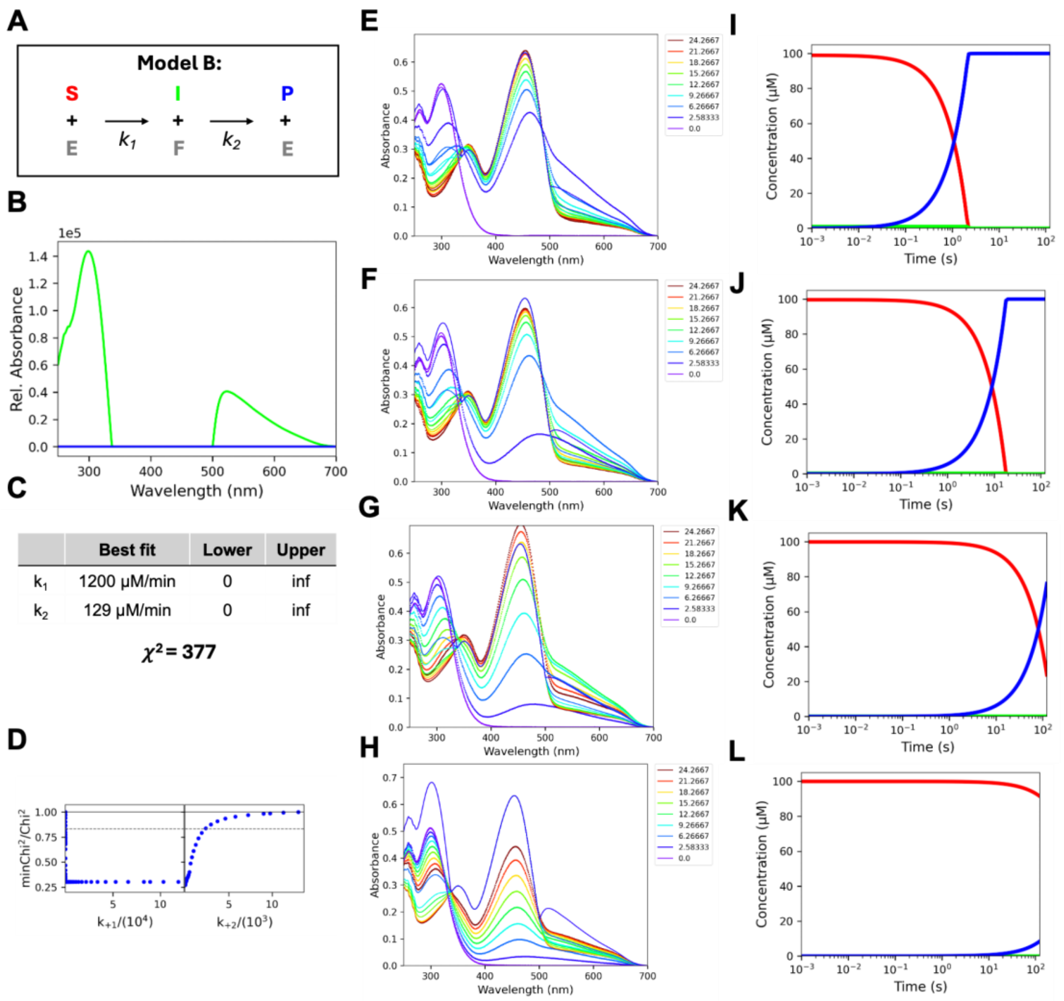

Figure S52. Singular Value Decomposition (SVD) and kinetic fit according to **Model B: Ping-pong.** A) Graphical representation of model B, which is a two-step, irreversible process with a ping-pong mechanism. Because the concentration of intermediate is limited by the concentration of enzyme in this model, the fit is not very good. B) Deconvoluted component spectra obtained by SVD and kinetic fits according to model B. The red trace represents the deconvoluted spectrum of starting material, the green trace represents the deconvoluted spectrum of the intermediate, and the blue trace represents the product. C) Best fit values for each kinetic parameter, which were not well-defined. χ^2 for this fit is also given. D) FitSpace analysis (Kintek Explorer) for the fit of model B. Plots indicate values of k_1 and k_2 for which χ^2 is closest to its global minimum. The dotted line on this plot represents a value for $\min(\chi^2)/\chi^2 = 0.83$, and gives a cut-off for convergence and upper and lower bounds. Because this model does not fit the data well, there is no single values for these parameters for which χ^2 minimized. E-H) Superposition of kinetic time course data (dashed lines) and the result of the best fit simulation data (solid lines) at that time point, for each enzyme concentration. Only the first 30 min of reaction time are shown in this plot, for clarity. I-L) Simulated concentration time courses for the substrate (blue), intermediate (green) and product (blue). These data are plotted on a log time axis, for clarity.

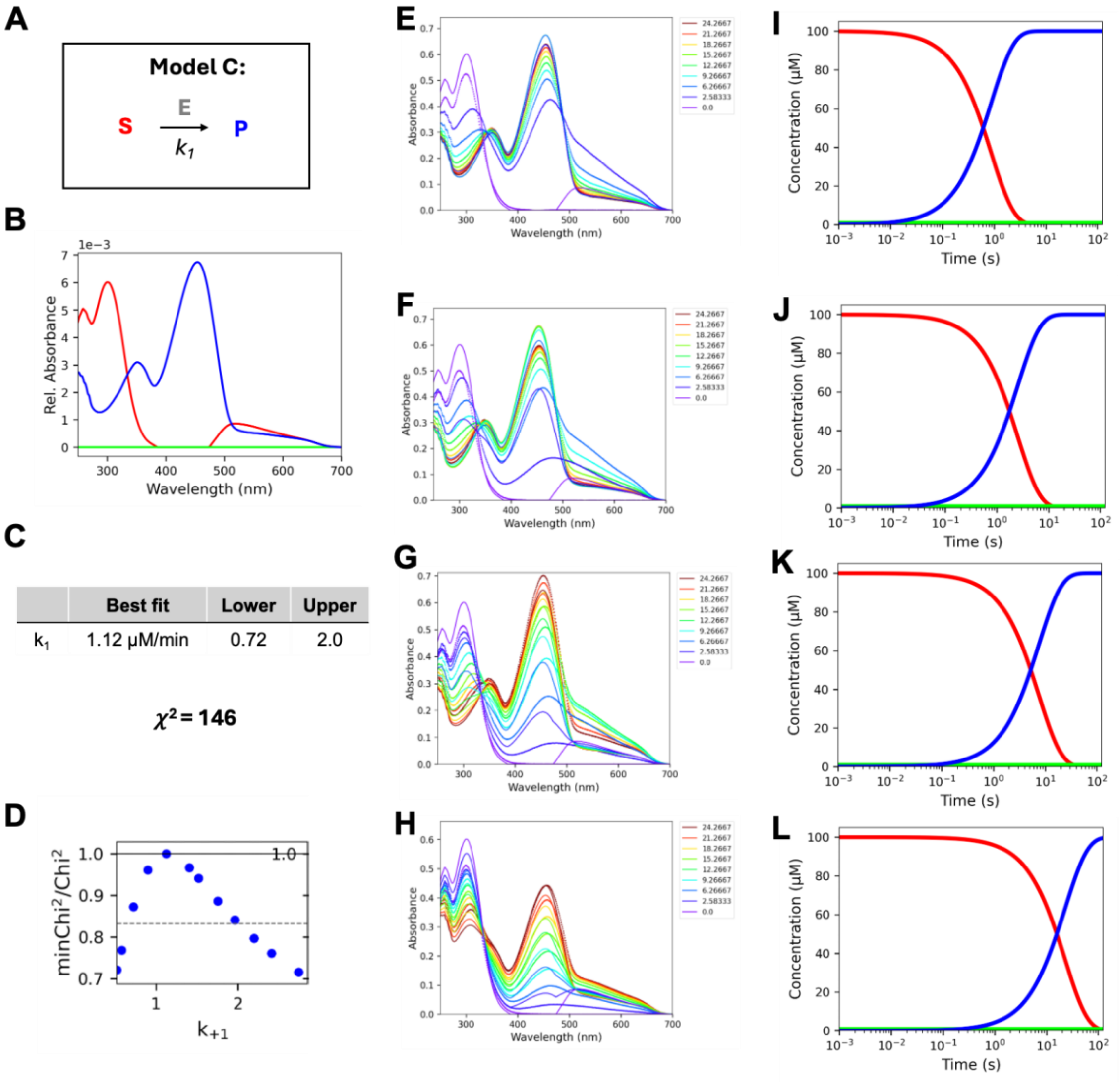

Figure S53. Singular Value Decomposition (SVD) and kinetic fit according to **Model C: One step.** A) Graphical representation of model C, which is a one-step, irreversible process, with no intermediate formed. As indicated by the lack of isosbestic points in the data, a model with at least one intermediate is necessary to describe the data well. B) Deconvoluted component spectra obtained by SVD and kinetic fits according to model B. The red trace represents the deconvoluted spectrum of starting material, the green trace represents the deconvoluted spectrum of the intermediate, and the blue trace represents the product. C) Best fit values for each kinetic parameter, which were not well-defined. χ^2 for this fit is also given. D) FitSpace analysis (Kintek Explorer) for the fit of model C. Plots indicate values of k_1 and k_2 for which χ^2 is closest to its global minimum. The dotted line on this plot represents a value for $\min(\chi^2)/\chi^2 = 0.83$, and gives a cut-off for convergence and upper and lower bounds. E-H) Superposition of kinetic time course data (dashed lines) and the result of the best fit simulation data (solid lines) at that time point, for each enzyme concentration. Only the first 30 min of reaction time are shown in this plot, for clarity. I-L) Simulated concentration time courses for the substrate (blue), intermediate (green) and product (blue). These data are plotted on a log time axis, for clarity.

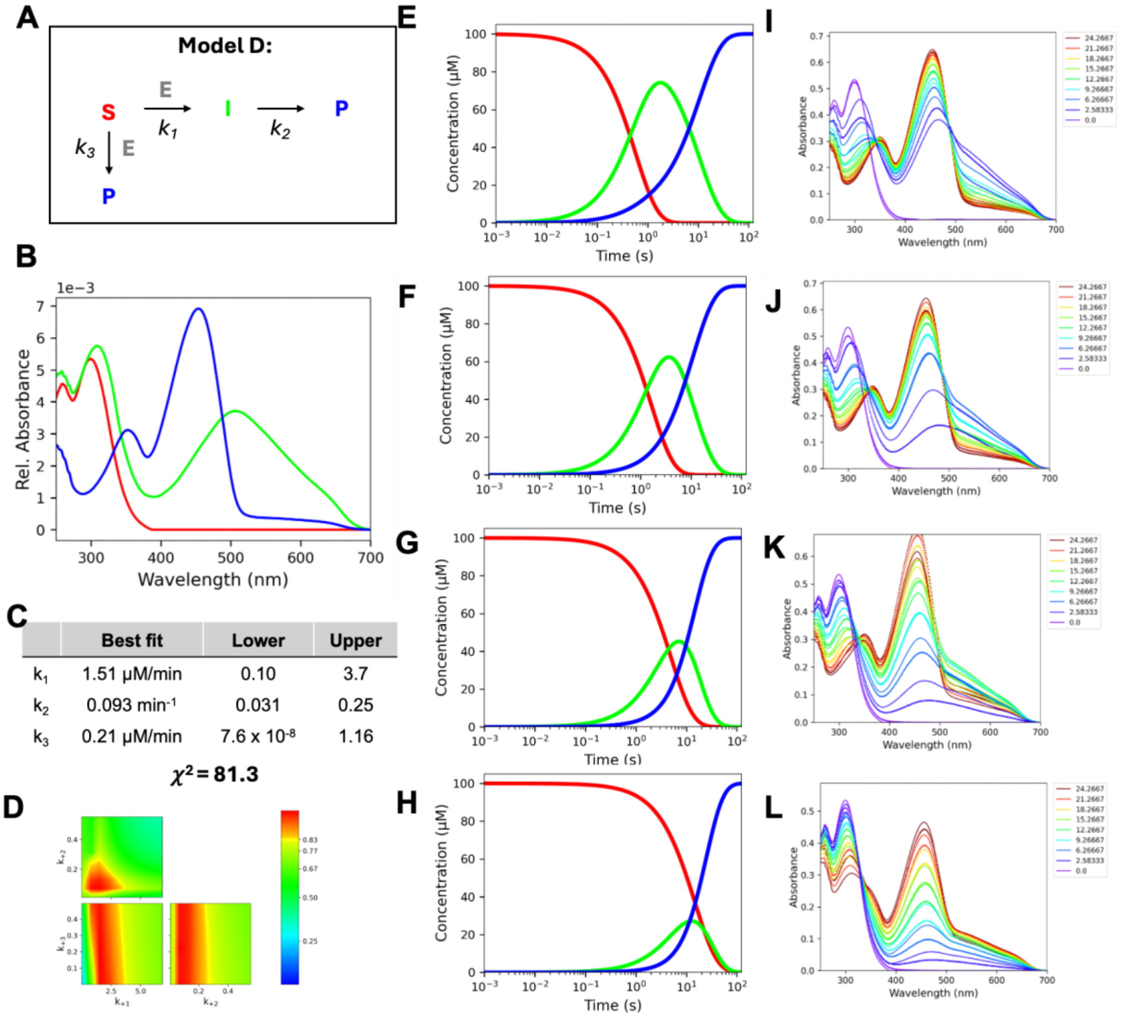

Figure S54. Singular Value Decomposition (SVD) and kinetic fit according to **Model D: branched.** This model qualitatively fits the data very well, and describes the enzyme-dependent increase in the maximal concentration of intermediate that is observed in the data. In this model, product can be formed through two distinct, enzyme-mediated pathways. A) Graphical representation of model D, which is a branched mechanism with a two-step, irreversible process, and one-step irreversible process. B) Deconvoluted component spectra obtained by SVD and kinetic fits according to model D. The red trace represents the deconvoluted spectrum of starting material, the green trace represents the deconvoluted spectrum of the intermediate, and the blue trace represents the product. C) Best fit values for each kinetic parameter, including upper and lower bounds for each value, and the overall Chi-squared value for the fit. D) Contour analysis obtained from FitSpace (Kintek Explorer). Colored areas indicate pairwise values of k_1, k_2, and k_3 for which χ^2 is closest to its global minimum, as shown in the key. While k_1 and k_2 are well-defined in this model, the value of k_3 is not well-defined, indicating that this step may not be necessary to describe the data. E-H) Superposition of kinetic time course data (dashed lines) and the result of the best fit simulation data (solid lines) at that time point, for each enzyme concentration. Only the first 30 min of reaction time are shown in this plot, for clarity. I-L) Simulated concentration time courses for the substrate (blue), intermediate (green) and product (blue). These data are plotted on a log time axis, for clarity.

Reaction optimizations

Dearomatization Reaction Optimization: Anaerobic vs Aerobic Reaction Conditions

To explore the effect of oxygen on the dearomatization reaction, a series of experiments were conducted using an Mbraun glovebox. A comparative analysis was performed, in which standard reaction conditions involved solvents, catalyst, and reagents prepared under ambient or anaerobic conditions. For the anaerobic reactions, 100 mM KPi pH = 6.0 and MeCN were subjected to three cycles of freeze-pump-thaw in a Schlenk flask before being transferred to the nitrogen glovebox. The catalyst (CoCYP119 9B12) in 100 mM KPi pH = 6.0, flash-frozen in liquid N_2, was added to a Schlenk flask, immediately cooled to -78°C, and subjected to three cycles of sustained high vacuum, thawing under a nitrogen atmosphere. The headspace was gently exchanged three times before the catalyst was transferred to the inert atmosphere glovebox. An aliquot of DMPS was also briefly purged with N_2 before being added to the glovebox. Dinitroanisole was added to the glove box as a solid.

Two reactions were set up in parallel according to a modified procedure for the dearomatization of nitro anisole derivatives, which can be found below. Specifically, 5 equivalents of DMPS was used and the reactions were conducted at 20°C on a 500 µL scale, either inside the inert atmosphere glovebox *or* under ambient conditions. Following the addition of the silane, a color change to brown was observed under both anaerobic and ambient conditions. The reactions proceeded over 18 hours, either inside the glovebox *or* on the benchtop, before being quenched with MeCN. The anaerobic reaction was then subsequently removed from the glovebox, and both analyzed by LC-MS.

Table S6. Comparative analysis of the effect of oxygen on the dearomatization reaction, showing the ratio of product **14b** to substrate (2,4-dinitroanisole, **14a**) absorbances at 250 nm. Apparent yield is calculated as: area of product / (area of product + area of substrate).

Reaction Condition:	Peak areas		Apparent yield	Fold conversion
	2,4-dintroanisole 14a	Dearomatized product 14b	Area prod. / (Area SM + Area Prod)	(Rel to std conditions)
Ambient	670,000	610,000	0.48	-
Anaerobic	630,000	630,000	0.50	1.06

Dearomatization Reaction Optimization: Thiol Additive Screen

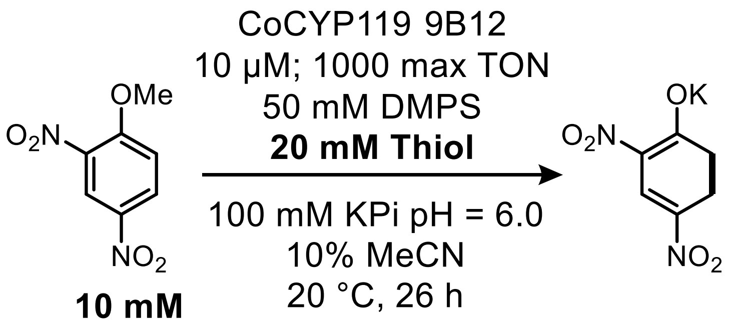

Thiols were evaluated as potential additives in the reaction, aiming to serve as hydrogen atom donors and potentially facilitate a dual catalytic mechanism, as reported by Holland, Miller, and West.[7] Reactions incorporating 2.0 equivalents a thiol additive were conducted in parallel using a modified version of the procedure for the dearomatization of nitro anisole derivatives. Specifically, 5 equivalents of DMPS were employed, and the reaction was carried out at 20 °C with 500 μL total reaction volume. Thiols were introduced as solutions in MeCN prior to the addition of the enzyme. Upon subsequent addition of the neat silane, a characteristic color change to brown was observed. The reactions were allowed to proceed on the benchtop for 26 hours, after which they were quenched with MeCN. The performance of the reactions was analyzed by LC-MS, with conversion compared to a control reaction lacking any additive. The results indicated that thiols had a deleterious effect on the reaction.

Table S7. List of thiols screened in the dearomatization reaction and analysis of the corresponding ratio of product **14b** to substrate (dinitroanisole, **14a**) absorbances at 250 nm, expressed as apparent yield: area of product / (area of product + area of substrate).

Reaction Condition:	Peak areas: 2,4-dintroanisole 14a	Peak areas: Dearomatized product 14b	Apparent yield Area prod. / (Area SM + Area Prod)	Fold conversion (Rel to std conditions)
No additive (control)	190,000	9,000	0.32	1
Thiophenol	330,000	70,000	0.18	0.56
Mercaptocyclopentane	290,000	74,000	0.20	0.63
BOC-Aminoethanethiol	330,000	71,000	0.18	0.56
N-Acetyl cysteamine	210,000	78,000	0.27	0.84
2-Phenylethylmercaptan	260,000	75,000	0.22	0.69

Dearomatization Reaction Optimization: Oxidant Additive Screen

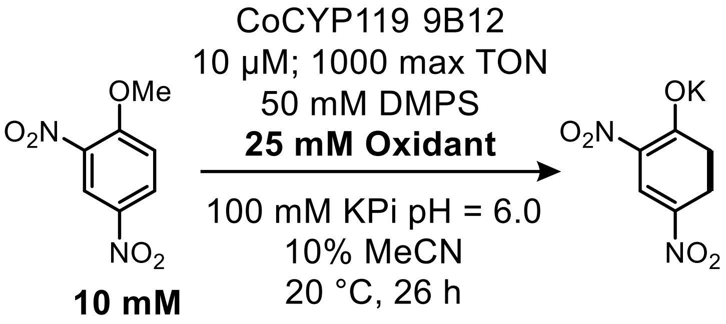

Oxidants were evaluated as additives in the reaction, hypothesized to function as terminal single-electron oxidants in a proposed Co(II/III) M-HAT cycle, as well-defined by Holland.[8] Reactions incorporating 2.5 equivalents of an oxidant were conducted in parallel using a modified procedure for the dearomatization of nitro anisole derivatives. Specifically, 5 equivalents of DMPS were used, and the reaction was carried out at 20 °C in 500 μL of solvent. Oxidants were introduced either neat or in solutions of MeCN or water, depending on their solubility, prior to the addition of the enzyme. Upon the subsequent addition of the neat silane, a characteristic color change to brown was observed in most samples, though the intensity of the color change varied significantly. The reactions were allowed to proceed on the benchtop for 26 hours, after which they were quenched with MeCN. The performance of the reactions was analyzed by LC-MS, with conversion rates

compared to a control reaction without any additive. The results indicated that most oxidants had a deleterious effect on the reaction, with the exception of *tert*-butyl hydroperoxide.

Table S8. List of oxidants screened in the dearomatization reaction and analysis of the corresponding ratio of product **14b** to substrate (**14a**, dinitroanisole) absorbances at 250 nm, expressed as apparent yield: area of product / (area of product + area of substrate).

Reaction Condition:	Peak areas: 2,4-dintroanisole 14a	Peak areas: Dearomatized product 14b	Apparent yield Area prod. / (Area SM + Area Prod)	Fold conversion (Rel to std conditions)
No additive (control)	960,000	380,000	0.28	1
tert-Butyl hydroperoxide	1,200,000	340,000	0.22	0.79
Hydrogen peroxide	1,000,000	46,000	0.04	0.14
Peracetic acid	1,300,000	18,000	0.01	0.04
m-CPBA	1,600,000	2,000	0	0
tert-Butyl hypochlorite	1,200,000	120,000	0.09	0.32
Sodium hypochlorite	1,300,000	0	0	0
Oxone	2,200,000	8,100	0	0
Benzoyl peroxide	2,400,000	0	0	0
tert-Butyl peroxybenzoate	1,700,000	270,000	0.13	0.46
1-Fluoro-2,4,6-trimethylpyridinium tetrafluoroborate	2,000,000	31,000	0.02	0.07

Mechanistic analysis of dearomatization

Deuterium Incorporation Experiments

To investigate the mechanism of the metal hydrogen atom transfer process, a series of deuterium incorporation experiments were conducted using deuterated DMPS or D_2O. When the reduction of 2,4-dinitroanisole was performed using either 1 or 10 equivalents of deuterated silane, both resulted in identical products with full deuterium incorporation at the 5-position carbon, which is meta to the exocyclic oxygen substituent and ortho/para to the electron-withdrawing nitro groups in the starting material. When the reaction was conducted with proto silane, but in buffer containing D_2O, full incorporation of deuterium occurred at the 6-position. The deuteration

reactions were conducted using modified versions of the general procedure for the dearomatization of nitro anisole derivatives, details described below.

D1-DMPS experiment:

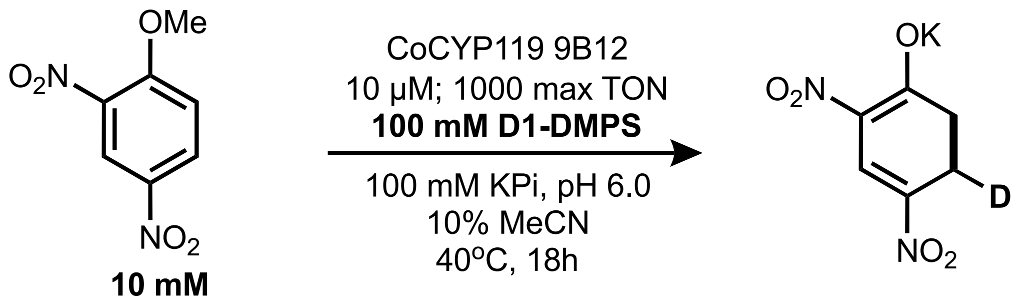

For the reaction with deuterated silane, 10 equivalents of $PhMe_2SiD$ (D1-DMPS) was used, and the reaction was carried out at a concentration of 10 mM **14a** on a 25 mL scale. The resulting deuterated product, (**D5-14b**) potassium 2,4-dinitrocyclohexa-1,3-dien-1-olate-5-d, was analyzed by ^{1}H NMR (see Figure S54).

D_2O experiment:

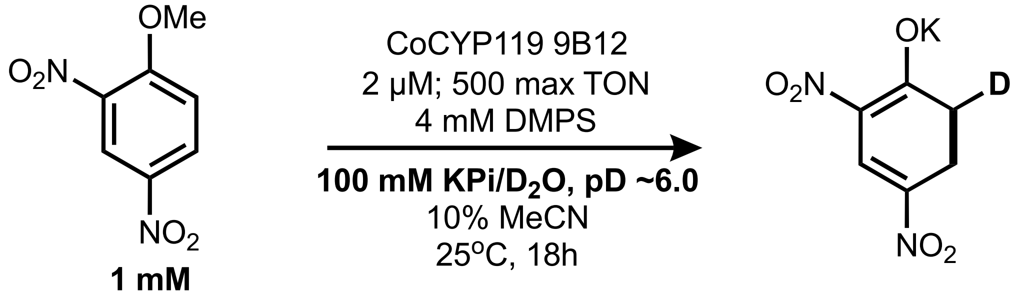

For the experiment in which dearomatization was carried out in D_2O, deuterated potassium phosphate buffer was prepared by lyophilizing 1.0 M potassium phosphate buffer pH = 6.0 and resuspending the resulting salt in D_2O to a concentration of 100 mM. This lyophilization-resuspension process was repeated once more. The dearomatization reaction was carried out at a concentration of 1 mM **14a** at a 50 mL scale, using the isotopically exchanged phosphate buffer. Purified CoCYP119 9B12 was added to a final concentration of 2 μM and the concentration of DMPS was 4 mM (4 equivalents). The resulting deuterated product, (**D6-14b**) potassium 2,4-dinitrocyclohexa-1,3-dien-1-olate-6-d was analyzed by ^1H NMR (see Figure S55).

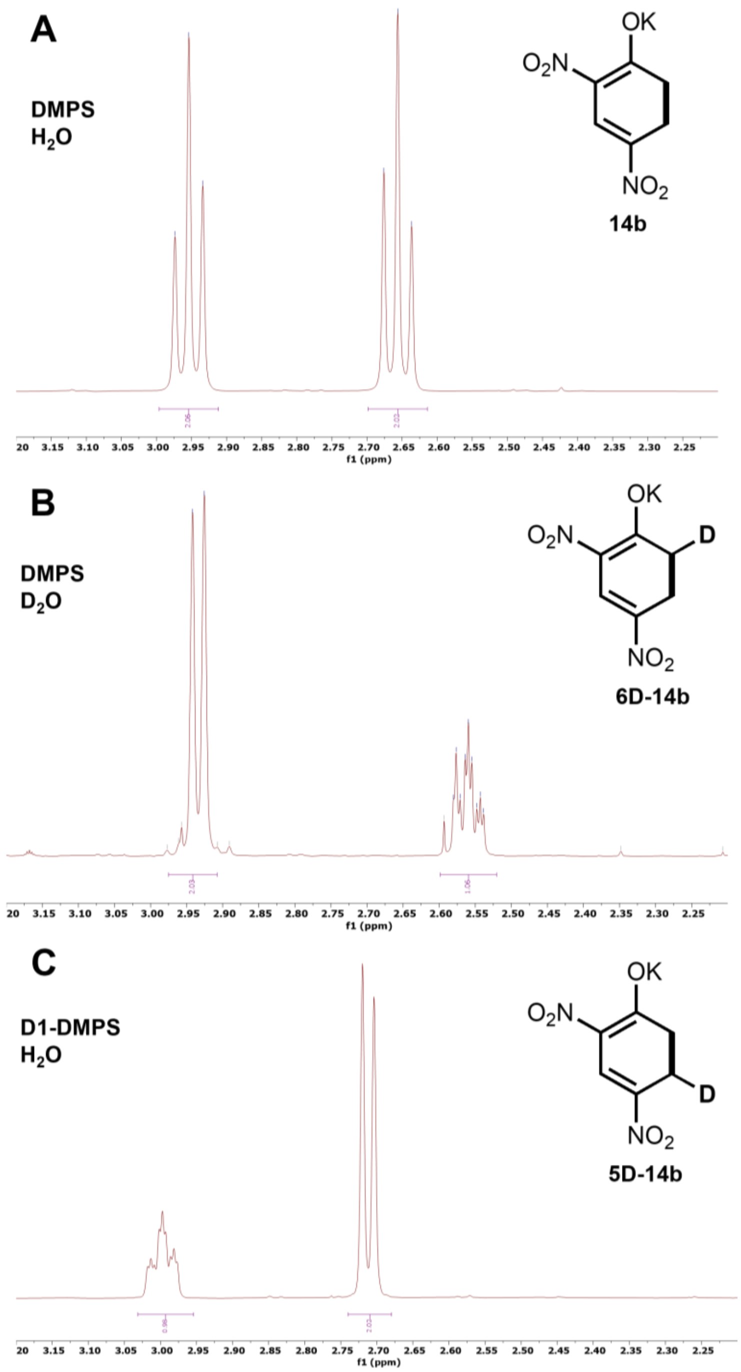

Figure S55. Direct comparison of deuteration patterns for dearomatization of **14a** when D1-DMPS or D_2O are used. Spectra A and C were collected in D_2O. Spectrum B was collected in D4-methanol.

Tempo Trapping Experiment

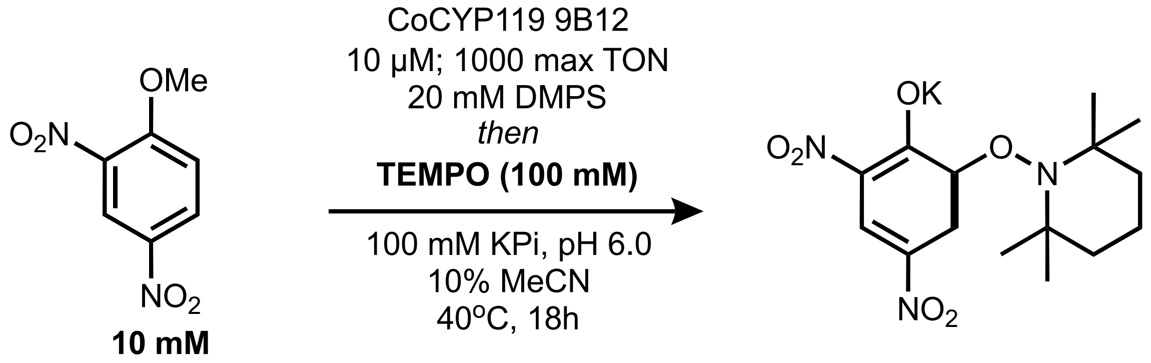

A 125 mL Erlenmeyer flask was loaded with 100 mM potassium phosphate buffer (pH = 6.0, final reaction volume of 10 mL). Heat-treated lysate of CoCYP119 9B12 was thawed and added to the flask (100 μM, 0.1 mol% catalyst, 1000 max TTN). The flask was capped with aluminum foil and placed into an orbital shaker at 40 °C and 200 rpm for 15 minutes.

At 40 °C, nitroarene was added as a solution (1 mL, 0.1 M in MeCN), to achieve a final concentration of 10 mM, 10% v/v MeCN. Then, neat dimethylphenylsilane was added dropwise to initiate the reaction (0.200 mmol, 20 mM final concentration). The reaction vessel was returned to the orbital shaker until a dark red to brown color change was observed. Then, TEMPO (156 mg, 1.00 mmol in minimal MeCN ~150 uL) was added to the reaction. The reaction vessel was returned to the orbital shaker for 18 hours, then an equal volume of MeOH was added and stirred for 15 minutes prior to centrifugation for 15 minutes at 4000xg. The supernatant was analyzed via UPLC-MS.

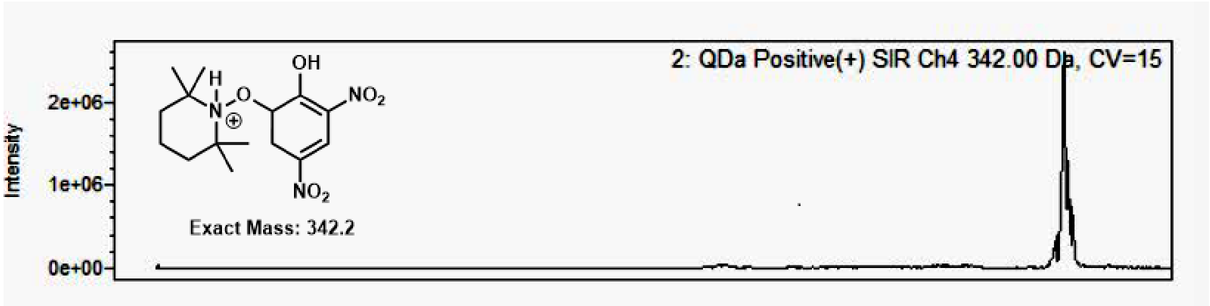

Figure S56. Low-resolution LC-MS analysis of the TEMPO trapping reaction. The adduct $[M+H]^+$ at m/z 342 was observed in positive ion mode using a C18 column with a mobile phase of MeCN:H_2O containing 0.1% formic acid.

Preparative scale enzymatic reactions

General Procedure for dearomatization of nitroanisole derivatives (0.5 mmol scale)

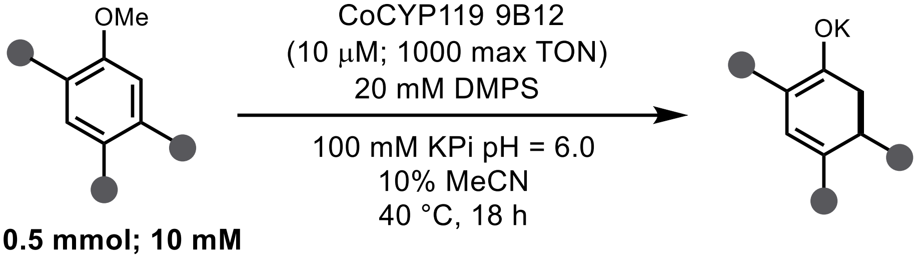

A 125 mL Erlenmeyer flask was loaded with 100 mM potassium phosphate buffer (pH = 6.0, final reaction volume of 50 mL). Heat-treated lysate of CoCYP119 9B12 was thawed and added to the flask (100 µM, 0.1 mol% catalyst, 1000 max TTN). The flask was capped with aluminum foil and placed into an orbital shaker at 40 °C and 200 rpm for 15 minutes.

At 40 °C, nitroarene was added as a solution (5 mL, 0.1 M in MeCN), to achieve a final concentration of 10 mM, 10% v/v MeCN. The slow addition of the nitroarene solution is important to achieve a suspension. Then, neat DMPS was added dropwise to initiate the reaction (1 mmol, 20 mM final concentration). The reaction vessel was returned to the orbital shaker for 18 hours. In most cases, a dark red to brown color change was observed within 20 minutes of silane addition.

The reaction was monitored by UPLC-MS in negative ion mode utilizing C18 column and MeCN:H_2O with 0.1 % formic acid.

After 18 hours, an equal volume of MeOH was added and stirred for 15 minutes prior to centrifugation for 15 minutes at 4000xg. Following decantation, the supernatant was removed in vacuo and the resulting residue taken up in a minimal amount of water (<5 mL) for purification utilizing reverse-phase flash chromatography over C18 with the desired compound eluting at 1-2% MeOH (MeOH:H_2O).

Fractions containing the products were reduced by rotary evaporation, and the residual subjected to lyophilization to afford the dearomatized products, which were characterized by 1D, 2D NMR, and HRMS.

Nitroarene Reductive Dearomatization Reaction Scope

14b: 2,4-dinitrocyclohexa-1,3-dien-1-olate

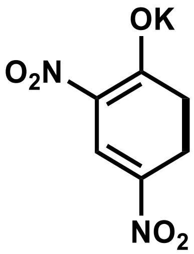

(**14b**) potassium 2,4-dinitrocyclohexa-1,3-dien-1-olate (60 mg, 0.269 mmol, 54%) was prepared according to **General Procedure for dearomatization of nitroanisole derivatives** utilizing the nitroarene 1-methoxy-2,4-dinitrobenzene (**14a**) (99 mg, 0.500 mmol). Analytical data for **14b**: ^{1}H NMR (500 MHz, D_2O) δ 8.75 (s, 1H), 3.01 (t, J = 7.9 Hz, 2H), 2.71 (t, J = 7.9 Hz, 2H). ^{13}C NMR (126 MHz, D_2O) δ 189.8, 134.0, 133.6, 121.4, 36.8, 22.0. HRMS (ESI/Q-IT) m/z: [M-K]- Calcd for C6H5N2O5 185.0204; Found 185.0196.

15b: potassium 5-methyl-2,4-dinitrocyclohexa-1,3-dien-1-olate

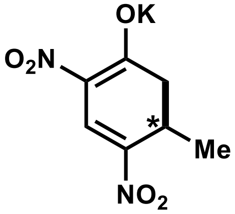

(**15b**) potassium 5-methyl-2,4-dinitrocyclohexa-1,3-dien-1-olate (29 mg, 0.122 mmol, 24%, 48% ee) was prepared according to **General Procedure for dearomatization of nitroanisole derivatives** utilizing the nitroarene 1-methoxy-5-methyl-2,4-dinitrocyclohexa-1,3-diene (**15a**) (107 mg, 0.500 mmol). Analytical data for **15b**: ^{1}H NMR (500 MHz, D_2O) δ 8.53 (s, 1H), 3.27 – 3.10 (m, 1H), 2.80 (dd, J = 16.1, 7.1 Hz, 1H), 2.19 (dd, J = 16.1, 1.8 Hz, 1H), 0.96 (d, J = 7.0 Hz, 3H). ^{13}C NMR (126 MHz, D_2O) δ 189.7, 138.7, 132.4, 121.3, 44.8, 28.7, 17.1. HRMS (ESI/Q-IT) m/z: [M-K]- Calcd for C7H7N2O5 199.0360; Found 199.0353.

Chiral HPLC analysis of DA-2

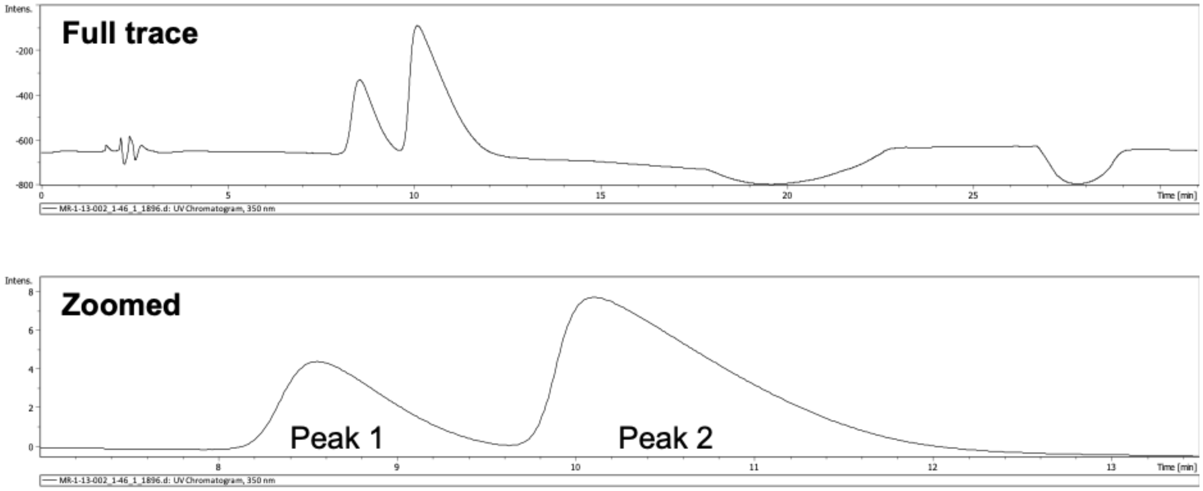

Peak #	RT (min)	Trace	Area
1	8.6	UV chromatogram, 350 nm	169.9
2	10.1	UV chromatogram, 350 nm	483.4
		**e.r.**	26 : 74
		**%ee**	48%

Figure S57. UV absorbance at 350 nm from chiral HPLC analysis of **15b**, used to determine an enantiomeric excess (ee) of 48% for **15b**.

18b: potassium 4-(methoxycarbonyl)-2-nitrocyclohexa-1,3-dien-1-olate

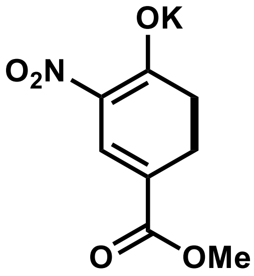

(18b) potassium 4-(methoxycarbonyl)-2-nitrocyclohexa-1,3-dien-1-olate (15 mg, 0.063 mmol, 13%) was prepared according to **General Procedure for dearomatization of nitroanisole derivatives** utilizing the nitroarene methyl 4-methoxy-3-nitrocyclohexa-1,3-diene-1-carboxylate (**18a**) (106 mg, 0.497 mmol). Analytical data for **18b**: ^{1}H NMR (500 MHz, D_2O) δ 8.09 (s, 1H), 3.81 (s, 3H), 2.66 – 2.61 (m, 2H), 2.59 – 2.55 (m, 2H). ^{13}C NMR (126 MHz, D_2O) δ 191.8, 170.0, 133.8, 122.2, 119.1, 52.2, 37.8, 21.8. HRMS (ESI/Q-IT) m/z: [M-K]- Calcd for C6H5N2O5 198.0408; Found 198.401.

17b: potassium 4-cyano-2-nitrocyclohexa-1,3-dien-1-olate

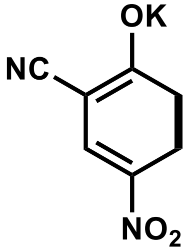

(17b) potassium 4-cyano-2-nitrocyclohexa-1,3-dien-1-olate (44 mg, 0.216 mmol, 60%) was prepared according to a modified (~70% scale) **General Procedure for dearomatization of nitroanisole derivatives** utilizing the nitroarene 4-methoxy-3-nitrocyclohexa-1,3-diene-1-carbonitrile (**17a**) (65 mg, 0.361 mmol). Analytical data for **17b**: ^{1}H NMR (500 MHz, D_2O) δ 8.45 (s, 1H), 3.03 (t, J = 8.1 Hz, 2H), 2.68 (t, J = 8.1 Hz, 3H).^{13}C NMR (126 MHz, D_2O) δ 197.7, 147.1, 124.3, 118.8, 93.6, 34.4, 22.4. HRMS (ESI/ ESI/Q-IT) m/z: [M-K]$^{-}$ Calcd for C7H5N2O3 165.0306; Found 165.0295.

18b: potassium 4-cyano-2-nitrocyclohexa-1,3-dien-1-olate

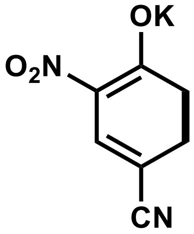

(18b) potassium 4-cyano-2-nitrocyclohexa-1,3-dien-1-olate (52 mg, 0.255 mmol, 51%) was prepared according to a modified **General Procedure for dearomatization of nitroanisole derivatives** utilizing the nitroarene 4-methoxy-3-nitrocyclohexa-1,3-diene-1-carbonitrile (**16a**) (89 mg, 0.500 mmol). Analytical data for **18b**: ^{1}H NMR (500 MHz, D_2O) δ 7.84 (d, J = 1.1 Hz, 0H), 2.71 – 2.43 (m, 1H). ^{13}C NMR (126 MHz, D_2O) δ 189.9, 138.8, 122.2, 121.5, 97.5, 37.1, 24.0. HRMS (ESI/Q-IT) m/z: [M-K]$^{-}$ Calcd for C7H5N2O3 165.0305; Found 165.0295.

Azoxybenzene synthesis: scale up of 1-nitro-4-(trifluoromethyl)benzene reaction to S5

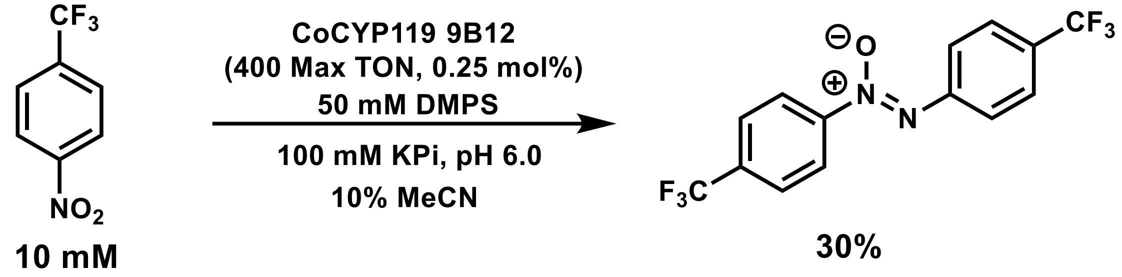

To a 125 mL Erlenmeyer flask containing Co-CYP119 9B12 lysate (12.1 mL, 108 µM, 0.0025 equiv, 1.31 µmol) and 100 mM KPi buffer (pH 6.0, 47 mL) was added 1-nitro-4-(trifluoromethyl)benzene (100 mg, 523 µmol, 1 equiv, 10 mM, 52.3 mL) dissolved in MeCN (4 mL). Dimethyl(phenyl)silane (357 mg, 402 µL, 2.62 mmol, 5 equiv) in MeCN (1.2 mL) was then added, and the reaction mixture was incubated overnight at 40 °C with shaking at 150 rpm for 18 h. The reaction was quenched by adding acetonitrile (50 mL), centrifuged at 4000 × g for 20 min, decanted, and concentrated in vacuo. The residue was purified by silica gel chromatography (gradient 0–5% EtOAc in hexanes). Fractions containing the observed product were combined and concentrated in vacuo to yield (E)-1,2-bis(4-(trifluoromethyl)phenyl)diazene 1-oxide **S5** (26 mg, 78 µmol, 30%) as a crystalline white solid. ^{1}H NMR (500 MHz, $CDCl_3$) δ 8.46 (d, J = 8.5 Hz, 2H), 8.23 (d, J = 8.3 Hz, 2H), 7.82 (d, J = 8.5 Hz, 2H), 7.76 (d, J = 8.4 Hz, 2H). The corresponding spectra agree with known literature values.[9]

Unproductive enzymatic M-HAT to nitroarenes

A variety of nitroarenes were screened for reductive dearomatization, many of which exhibited measurable conversion relative to the no-enzyme control (NEC). However, LC-MS analysis in negative ion mode did not reveal product ions consistent with dearomatization and methyl group loss, as was observed in the case of 2,4-dinitroanisoles, suggesting that for these substrates, hydrolysis of the anisole moiety may not occur. Attempts to scale up and isolate material from these reactions did not yield the desired diene products.

Reactions were performed at 100 μL scale in duplicate. To the enzyme solution (0.25 mol% in 100 mM KPi, pH 6.0; only buffer in NEC), nitroarene substrate (5 μL, 20 mM) was added, followed by DMPS (5 μL, 100 mM) to initiate the reaction. Mixtures were incubated overnight at 40 °C in an orbital shaker (150 rpm) for 18 h. Reactions were then diluted 10-fold with MeCN, centrifuged at 15,000 × g for 10 minutes, and analyzed by LC-MS.

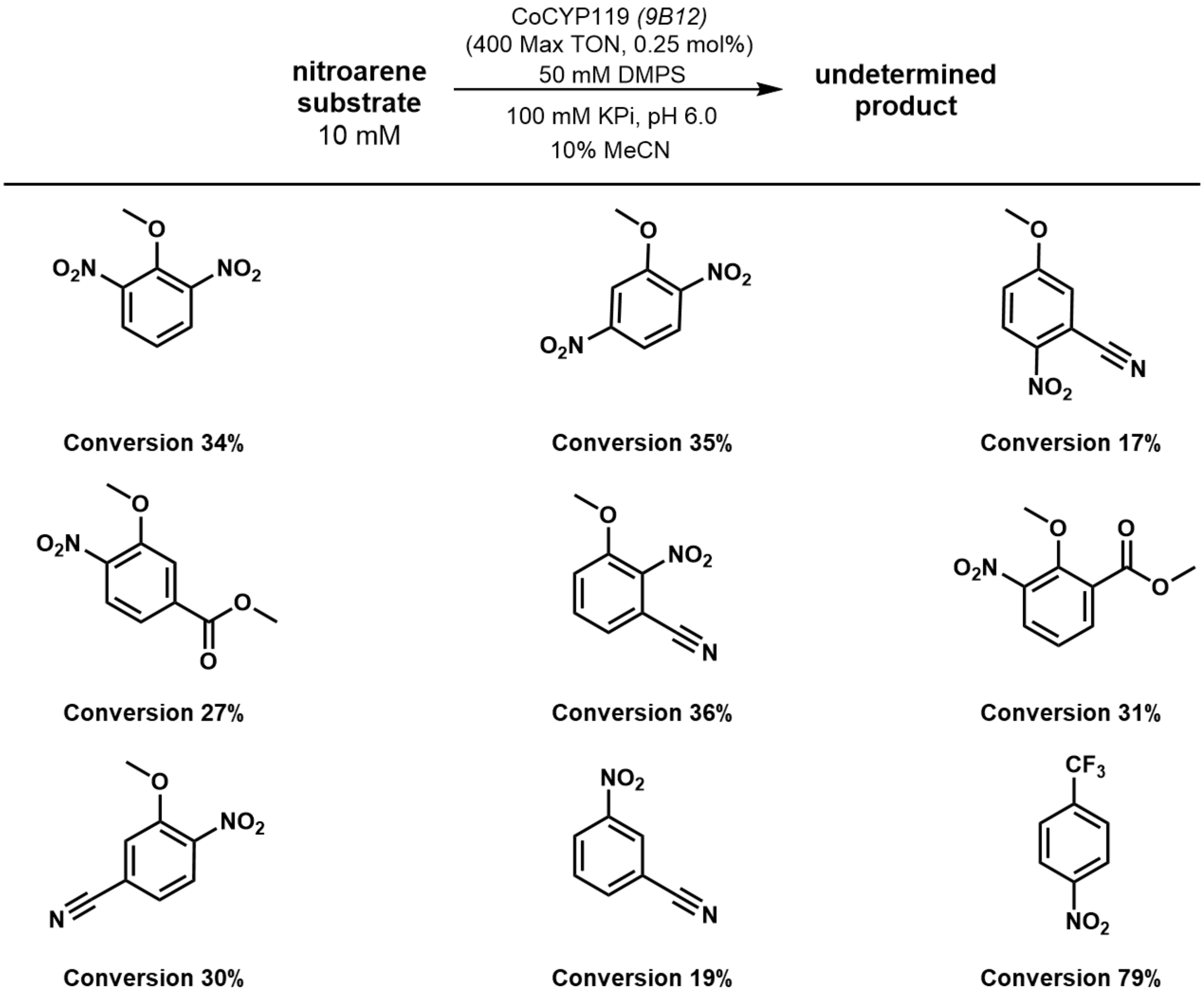

Figure S58. Scope of nitroarenes screened in the reductive dearomatization reaction for which we observed high conversion but were not able to isolate products. Conversion reported relative to a no-enzyme control as determined by LC-MS analysis and detection a 254 nm.

2-methoxy-1,3-dinitrobenzene

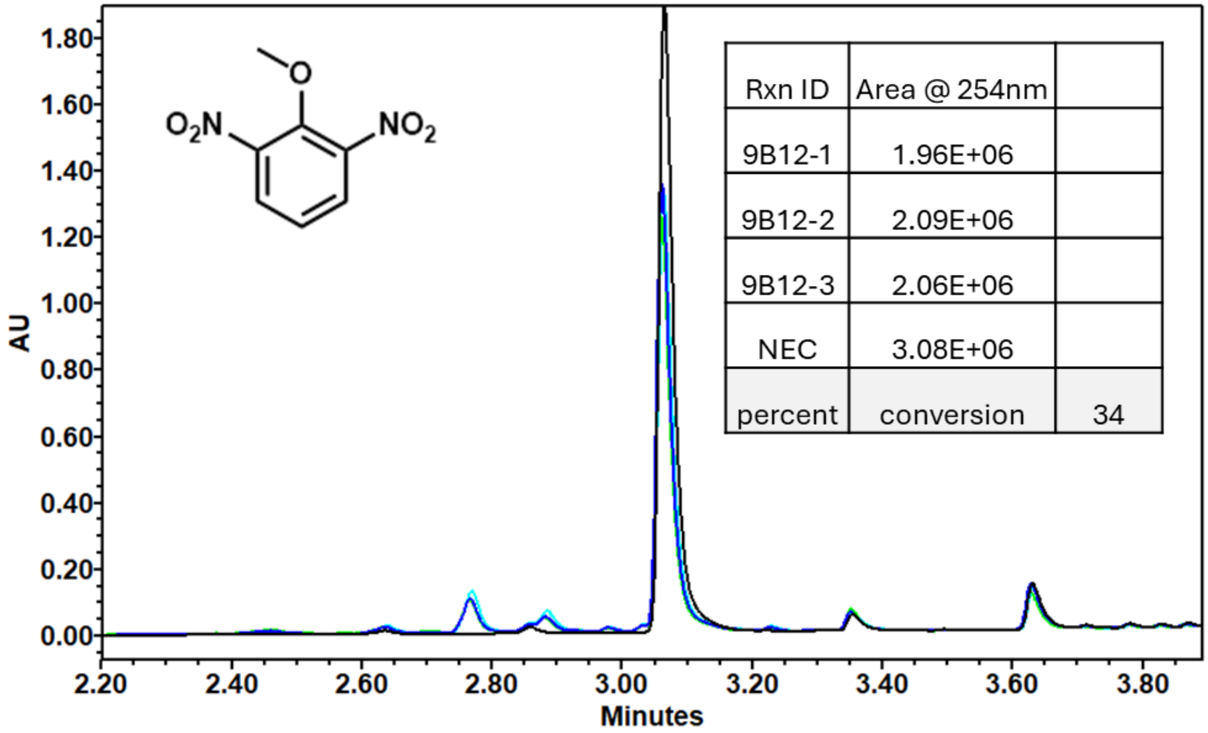

Figure S59. CoCYP119 9B12 (blue, green, and teal traces) compared to a no-enzyme control (black trace) as determined by absorbance at 254 nm, demonstrating consumption of 2-methoxy-1,3-dinitrobenzene. These reactions were carried out with 400 max TON catalyst, 10 mM substrate, 50 mM DMPS, 100 mM KPi (pH 6.0), 10% MeCN, and an 18-hour reaction time at 40 °C with shaking at 150 rpm. In the no-enzyme controls, 100 mM KPi (pH 6.0) was used in place of the cell lysate. Traces represent 10-fold dilutions of these reactions.

2-methoxy-1,4-dinitrobenzene

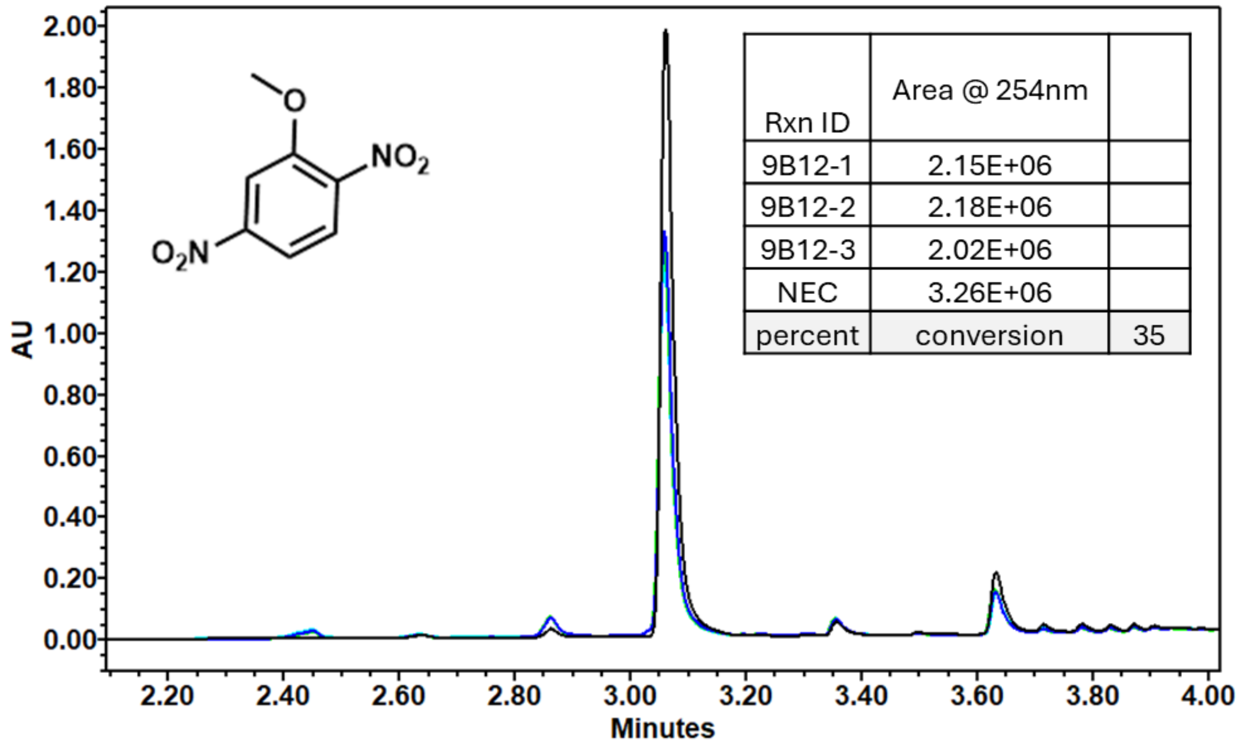

Figure S60. CoCYP119 9B12 (blue and green traces) compared to a no-enzyme control (black trace) as determined by absorbance at 254 nm, demonstrating consumption of 2-methoxy-1,4-dinitrobenzene. These reactions were carried out with 400 max TON catalyst, 10 mM substrate, 50 mM DMPS, 100 mM KPi (pH 6.0), 10% MeCN, and an 18-hour reaction time at 40 °C with shaking at 150 rpm. In the no-enzyme controls, 100 mM KPi (pH 6.0) was used in place of the cell lysate. Traces represent 10-fold dilutions of these reactions.

5-methoxy-2-nitrobenzonitrile

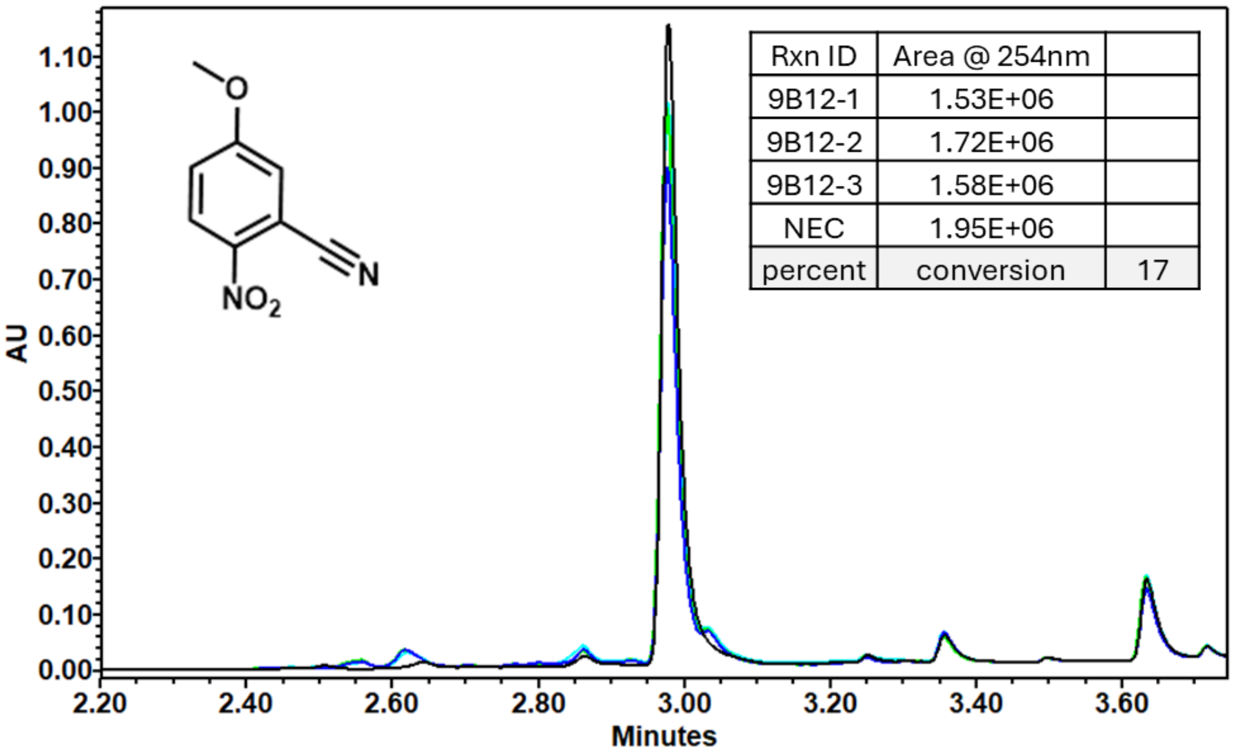

Rxn ID	Area @ 254nm	
9B12-1	1.53E+06	
9B12-2	1.72E+06	
9B12-3	1.58E+06	
NEC	1.95E+06	
percent	conversion	17

Figure S61. CoCYP119 9B12 (blue and green traces) compared to a no-enzyme control (black trace) as determined by absorbance at 254 nm, demonstrating consumption of 5-methoxy-2-nitrobenzonitrile. These reactions were carried out with 400 max TON catalyst, 10 mM substrate, 50 mM DMPS, 100 mM KPi (pH 6.0), 10% MeCN, and an 18-hour reaction time at 40 °C with shaking at 150 rpm. In the no-enzyme controls, 100 mM KPi (pH 6.0) was used in place of the cell lysate. Traces represent 10-fold dilutions of these reactions.

methyl 3-methoxy-4-nitrobenzoate

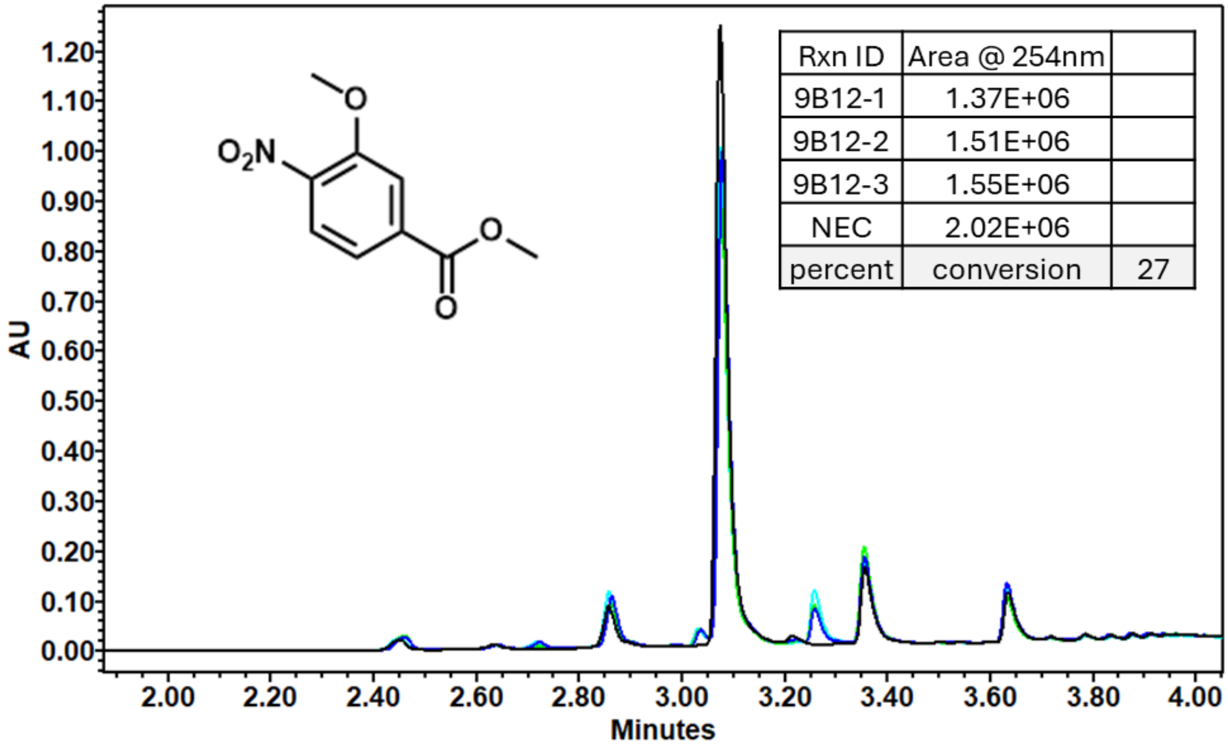

Rxn ID	Area @ 254nm	
9B12-1	1.37E+06	
9B12-2	1.51E+06	
9B12-3	1.55E+06	
NEC	2.02E+06	
percent	conversion	27

Figure S62. CoCYP119 9B12 (blue and green traces) compared to a no-enzyme control (black trace) as determined by absorbance at 254 nm, demonstrating consumption of methyl 3-methoxy-4-nitrobenzoate. These reactions were carried out with 400 max TON catalyst, 10 mM substrate, 50 mM DMPS, 100 mM KPi (pH 6.0), 10% MeCN, and an 18-hour reaction time at 40 °C with shaking at 150 rpm. In the no-enzyme controls, 100 mM KPi (pH 6.0) was used in place of the cell lysate. Traces represent 10-fold dilutions of these reactions.

3-methoxy-2-nitrobenzonitrile

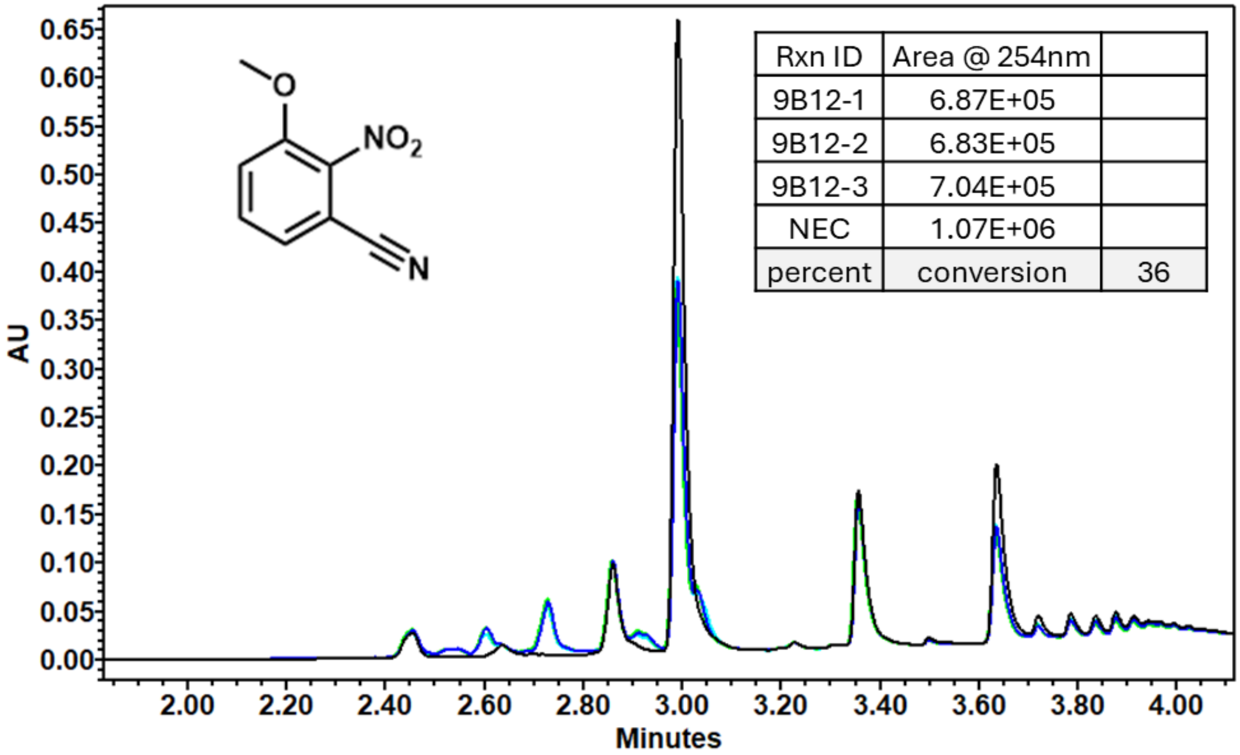

Rxn ID	Area @ 254nm	
9B12-1	6.87E+05	
9B12-2	6.83E+05	
9B12-3	7.04E+05	
NEC	1.07E+06	
percent	conversion	36

Figure S63. CoCYP119 9B12 (blue and green traces) compared to a no-enzyme control (black trace) as determined by absorbance at 254 nm, demonstrating consumption of methyl 3-methoxy-2-nitrobenzonitrile. These reactions were carried out with 400 max TON catalyst, 10 mM substrate, 50 mM DMPS, 100 mM KPi (pH 6.0), 10% MeCN, and an 18-hour reaction time at 40 °C with shaking at 150 rpm. In the no-enzyme controls, 100 mM KPi (pH 6.0) was used in place of the cell lysate. Traces represent 10-fold dilutions of these reactions.

methyl 2-methoxy-3-nitrobenzoate

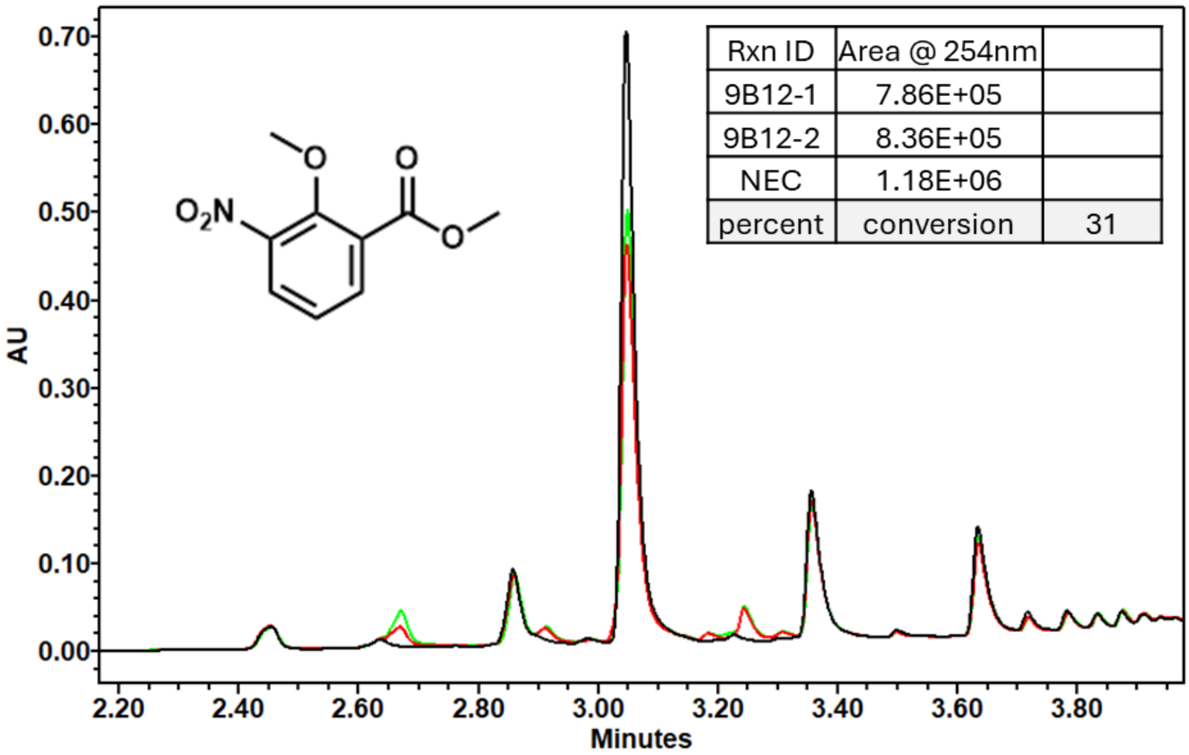

Figure S64. CoCYP119 9B12 (red and green traces) compared to a no-enzyme control (black trace) as determined by absorbance at 254 nm, demonstrating consumption of methyl 3-methoxy-2-nitrobenzonitrile. These reactions were carried out with 400 max TON catalyst, 10 mM substrate, 50 mM DMPS, 100 mM KPi (pH 6.0), 10% MeCN, and an 18-hour reaction time at 40 °C with shaking at 150 rpm. In the no-enzyme controls, 100 mM KPi (pH 6.0) was used in place of the cell lysate. Traces represent 10-fold dilutions of these reactions.

3-methoxy-4-nitrobenzonitrile

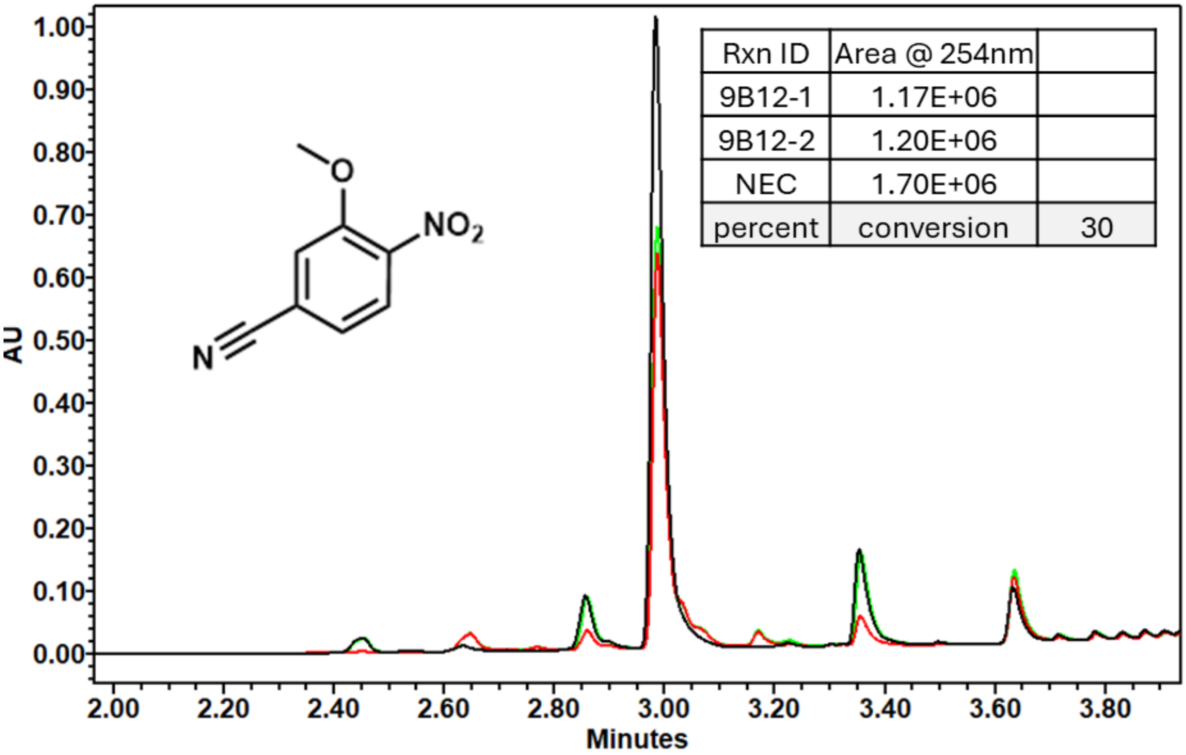

Figure S65. CoCYP119 9B12 (red and green traces) compared to a no-enzyme control (black trace) as determined by absorbance at 254 nm, demonstrating consumption of methyl 3-methoxy-2-nitrobenzonitrile. These reactions were carried out with 400 max TON catalyst, 10 mM substrate, 50 mM DMPS, 100 mM KPi (pH 6.0), 10% MeCN, and an 18-hour reaction time at 40 °C with shaking at 150 rpm. In the no-enzyme controls, 100 mM KPi (pH 6.0) was used in place of the cell lysate. Traces represent 10-fold dilutions of these reactions.

3-nitrobenzonitrile

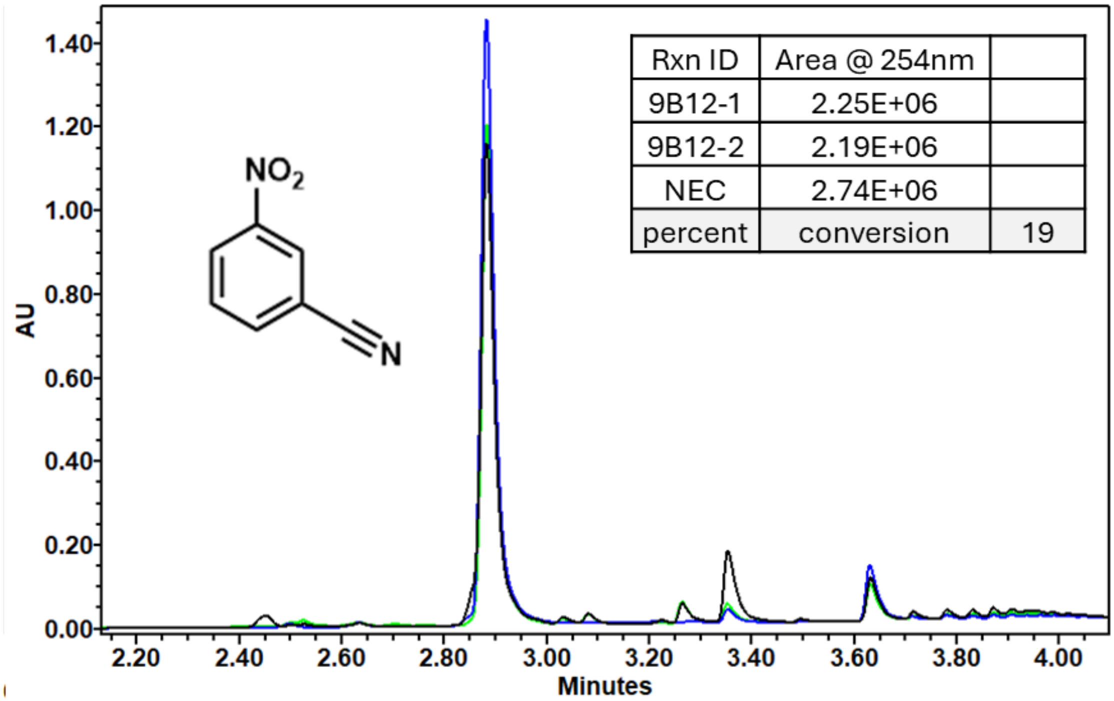

Figure S66. CoCYP119 9B12 (black and green traces) compared to a no-enzyme control (blue trace) as determined by absorbance at 254 nm, demonstrating consumption of 3-nitrobenzonitrile. These reactions were carried out with 400 max TON catalyst, 10 mM substrate, 50 mM DMPS, 100 mM KPi (pH 6.0), 10% MeCN, and an 18-hour reaction time at 40 °C with shaking at 150 rpm. In the no-enzyme controls, 100 mM KPi (pH 6.0) was used in place of the cell lysate. Traces represent 10-fold dilutions of these reactions.

1-nitro-4-(trifluoromethyl)benzene

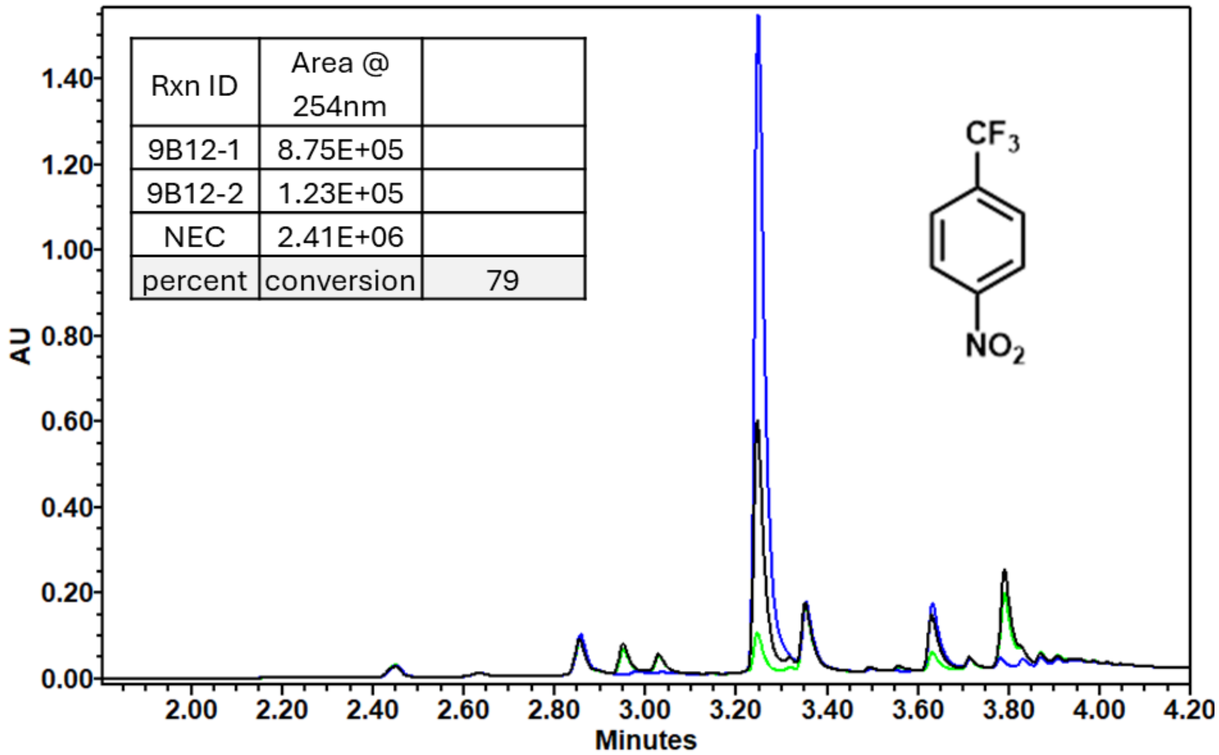

Rxn ID	Area @ 254nm	
9B12-1	8.75E+05	
9B12-2	1.23E+05	
NEC	2.41E+06	
percent	conversion	79

Figure S67. CoCYP119 9B12 (black and green traces) compared to a no-enzyme control (blue trace) as determined by absorbance at 254 nm, demonstrating consumption of 3-nitrobenzonitrile. These reactions were carried out with 400 max TON catalyst, 10 mM substrate, 50 mM DMPS, 100 mM KPi (pH 6.0), 10% MeCN, and an 18-hour reaction time at 40 °C with shaking at 150 rpm. In the no-enzyme controls, 100 mM KPi (pH 6.0) was used in place of the cell lysate. Traces represent 10-fold dilutions of these reactions.

Unsuccessful and Challenging Dearomatization Substrates:

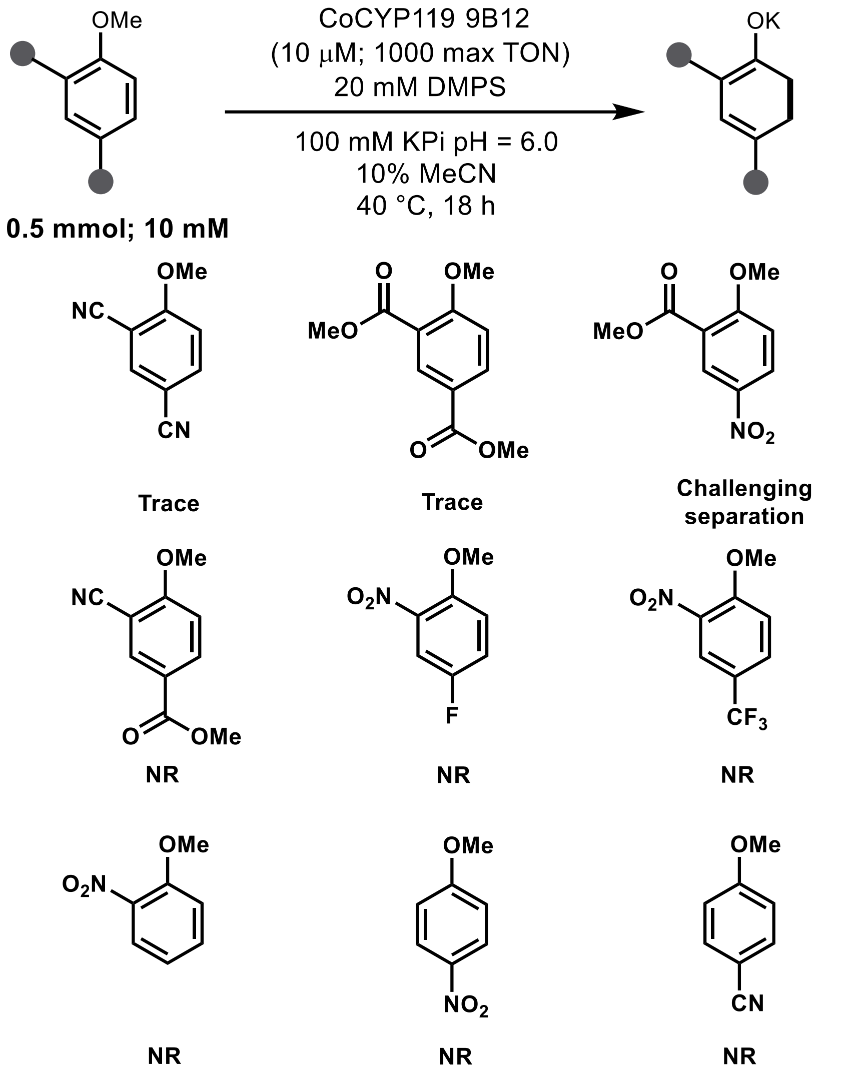

Figure S68. Substrates exhibiting limited or no reactivity in the enzyme-mediated dearomatization reaction. NR indicates no reaction; Trace indicates limited reactivity observed in LC-MS analysis; Challenging separation indicates difficult isolation due to co-elution of impurities.

UV-Visible and EPR spectroscopy of reaction intermediates

Electronic absorption spectra measurements of CoCYP119 and PS adducts

The spectrometer was blanked to a quartz cuvette (Starna) containing 100 mM potassium phosphate buffer pH = 6.0. Spectra of as isolated protein were obtained by adding purified protein solution to this cuvette (final concentration 5-30 μM) and absorbance was measured between 250-700 nm.

Reduced spectra were taken by adding a few crystals of solid sodium dithionite to this cuvette and inverting the cuvette several times to mix. The enzyme sample was heated briefly to 50 °C to accelerate reduction, and absorbance spectra were taken from 250-700 nm.

The cobalt hydride intermediate was captured by adding PS to the enzyme as a solution in DMSO, such that the final concentration of PS was 10 mM, and the solvent was 20% DMSO. The solution was mixed by inverting vigorously several times, and then absorption spectra were taken at ambient temperature, usually every 1-2 minutes over the course of 20 minutes or until the split Soret feature dissipated.

General procedure for EPR characterization of dearomatization radical intermediate:

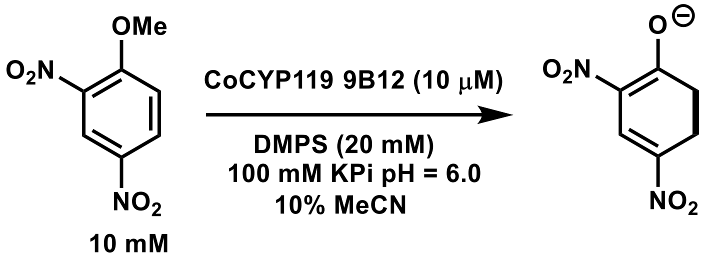

100 mM KPi buffer (pH = 6.0) and MeCN were sparged for 30 minutes with heavy nitrogen flow. To a 1.5 mL microcentrifuge tube 20 μL of CoCYP119 9B1, prepared from supplemented M9 broth (M9*) using an adaptation of the recipe from Maijtan et al.,[10] was added

from a 100 μM stock. 160 μL of sparged 100 mM KPi pH = 6.0 buffer was added to the microcentrifuge tube. An organic master mix consisting of 100 mM dinitroanisole and 200 mM DMPS was prepared in sparged acetonitrile. To initiate the reaction, 20 μL of organic master mix was added to the microcentrifuge tube. The reaction was performed for approximately 30 seconds at room temperature before transferring the contents of the reaction to an EPR tube via syringe. The EPR tube was immediately frozen in liquid nitrogen and was stored under liquid nitrogen temperatures before any spectroscopic data was collected.

X-band EPR data were collected using a Bruker ELEXSYS E500 spectrometer. Sample temperature was maintained at 20 K by an Oxford ESR 900 continuous flow liquid He cryostat regulated by an Oxford ITC-503S temperature controller. All EPR spectra were obtained using the following experimental parameters: frequency = 9.381 GHz; microwave power = 2.000 mW; modulation amplitude = 10.980 G; and modulation frequency = 100 kHz. An EPR spectral fit with an isotropic g value of 2.0023 (characteristic of an organic radical) was performed using the SIMPOW program.

Computational characterization of enzyme and other reaction intermediates

Geometry Optimizations

Initial coordinates for the Co-porphyrin models were obtained from the high-resolution crystal structure the thermophilic cytochrome P450 (CYP119) (PDB 1IO7).[1] Computational models were created by substituting Co for Fe and replacing the carboxylate side chains with methyl groups. All other side chains, including the vinyl groups, were included in the computational models. Ethane thiol and ethane thiolate were used to model protonated and deprotonated axial Cys ligation, respectively. DFT geometry optimizations were performed using the ORCA 4.2.1 program package[11] with the Perdew-Burke-Ernzerhof (PBE) generalized gradient

approximation (GGA) exchange and correlation functional.[12] The def2-TZVP basis set was used for Co and all ligating atoms and the def2-SVP basis set for all other atoms.[13] The Resolution of identity approximation for Coulomb integrals (RI-J) was used with the general def2/J auxiliary basis set.[14] Grid5 integration grid with VeryTightSCF convergence was used for all geometry optimizations. Grimme's D3 dispersion correction was implemented with the "D3" keyword.[15]

Models for the 2,4-dinitroanisole **14a**, three viable reaction intermediates (the radical species Radical and Radical2, as well as the anionic species Anion), and two possible products (the oxyanion Product and carbanion Product2) were generated using Chem3D. DFT geometry optimizations were performed using the ORCA 5.0.4 program package[11] with the Perdew-Burke-Ernzerhof (PBE) generalized gradient approximation (GGA) exchange and correlation functional.[12] The def2-TZVP basis set was used for all atoms.[13] TigthtSCF and TightOPT convergence criteria were used for all geometry optimizations and solvation was accounted for using the conductor-like polarizable continuum model with water as the solvent.

TD-DFT Computed Absorption Spectra

Coordinates from the geometry optimized models were used for subsequent TD-DFT calculations. For the Co-porphyrin models, the CAM-B3LYP functional[16] was used for TD-DFT with the same def2-SVP/def2-TZVP split basis sets as described for the geometry optimizations. Additionally, the RIJCOSX approximation was employed with the def2/J auxiliary basis set for the Coulomb terms, and the def2-SVP/C auxiliary basis set[17] was used for evaluating the Hartree-Fock exchange. The same integration grid, SCF convergence, and dispersion correction were used as described above. Vertical excitation energies were calculated by the TD-DFT method within the Tamm−Dancoff approximation. For each calculation, the 40 lowest-energy excited states within an energy window of ±3 hartrees with respect to the highest occupied molecular orbital

(HOMO)/lowest unoccupied molecular orbital (LUMO) energies were calculated. These data were used to simulate Abs spectra, with each electronic transition assumed to give rise to a Gaussian-shaped band with a full width at half-maximum of 1500 cm^{-1}.

For the models of the 2,4-dinitroanisole **14a**, the reaction intermediates, and product species, TD-DFT computations were also performed with the CAM-B3LYP functional[16] and using def2-TZVP split basis set. The same SCF convergence criteria and solvation model were used as described for the geometry optimizations. Vertical excitation energies were calculated by the TD-DFT method within the Tamm−Dancoff approximation. For each calculation, the 40 lowest-energy excited states within an energy window of ±3 hartrees with respect to the HOMO/LUMO energies were calculated. These data were used to simulate Abs spectra, with each electronic transition assumed to give rise to a Gaussian-shaped band with a full width at half-maximum of 3200 cm^{-1} to match the experimental absorption bandwidths.

Comparison of calculated iron and cobalt-bound hydride species

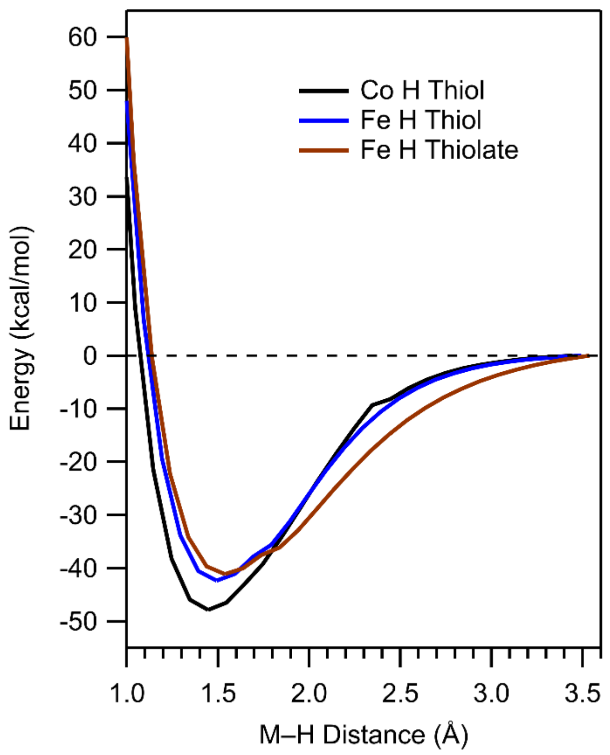

Figure S69. Potential energy curves (PECs) for the of the low spin, metal-hydride thiol(ate) species. For each species, the energies were set to zero after the equilibrium M–H bond distance had been elongated by 2.0 Å.

Metal Hydride Species	M–H Distance (Å)
Co H Thiol	1.45
Fe H Thiol (LS)	1.49
Fe H Thiolate (LS)	1.54
Fe H Thiol (HS)	1.50
Fe H Thiolate (HS)	1.53

High spin (HS) – Low spin (LS) energy difference. (Stabilization of LS vs. HS species)

+ 44.7 kcal/mol Fe H Thiolate

+ 58.2 kcal/mol Fe H Thiol

All geometry optimizations for the Fe-hydride species were performed analogously to that described for the Co hydride thiol species, except that the spin unrestricted Kohn-Sham formalism was necessarily used for the low spin (multiplicity = 2) and high spin (multiplicity = 6) Fe-hydride species.

To construct potential energy curves (PECs), a relaxed potential energy scan was performed by incrementally changing the M–H bond length by 0.1 Å using the "%geom Scan" keyword in ORCA 4.2.1. The same basis sets, functional, grid size, and dispersion corrections described for the geometry optimizations were used for the relaxed potential energy scan. The spin unrestricted Kohn-Sham formalism was used for the Co-hydride species (multiplicity = 1) in addition to the Fe-hydride species as described above. The M–H bond was elongated by 2.0 Å from the original distance observed in the geometry optimized structures. Additionally, the M–H bond was shortened until the M–H bond distance was less than 1.0 Å. The dispersion-corrected SCF energies at each stationary point were plotted as a function of M–H bond distance. The dispersion-corrected SCF energies of the species formed after the M–H bond had been elongated by 2.0 Å were set to zero and the other dispersion-corrected SCF energies were offset accordingly.

Synthesis of substrates

General procedure for preparation of protected dinitro substrates 1-3a

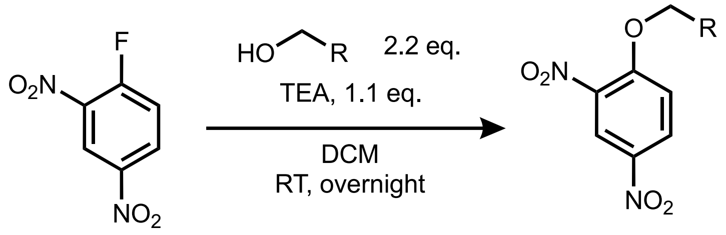

A 100 mL round bottom flask was charged with 1-fluoro-2,4-dinitrobenzene (186 g, 1 mmol), 5 mL of dichloromethane, and a magetic sitr bar. To this solution, allyl or benzyl alcohol (2.2 mmol, 2 eq.) and neat triethylamine (1.1 mmol, 1.1 eq.) were added and the reaction was stirred at room temperature overnight. After 16 h., the solvent was evaporated and the resulting oil was resuspended in ethylacetate. The solution was washed thrice with brine. The organic layer was dried with magnesium sulfate, filtered and the filtrate was evaporated under vaccuum. The resulting residue was purified via silica column chromatography (Ethyl acetate/hexane) using a Biotage flash purification system.

Characterization of 1-3a:

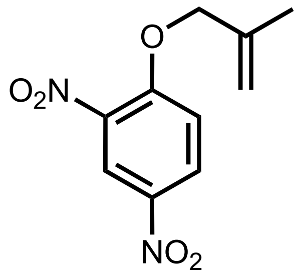

1a (1-((2-methylallyl)oxy)-2,4-dinitrobenzene) was obtained as a yellow oil which solidified to a waxy yellow solid over time, (190 mg, 80% yield). **^{1}H NMR** (500 MHz, $CDCl_3$): δ 8.74 (d, *J* = 2.7 Hz, 1H), 8.40 (dd, *J* = 9.2, 2.8 Hz, 1H), 7.20 (d, *J* = 9.2 Hz, 1H), 5.20 (s, 1H),

5.16 (s, 1H), 4.70 (s, 2H), 1.86 (s, 3H). **^{13}C NMR** (126 MHz, $CDCl_3$): δ 156.5, 140.3, 139.2, 138.3, 129.1, 122.0, 114.9, 114.8, 74.0, 19.2.

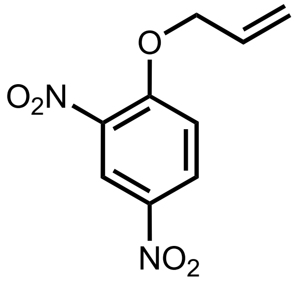

2a (1-(allyloxy)-2,4-dinitrobenzene) was obtained as a yellow oil, which solidified to a waxy yellow solid over time, (913 mg, 83% yield). **^{1}H NMR** (500 MHz, $CDCl_3$): δ 8.78 (d, J = 2.8 Hz, 1H), 8.44 (dd, J = 9.3, 2.8 Hz, 1H), 7.22 (d, J = 9.2 Hz, 1H), 6.06 (ddt, J = 17.3, 10.4, 5.0 Hz, 1H), 5.55 (dq, J = 17.2, 1.6 Hz, 1H), 5.45 (dq, J = 10.7, 1.4 Hz, 1H), 4.85 (dt, J = 5.0, 1.6 Hz, 2H). Spectrum is consistent with information in the literature.[18]

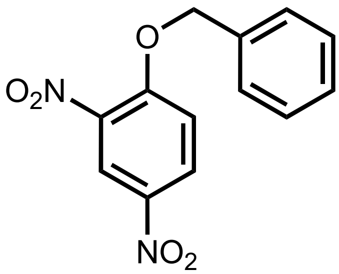

3a (1-(benzyloxy)-2,4-dinitrobenzene) was obtained as a white powder (70 mg, 16% yield). **^{1}H NMR** (500 MHz, $CDCl_3$): δ 8.79 (d, J = 2.8 Hz, 1H), 8.44 (dd, J = 9.3, 2.8 Hz, 1H), 7.42 (m, 5H), 5.4 (s, 2H), 7.26 (d, J = 10 Hz, 1H). Spectrum is consistent with information in the literature.[19]

Preparation of deuterated dimethyl(phenyl)silane:

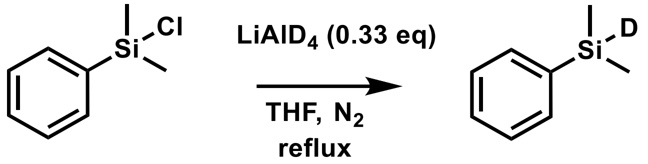

Procedure adapted from known literature procedure[20]: 0.391 g (9.3 mmol, 0.33 eq) of lithium aluminum deuteride (CDN isotopes, 99.6% d4) was added to a 250 mL flame dried round bottom flask containing ~ 50 mL of dry THF under a blanket of N_2. 4.808 g (28.2 mmol) of chlorodimethyl(phenyl)silane was added dropwise to the flask. The reaction was refluxed overnight under N_2. To quench the reaction, the flask was placed on ice, and ~ 100 mL of EtOAc was slowly added. A saturated solution of potassium sodium tetrahydrate in water was added dropwise to the flask (intended to break up emulsion of lithium salts). The contents of the flask were transferred to a separatory funnel and washed three times with 1 M citric acid. The organic layer was dried with $MgSO_4$ and was carefully concentrated in vacuo to avoid evaporating the relatively volatile product until reaching an approximate volume of 10 mL. The contents of the flask were transferred to a distillation flask, and the product was isolated via vacuum distillation (bp = 30˚C, 10 mbar) as a colorless oil (1.441 g, 38% yield, >95% deuterium incorporation).

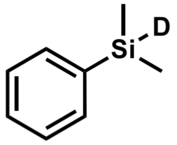

D1-DMPS

Dimethyl(phenyl)silane-*d*: colorless oil, 1.441 g, 38% yield.

^{1}H NMR (500 MHz, $CDCl_3$): δ 7.58 – 7.56 (m, 2H), 7.40 – 7.35 (m, 3H), 0.36 (s, 1H).

^{13}C NMR (126 MHz, $CDCl_3$): δ 137.59, 134.15, 129.33, 128.03, -3.74.

Spectra are consistent with information in the literature.[21]

General procedure for methallylation of phenols:

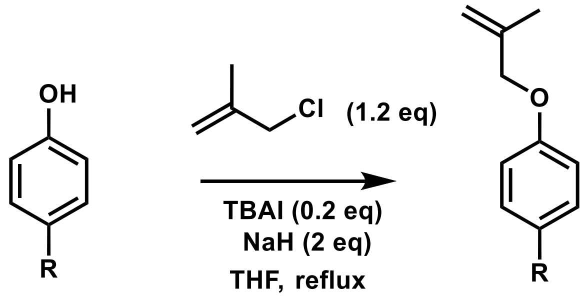

To a flame dried 100 mL round bottom flask was added THF (~ 20 mL) via syringe from a Sure/Seal™ bottle. The flask was subsequently charged with 0.500 g of the phenol derivative and 0.2 equivalents of tetrabutylammonium iodide (TBAI). Methallyl chloride (1.2 equiv) was added via syringe, and the flask was stirred for approximately 1 minute. The flask was placed on ice, and 2 equivalents of NaH (60% NaH in mineral oil) were slowly introduced. The flask was removed from ice and attached to a reflux condenser. The reaction was refluxed until reaction completion was determined via TLC (mobile phase composition generally 20% EtOAc:80% hexanes). After reaching completion, methanol was slowly added to quench remaining NaH. After quenching, residual THF and MeOH were removed in vacuo and the reaction mixture was resuspended in DCM. The reaction mixture was washed three times with 1 M KOH and three times with saturated NH_4Cl. The organic layer was dried with $MgSO_4$ and concentrated in vacuo. To remove residual mineral oil, the crude product was purified via automated silica flash column chromatography, eluting with 100% hexanes.

Characterization of methallylated phenols:

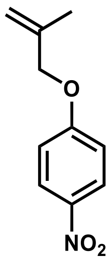

4a

1-((2-methylallyl)oxy)-4-nitrobenzene: Pale yellow oil, 0.769 g, 55% yield.

Deviations from general procedure: Performed on 1 gram scale. Performed reaction with two equivalents of DBU instead of NaH. Added 0.5 equiv of TBAI. After reaction completion and resuspending in DCM, washes were performed with 1 M HCl (3x) and 2 M KOH (3x). Purified over silica with automated flash chromatography (10% EtOAc : 90% hexane).

^{1}H NMR (500 MHz, CDCl$_3$): δ 8.18 (m, 2H), 6.96 (m, 2H), 5.09 (s, 1H), 5.04 (s, 1H), 4.53 (s, 2H), 1.83 (s, 3H).

^{13}C NMR (126 MHz, CDCl$_3$): δ 163.9, 141.7, 139.7, 126.0, 114.9, 113.8, 72.4, 19.4.

Spectra are consistent with information in the literature.[22]

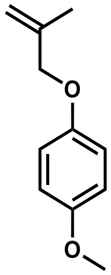

6a

1-methoxy-4-((2-methylallyl)oxy)benzene: White solid, 0.197 g, 28% yield.

Deviations from general procedure: After removing THF in vacuo, the crude reaction mixture was resuspended in hexane. This was washed three times with 1 M KOH and three times with

saturated NH_4Cl. The hexane layer was then washed with acetonitrile in an attempt to remove mineral oil. The acetonitrile layer was dried with $MgSO_4$ and concentrated in vacuo. ^{1}H NMR revealed an impurity at ~ 2.00 ppm (the authors recommend forgoing the wash with acetonitrile and instead perform the general procedure to improve yield). The crude material was purified via automated silica flash chromatography (eluted at 7% EtOAc : 93% hexane), yielding a clear oil that crystalized into a white solid when placed in a 4˚C fridge.

^{1}H NMR (500 MHz, $CDCl_3$): δ 6.86 (m, 4H), 5.08 (s, 1H), 4.98 (s, 1H), 4.39 (s, 2H), 3.77 (s, 3H), 1.83 (s, 3H).

^{13}C NMR (126 MHz, $CDCl_3$): δ 154.0, 153.1, 141.4, 115.9, 114.7, 112.7, 72.6, 55.9, 19.6.

Spectra are consistent with information in the literature.[23]

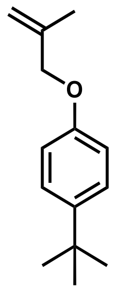

7a

1-(*tert*-butyl)-4-((2-methylallyl)oxy)benzene: Colorless oil, 0.348 g, 52% yield.

Deviations from general procedure: Added 0.14 equiv of TBAI to reaction mixture.

^{1}H NMR (500 MHz, $CDCl_3$): δ 7.31 (m, 1H), 6.88 (m, 1H), 5.11 (s, 1H), 5.00 (s, 1H), 4.43 (s, 1H), 1.85 (s, 3H), 1.32 (s, 9H).

^{13}C NMR (126 MHz, $CDCl_3$): δ 156.7, 143.6, 141.4, 126.3, 114.3, 112.7, 71.9, 34.1, 31.7, 19.6.

Spectra are consistent with information in the literature.[24]

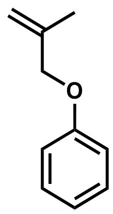

8a

((2-methallyl)oxy)benzene: Colorless oil, 0.372 g, 47% yield.

^{1}H NMR (500 MHz, CDCl$_3$): δ 7.31-7.28 (m, 2H), 6.98-6.94 (m, 3H), 5.12 (s, 1H), 5.01(s, 1H), 4.45 (s, 2H), 1.86 (s, 3H).

^{13}C NMR (126 MHz, CDCl$_3$): δ 158.9, 141.1, 129.5, 120.9, 114.9, 112.8, 71.8, 19.6.

Spectra are consistent with information in the literature.[25]

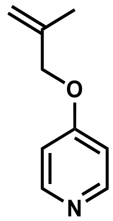

9a

4-((2-methylallyl)oxy)pyridine: White solid, 0.370 g, 62% yield.

Procedure: 0.380 g (4 mmol) of 4-hydroxypyridine was added to a 100 mL round bottom flask. 0.446 mL (4.4 mmol) of methallyl bromide was added via syringe to the flask, followed by 0.828 g (6 mmol) of potassium carbonate. 10 mL of methanol was added to the flask and the contents were stirred. The flask was heated at 50˚C. After four hours, a white precipitate formed, and the contents of the flask were filtered. The filtrate was then purified over C18 with automated flash chromatography, eluting with 20% MeOH : 80% H_2O. The product was lyophilized, yielding a white solid (0.370 g).

^{1}H NMR (500 MHz, CDCl$_3$): δ 7.25 (m, 2H), 6.39 (m, 2H), 5.06 (s, 1H), 4.87 (s, 1H), 4.26 (s, 2H), 1.72 (s, 1H).

^{13}C NMR (126 MHz, CDCl$_3$): δ 179.1, 140.0, 139.6, 118.9, 115.6, 62.6, 19.6.

HRMS (ESI): Calcd for $C_9H_{12}NO^+$ $[M+H]^+$ 150.0913 ; measured 150.0912

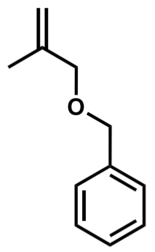

10a

(((2-methylallyl)oxy)methyl)benzene*:* Colorless oil, 0.537 g, 36% yield.

Deviations from general procedure: Reaction performed on 1 gram scale. 1.2 equiv of methallyl bromide used instead of 1.2 equiv of methallyl chloride. A few crystals of TBAI were used as a catalyst (instead of 0.2 equiv). After performing aqueous workup, ^{1}H NMR was collected and mineral oil in the aliphatic region was observed. To remove the residual mineral oil, the crude material was washed with hexane and acetonitrile. The acetonitrile layer was dried with $MgSO_4$ and concentrated to yield pure product.

^{1}H NMR (500 MHz, CDCl$_3$): δ 7.38 – 7.34 (m, 4H), 7.32 – 7.28 (m, 1H), 5.02 (s, 1H), 4.93 (s, 1H), 4.51 (s, 2H), 3.95 (s, 2H), 1.79 (s, 3H).

Spectra consistent with information in the literature.[26]

General procedure for allylation of phenols:

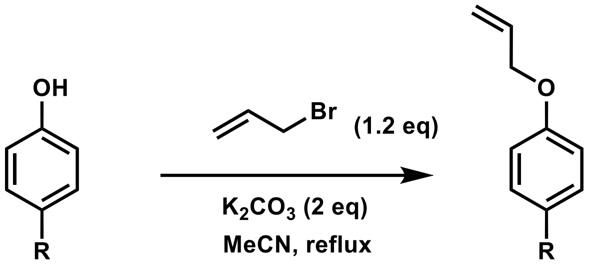

A 100 mL round bottom flask was charged with 0.300 g of the phenol derivative, followed by solid potassium carbonate (2 equiv). 1.2 eq of allyl bromide was introduced via syringe, and 10-20 mL of acetonitrile was added to the flask. The contents of the flask were stirred and refluxed until reaction completion was achieved. The reaction was monitored via TLC using a mobile phase composition of 20% EtOAc : 80% hexanes. After the reaction reached completion, the crude mixture was concentrated in vacuo and resuspended in DCM. This mixture was washed with 1 M HCl (3x) and saturated sodium bicarbonate (3x). The organic layer was dried over $MgSO_4$ and concentrated in vacuo. The crude material was purified with automated silica flash chromatography, with pure material generally eluting at 10% EtOAc : 90% hexanes.

General procedure for synthesis of anisoles:

1 mmol of fluorinated substrate was added to a 100 mL round bottom flask with a stir bar. 10 mL of methanol was added to the flask, and the contents of the flask were stirred. 3 equiv (3 mmol) of triethylamine was added dropwise to the flask. The reaction was refluxed until completion as determined by TLC. **15a**, 1-methoxy-5-methyl-2,4-dinitrobenzene, 98% yield, ^{1}H NMR (400 MHz, $CDCl_3$) δ 8.71 (s, 1H), 6.99 (s, 1H), 4.07 (s, 3H), 2.75 (s, 3H). **19a**, 4-methoxyisophthalonitrile, 32% yield, ^{1}H NMR (400 MHz, CD_3CN) δ 8.02 (d, J = 2.2 Hz, 1H), 7.95 (dd, J = 8.9, 2.2 Hz, 1H), 7.26 (d, J = 8.9 Hz, 1H), 4.00 (s, 3H).

Characterization of allylated phenols:

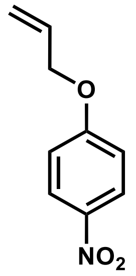

5a

1-(allyloxy)-4-nitrobenzene (**5a**): Yellow oil, 0.305 g, 79%

^{1}H NMR (500 MHz, CDCl$_3$): δ 8.21 – 8.17 (m, 2H), 6.98-6.95 (m, 2H), 6.04 (ddt, *J* = 17.2, 10.5, 5.3 Hz, 1H), 5.43 (dq, *J* = 17.2, 1.5 Hz, 1H), 5.35 (dq, *J* = 10.5, 1.4 Hz, 1H), 4.64 (dt, *J* = 5.3, 1.6 Hz, 1H).

^{13}C NMR (126 MHz, CDCl$_3$): δ 163.7, 141.7, 132.0, 126.0 118.8, 114.8, 69.5.

Spectra are consistent with information in the literature.[27]

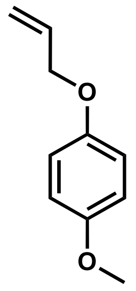

11a

1-(allyloxy)-4-methoxybenzene (**11a**): Pale yellow oil, 0.100 g, 25% yield.

Deviations from general procedure: Heated reaction mixture at 50˚C (authors recommend to instead follow the general procedure and reflux the reaction).

^{1}H NMR (500 MHz, CDCl$_3$): δ 6.86 (m, 4H), 6.05 (ddt, *J* = 17.2, 10.6, 5.3 Hz, 1H), 5.40 (dq, *J* = 17.3, 1.7 Hz, 1H), 5.27 (dq, *J* = 10.4, 1.5 Hz, 1H), 4.49 (dt, *J* = 5.3, 1.5 Hz, 2H), 3.77 (s, 3H).

^{13}C NMR (126 MHz, CDCl$_3$): δ 154.1, 152.9, 133.8, 117.6, 115.9, 114.8, 69.7, 55.9.

Spectra are consistent with information in the literature.[28]

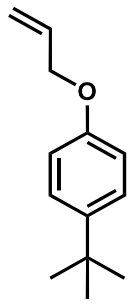

12a

1-(allyloxy)-4-(tert-butyl)benzene (**12a**): clear oil, 0.214 g, 16% yield.

Procedure: 60 mL of DMF was added via syringe to a flame dried 250 mL round bottom flask under N_2. The flask was charged with 1 gram of 4-t-butyl phenol (6.7 mmol) followed by 2 equiv of allyl bromide (13.4 mmol, 1.16 mL). 2.9 eq (0.780 g) of 60% NaH in mineral oil was added to the flask. The reaction was stirred at room temperature for 22 hours under an N_2 atmosphere. After 22 hours, ~ 20 mL of DI H_2O was carefully added to the flask to quench remaining NaH. The crude reaction mixture was concentrated in vacuo to remove DMF. The crude product was resuspended in EtOAc and transferred to a separatory funnel. The organic layer was washed with a LiCl solution to remove residual DMF. The organic layer was subsequently dried with $MgSO_4$. The resulting oil was purified via automated silica flash chromatography, eluting with 10% EtOAc : 90% hexanes (0.214 g, 16% yield).

^{1}H NMR (500 MHz, CDCl$_3$): δ 7.33 – 7.30 (m, 2H), 6.89 – 6.86 (m, 2H), 6.08 (ddt, J = 17.2, 10.6, 5.3 Hz, 1H), 5.42 (dq, *J* = 17.2, 1.6 Hz, 1H), 5.29 (dq, *J* = 10.4, 1.5 Hz, 1H), 4.54 (dt, *J* = 5.3, 1.6 Hz, 1H), 1.32 (s, 1H).

^{13}C NMR (126 MHz, CDCl$_3$): δ 156.5, 143.7, 133.8, 126.3, 117.6, 114.3, 69.0, 34.2, 31.7.

Spectra are consistent with information in the literature.[25]

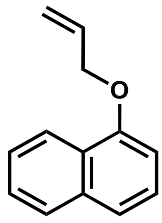

13a

1-(allyloxy)naphthalene (**13a**): Clear oil, 0.295 g, 23% yield.

Procedure: 1 gram (6.94 mmol) of 1-naphthol was added to a 100 mL round bottom flask. 1.2 equiv of allyl bromide (0.720 mL, 8.33 mmol) was introduced into the flask, followed by 2 equiv of potassium carbonate (1.918 g, 13.9 mmol). 30 mL of HPLC grade acetone was added to the flask, and the contents of the flask were stirred and heated at 60˚C. After one hour, reaction progress was monitored with TLC (20% EtOAc : 80% hexanes) and starting material was still present. 1 eq of KI (6.94 mmol, 1.152 g) was subsequently added to the flask to increase the reaction rate. Two hours after addition of KI, the contents of the flask were filtered over celite to remove potassium carbonate, and the filtrate was concentrated in vacuo. The crude product was subsequently purified with silica flash chromatography (eluting at 100% hexanes – 15% EtOAc : 85% hexanes), yielding a clear oil after concentrating in vacuo (0.295 g, 23%).

^{1}H NMR (500 MHz, $CDCl_3$): δ 8.34 (m, 1H), 7.81 (m, 1H), 7.52 - 7.44 (m, 3H), 7.37 (t, J = 7.4 Hz, 1H), 6.83 (d, J = 7.6 Hz, 1H), 6.19 (ddt, J = 17.4, 10.5, 5.2 Hz, 1H), 5.54 (dq, *J* = 17.3, 1.7 Hz, 1H), 5.35 (dq, *J* = 10.8, 1.5 Hz, 1H), 4.74 (dt, *J* = 5.2, 1.6 Hz, 2H).

^{13}C NMR (126 MHz, $CDCl_3$): δ 154.5, 134.7, 133.5, 127.6, 126.5, 125.9, 125.3, 122.2, 120.5, 117.5, 105.2, 69.1.

Spectra are consistent with information in the literature.[29]

NMR Spectra of novel compounds

1a: (1-((2-methylallyl)oxy)-2,4-dinitrobenzene)
^{1}H NMR (500 MHz, $CDCl_3$):

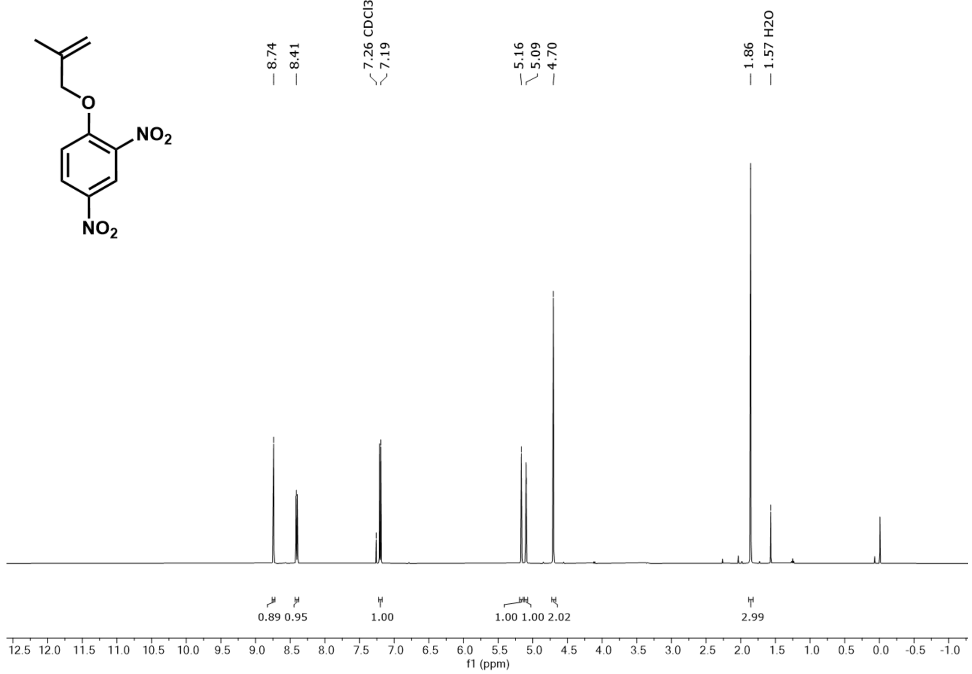

^{13}C NMR (126 MHz, $CDCl_3$):

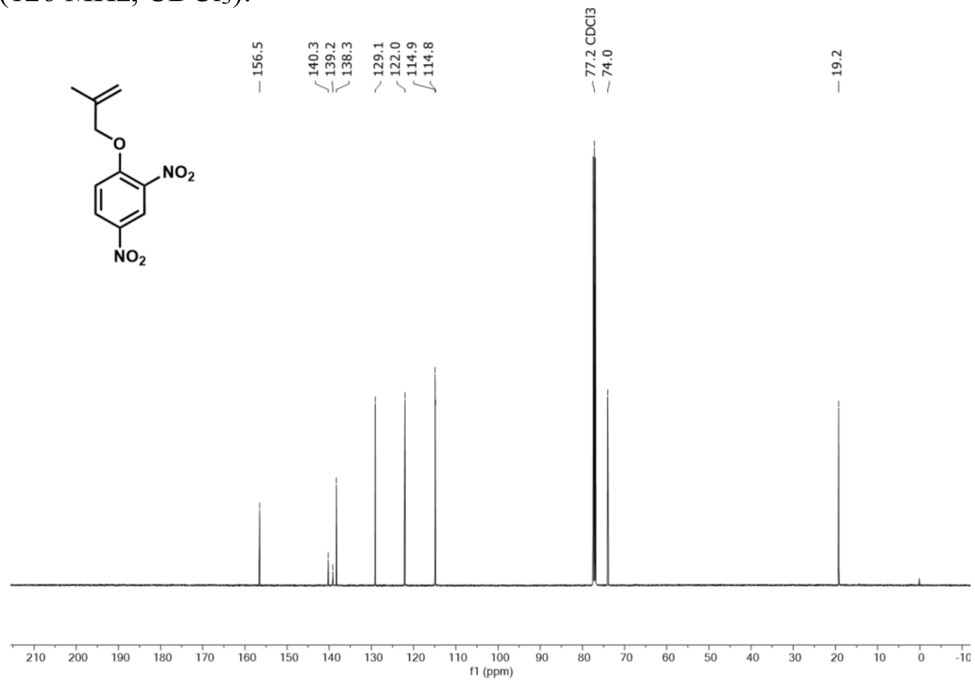

D1-DMPS: Dimethyl(phenyl)silane-*d*

^{1}H NMR (500 MHz, $CDCl_3$):

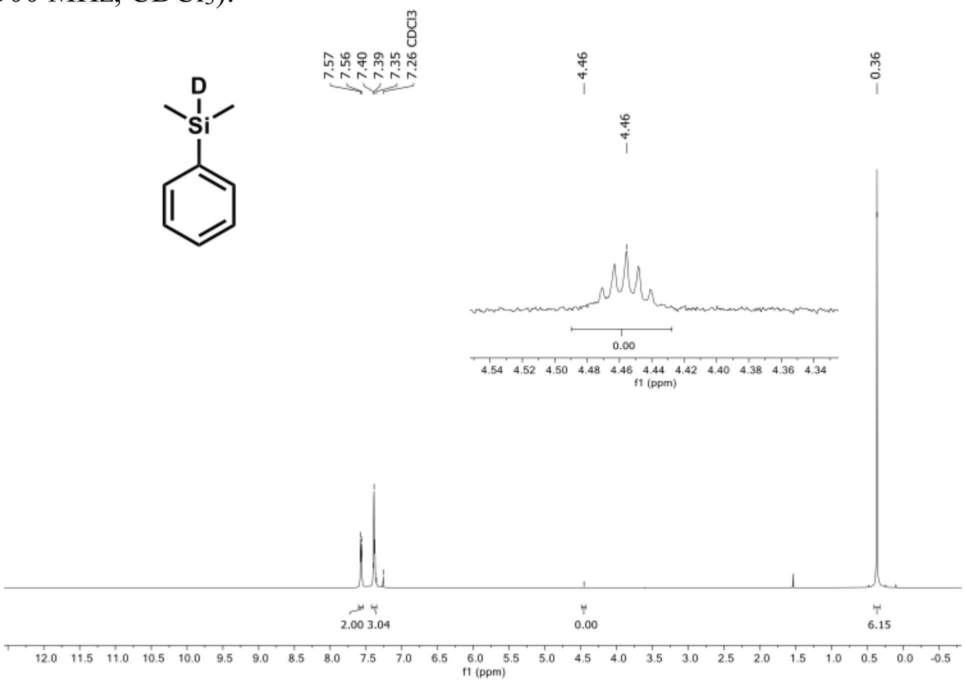

^{13}C NMR (126 MHz, $CDCl_3$):

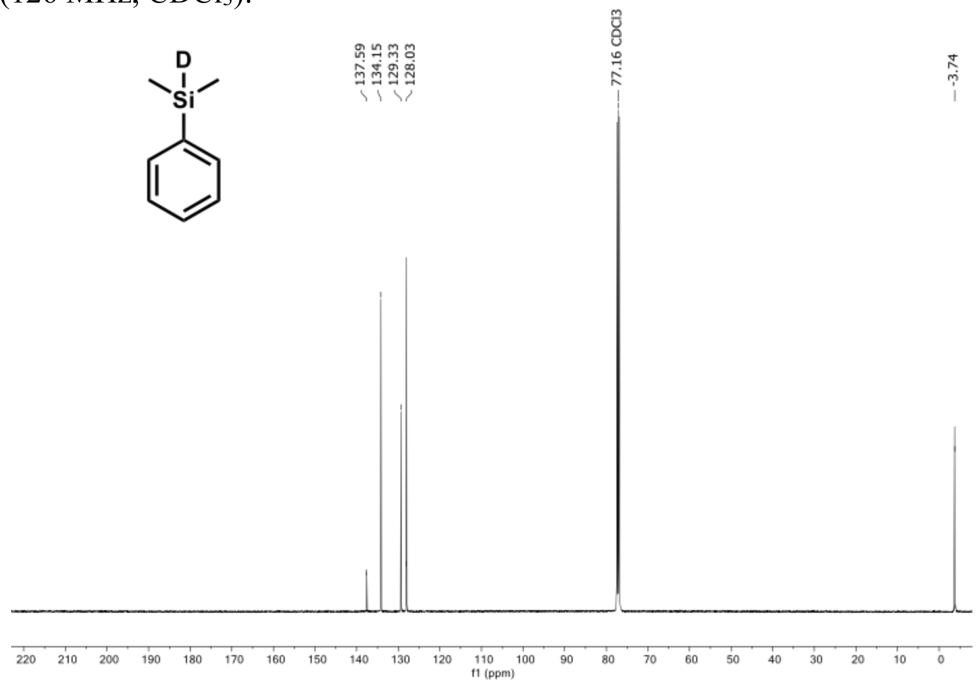

4a: 1-((2-methylallyl)oxy)-4-nitrobenzene

^{1}H NMR (500 MHz, $CDCl_3$):

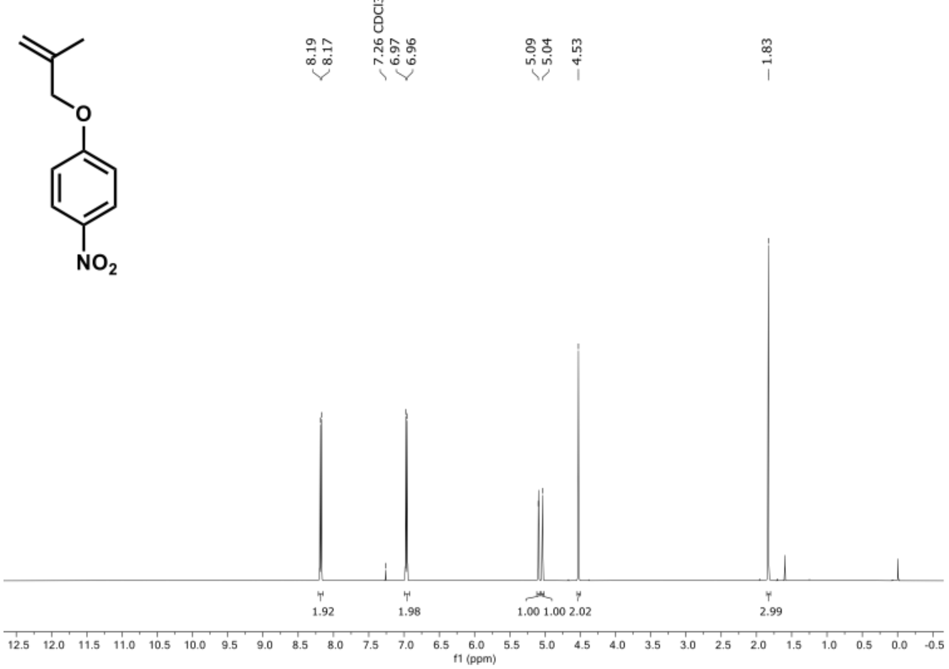

^{13}C NMR (126 MHz, $CDCl_3$):

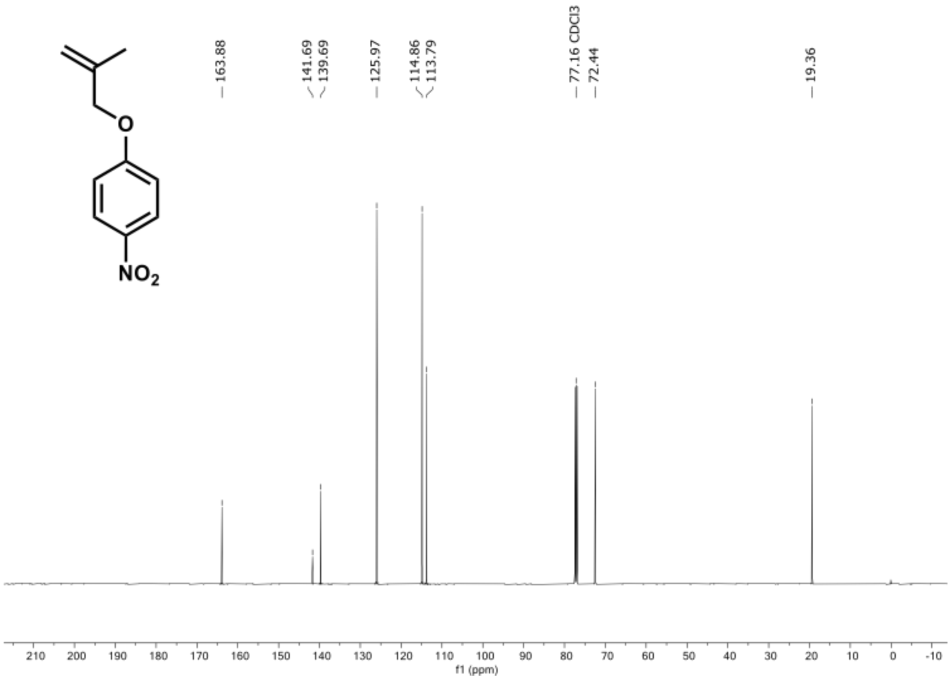

5a: 1-(allyloxy)-4-nitrobenzene

^{1}H NMR (500 MHz, $CDCl_3$):

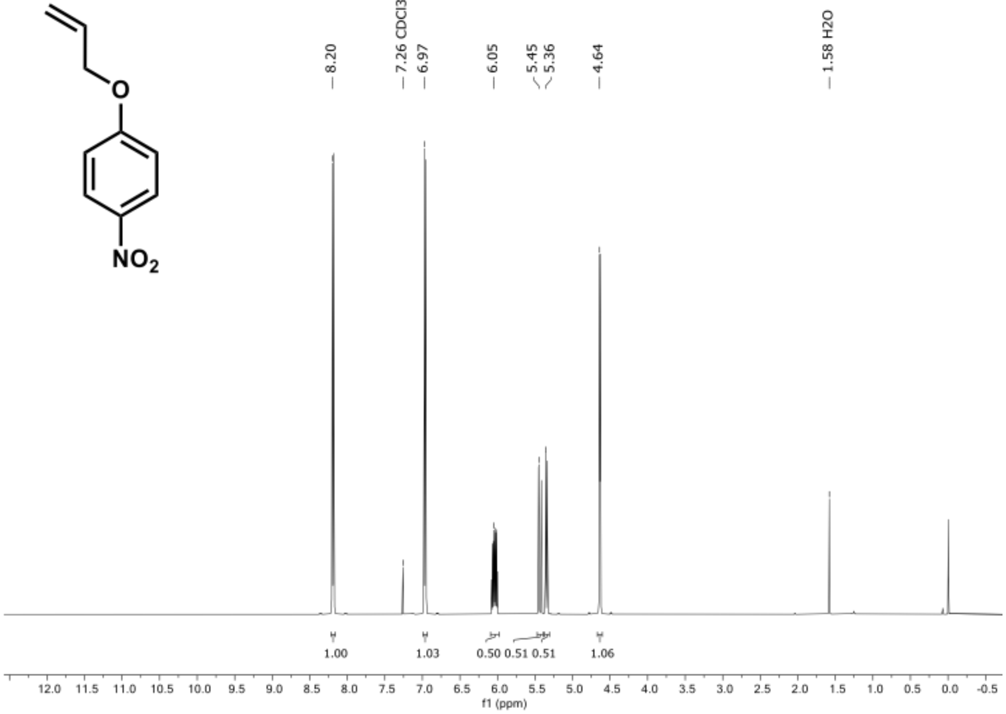

^{13}C NMR (126 MHz, $CDCl_3$):

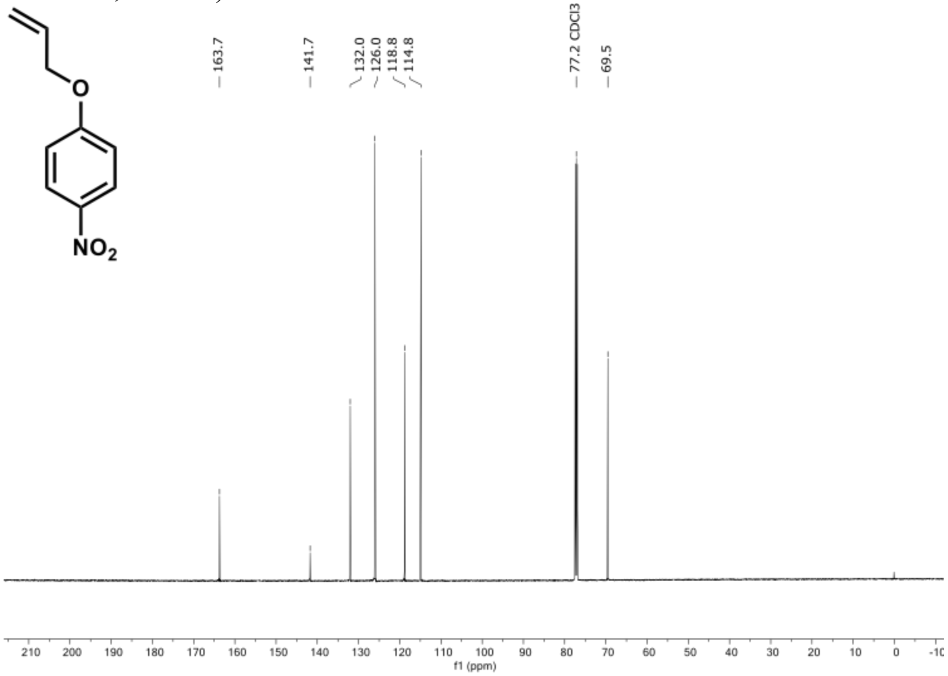

6a: 1-methoxy-4-((2-methylallyl)oxy)benzene

^{1}H NMR (500 MHz, $CDCl_3$):

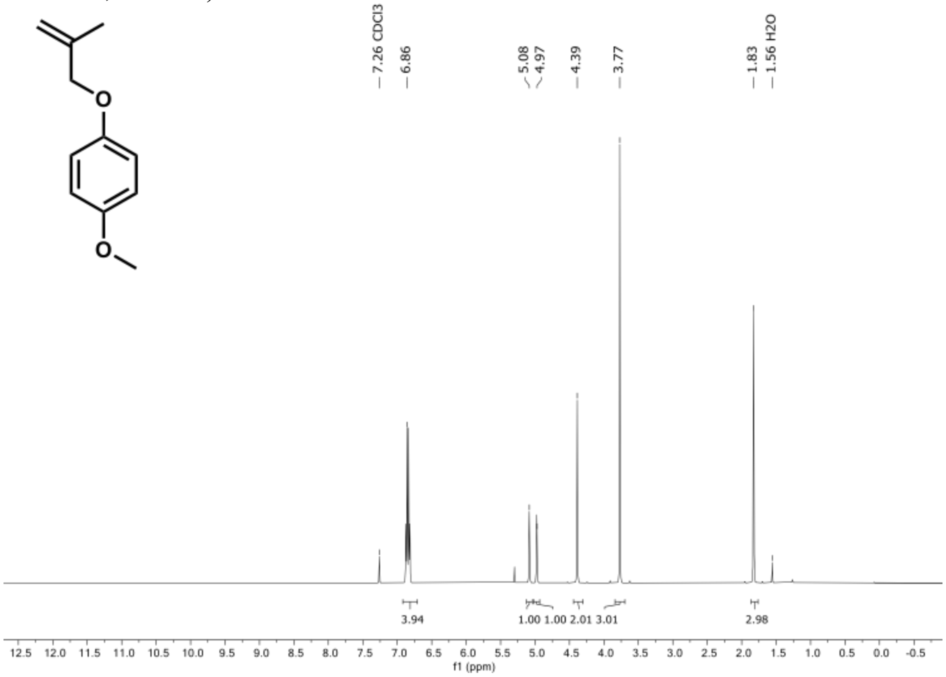

^{13}C NMR (126 MHz, $CDCl_3$):

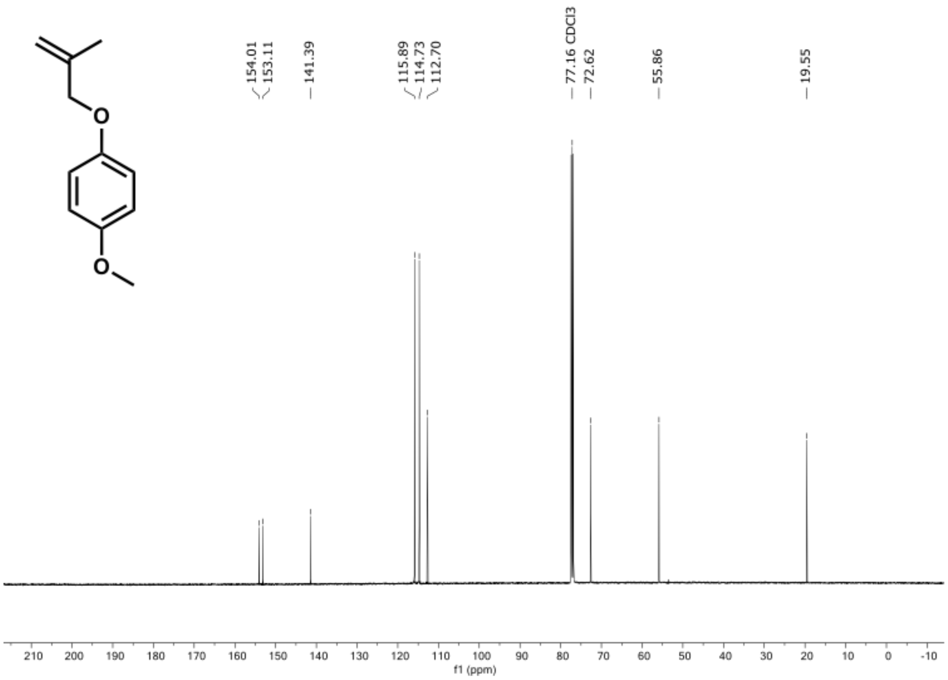

7a: 1-(*tert*-butyl)-4-((2-methylallyl)oxy)benzene

^{1}H NMR (500 MHz, $CDCl_3$):

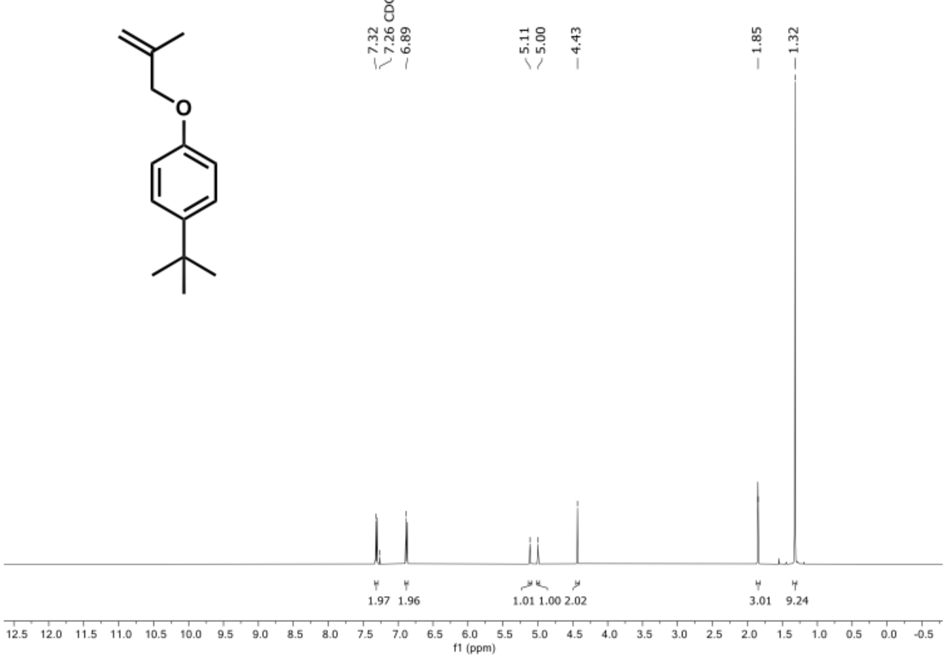

^{13}C NMR (126 MHz, $CDCl_3$):

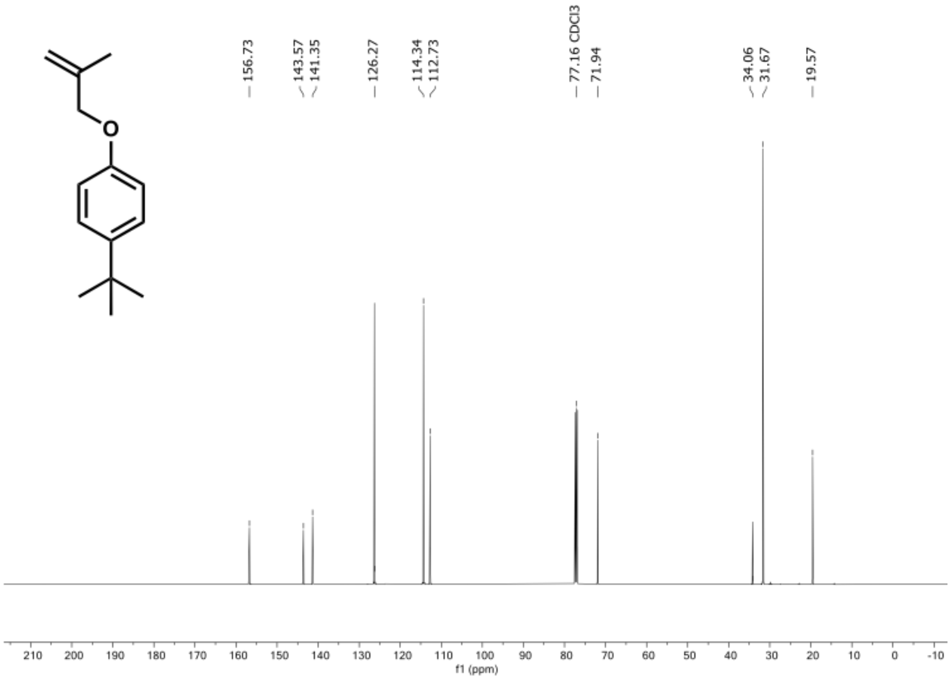

8a: ((2-methallyl)oxy)benzene

^{1}H NMR (500 MHz, $CDCl_3$):

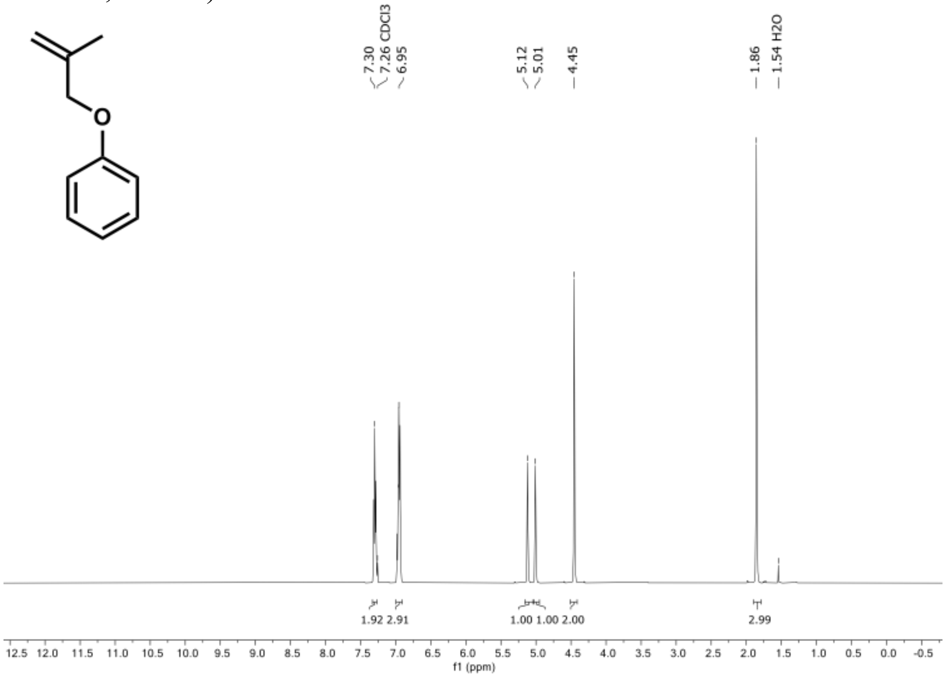

^{13}C NMR (126 MHz, $CDCl_3$):

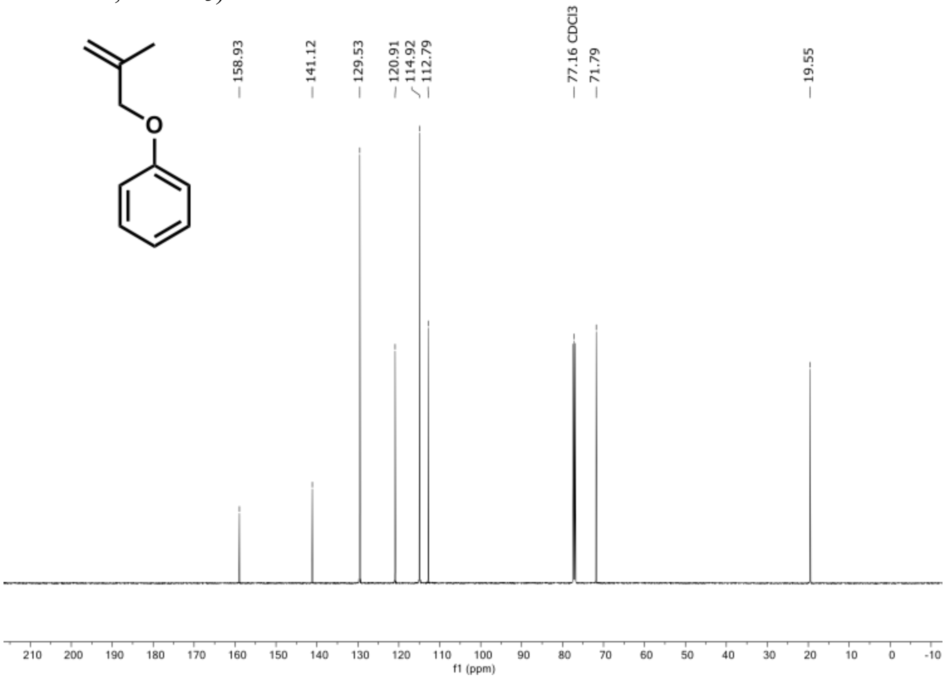

9a: 4-((2-methylallyl)oxy)pyridine

^{1}H NMR (500 MHz, $CDCl_3$):

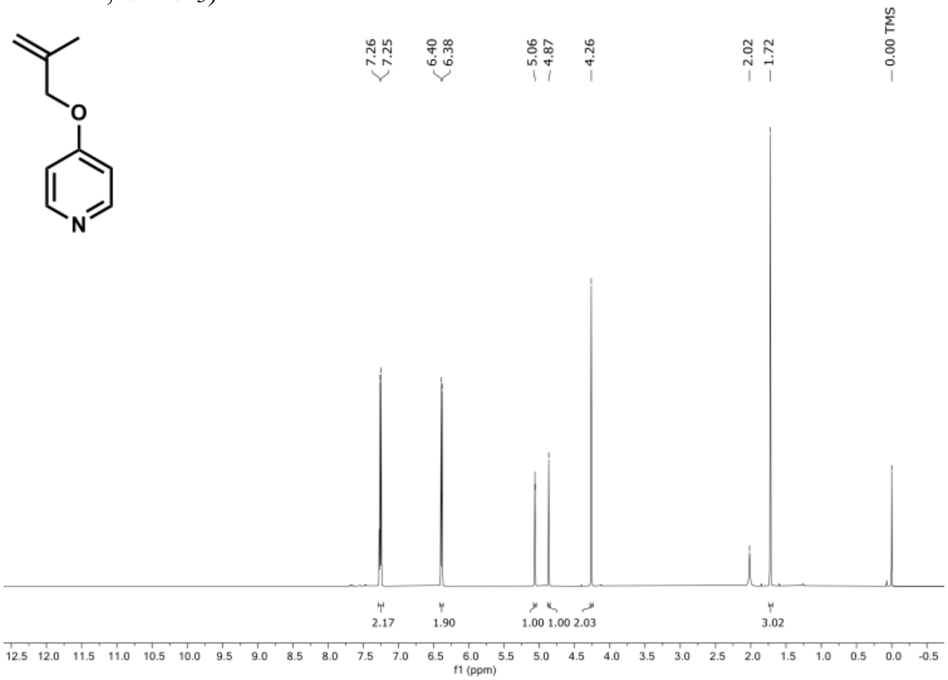

^{13}C NMR (126 MHz, $CDCl_3$):

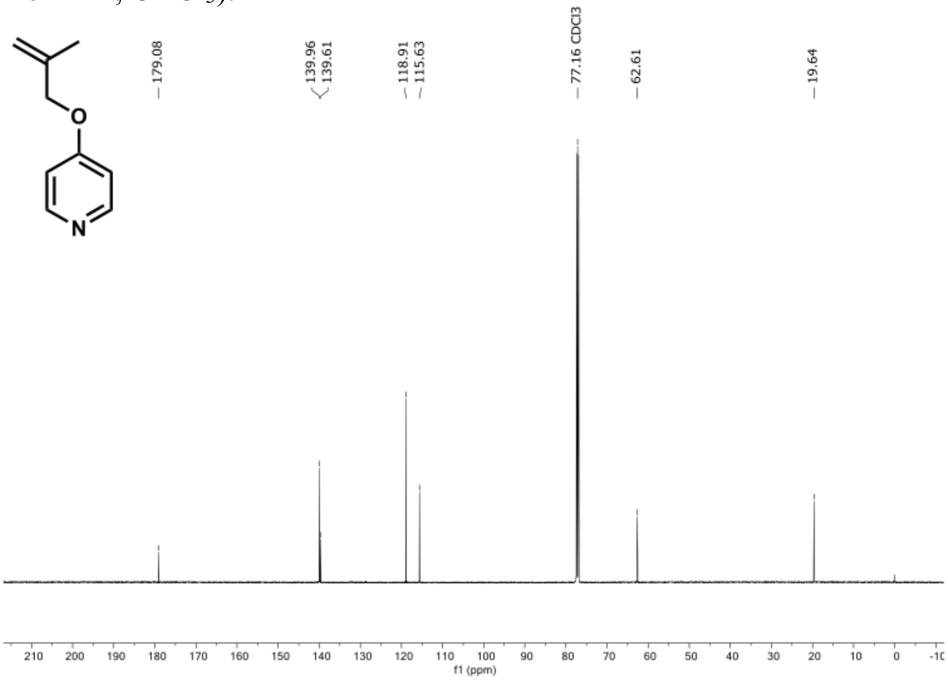

10a: (((2-methylallyl)oxy)methyl)benzene

^{1}H NMR (500 MHz, $CDCl_3$):

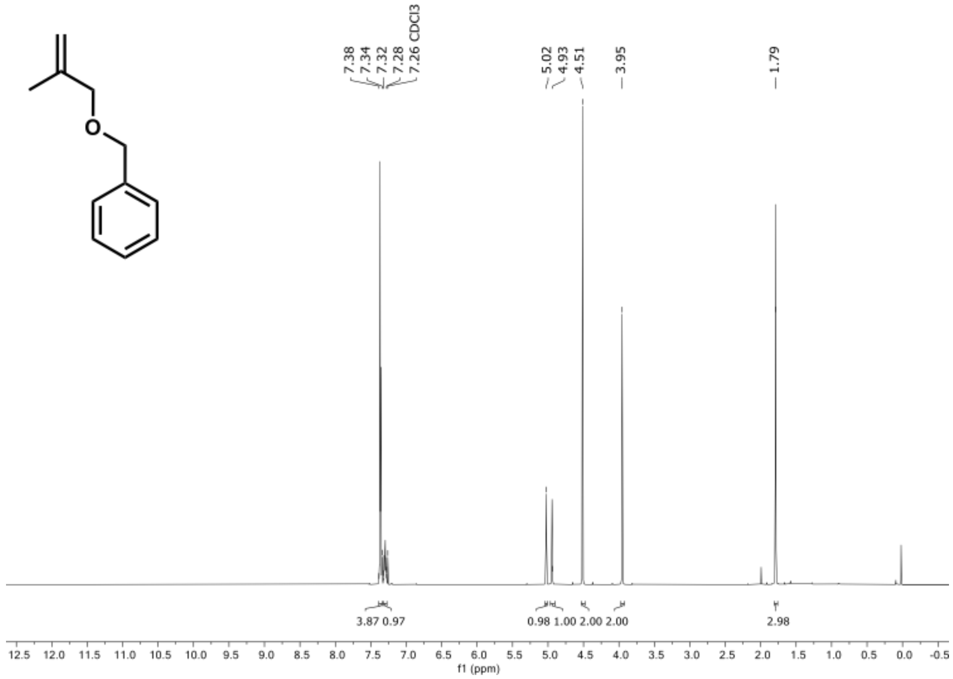

11a: 1-(allyloxy)-4-methoxybenzene

^{1}H NMR (500 MHz, $CDCl_3$):

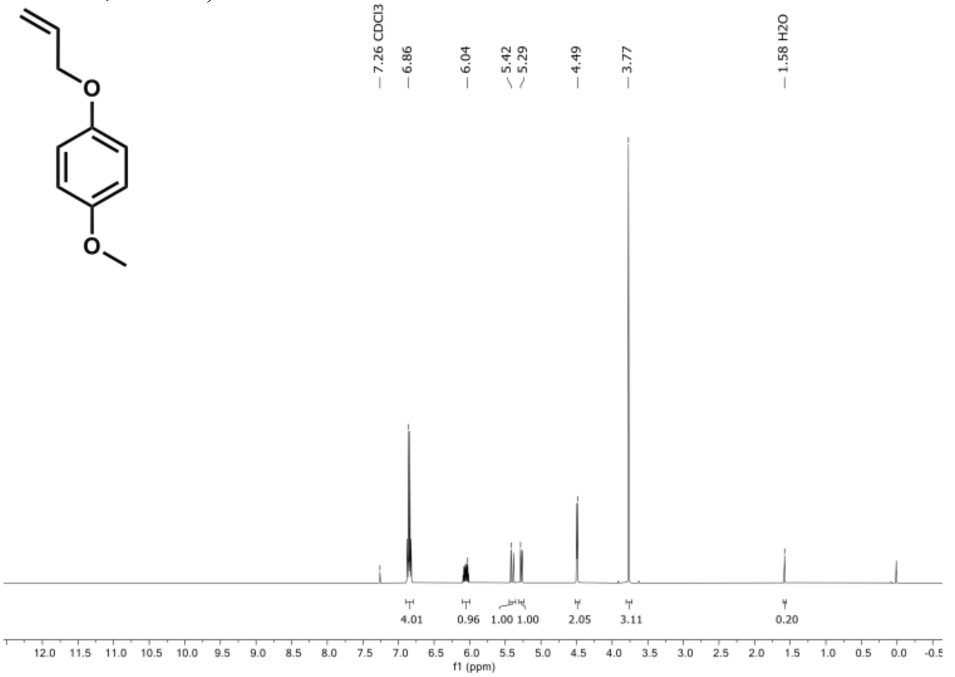

^{13}C NMR (126 MHz, $CDCl_3$):

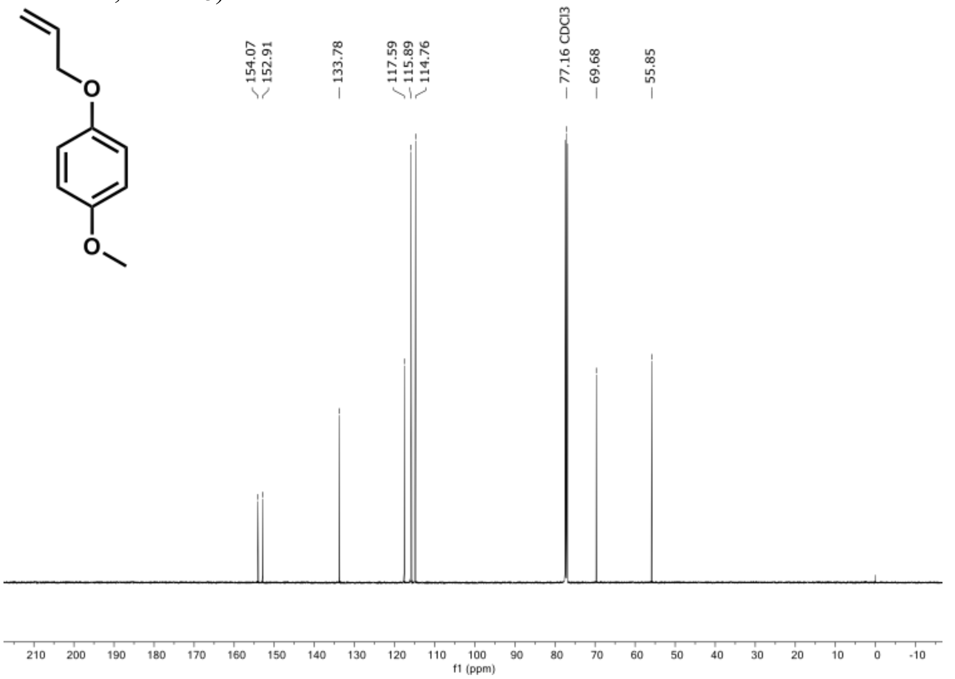

12a: 1-(allyloxy)-4-(tert-butyl)benzene
^{1}H NMR (500 MHz, $CDCl_3$):

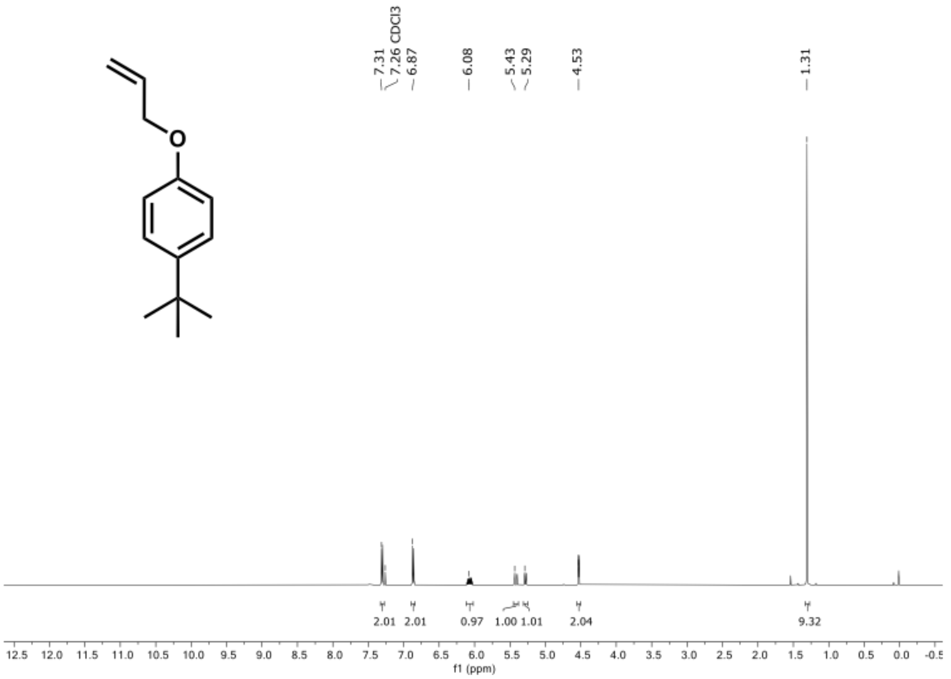

^{13}C NMR (126 MHz, $CDCl_3$):

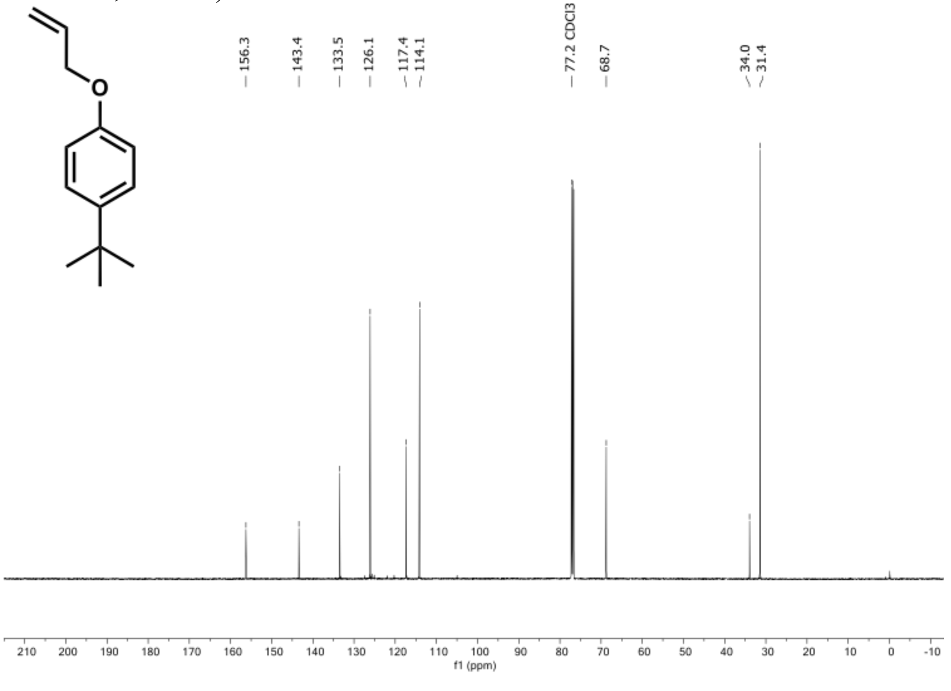

13a: 1-(allyloxy)naphthalene

^{1}H NMR (500 MHz, $CDCl_3$):

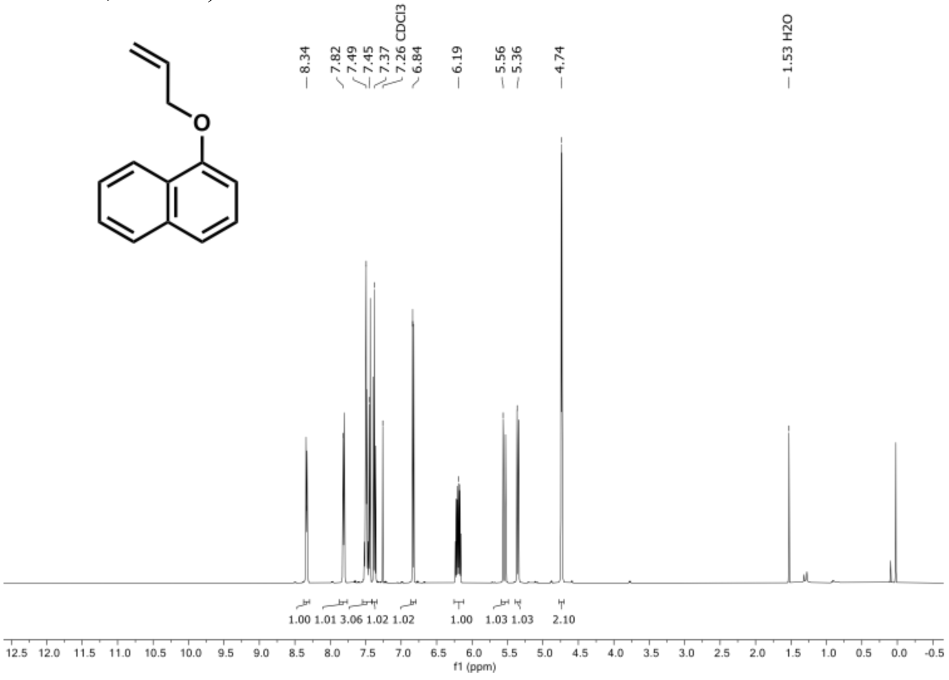

^{13}C NMR (126 MHz, $CDCl_3$):

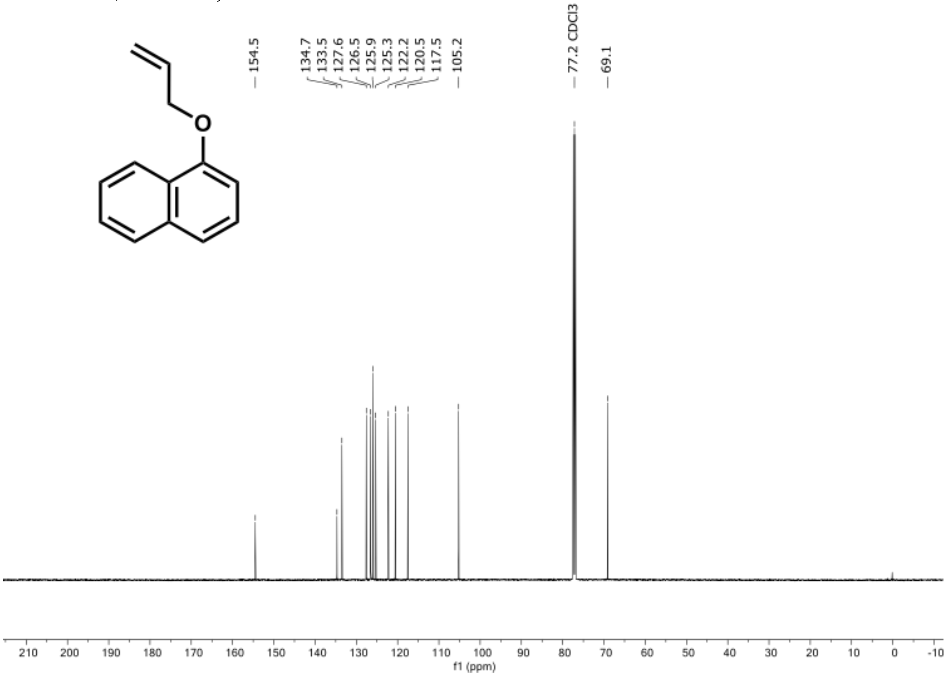

15a:1-methoxy-5-methyl-2,4-dinitrobenzene
^1H NMR (400 MHz, $CDCl_3$)

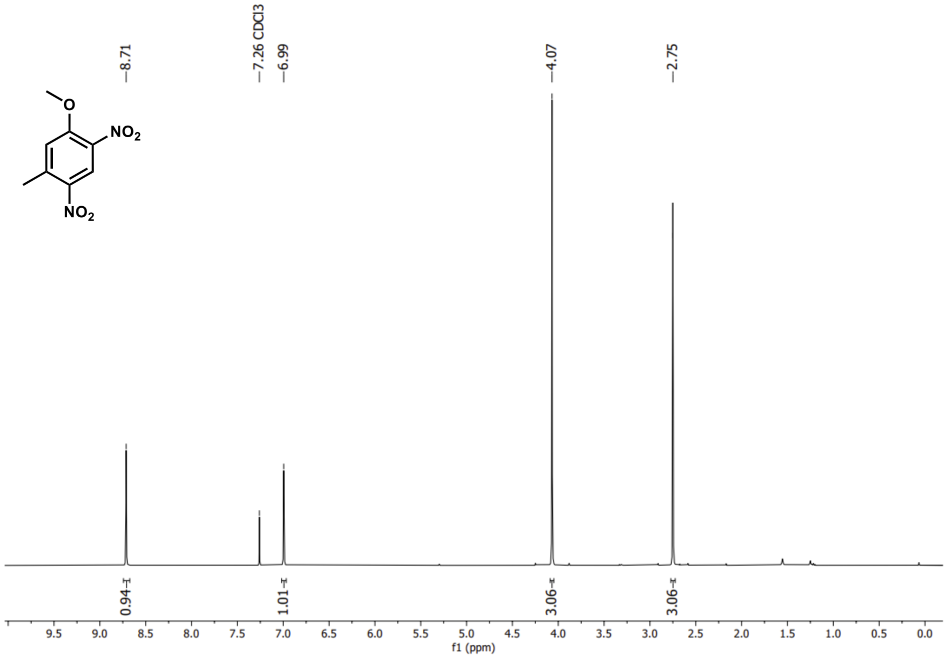

19a: 4-methoxyisophthalonitrile
^1H NMR (400 MHz, CD_3CN)

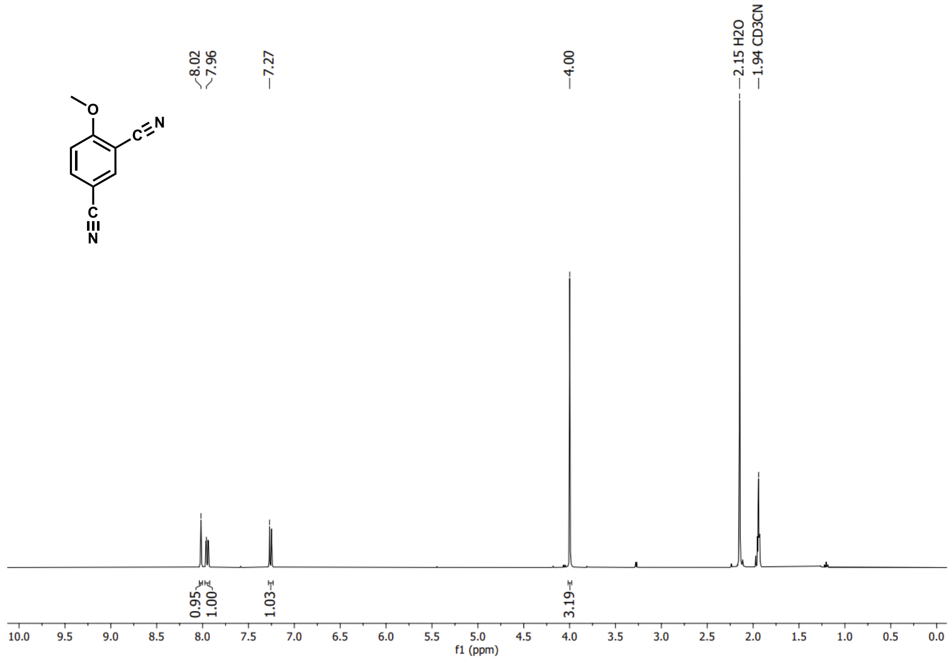

14b: 2,4-dinitrocyclohexa-1,3-dien-1-olate

^{1}H NMR spectrum of **14b** (500 MHz, D2O)

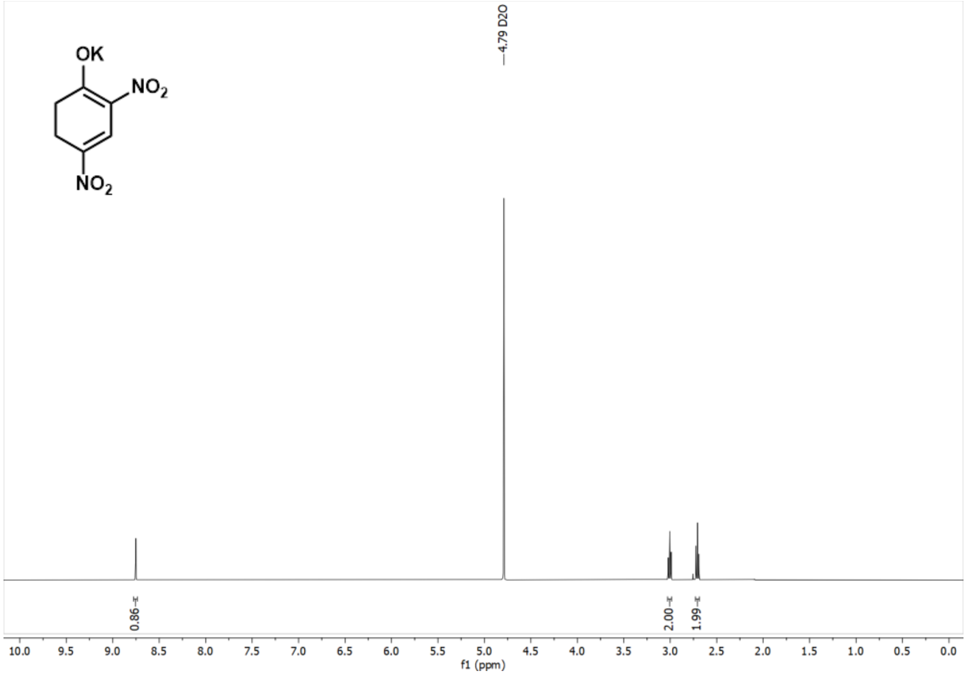

^{13}C NMR spectrum of **14b**(126 MHz, CDCl3):

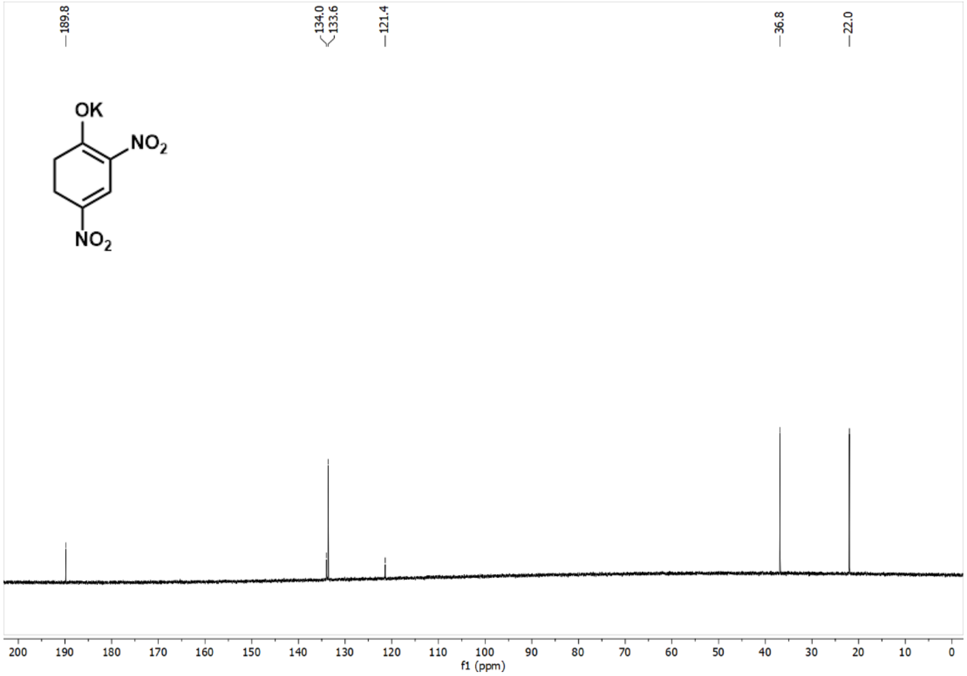

HSQC NMR spectrum of **14b** (500 MHz, D2O):

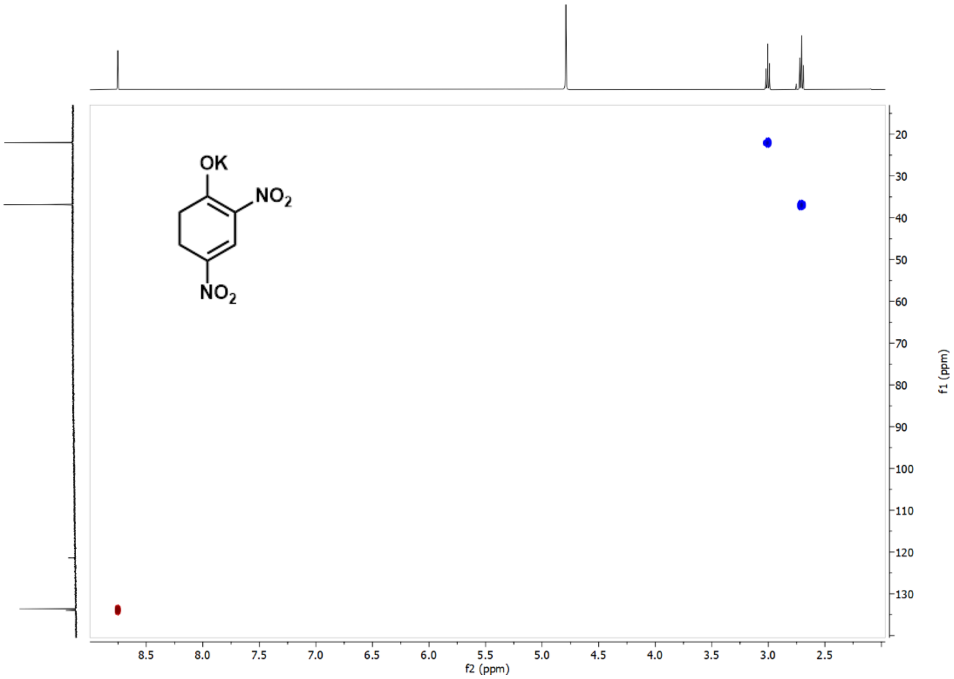

HMBC NMR spectrum of **14b** (126 MHz, CDCl3):

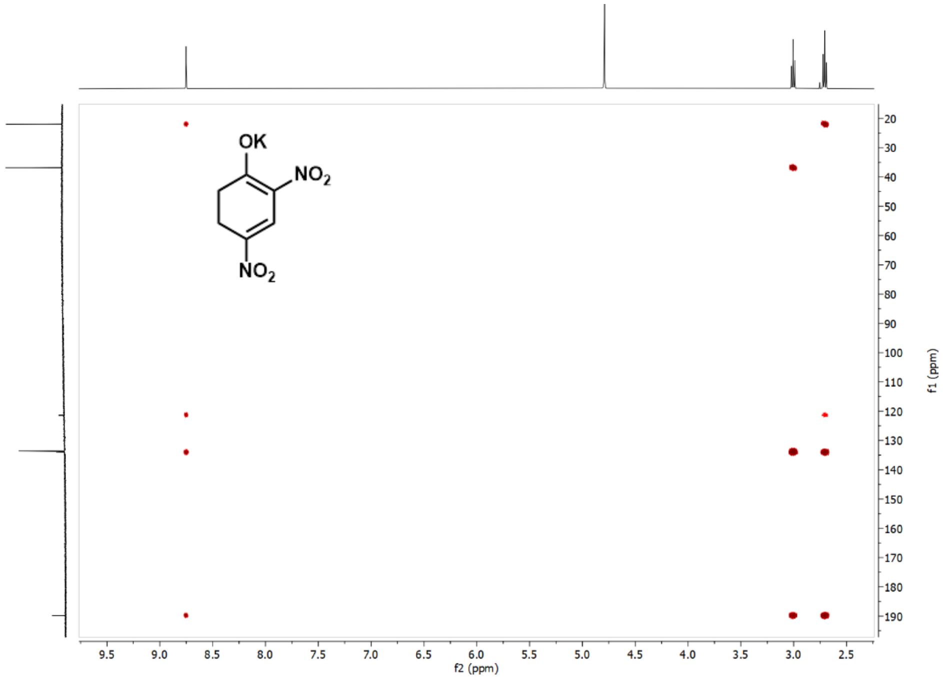

15b: potassium 5-methyl-2,4-dinitrocyclohexa-1,3-dien-1-olate

^{1}H NMR spectrum of **15b** (500 MHz, D2O)

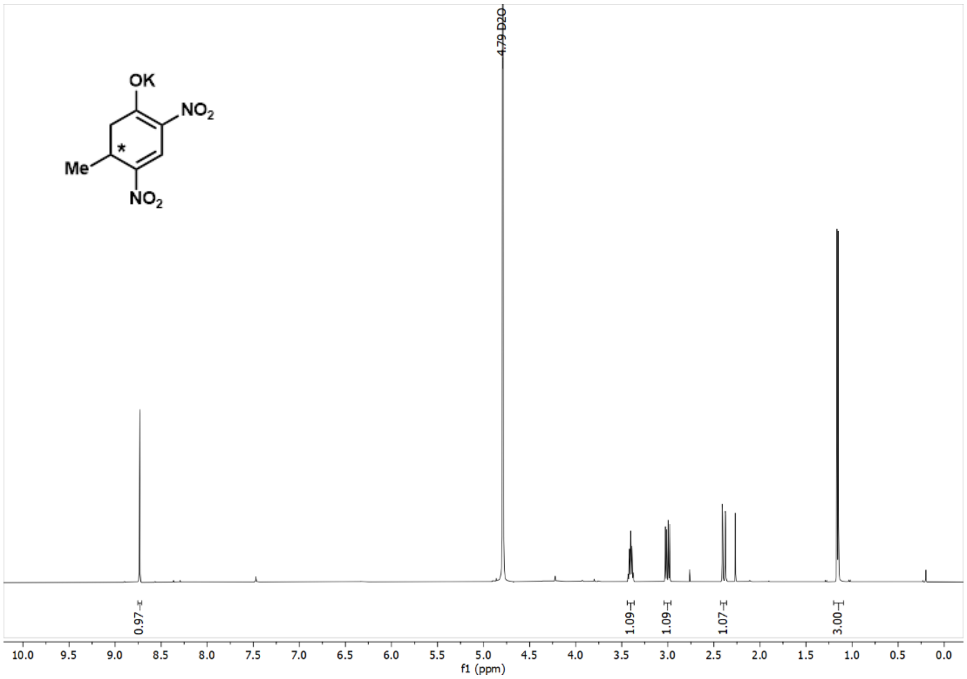

^{13}C NMR spectrum of **15b**(126 MHz, CDCl3):

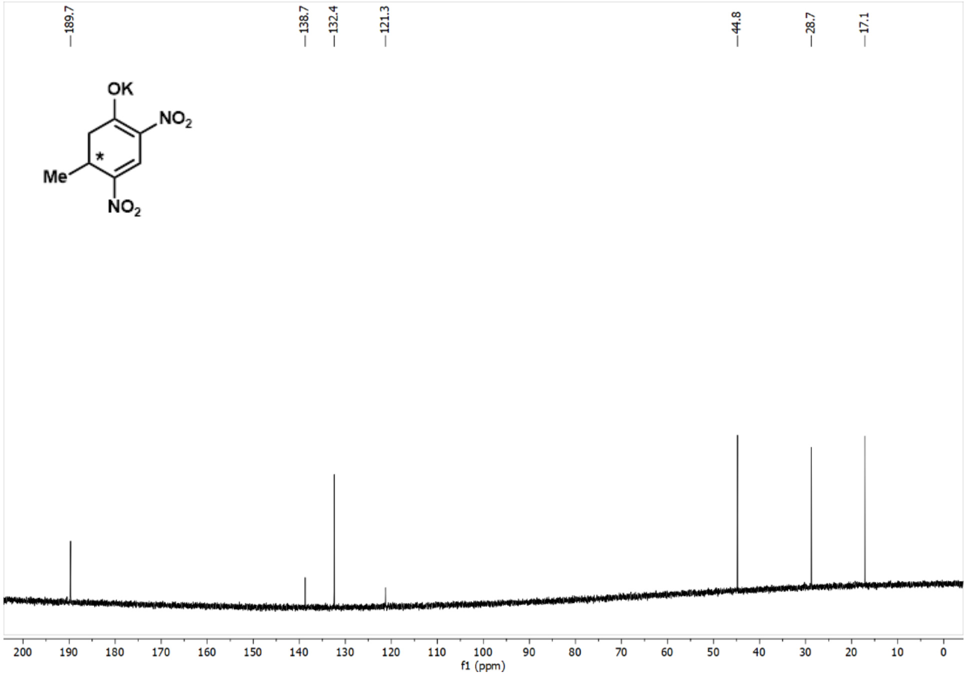

HSQC NMR spectrum of **15b** (500 MHz, D2O)

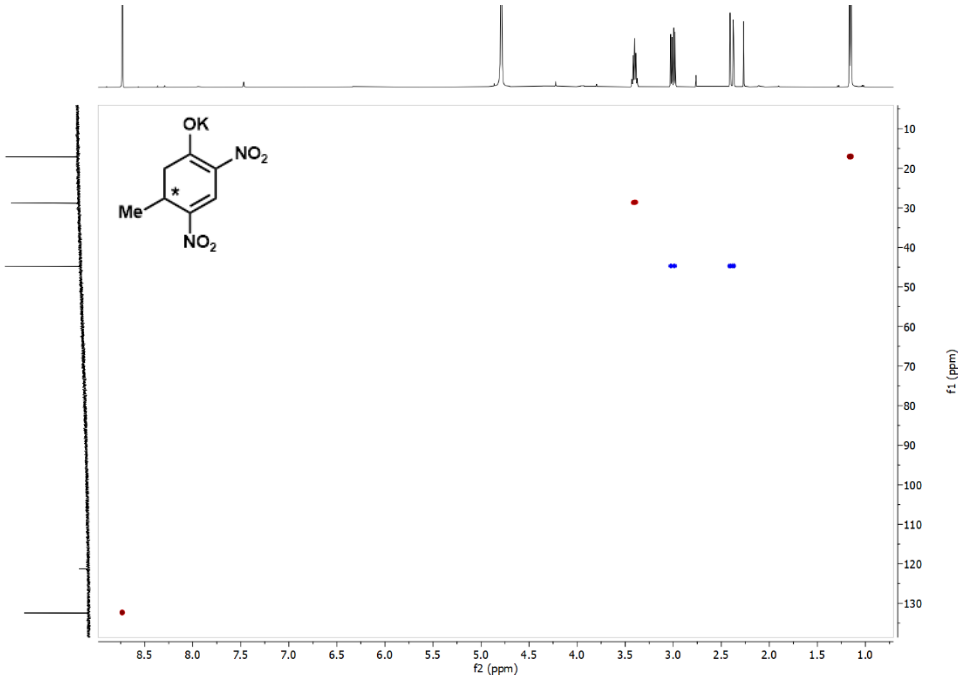

HMBC NMR spectrum of **15b** (126 MHz, CDCl3):

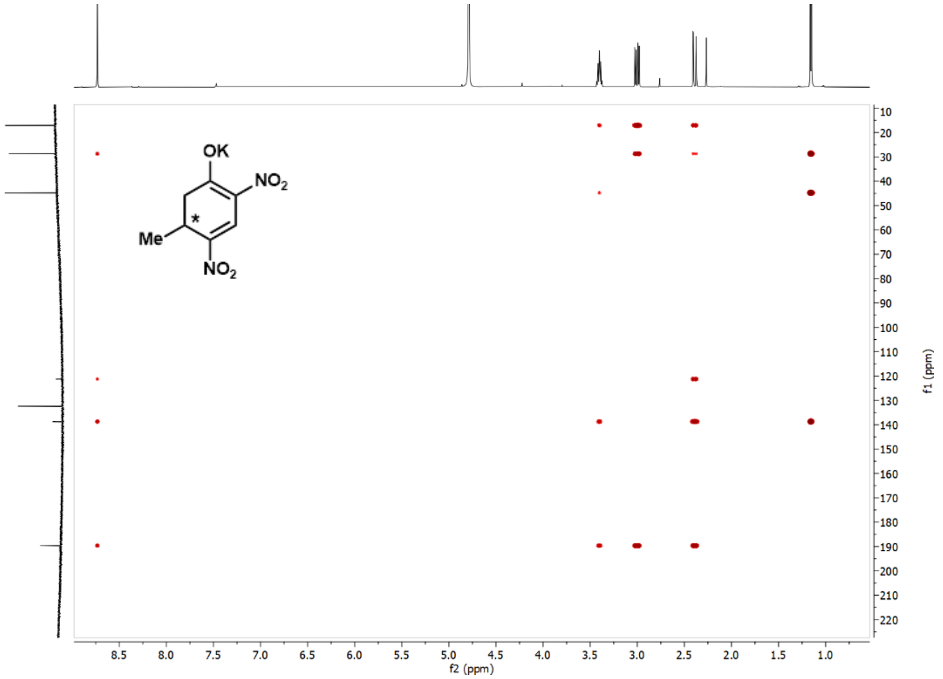

18b: potassium 4-cyano-2-nitrocyclohexa-1,3-dien-1-olate
^{1}H NMR spectrum of **18b** (500 MHz, D2O)

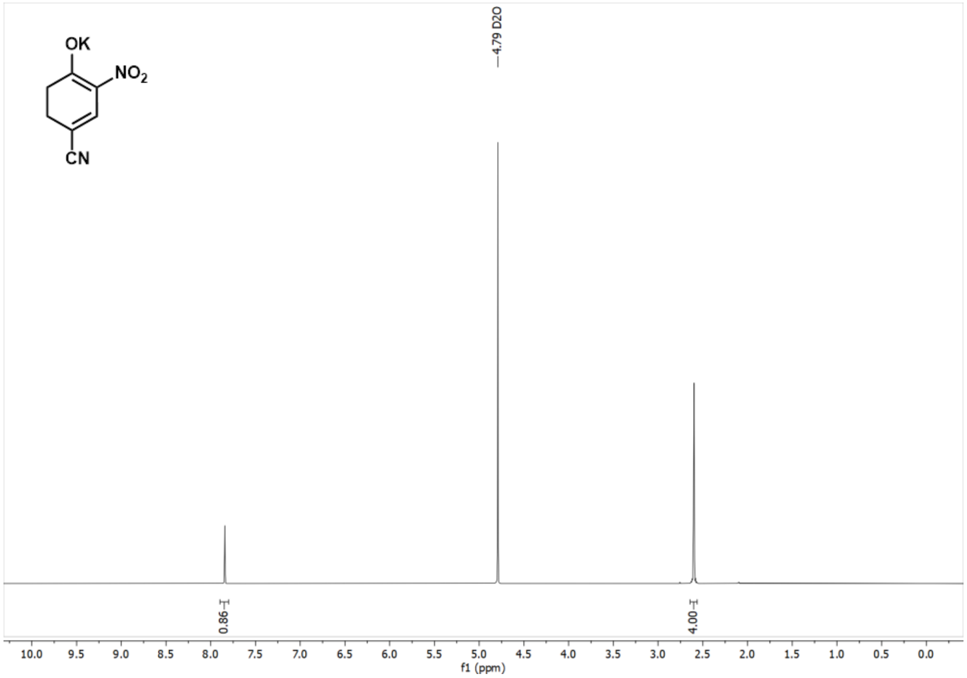

^{13}C NMR spectrum of **18b** (126 MHz, CDCl3):

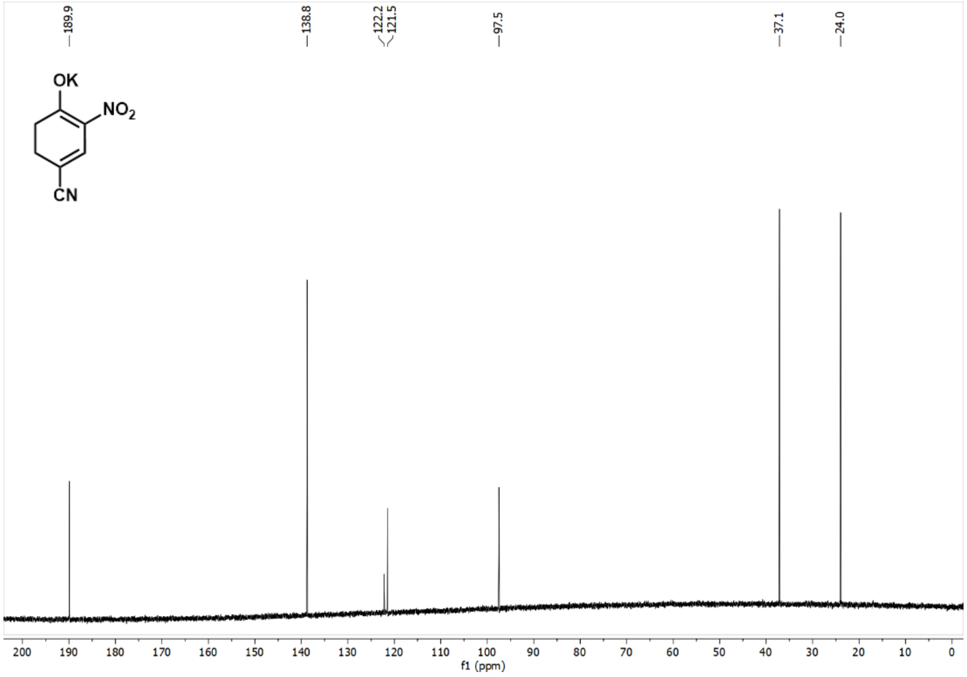

HSQC NMR spectrum of **18b**(500 MHz, D2O)

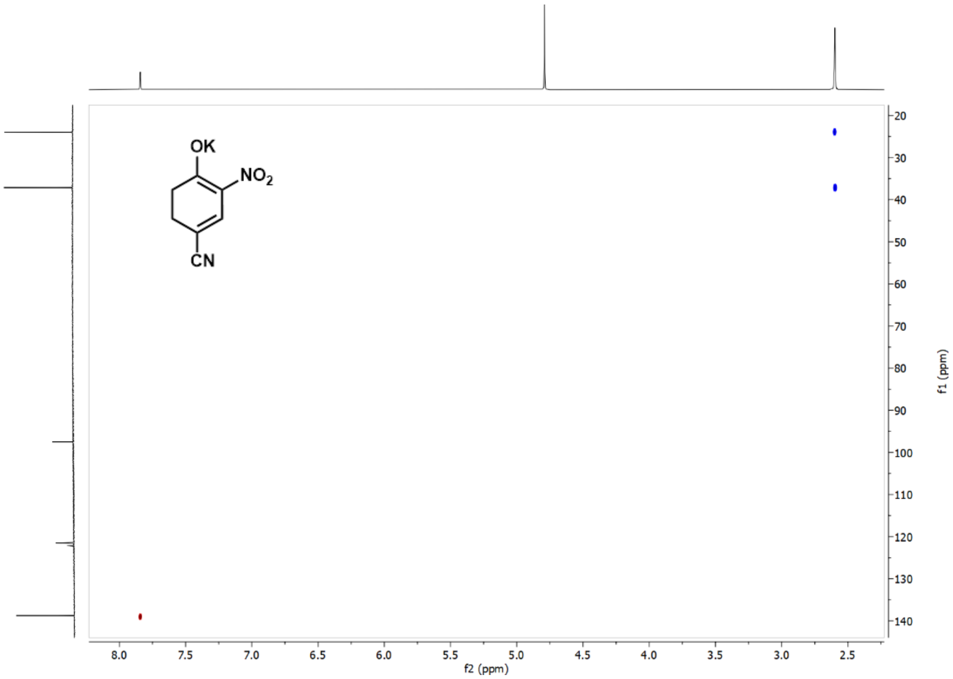

HMBC NMR spectrum of **18b** (126 MHz, CDCl3):

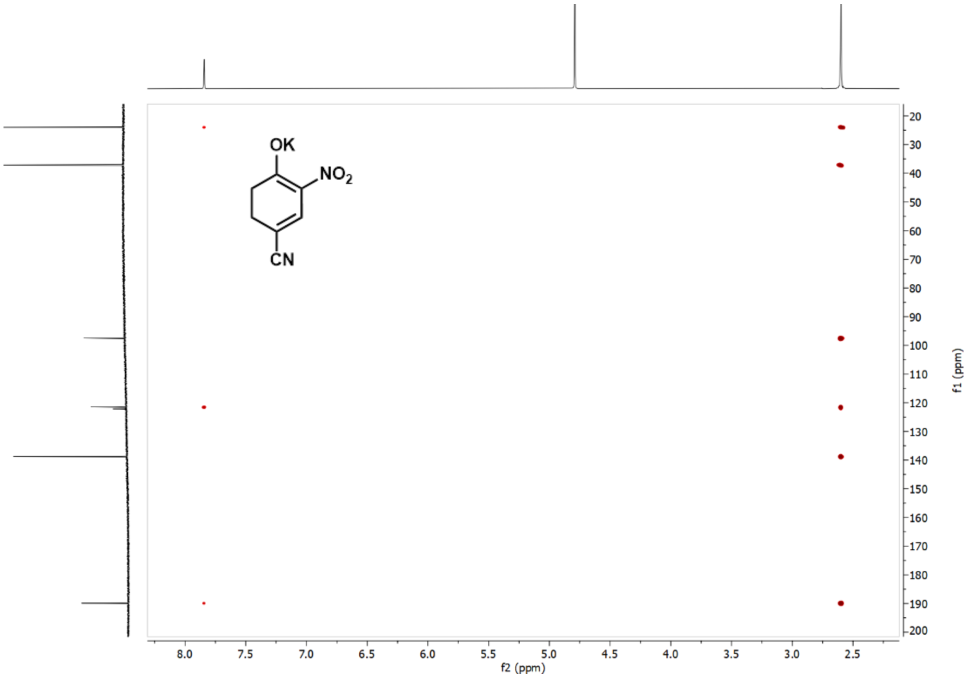

17b: potassium 4-cyano-2-nitrocyclohexa-1,3-dien-1-olate

^{1}H NMR spectrum of **17b** (500 MHz, D2O)

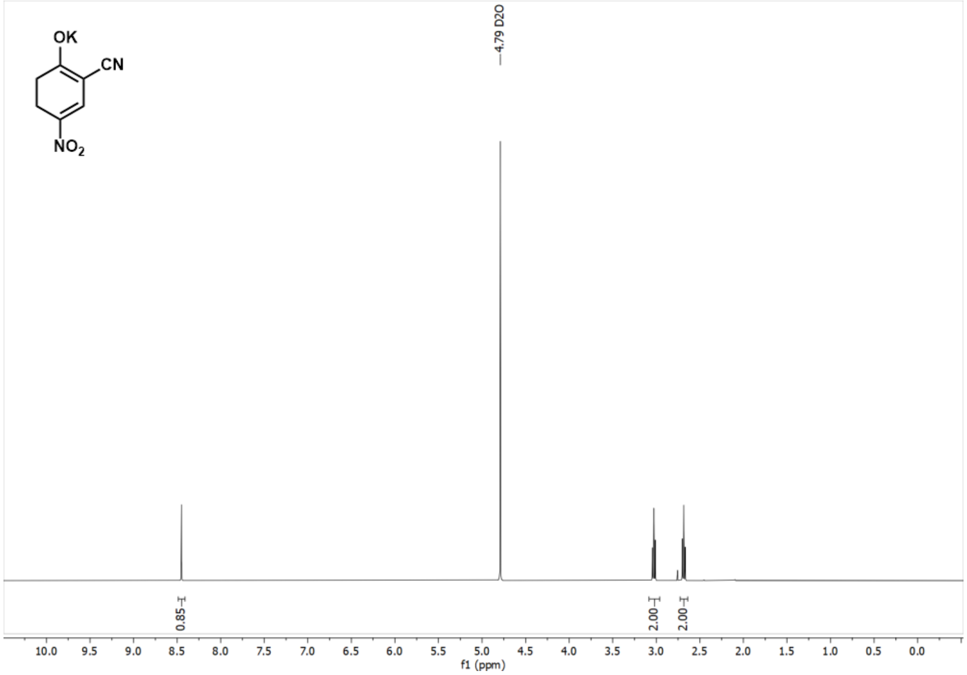

^{13}C NMR spectrum of **17b** (126 MHz, CDCl3):

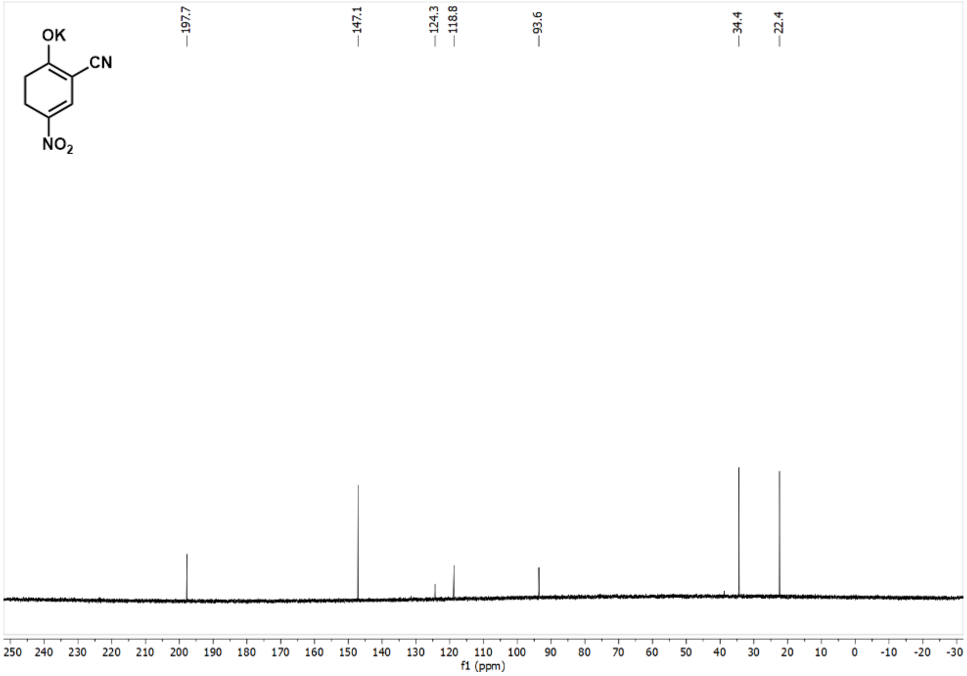

HSQC NMR spectrum of **17b** (500 MHz, D2O)

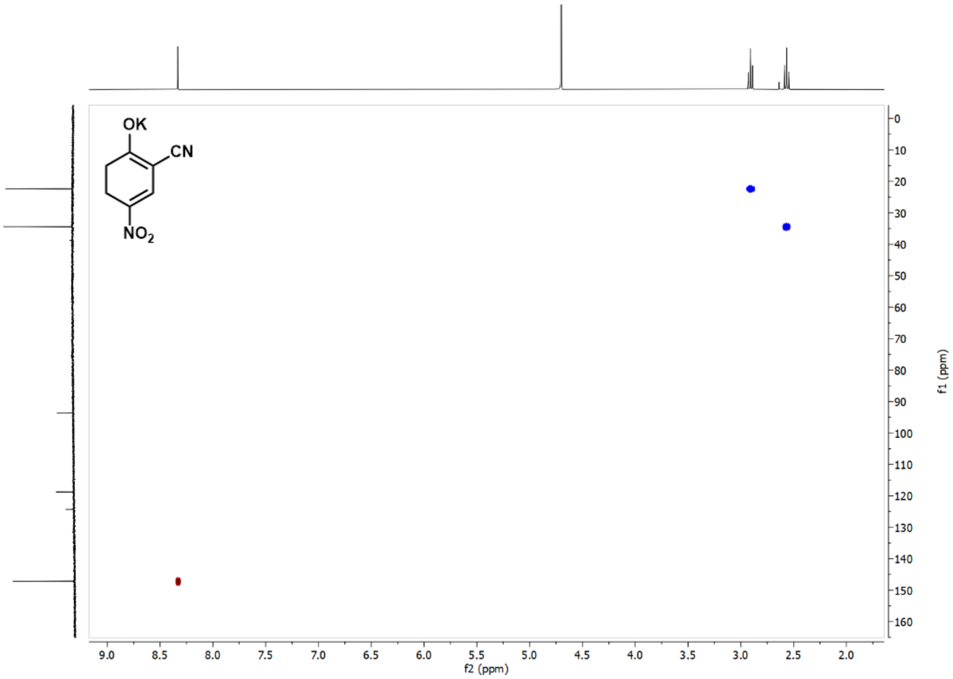

HMBC NMR spectrum of **17b** (126 MHz, CDCl3):

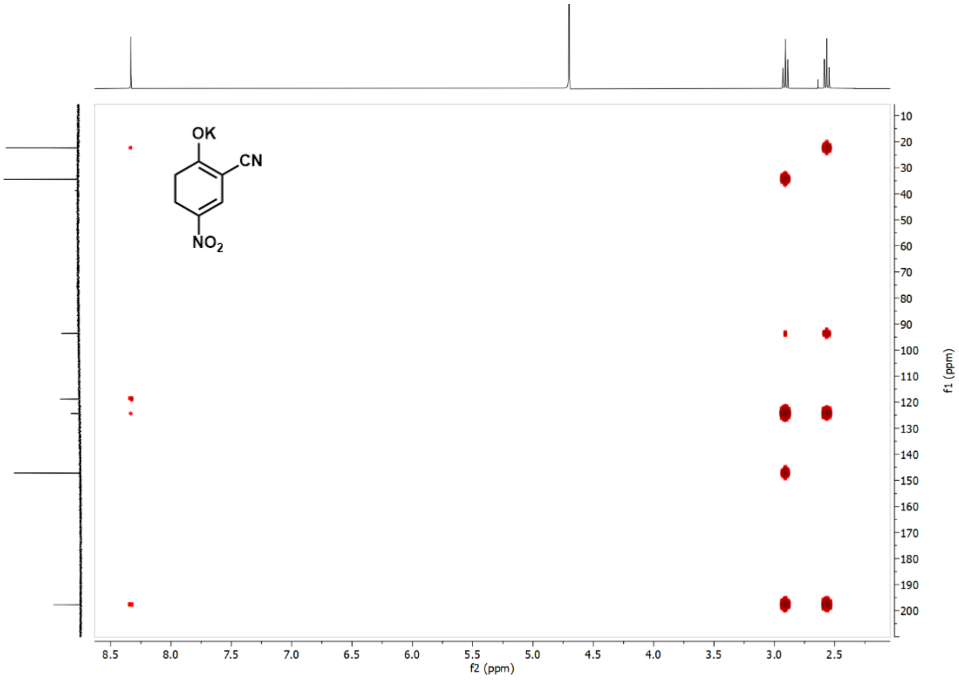

18b: potassium 4-(methoxycarbonyl)-2-nitrocyclohexa-1,3-dien-1-olate

^{1}H NMR spectrum of **18b** (500 MHz, D2O)

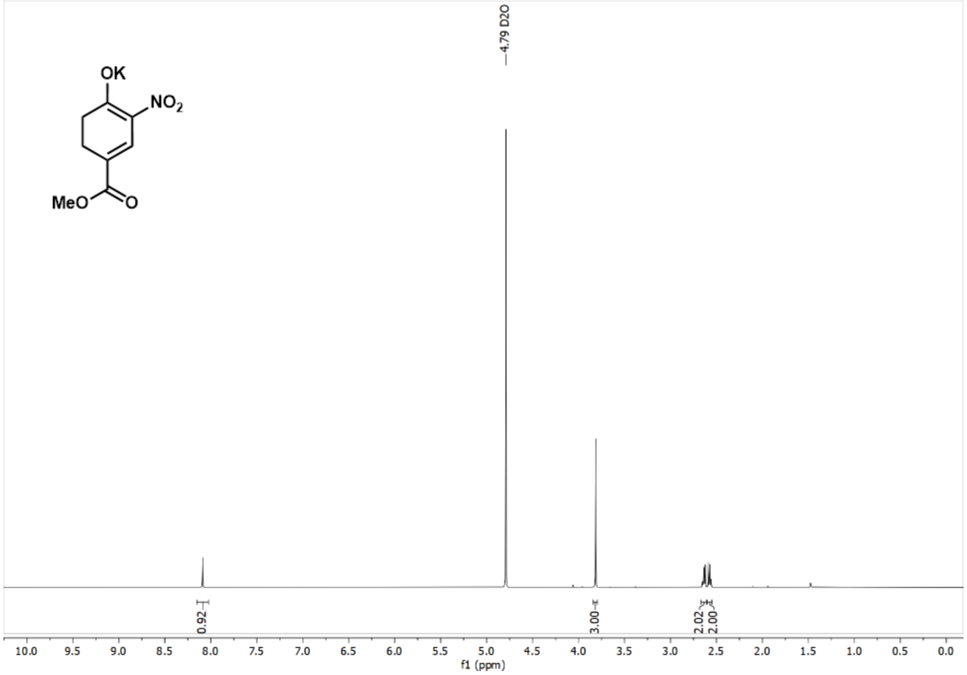

^{13}C NMR spectrum of **18b** (126 MHz, CDCl3):

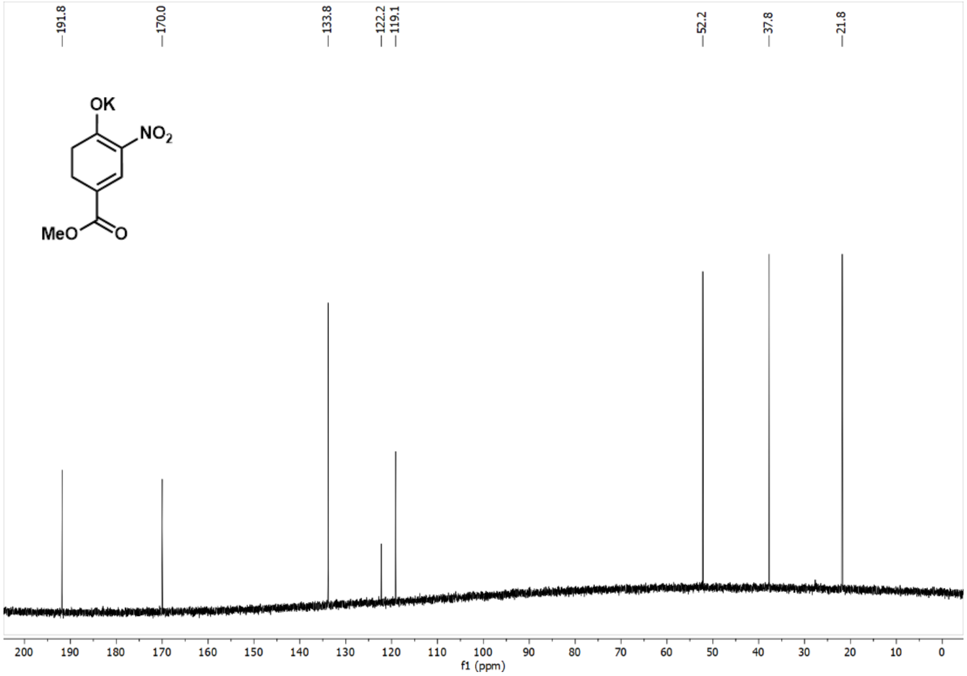

HSQC NMR spectrum of **18b** (500 MHz, D2O)

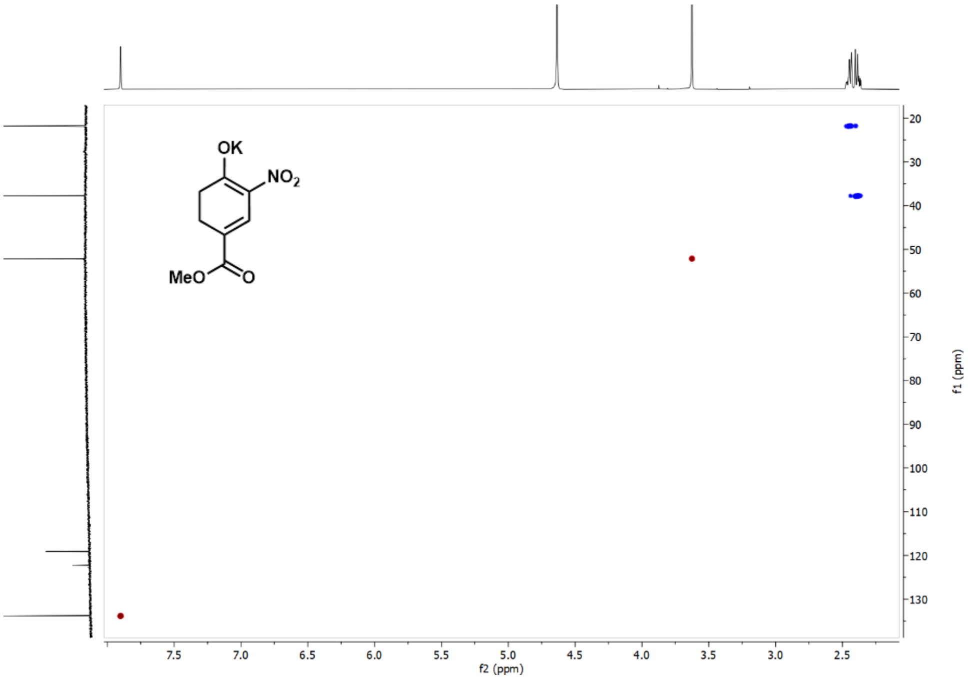

HMBC NMR spectrum of **18b** (126 MHz, CDCl3):

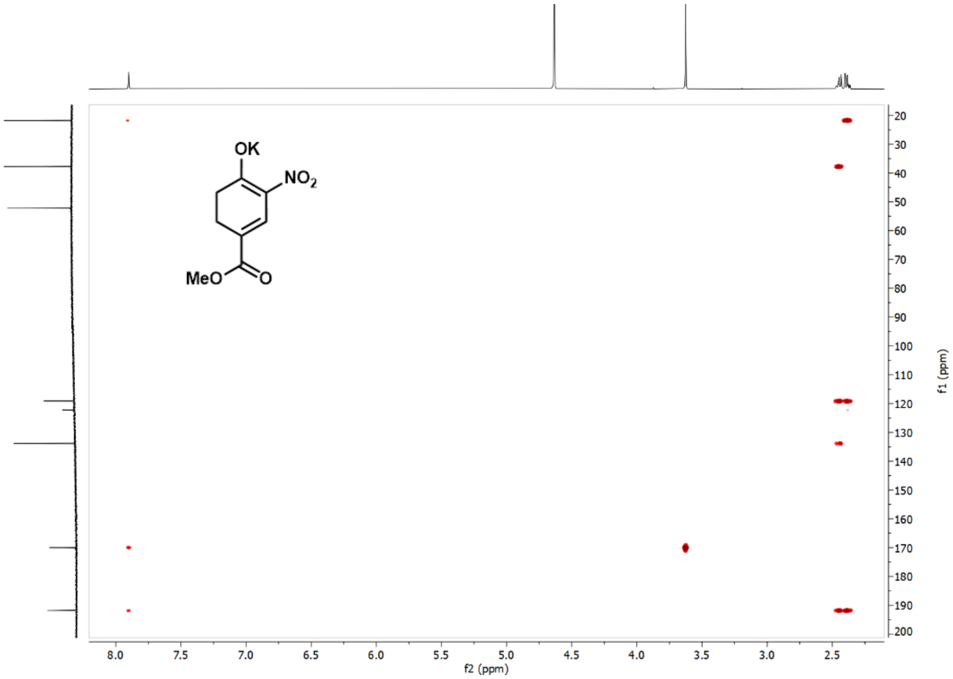

5D-14b: potassium 2,4-dinitrocyclohexa-1,3-dien-1-olate-5-d

^{1}H NMR spectrum of **5D-14b** (500 MHz, D2O):

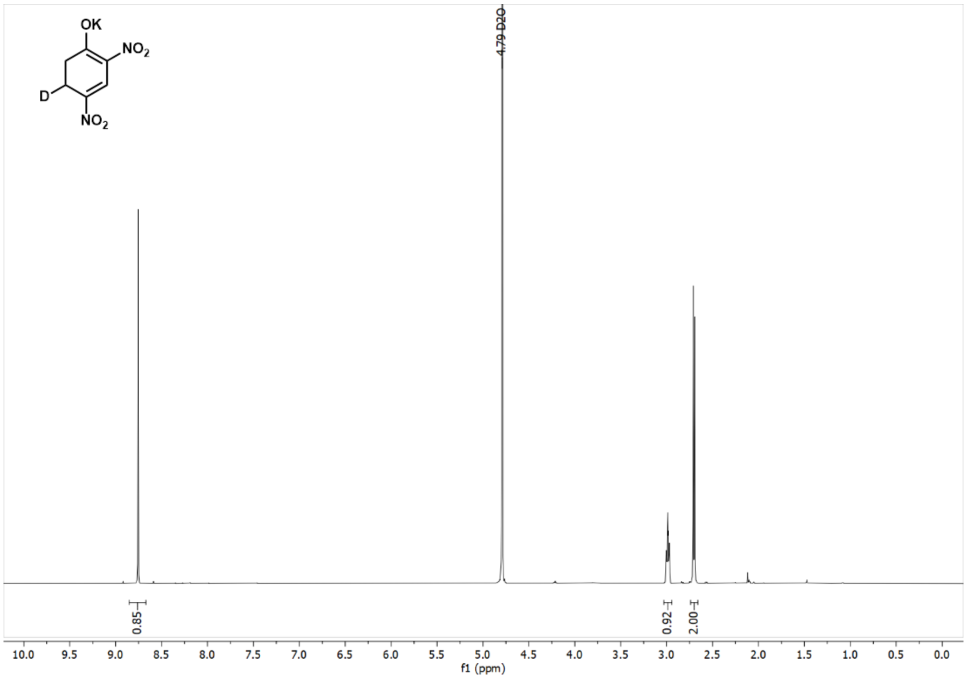

HSQC NMR spectrum of **5D-14b** (500 MHz, D2O):

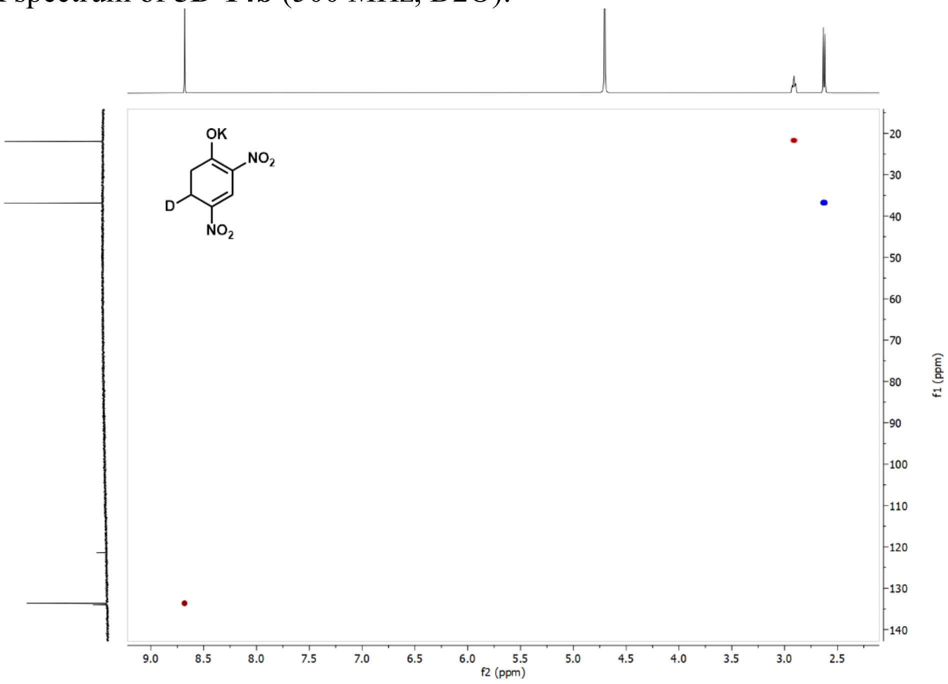

S5: 1,2-bis(4-(trifluoromethyl)phenyl)diazene 1-oxide
^{1}H NMR (500 MHz, $CDCl_3$):

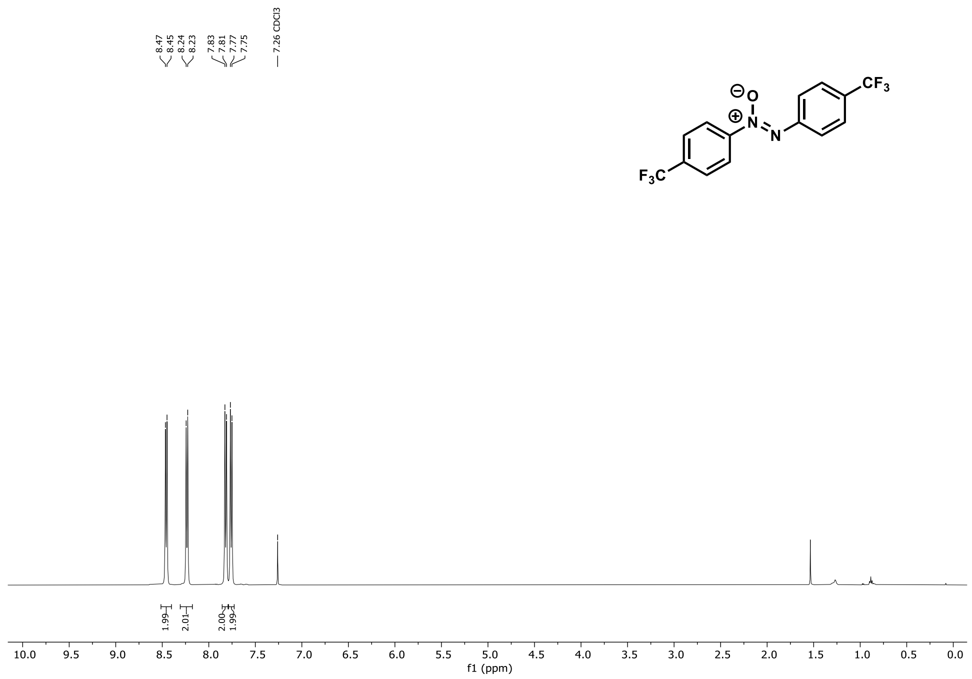

Small molecule X-ray crystallography

Data Collection

An orange crystal with approximate dimensions 0.08 × 0.08 × 0.06 mm^3 was selected under oil under ambient conditions and attached to the tip of a MiTeGen MicroMount©. The crystal was mounted in a stream of cold nitrogen at 100(1) K and centered in the X-ray beam by using a video camera.

The crystal evaluation and data collection were performed on a Bruker D8 VENTURE PhotonIII four-circle diffractometer with Cu Kα (λ = 1.54178 Å) radiation and the detector to crystal distance of 5.0 cm.

The initial cell constants were obtained from a 180° φ scan conducted at a 2θ = 50° angle with the exposure time of 1 second per frame. The reflections were successfully indexed by an automated indexing routine built in the APEX3 program. The final cell constants were calculated from a set of 9459 strong reflections from the actual data collection.

The data were collected by using the full sphere data collection routine to survey the reciprocal space to the extent of a full sphere to a resolution of 0.78 Å. A total of 43998 data were harvested by collecting 15 sets of frames with 0.9° scans in ω and φ with an exposure time 1–3 sec

per frame. These highly redundant datasets were corrected for Lorentz and polarization effects. The absorption correction was based on fitting a function to the empirical transmission surface as sampled by multiple equivalent measurements.[30,31]

Structure Solution and Refinement

The systematic absences in the diffraction data were uniquely consistent for the space group *Pbca* that yielded chemically reasonable and computationally stable results of refinement.[32]

A successful solution by intrinsic phasing provided most non-hydrogen atoms from the *E*-map. The remaining non-hydrogen atoms were located in an alternating series of least-squares cycles and difference Fourier maps. All non-hydrogen atoms were refined with anisotropic displacement coefficients. All hydrogen atoms were included in the structure factor calculation at idealized positions and were allowed to ride on the neighboring atoms with relative isotropic displacement coefficients.

The final least-squares refinement of 281 parameters against 4199 data resulted in residuals *R* (based on F^2 for $I \geq 2\sigma$) and *wR* (based on F^2 for all data) of 0.0299 and 0.0723, respectively. The final difference Fourier map was featureless.

Summary

Crystal Data for $C_{14}H_{13}K_2N_5O_{10}$ (M =489.49 g/mol): orthorhombic, space group Pbca (no. 61), a = 13.6247(17) Å, b = 7.3982(7) Å, c = 38.728(4) Å, V = 3903.7(7) Å^3, Z = 8, T = 100.00 K, μ(Cu Kα) = 4.913 mm^{-1}, $Dcalc$ = 1.666 g/cm^3, 43998 reflections measured (4.564° ≤ 2Θ ≤ 158.276°), 4199 unique (R_{int} = 0.0438, R_{sigma} = 0.0209) which were used in all calculations. The final R_1 was 0.0299 (I > 2σ(I)) and wR_2 was 0.0723 (all data).

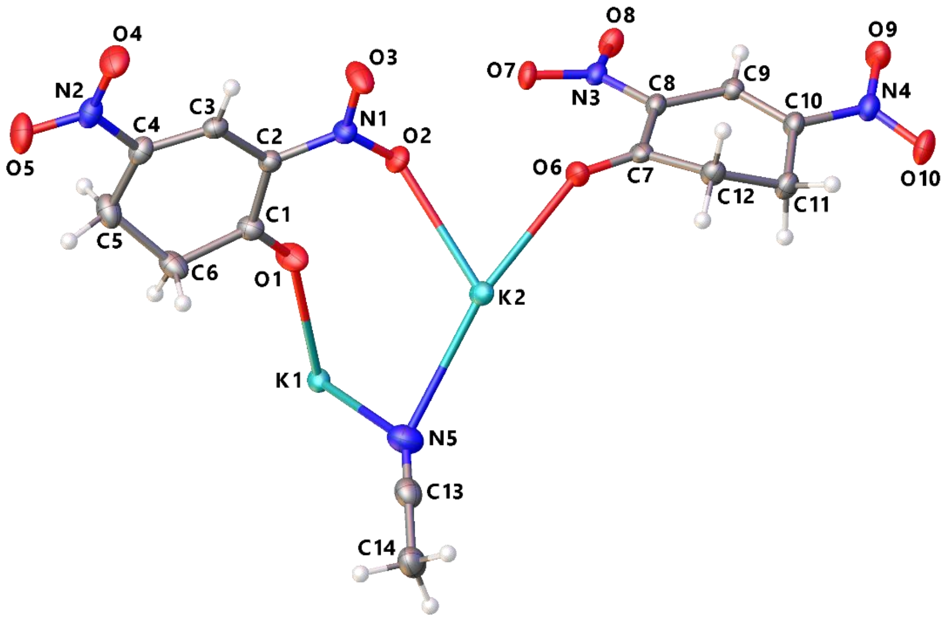

Figure S70. A molecular drawing of **14b** shown with 50% probability ellipsoids.

Table S9. Crystal data and structure refinement for 14b.

Identification code	14b
Empirical formula	$[K(C_6H_5N_2O_5)]_2$ MeCN
Formula weight	489.49
Temperature/K	100.00
Crystal system	orthorhombic
Space group	Pbca
a/Å	13.6247(17)
b/Å	7.3982(7)
c/Å	38.728(4)
α/°	90
β/°	90
γ/°	90
Volume/Å^3	3903.7(7)
Z	8
ρ_{calc}g/cm^3	1.666
μ/mm^{-1}	4.913
F(000)	2000.0
Crystal size/mm^3	0.08 × 0.08 × 0.06
Radiation	Cu Kα (λ = 1.54178)
2Θ range for data collection/°	4.564 to 158.276
Index ranges	$-17 \leq h \leq 16$, $-9 \leq k \leq 8$, $-45 \leq l \leq 49$
Reflections collected	43998
Independent reflections	4199 [R_{int} = 0.0438, R_{sigma} = 0.0209]
Data/restraints/parameters	4199/0/281
Goodness-of-fit on F^2	1.071
Final R indexes [I>=2σ (I)]	R_1 = 0.0299, wR_2 = 0.0714
Final R indexes [all data]	R_1 = 0.0316, wR_2 = 0.0723
Largest diff. peak/hole / e Å^{-3}	0.32/-0.24

References

(1) Park, S. Y.; Yamane, K.; Adachi, S. I.; Shiro, Y.; Weiss, K. E.; Maves, S. A.; Sligar, S. G. Thermophilic Cytochrome P450 (CYP119) from Sulfolobus Solfataricus: High Resolution Structure and Functional Properties. *J. Inorg. Biochem.* **2002**, *91*, 491–501.

(2) Kille, S.; Acevedo-Rocha, C. G.; Parra, L. P.; Zhang, Z. G.; Opperman, D. J.; Reetz, M. T.; Acevedo, J. P. Reducing Codon Redundancy and Screening Effort of Combinatorial Protein Libraries Created by Saturation Mutagenesis. *ACS Synth. Biol.* **2013**, *2*, 83–92.

(3) Berry, E. A.; Trumpower, B. L. Simultaneous Determination of Hemes a, b, and c from Pyridine Hemochrome Spectra. *Anal. Biochem.* **1987**, *161*, 1–15.

(4) Perkins, L. J.; Weaver, B. R.; Buller, A. R.; Burstyn, J. N. De Novo Biosynthesis of a Nonnatural Cobalt Porphyrin Cofactor in E. Coli and Incorporation into Hemoproteins. *Proc. Natl. Acad. Sci. U. S. A.* **2021**, *118,* e2017625118

(5) Gibson, D. G.; Young, L.; Chuang, R. Y.; Venter, J. C.; Hutchison, C. A.; Smith, H. O. Enzymatic Assembly of DNA Molecules up to Several Hundred Kilobases. *Nat. Methods* **2009**, *6*, 343–345.

(6) Johnson, K. *Kinetic Analysis for the New Enzymology: Using Computer Simulation to Learn Kinetics and Solve Mechanisms*; KinTek Corporation.

(7) Buzsaki, S. R.; Mason, S. M.; Kattamuri, P. V.; Serviano, J. M. I.; Rodriguez, D. N.; Wilson, C. V.; Hood, D. M.; Ellefsen, J. D.; Lu, Y. C.; Kan, J.; West, J. G.; Miller, S. J.; Holland, P. L. Fe/Thiol Cooperative Hydrogen Atom Transfer Olefin Hydrogenation: Mechanistic Insights That Inform Enantioselective Catalysis. *J. Am. Chem. Soc.* **2024**, *146*, 17296–17310.

(8) Wilson, C. V.; Holland, P. L. Mechanism of Alkene Hydrofunctionalization by Oxidative Cobalt(Salen) Catalyzed Hydrogen Atom Transfer. *J. Am. Chem. Soc.* **2024**, *146*, 2685–2700.

(9) De Carvalho, G. S. G.; Chagas, L. H.; Fonseca, C. G.; De Castro, P. P.; Sant’Ana, A. C.; Leitão, A. A.; Amarante, G. W. Nb2O5 Supported on Mixed Oxides Catalyzed Oxidative and Photochemical Conversion of Anilines to Azoxybenzenes. *New J. Chem.* **2019**, *43*, 5863–5871.

(10) Majtan, T.; Freeman, K. M.; Smith, A. T.; Burstyn, J. N.; Kraus, J. P. Purification and Characterization of Cystathionine β-Synthase Bearing a Cobalt Protoporphyrin. *Arch. Biochem. Biophys.* **2011**, *508*, 25–30.

(11) Neese, F. The ORCA Program System. *Wiley Interdiscip. Rev. Comput. Mol. Sci.* **2012**, *2*, 73–78.

(12) Perdew, J. P.; Burke, K.; Ernzerhof, M. Generalized Gradient Approximation Made Simple. *Phys. Rev. Lett.* **1996**, *77*, 3865–3868.

(13) Weigend, F.; Ahlrichs, R. Balanced Basis Sets of Split Valence, Triple Zeta Valence and Quadruple Zeta Valence Quality for H to Rn: Design and Assessment of Accuracy. *Phys. Chem. Chem. Phys.* **2005**, *7*, 3297–3305.

(14) Weigend, F. Accurate Coulomb-Fitting Basis Sets for H to Rn. *Phys. Chem. Chem. Phys.* **2006**, *8*, 1057–1065.

(15) Grimme, S.; Antony, J.; Ehrlich, S.; Krieg, H. A Consistent and Accurate Ab Initio Parametrization of Density Functional Dispersion Correction (DFT-D) for the 94 Elements H-Pu. *J. Chem. Phys.* **2010**, *132*, 154104.

(16) Yanai, T.; Tew, D. P.; Handy, N. C. A New Hybrid Exchange-Correlation Functional Using the Coulomb-Attenuating Method (CAM-B3LYP). *Chem. Phys. Lett.* **2004**, *393*, 51–57.

(17) Hellweg, A.; Hättig, C.; Höfener, S.; Klopper, W. Optimized Accurate Auxiliary Basis Sets for RI-MP2 and RI-CC2 Calculations for the Atoms Rb to Rn. *Theor. Chem. Acc.* **2007**, *117*, 587–597.

(18) Shulman, G. I.; Spiegel, D. A. Therapeutic DNP Derivatives and Methods Using Same. WO 2015/031598 A2, March 5, 2015. https://worldwide.espacenet.com/patent/search?q=pn%3DWO2015031598A2 (accessed 2024-10-17).

(19) Bahrami, F.; Zhao, Y. Rational Design and Synthesis of an Artificial Enzyme for SN2 Reactions through Micellar Imprinting. *Org. Lett.* **2024**, *26*, 73–77.

(20) Wang, Y.; Wang, Q.; Wu, L.; Jia, K.; Wang, M.; Qiu, Y. Electroreduction of Unactivated Alkenes Using Water as Hydrogen Source. *Nat. Commun.* **2024**, *15*, 2780.

(21) Zhang, Q.; Peng, M.; Gao, Z.; Guo, W.; Sun, Z.; Zhao, Y.; Zhou, W.; Wang, M.; Mei, B.; Du, X. L.; Jiang, Z.; Sun, W.; Liu, C.; Zhu, Y.; Liu, Y. M.; He, H. Y.; Li, Z. H.; Ma, D.; Cao, Y. Nitrogen-Neighbored Single-Cobalt Sites Enable Heterogeneous Oxidase-Type Catalysis. *J. Am. Chem. Soc.* **2023**, 145, 4166–417.

(22) Fischer, O.; Heinrich, M. R. 2-Fluoro-5-Nitrophenyldiazonium: A Novel Sanger-Type Reagent for the Versatile Functionalization of Alcohols. *Chemistry - A European Journal* **2021**, *27* (17), 5417–5421. https://doi.org/10.1002/chem.202100187.

(23) Nakayama, Y.; Maser, M. R.; Okita, T.; Dubrovskiy, A. V.; Campbell, T. L.; Reisman, S. E. Total Synthesis of Ritterazine B. *J. Am. Chem. Soc.* **2021**, *143*, 4187–4192.

(24) Sinha, A.; Khatua, S.; Bhattacharjee, M. Synthesis and Structure of [Ru(PPh3)2(Bipy)(MeCN)Cl][BPh4] and It's Catalytic Property towards Regioselective and Stereoselective Allylation of Phenols. *J. Organomet. Chem.* **2014**, *770*, 116–120.

(25) Lv, X.; Liu, C.; Chen, Y.; Wang, D.; Yu, P.; Jin, M. Y.; Xu, C. Highly Enantioselective Dihydroxylation of 1,1-Disubstituted Aliphatic Alkenes Enabled by Orchestrated Noncovalent π-Interactions. *Org. Lett.* **2024**, *26*, 1399–1404.

(26) Zhao, K.; Knowles, R. R. Contra-Thermodynamic Positional Isomerization of Olefins. *J. Am. Chem. Soc.* **2022**, *144*, 137–144.

(27) Arndt, T.; Raina, A.; Breugst, M. Iodine-Catalyzed Claisen-Rearrangements of Allyl Aryl Ethers and Subsequent Iodocyclizations. *Chem. Asian J.* **2023**, *18*, e202201279.

(28) Templ, J.; Schnürch, M. Allylation of C-, N-, and O-Nucleophiles via a Mechanochemically-Driven Tsuji–Trost Reaction Suitable for Late-Stage Modification of Bioactive Molecules. *Angew. Chem.* **2024**, *63*, e202314637.

(29) Arndt, T.; Raina, A.; Breugst, M. Iodine-Catalyzed Claisen-Rearrangements of Allyl Aryl Ethers and Subsequent Iodocyclizations. *Chem. Asian J.* **2023**, *18*, e202201279.

(30) Krause, L.; Herbst-Irmer, R.; Sheldrick, G. M.; Stalke, D. Comparison of Silver and Molybdenum Microfocus X-Ray Sources for Single-Crystal Structure Determination. *J. Appl. Crystallogr.* **2015**, *48*, 3–10.

(31) Dolomanov, O. V.; Bourhis, L. J.; Gildea, R. J.; Howard, J. A. K.; Puschmann, H. OLEX2: A Complete Structure Solution, Refinement and Analysis Program. *J. Appl. Crystallogr.* **2009**, *42*, 339–341.

(32) Sheldrick, G. M. A Short History of SHELX. *Acta Crystallogr. A* ***2008**, 64, 112–122*
